# From aniline to phenol: carbon-nitrogen bond activation via uranyl photoredox catalysis

**DOI:** 10.1093/nsr/nwab156

**Published:** 2021-08-20

**Authors:** Deqing Hu, Yilin Zhou, Xuefeng Jiang

**Affiliations:** Shanghai Key Laboratory of Green Chemistry and Chemical Process, School of Chemistry and Molecular Engineering, East China Normal University, Shanghai 200062, China; Shanghai Key Laboratory of Green Chemistry and Chemical Process, School of Chemistry and Molecular Engineering, East China Normal University, Shanghai 200062, China; Shanghai Key Laboratory of Green Chemistry and Chemical Process, School of Chemistry and Molecular Engineering, East China Normal University, Shanghai 200062, China; State Key Laboratory of Organometallic Chemistry, Shanghai Institute of Organic Chemistry, Chinese Academy of Sciences, Shanghai 200032, China; State Key Laboratory of Elemento-Organic Chemistry, Nankai University, Tianjin 300071, China

**Keywords:** C−N bond activation, C−O bond formation, uranyl cations, photoredox catalysis

## Abstract

Carbon-nitrogen bond activation, via uranyl photoredox catalysis with water, enabled the conversion of 40 protogenetic anilines, 8 N-substituted anilines and 9 aniline-containing natural products/pharmaceuticals to the corresponding phenols in an ambient environment. A single-electron transfer process between a protonated aniline and uranyl catalyst, which was disclosed by radical quenching experiments and Stern-Volmer analysis, facilitated the following oxygen atom transfer process between the radical cation of protonated anilines and uranyl peroxide originating from water-splitting. ^18^O labeling and ^15^N tracking unambiguously depicted that the oxygen came from water and amino group left as ammonium salt. The 100-fold efficiency of the flow operation demonstrated the great potential of the conversion process for industrial synthetic application.

## Teaser

From aniline to phenol: carbon-nitrogen bond activation and carbon-oxygen bond formation via uranyl photoredox catalysis with water at ambient environment.

## INTRODUCTION

**Scheme 1.**
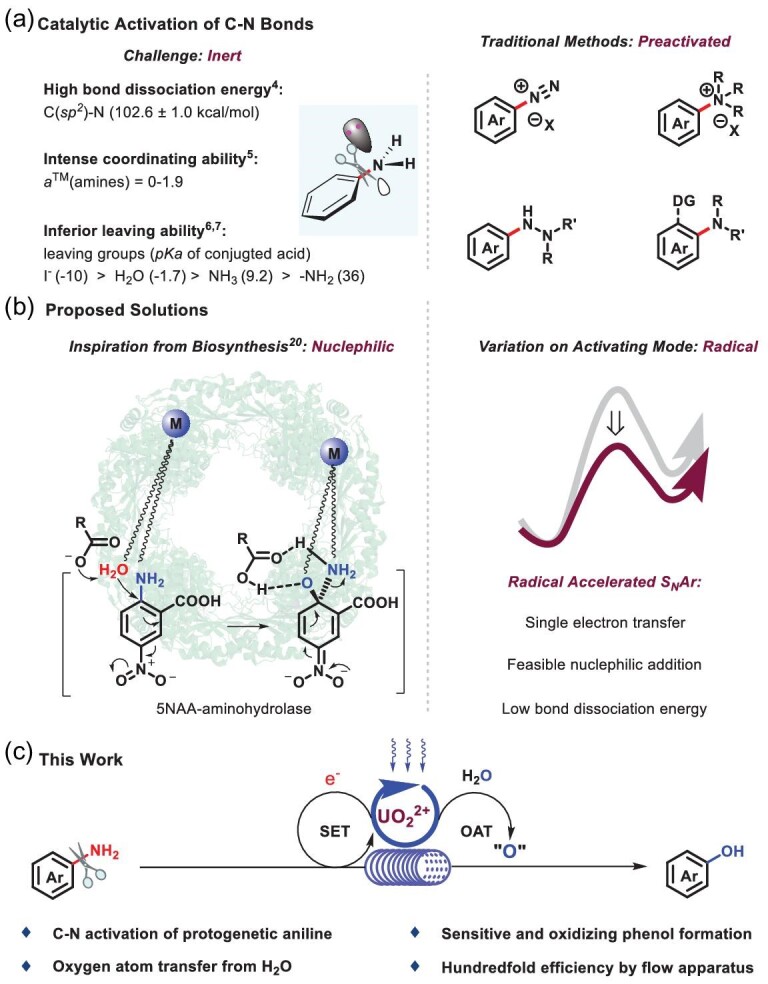
Activation of carbon-nitrogen bond in aniline. (a) Catalytic activation of C−N bonds. (b) Proposed solutions. (c) This work.

C*sp^2^*−N bond activation remains an intractable challenge with regard to the transformation of inert chemical bonds [1–3], due to the high bond dissociation energy [C−N BDE (PhNH_2_) = 102.6 ± 1.0 kcal/mol] [4], the intense coordinating ability [*a*^TM^ (amines) = 0–1.9 vs. *a*^TM^ (ethers) = −2.5–0.1] [5] and the inferior leaving ability [*pKa* (−NH_2_) = 36] [6,7] (Scheme 1a, left). Conventionally, prefunctionalization is the essential solution for C−N bond transformation in anilines, such as up-front operations to diazonium salts [8–10], quaternary ammonium salts [11–13], hydrazines and amines with vicinal directing groups [14–18] (Scheme 1a, right). Akiyama *et al.* reported the pioneering progress of C*sp^2^*−N bond cleavage of undecorated aniline with stoichiometric palladium acetate [19]. Remarkably, the amino of 5-nitroanthranilic acid (5NAA), associated with tryptophan biosynthesis in the living system, was transformed into a hydroxyl group that was catalyzed by 5NAA-aminohydrolase at body temperature with water (Scheme 1b, left), which shed light on C−N activation [20]. Encouragingly, Nicewicz realized C−O bond activation via nucleophilic aromatic substitution accelerated by cation radical (Scheme 1b, right) [21–23]. With the development of the photocatalysis technique [24–28], it has been found that uranyl catalysts are characterized by a ligand-to-metal charge transfer (LMCT) process and show superior oxidative ability [*E^ox^* = +2.60 V vs. saturated calomel electrode (SCE)] [29–36]. Following our previous work on uranyl photoredox catalysis [37,38], C−N bond activation in protogenetic anilines was realized to generate corresponding phenols at ambient environment with water via a synergistic process of single electron transfer (SET) and oxygen atom transfer (OAT) (Scheme 1c).

## RESULTS AND DISCUSSION

We commenced the study with 4-(*tert*-butyl)aniline as reactant and uranyl nitrate hexahydrate as photoredox catalyst irradiated by blue light (460 nm) at room temperature. Lewis and Brønsted acids, considered as coactivators (Table 1, entries 1–8), were added to the system respectively, in which trifluoroacetic (TFA) supplied the optimal result with 85% isolated yield of the desired product. Compared with uranyl ion, Ir[dF(CF_3_)ppy]_2_dtbpy·PF_6_ [*E_1/2_* = +1.21 V vs. SCE] [39], Ru(bpy)_3_Cl_2_ · 6H_2_O [*E_1/2_* = +0.77 V vs. SCE] [39] and riboflavintetra-acetate [*E* = +1.67 V vs. SCE] [26] were inefficient for the transformation (Table 1, entry 9). Solvents also played a crucial role; acetonitrile was the best choice (Table 1, entries 10 and 11). Control experiments further demonstrated that UO_2_(NO_3_)_2_ · 6H_2_O, TFA and light were all essential conditions (Table 1, entries 12–14).

**Table 1.** Optimization conditions. General conditions: **1a** (0.2 mmol), UO_2_(NO_3_)_2_·6H_2_O (4 mol%), acid (0.2 mmol) and H_2_O (0.6 mmol) were stirred in solvent (2 mL) at room temperature for 24 hours under blue light (460 nm). ^1^H nuclear magnetic resonance (NMR) yields with CH_2_Br_2_ as the internal standard. (a) Acid (30 mmol%). (b) Isolated yields. (c) Ir[dF(CF_3_)ppy]_2_dtbpy·PF_6_, Ru(bpy)_3_Cl_2_·6H_2_O or Riboflavin tetraacetate instead of UO_2_(NO_3_)_2_·6H_2_O. (d) Without UO_2_(NO_3_)_2_·6H_2_O. (e) No light. NR = no reaction.

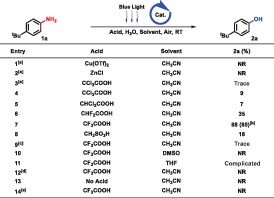

**Scheme 2.**
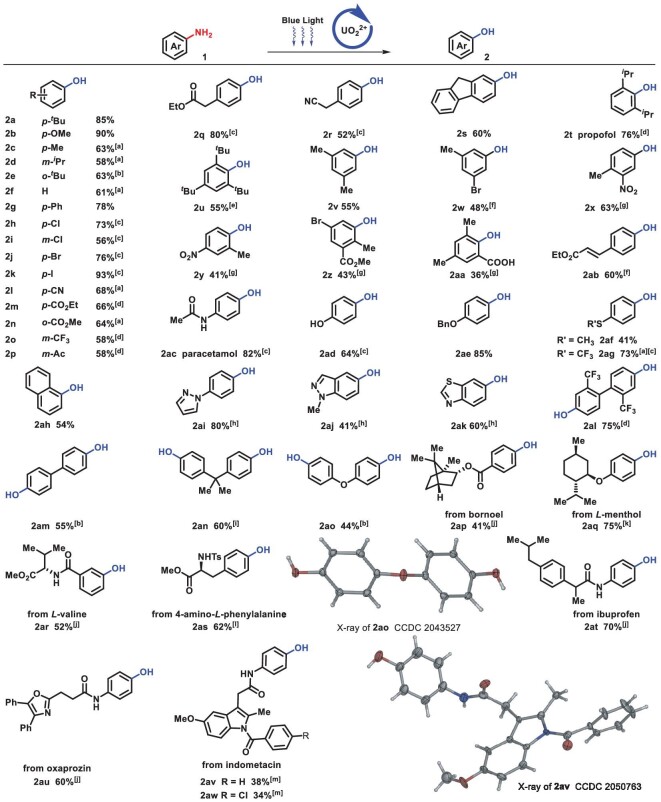
Scope of anilines. Standard conditions: **1** (0.2 mmol), UO_2_(NO_3_)_2_·6H_2_O (4 mol%), TFA (0.2 mmol) and H_2_O (0.6 mmol) were stirred in CH_3_CN (2 mL) at room temperature under blue LED (460 nm) in the air, isolated yields. (a) UO_2_(OAc)_2_·2H_2_O (4 mol%), N_2_. (b) TFA (0.4 mmol). (c) HFIP (2 mL). (d) TFA (0.4 mmol), HFIP (2 mL), N_2_. (e) CH_3_NO_2_ (2 mL). (f) UO_2_(OAc)_2_·2H_2_O (4 mol%), HFIP (2 mL), N_2_. (g) UO_2_(OAc)_2_·2H_2_O (8 mol%), TFA (0.4 mmol), HFIP (2 mL), N_2_. (h) UO_2_(NO_3_)_2_·6H_2_O (8 mol%), TFA (0.4 mmol), HFIP (2 mL), N_2_. (i) TFA (0.4 mmol), CH_3_NO_2_ (2 mL). (j) **1** (0.1 mmol), HFIP (2 mL), N_2_. (k) **1** (0.1 mmol), HFIP (1 mL), N_2_. (l) **1** (0.1 mmol), CH_3_NO_2_ (2 mL), N_2_. (m) **1** (0.1 mmol), CH_3_CN (2 mL), N_2_.

**Scheme 3.**
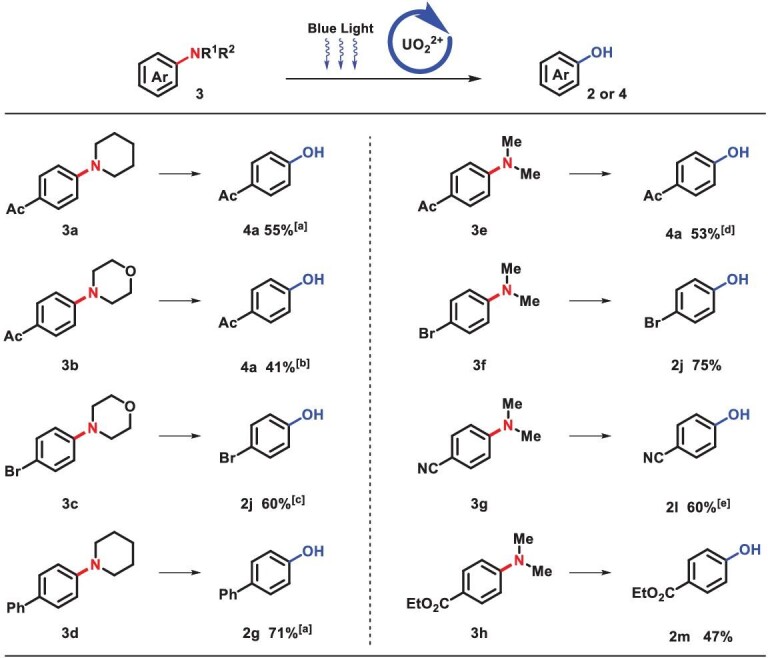
Transformation of tertiary anilines. Standard conditions: **1** (0.2 mmol), UO_2_(NO_3_)_2_·6H_2_O (4 mol%), TFA (0.2 mmol) and H_2_O (0.6 mmol) were stirred in HFIP (2 mL) at room temperature under blue light (460 nm), isolated yields. (a) UO_2_(NO_3_)_2_·6H_2_O (8 mol%), TFA (0.4 mmol), N_2_. (b) UO_2_(OAc)_2_·2H_2_O (4 mol%), TFA (0.4 mmol). (c) UO_2_(NO_3_)_2_·6H_2_O (8 mol%). (d) N_2_. (e) UO_2_(OAc)_2_·2H_2_O (8 mol%), TFA (0.6 mmol).

Under optimal conditions, the scope of undecorated anilines was investigated comprehensively (Scheme 2). Diverse anilines with electron-rich substitutions produced corresponding phenols in an effective way, despite the fact that they were prone to being oxidized (2a–2e). Subsequently, we found that electron-neutral substituted aniline could be transformed smoothly (2f and 2g). For electron-deficient substrates (2h–2p), 1,1,1,3,3,3-hexafluoro-2-propanol (HFIP) was found to be a more helpful solvent, due to its ability to stabilize cation radicals [40]. Notably, halides were well tolerated under this condition (2h–2k), especially the commonly light-sensitive iodo-group (2k). Easily hydrolytic cyano- (2l) and carboxylic ester (2m and 2n) were preserved in this water-involving reaction. Furthermore, various active C−H bonds were well tolerated, such as acetyl (2p) and dually activated benzyl (2q–2s). Due to steric and electronic effects, polysubstituted phenol synthesis is always challenging but imperative, and phenols with 2,6-diisopropyl- (2t), 2,4,6-tritertbutyl (2u) and 3,5-dimethyl (2v) groups were successfully achieved in our system with sterically bulky hinderance. Besides, multiple substituents with distinct electronic properties, such as bromo- (2w), nitro- (2x and 2y), carboxylic ester (2z) and acid (2aa) groups, were compatible. Michael acceptor containing motif (2ab) was well preserved. Remarkably, when only one amino group of *p*-phenylenediamine was protected, highly selective conversion of the unprotected amino group occurred, which yielded 82% paracetamol, a clinically applied antipyretic and analgesic drug (2ac). Undecorated or substituted hydroxyl (2ad and 2ae) and thioethers with electron-rich or -deficient substituent group (2af and 2ag) were all compatible during C−N activation. The amino group on the condensed cyclics (2ah) and heterocylics (2ai-2ak) was smoothly activated, in spite of high electron density or coordinating effect. Moreover, a series of diphenylaminos could be transformed to diphenols successfully (2al–2ao). X-ray diffraction of 2ao (cambridge crystallographic data centre (CCDC) 2043527) further confirmed its structure. The applicability and compatibility of C−N activation were demonstrated in natural products and pharmaceuticals. Terpenoid (borneol and menthol) derivatives and amino-acid-containing molecules (valine and phenylalanine) were transformed into corresponding phenols (2ap–2as) in moderate yields. Subsequently, ibuprofen, a non-steroidal anti-inflammatory drug, was proven to have 70% yield (2at). Late-stage modification of oxaprozin (2au) and indometacin (2av and 2aw) were achieved in spite of highly active sites on heterocycles. X-ray diffraction (CCDC 2050763) further confirmed the structure of 2av. Phenylpiperidine, *N*, *N*-dimethylanilines and phenyl-morpholine analogous yielded corresponding phenol efficiently, fulfilling the tough target of traditional cross coupling [18] (Scheme 3).

**Scheme 4.**
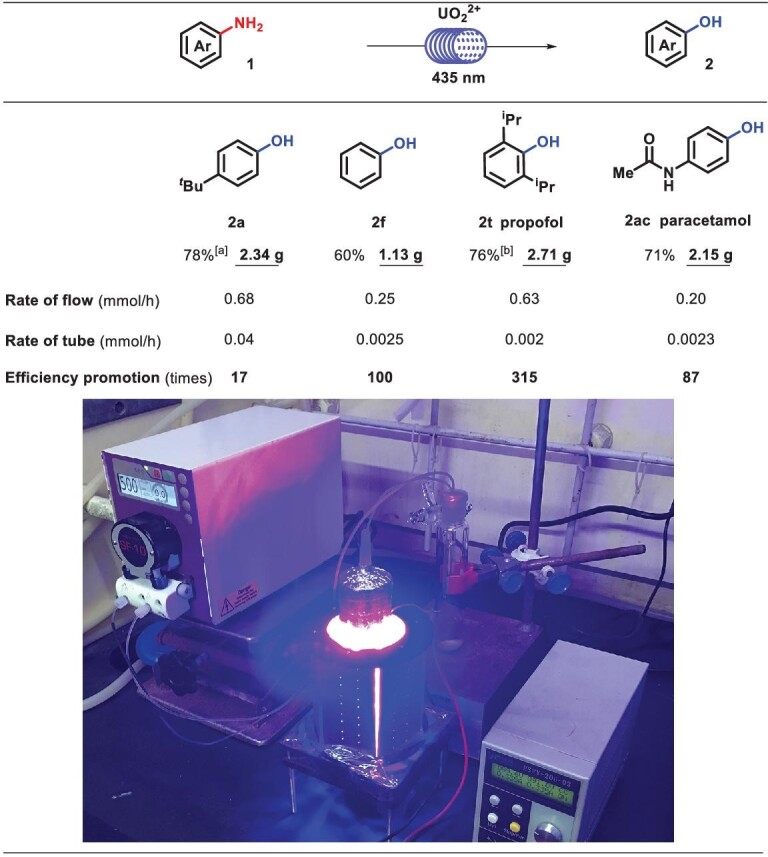
Flow reaction. Standard conditions: 1 (20 mmol), UO_2_(NO_3_)_2_^.^6H_2_O (2 mol%), TFA (40 mmol) and H_2_O (60 mmol) were stirred in CH_3_CN/HFIP (25 mL/25 mL) at room temperature irradiated with blue light (435 nm) in the air, isolated yields. (a) TFA (60 mmol), HFIP (50 mL). (b) UO_2_(NO_3_)_2_^.^6H_2_O (3 mol%), TFA (60 mmol), CH_3_CN/HFIP (25 mL/45 mL).

To further demonstrate the application potential of anilines, flow reactions were conducted, which were more efficient (0.68 mmol/h for 2a, 20 mmol scale) than those done with parallel reactors (0.04 mmol/h for 2a, 10 mmol scale). It is noteworthy that clinically applied pharmaceuticals, i.e. propofol and paracetamol by flow reactions could be, at most, 315 times as efficient as by tube operation, though the residue volume of flow pipeline was only ∼4.7 mL (<1/10 of the total volume) (Scheme 4).

**Scheme 5.**
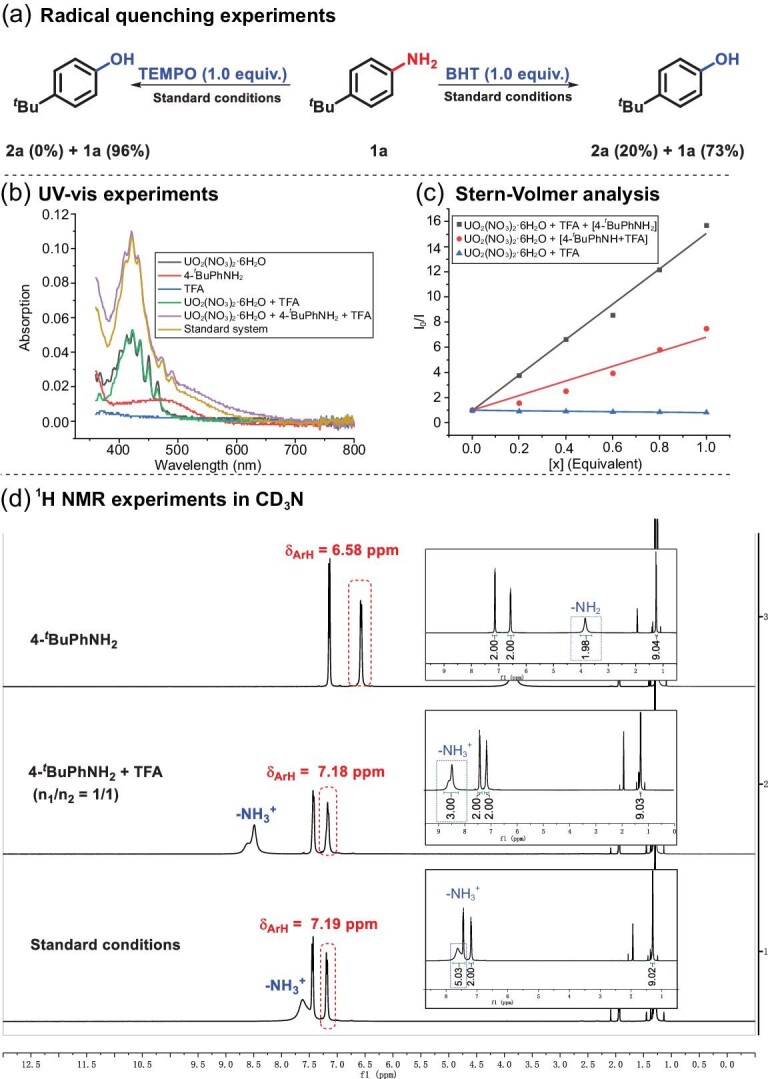
Mechanistic studies of SET mode. (a) Radical quenching experiments. (b) UV-vis experiments. (c) Stern-Volmer analysis. (d) ^1^H NMR experiments in CD_3_CN.

The mechanistic study was carried out to understand the process. Firstly, radical quenching experiments with 2,2,6,6-tetramethyl-1-piperinedinyloxy (TEMPO) and butylated hydroxytoluene (BHT) suggested the radical property of this system (Scheme 5a, supplementary information (SI), Section IV-1). UV-vis absorption between catalyst and each component demonstrated that uranyl salt served as a photosensor. The addition of aniline salt to uranyl solution enhanced the absorption efficiency, illustrating the interaction between the uranyl species and aniline complex (Scheme 5b, SI, Section IV-2). Active uranyl cation was quenched by aniline/TFA complex, as detected by Stern-Volmer analysis (Scheme 5c, SI, Section IV-3), and energy transfer process was ruled out considering the lower value of the lowest triplet energy of the uranyl cation (*E_T_* = 58.5 kcal/mol) compared with that of anilines [41,42]. Meanwhile, the ammonium salt was instantaneously generated, as was monitored by ^1^H NMR experiments before C−N bond activation (Scheme 5d, SI, Section IV-4). Furthermore, the quenching effect between uranyl species and protonated anilines was much stronger than in those with Ir[dF(CF_3_)ppy]_2_dtbpy^.^PF_6_, Ru(bpy)_3_Cl_2_^.^6H_2_O and Riboflavin tetraacetate, revealing the unique interaction property between uranyl ion and substrate in the transformation (SI, Section IV-3).

**Scheme 6.**
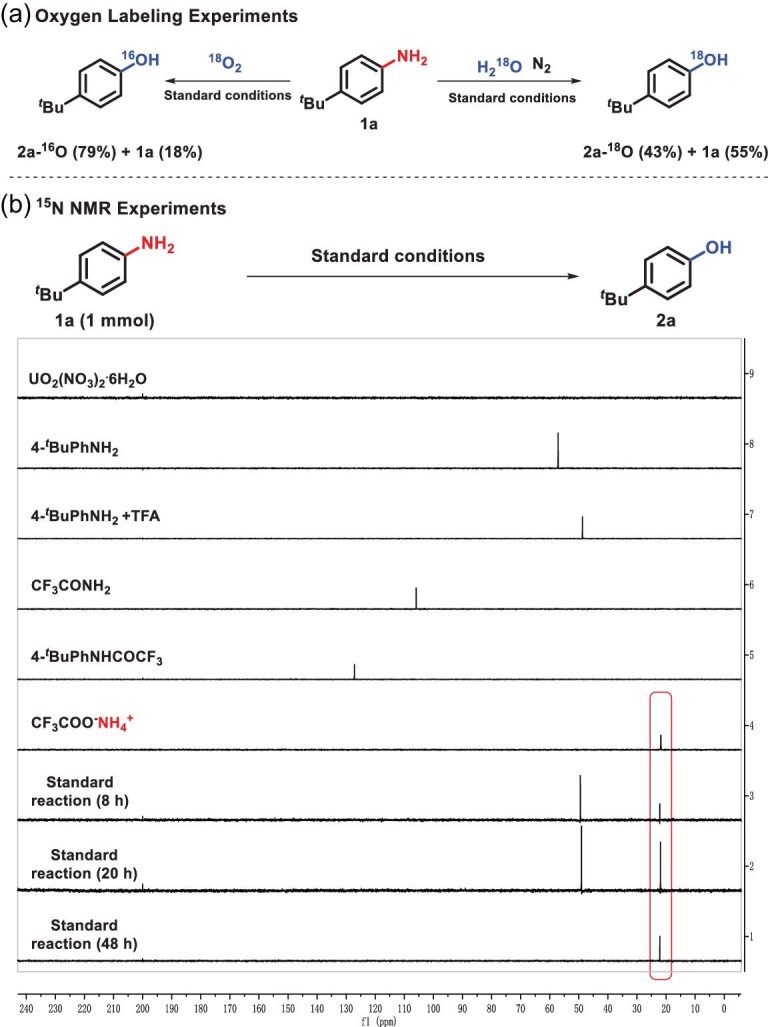
Mechanistic studies. (a) Oxygen labeling experiments. (b) ^15^N NMR experiments.

Labeling experiments with H_2_^18^O and ^18^O_2_ unambiguously demonstrated that the oxygen atom of the product phenols originated from water rather than oxygen atmosphere (Scheme 6a, SI, Section IV-5). According to previous studies [38,43,44], uranyl peroxide complexes were obtained from uranyl photolysis of water, which is responsible for the oxygen atom transfer. ^15^N NMR tracking experiments showed that only ammonium trifluoroacetate was obtained, which indicated that the amino group on anilines left in the form of ammonia followed by neutralization with TFA (Scheme 6b, SI, Section IV-6). In addition, both on-off experiments (SI, Section IV-6) and the quantum yield of 8.4 (SI, Section IV-7) demonstrated the existence of a radical chain propagation process during the transformation.

**Scheme 7.**
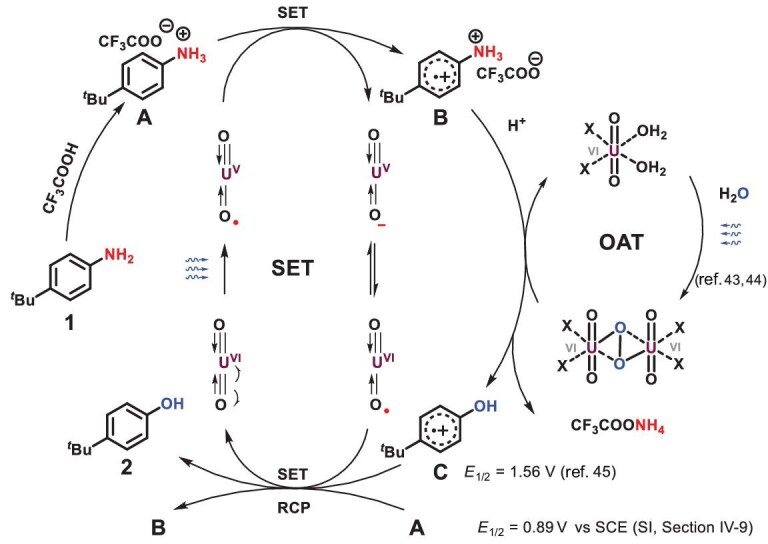
Proposed mechanism.

Based on the mechanistic study, a possible reaction pathway was depicted as shown in Scheme 7. Under blue light, uranyl photoredox catalysis was stimulated and generated ^*^UO_2_^2+^ through the LMCT process. Then, the single electron transfer process between ^*^UO_2_^2+^ and protonated anilines **A** brought forth UO_2_^+^ and radical cation **B**. Another uranyl peroxide dimer was generated from water-splitting [43,44], capturing **B** with C−O bond formation and C−N bond fracture to get the radical cation of phenol **C**. Single electron transfer between **C** and UO_2_^+^ afforded the desired product **2** and regenerated the catalyst. Meanwhile, the radical chain propagation process was also in progress during this transformation owing to the higher oxidation potential of intermediate **C** (*E_1/2_* = 1.56 V) [45] compared with protonated anilines **A** (*E_1/2_* = 0.89 V).

## CONCLUSION

In summary, oxygen atom transfer from water to organic molecules via uranyl photoredox catalysis was discovered in photoredox circulation. Accordingly, C−N bond activation in undecorated anilines was systematically established at ambient conditions, generating a series of sensitive and fragile phenols. The 100-fold efficiency of the flow set-up indicated the industrial application potential of the strategy. Radical trapping experiments, Stern-Volmer analysis and ^1^H NMR experiments demonstrated the interaction between active uranyl species and protonated anilines. Further studies in uranyl catalysis are on-going in our laboratory.

## Supplementary Material

nwab156_Supplemental_Files
