# Supplementary material for: From aniline to phenol: carbon-nitrogen bond activation via uranyl photoredox catalysis
Source: Natl Sci Rev. 2021 Aug 20;9(6):nwab156. doi: 10.1093/nsr/nwab156 (PMC9283103; doi:10.1093/nsr/nwab156)
Supplement: nwab156_Supplemental_Files [file nwab156_supplemental_files.zip › SI.pdf]

Supporting Information

From Aniline to Phenol: Carbon-Nitrogen Bond Activation via Uranyl Photoredox Catalysis

Deqing Hu,[†] Yilin Zhou,[†] Xuefeng Jiang*[,†,‡,§]

[†]Shanghai Key Laboratory of Green Chemistry and Chemical Process, School of Chemistry and Molecular Engineering, East China Normal University, 3663 North Zhongshan Road, Shanghai 200062, P. R. China.

[‡]State Key Laboratory of Organometallic Chemistry, Shanghai Institute of Organic Chemistry, Chinese Academy of Sciences, 345 Lingling Road, Shanghai 200032, P. R. China.

[§]State Key Laboratory of Elemento-Organic Chemistry, Nankai University, Tianjin 300071, P. R. China.

xfjiang@chem.ecnu.edu.cn

Index

I. **General Information**……………………………………….……….. **S2**

II. **Essential datas and Uranium Residue**……………………………… **S4**

III. **Supplementary Experiments.**……………………………………… **S5**

IV. **Mechanistic Studies**……….………………………………………... **S8**

V. **Procedures and Datas for Starting Materials in Fig. 2.**…………… **S26**

VI. **General Procedures for Fig. 2, 3, and 4.**………………………....... **S34**

VII. **Procedures and Datas for Fig. 2, 3, and 4.**………………………… **S36**

VIII. **X-ray Crystallography Analysis of Compounds 2ao, 2av**…………... **S63**

IX. **NMR Spectra**…………………………………………………….... **S65**

X. **References**……..………………………………………………....... **S168**

I. General Information

NMR Spectrum:

^{1}H and ^{13}C spectra were collected on 400 MHz or 500 MHz NMR spectrometers (Bruker AVANCE). Chemical shifts for protons are reported in parts per million (ppm) downfield and are referenced to residual protium in the NMR solvent ($CHCl_3$ = δ 7.26, DMSO = δ 2.50). Chemical shifts for carbon are reported in parts per million downfield and are referenced to the carbon resonances of solvent ($CHCl_3$ = δ 77.00, DMSO = δ 39.52). Dates are represented as follows: chemical shift, multiplicity (br = broad, s = singlet, d = double, t = triplet, q = quartet, m = multiplet-), coupling constants in Hertz (Hz), integration.

Mass Spectroscopy:

Mass spectra were in general recorded on a Shimadzu GCMS-QP2010 Ultra and a HP 5989A mass selective detector.

Chromatography:

Column chromatography was performed with silica gel (300 – 400 mesh ASTM).

IR:

SHIMADZU IR Tracer-100 Spectrometers.

Solvent:

Acetonitrile (CH_3CN), 1,1,1,3,3,3-Hexafluoro-2-propanol (HFIP), Nitromethane (CH_3NO_2) was bought and used without further purification.

II. Essential datas and Uranium Residue

1) Essential datas

Compounds	Oxidation potential
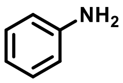	1.06 V vs Ag/AgCl[1]
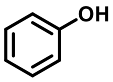	1.67 V vs SHE[2]

Compounds	Chemical Bond	BDE (kcal/mol)[3]
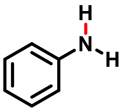	N-H	88.0 ± 2.0
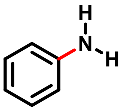	C-N	102.6 ± 1.0
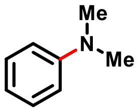	C*sp*^{2}-N	93.2 ± 2.5
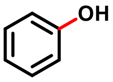	C-O	110.8 ± 1.0

2) Uranium Residue

a) Component Analysis

	^{234}U	^{235}U	^{238}U
natural uranium	0.0057%	0.72%	99.28%
depleted uranium	0.001%	0.2%	99.8%
$UO_2(NO_3)_2$	0	0	100%
radioactivity	strong	strong	weak (0~199 μSv)

1 mSv=1000 μSv

b) Radioactive Source and Radiation Quantity

from air, water, and food for people	250 μSv/year
x-ray inspection	100 μSv/time
luminous watch	20 μSv/year

c) Hazardous Effects of Radiation

0~199 μSv	no damage
200 μSv~1 mSv	minor damage
1~399 mSv	mild dizziness
400~1000 mSv	dizziness and vomiting
1001~4000 mSv	danger
>4000 mSv	extremely danger

d) Uranium Residue

compounds

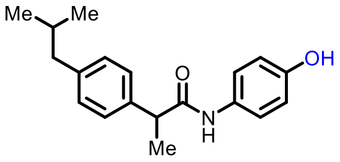

from ibuprofen, 2at

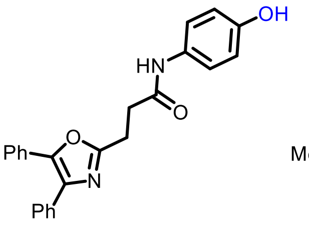

from oxaprozin, 2au

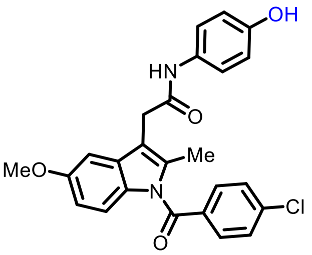

from indometacin, 2aw

	2at	2au	2aw
uranium residue (ppm)	0.9	6.4	2.4

The uranium residue of three highly functionalized molecules with some coordination groups were detected with HPLC-ICPMS [NexION 2000-(A-10)], which range from 0.9-6.4 ppm. Meanwhile, the uranium's average abundance in the Earth's crust and some common rocks such as granite and shales are 1.8-25 ppm.

III. Supplementary Experiments

a) Comparison between $UO_2(NO_3)_2\cdot6H_2O$ and $UO_2(OAc)_2\cdot6H_2O$

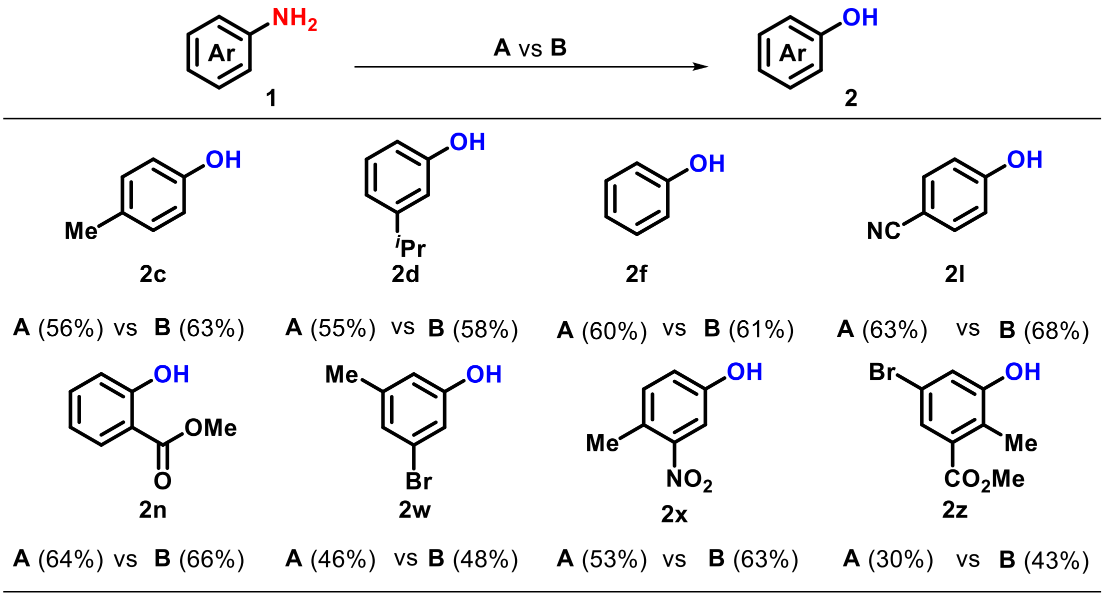

A: $UO_2(NO_3)_2{\cdot}6H_2O$; **B:** $UO_2(OAc)_2{\cdot}2H_2O$

The redox potential varied among different substrates and adjustment of uranium ions served to regulate the redox process for high efficiency of phenol obtained. Generally, corresponding differences on yields were within 10%.

b) Scope of Heterocyclic Amines

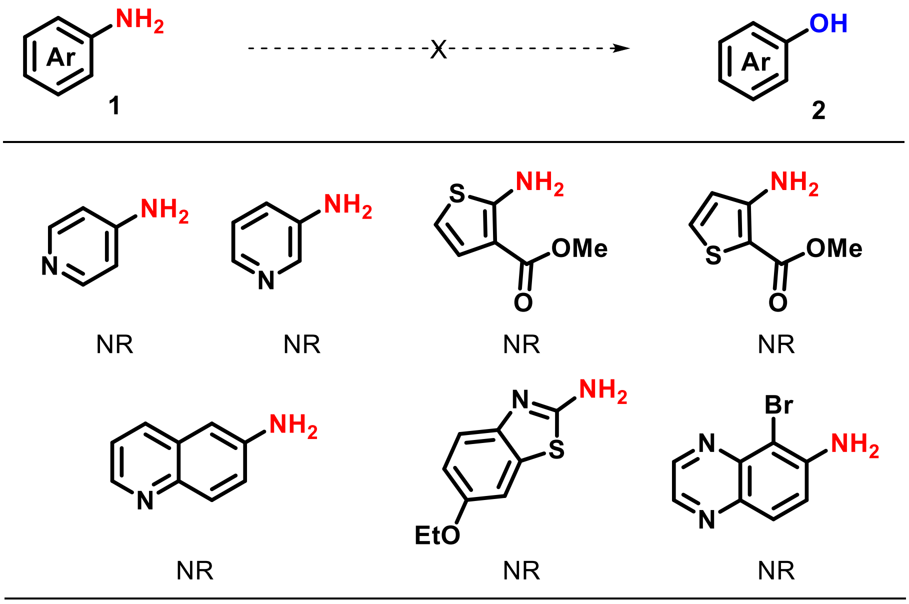

NR means no reaction.

Generally, the tolerance with heterocyclic amines (both 6-menbered and 5-menbered)

was poor.

c) Conversion with Different Light Wavelengths

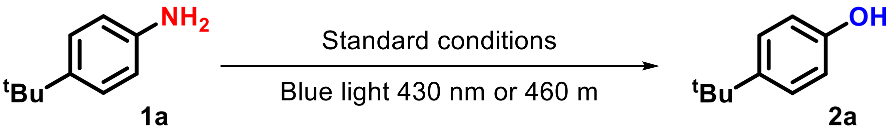

Wavelength (nm)	6 h	12 h
430	**2a** (80%), **1a** (15%)	**2a** (87%), **1a** (10%)
460	**2a** (66%), **1a** (28%)	**2a** (77%), **1a** (20%)

Yields by ^{1}H NMR with CH_2Br_2 as the internal standard.

460 nm LEDs is readily available and cheap. 430 nm LEDs were applied in flow scale-up reaction, which was more efficient.

d) Control Experiments

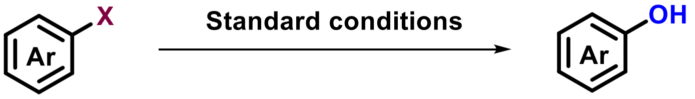

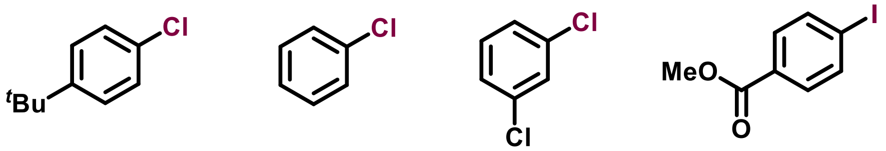

trace	trace	trace	trace

trace means less than 5%

There was no competing dehalogenation discovered, which was further confirmed through GC detection. In addition, when halogenated benzenes without amino group were investigated under standard conditions of C-N bond transformation, trace amounts of phenols were detected.

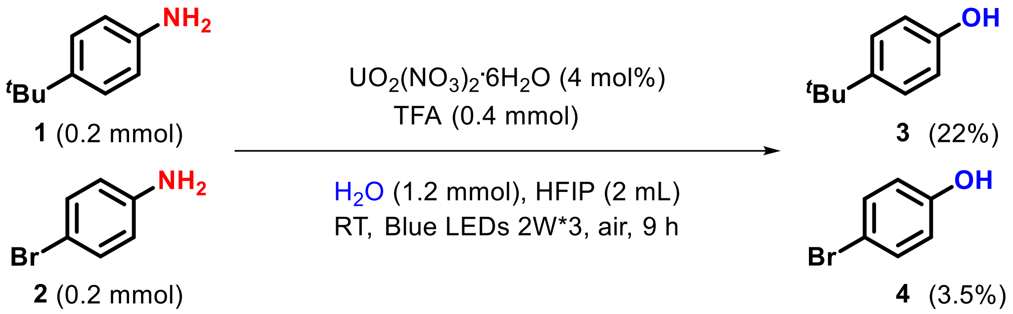

Yields by ^{1}H NMR with CH_2Br_2 as the internal standard.

Substrate with electron-rich group was found to be more efficient than that with electron-deficient one, which was also showed in our substrate table.

IV. Mechanistic Studies

1) Radical Quenching Experiments

All reactions were operated under standard conditions with extra TEMPO (1.0 equiv.) or BHT (1.0 equiv.). The result was detected by TLC and GC. Yields by ^{1}H NMR with CH_2Br_2 as the internal standard.

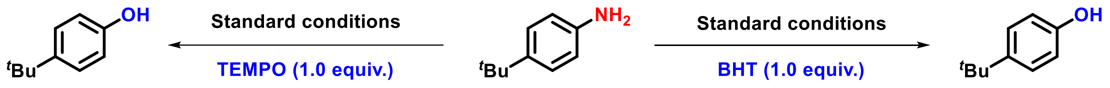

2a (0) + 1a (96%)   1a   2a (20%) + 1a (73%)

Radical trapping experiments revealed that 2,2,6,6-tertramethylpiperidine-1-oxyl (TEMPO), or 2,6-Di-tert-butyl-4-methylphenol (BHT) could efficiently quench the process of the cleavage of C-N bond.

2) Ultraviolet-Visible Absorption Experiments with $UO_2(NO_3)_2{\cdot}6H_2O$

Ultraviolet-visible absorption experiments were performed using a Shimadzu UV-2700 UV-visible spectrophotometer. In each experiment, the varying samples were combined in CH_3CN in screw-top 1.0 cm quartz cuvettes. The concentration of each component under standard conditions.

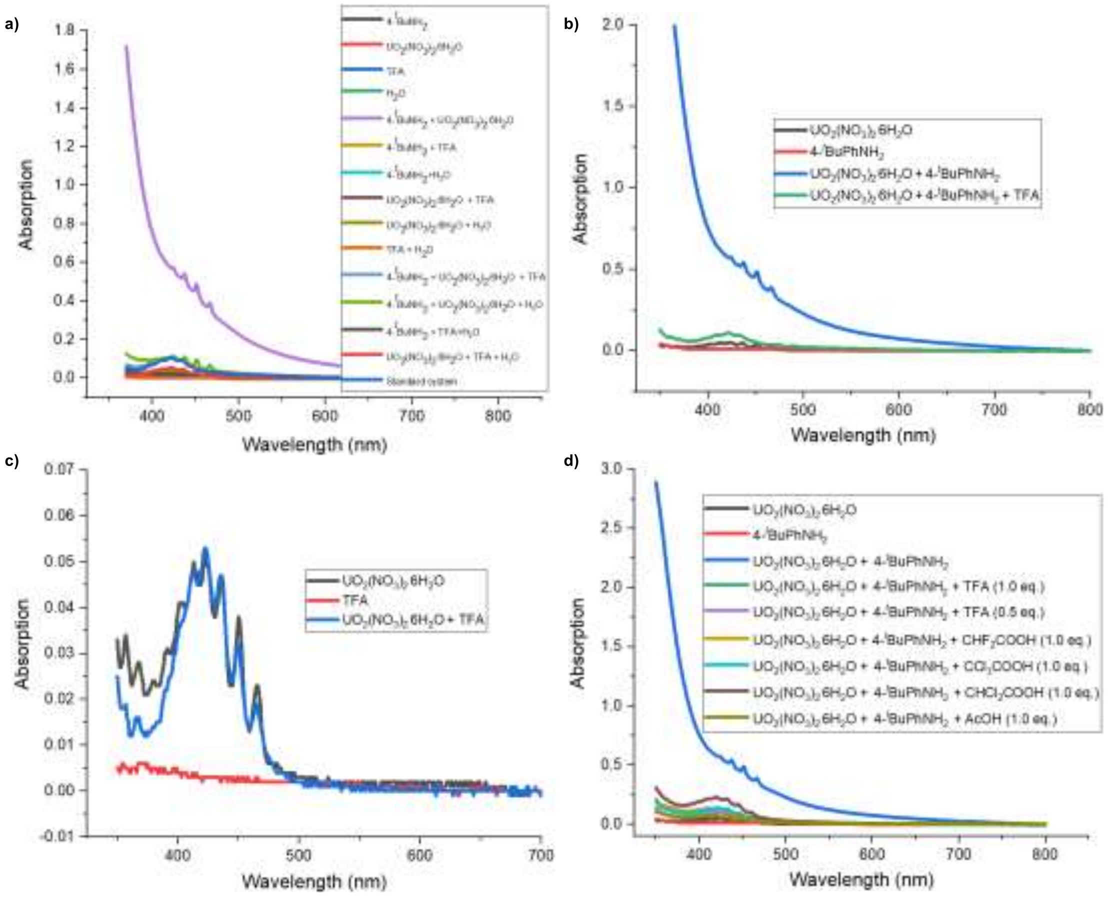

Figure S1: Ultraviolet-visible absorption experiments a), b), c), and d) in CH_3CN.

a), *ultraviolet-visible absorption of each reaction component indicated that $UO_2(NO_3)_2{\cdot}6H_2O$ served as photoredox catalysis in this system.* ***b), c), and d),*** *The ultraviolet-visible absorption of $UO_2(NO_3)_2{\cdot}6H_2O$ could be greatly enhanced by 4-^{t}BuPhNH$_2$, but no effect with TFA. In addition, the effect of 4-^{t}BuPhNH$_2$ on ultraviolet-visible absorption of $UO_2(NO_3)_2{\cdot}6H_2O$ was sharply reduced by the protonation with different Brøsted acids.*

3) Stern–Volmer Fluorescence Quenching Experiments

Stern–Volmer Fluorescence Quenching Experiments with $UO_2(NO_3)_2{\cdot}6H_2O$

Fluorescence quenching studies were performed using a Shimadzu RF-6000 Fluorescence Spectrophotometer. In each experiment, the photoredox catalyst and varying concentrations of quencher were combined in CH_3CN in screw-top 1.0 cm quartz cuvettes. For the emission quenching of $UO_2(NO_3)_2{\cdot}6H_2O$, the photoredox catalyst concentration was $2*10^{-3}$ M, the solution was irradiated at 424 nm.

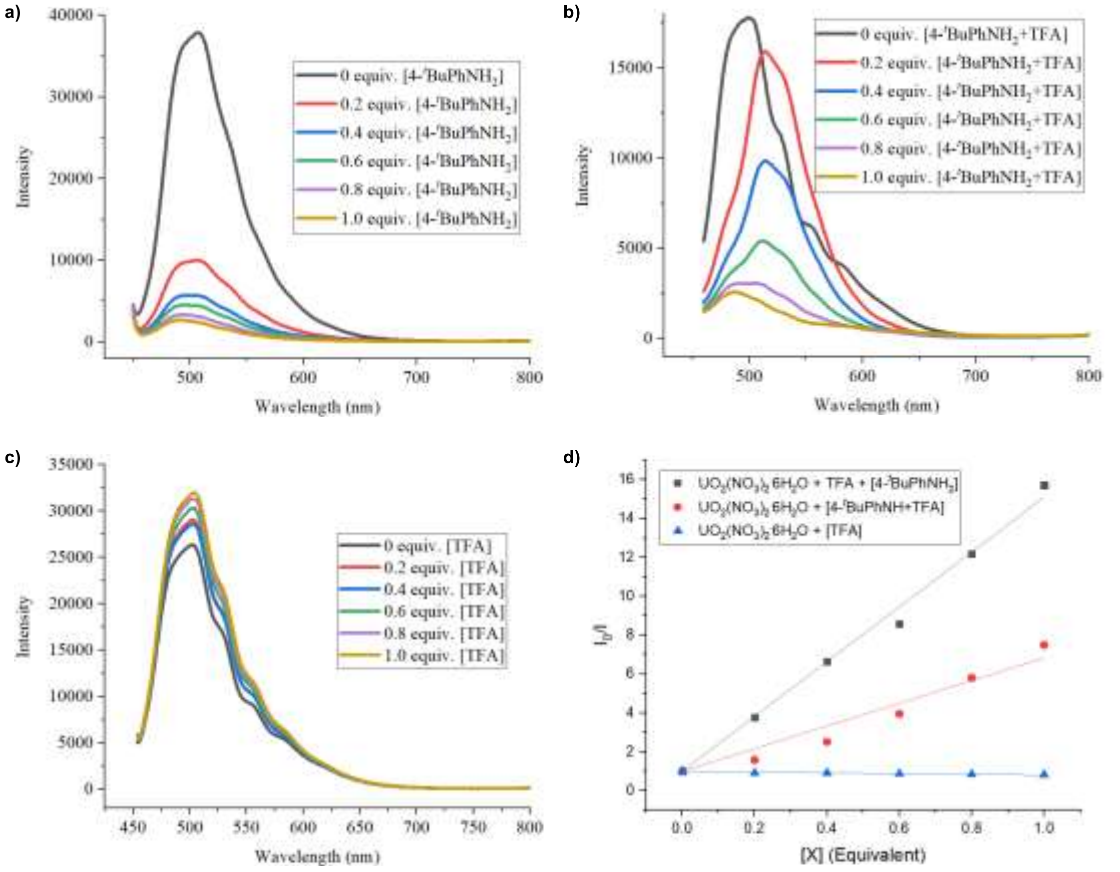

Figure S2: a) Fluorescence quenching of $UO_2(NO_3)_2{\cdot}6H_2O$ and TFA (25 equiv.) with varied [4-^{t}BuPhNH$_2$] in CH_3CN. **b)** Fluorescence quenching of $UO_2(NO_3)_2{\cdot}6H_2O$ with varied [4-^{t}BuPhNH$_2$ + TFA (n_1:n_2 = 1:1)] in CH_3CN. **c)** Fluorescence quenching of $UO_2(NO_3)_2{\cdot}6H_2O$ with varied [TFA] in CH_3CN. **d)** Stern-Volmer plots of fluorescence quenching experiments **a)**, **b)**, **c)**.

The Stern-Volmer analysis revealed that the excited state of $UO_2(NO_3)_2{\cdot}6H_2O$ photoredox catalysis was efficiently quenched by the mixture of 4-^{t}BuPhNH$_2$ and TFA in CH_3CN at room temperature.

Ultraviolet-Visible Absorption Experiments and Fluorescence Quenching Experiments with Ir[dF(CF_3)ppy]$_2$dtbpy·PF_6, Ru(bpy)$_3$$Cl_2$·6$H_2$O and Riboflavin tetraacetate

Ultraviolet-visible absorption experiments were performed using a Shimadzu UV-2700 UV-visible spectrophotometer. In each experiment, the varying samples were combined in CH_3CN in screw-top 1.0 cm quartz cuvettes. The concentration of each component under standard conditions. Fluorescence quenching studies were performed using a Shimadzu RF-6000 Fluorescence Spectrophotometer. In each experiment, the photoredox catalyst and varying concentrations of quencher were combined in CH_3CN in screw-top 1.0 cm quartz cuvettes. For the emission quenching of Ir[dF(CF_3)ppy]$_2$dtbpy·PF_6, Ru(bpy)$_3$$Cl_2$·6$H_2$O or Riboflavin tetraacetate, the photoredox catalyst concentration was $2*10^{-4}$ M, the solution was irradiated at 447 nm, 451 nm or 440 nm, respectively.

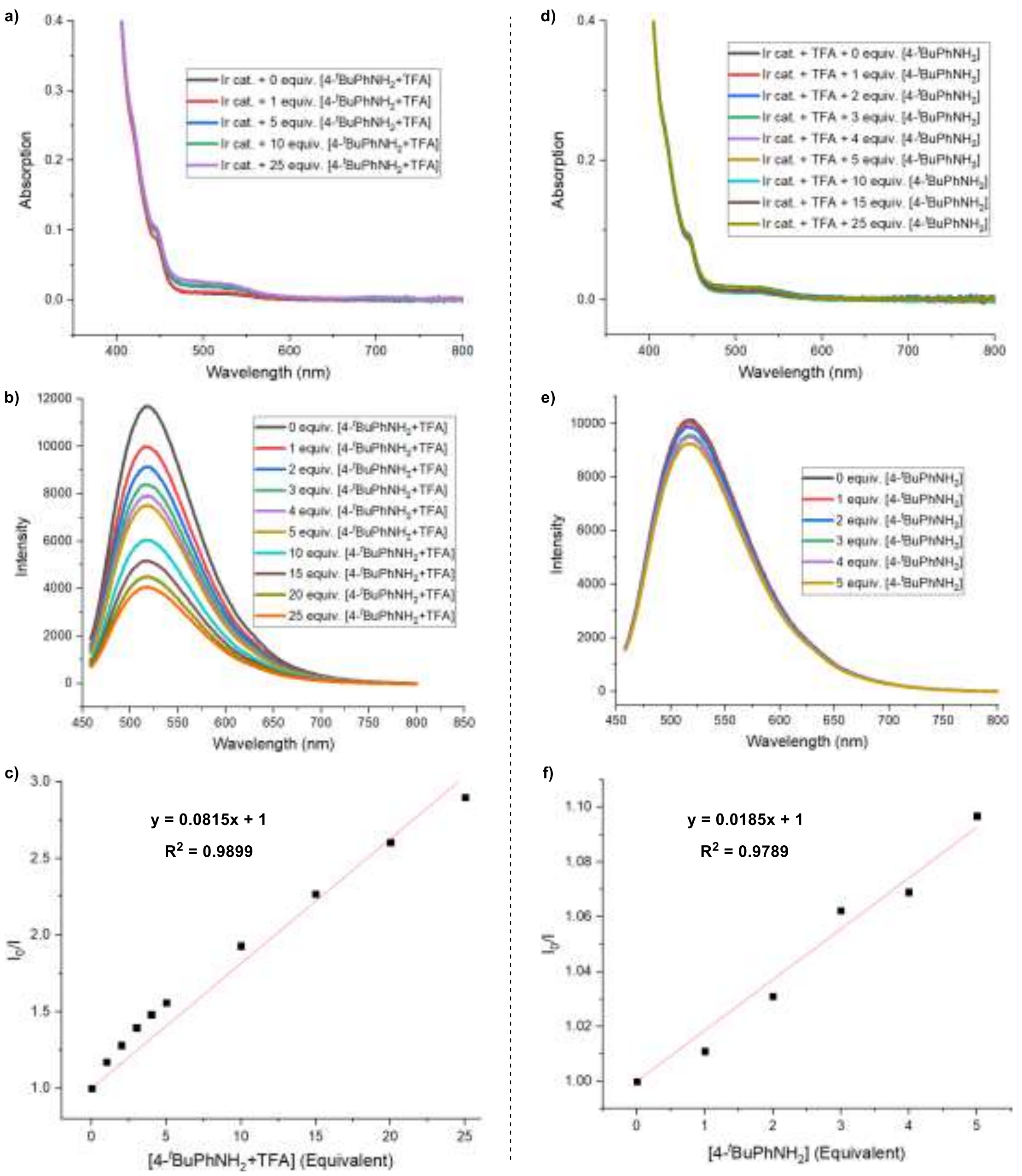

Figure S3. Ultraviolet-Visible Absorption, Fluorescence Quenching Experiments and Stern–Volmer Analysis for Ir[dF(CF_3)ppy]$_2$dtbpy·PF_6 in CH_3CN.

The Stern-Volmer analysis revealed that the excited state of Ir[dF(CF_3)ppy]$_2$dtbpy·PF_6, or the mixture of TFA and the excited state of Ir[dF(CF_3)ppy]$_2$dtbpy·PF_6 was slight quenched by the mixture of 4-^{t}BuPhNH$_2$ and TFA, or 4-^{t}BuPhNH$_2$ in CH_3CN at room temperature, respectively.

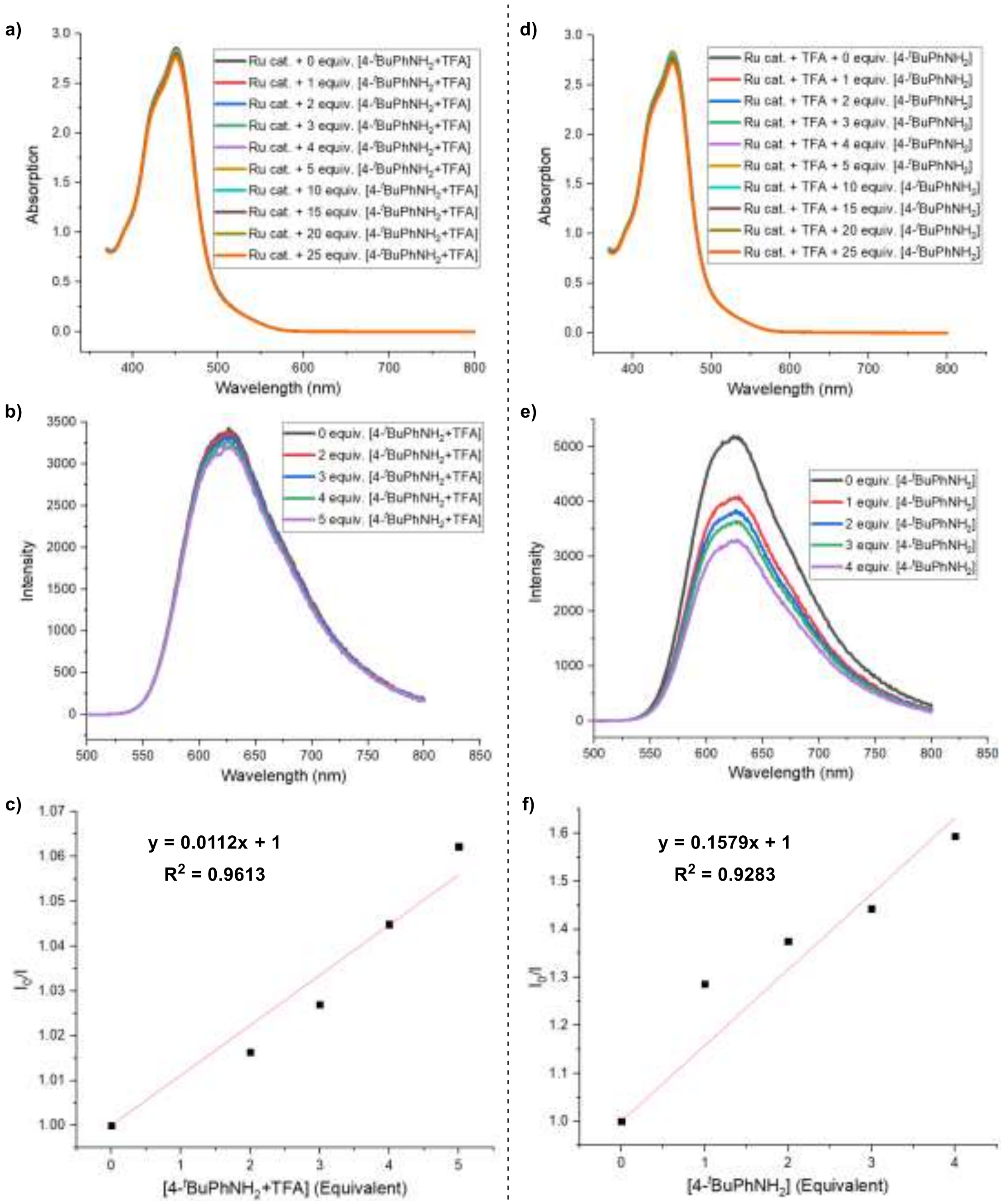

Figure S4. Ultraviolet-Visible Absorption, Fluorescence Quenching Experiments and Stern–Volmer Analysis for $Ru(bpy)_3Cl_2{\cdot}6H_2O$ in CH_3CN.

The Stern-Volmer analysis revealed that the excited state of $Ru(bpy)_3Cl_2{\cdot}6H_2O$, or the mixture of TFA and the excited state of $Ru(bpy)_3Cl_2{\cdot}6H_2O$ was slight quenched by the mixture of 4-^{t}BuPhNH$_2$ and TFA, or 4-^{t}BuPhNH$_2$ in CH_3CN at room temperature, respectively.

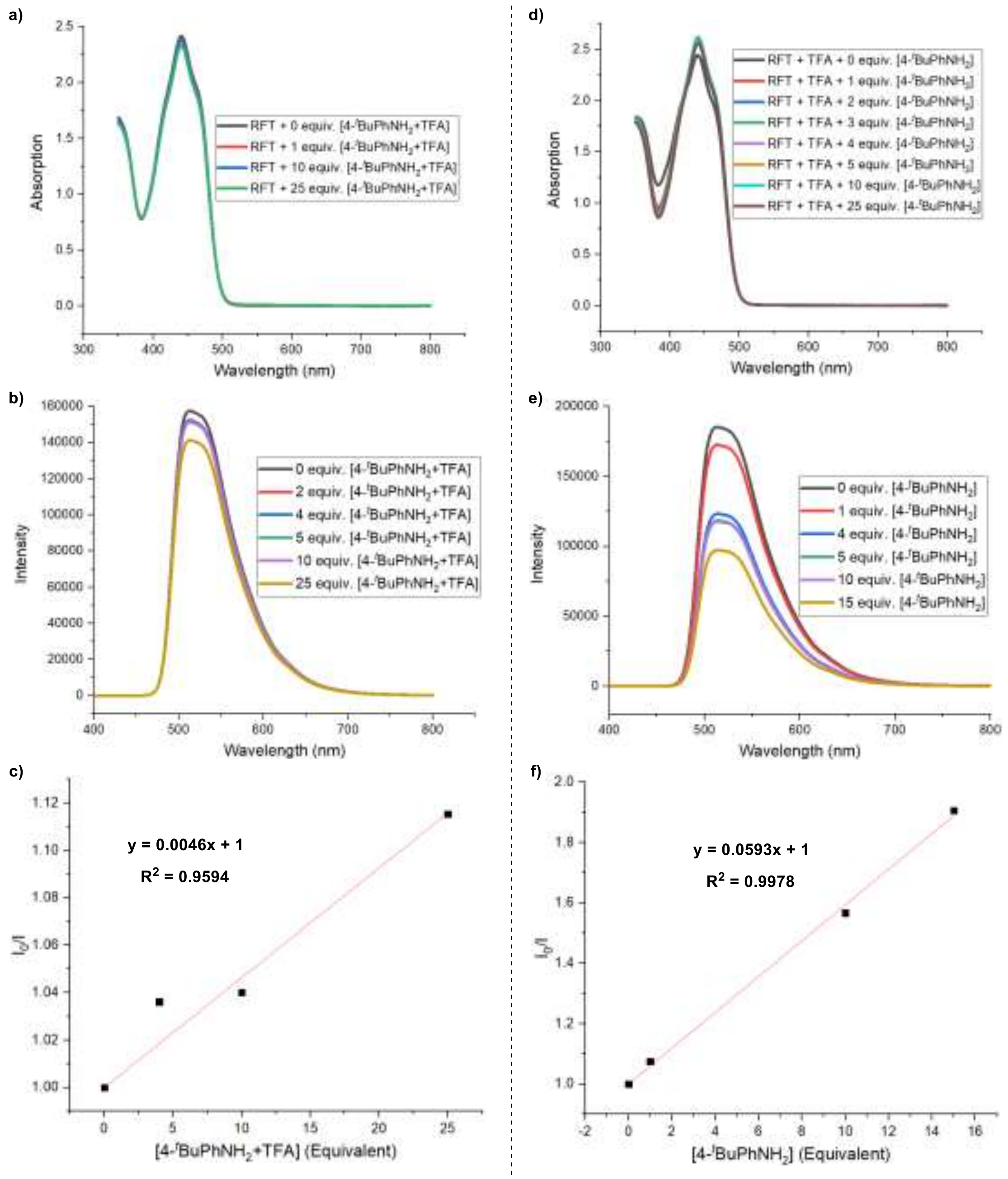

Figure S5. Ultraviolet-Visible Absorption, Fluorescence Quenching Experiments and Stern–Volmer Analysis for Riboflavin tetraacetate in CH_3CN.

The Stern-Volmer analysis revealed that the excited state of Riboflavin tetraacetate, or the mixture of TFA and the excited state of Riboflavin tetraacetate was slight quenched by the mixture of 4-^{t}BuPhNH$_2$ and TFA, or 4-^{t}BuPhNH$_2$ in CH_3CN at room temperature, respectively.

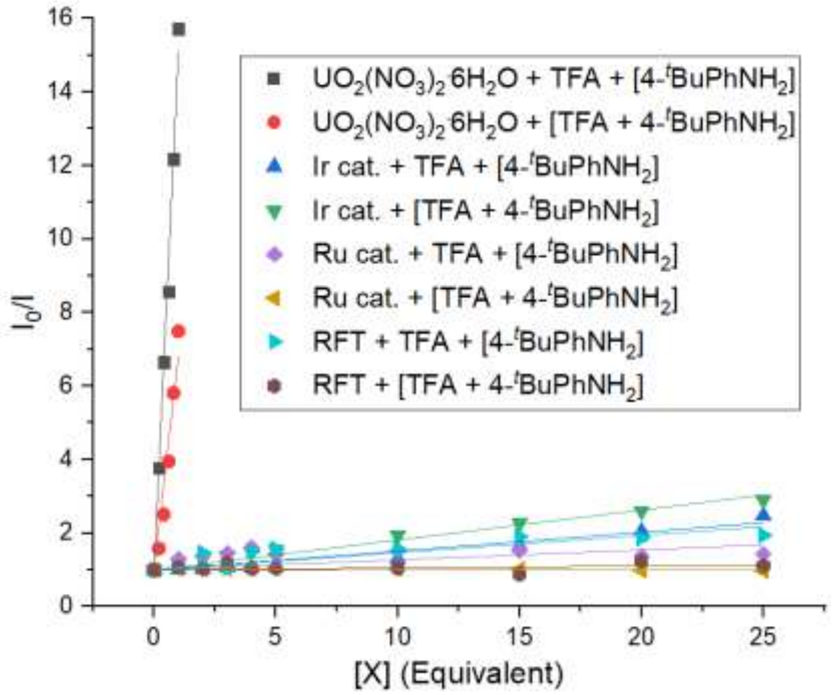

Figure S6. Stern–Volmer Analysis for $UO_2(NO_3)_2$·$6H_2O$, Ir[dF(CF_3)ppy]$_2$dtbpy·PF_6, Ru(bpy)$_3Cl_2$·$6H_2O$, Riboflavin tetraacetate, respectively.

The result of Stern–Volmer analysis indicated that the quenching efficiency of protonated 4-^{t}BuPhNH$_2$ for $UO_2(NO_3)_2$·$6H_2O$ was much stronger than Ir[dF(CF_3)ppy]$_2$dtbpy·PF_6, Ru(bpy)$_3Cl_2$·6PF_6 and Riboflavin tetraacetate in the corresponding system.

4) ^{1}H NMR Experiments

^{1}H spectra with CD_3CN was collected on 400 MHz NMR spectrometers (Bruker AVANCE).

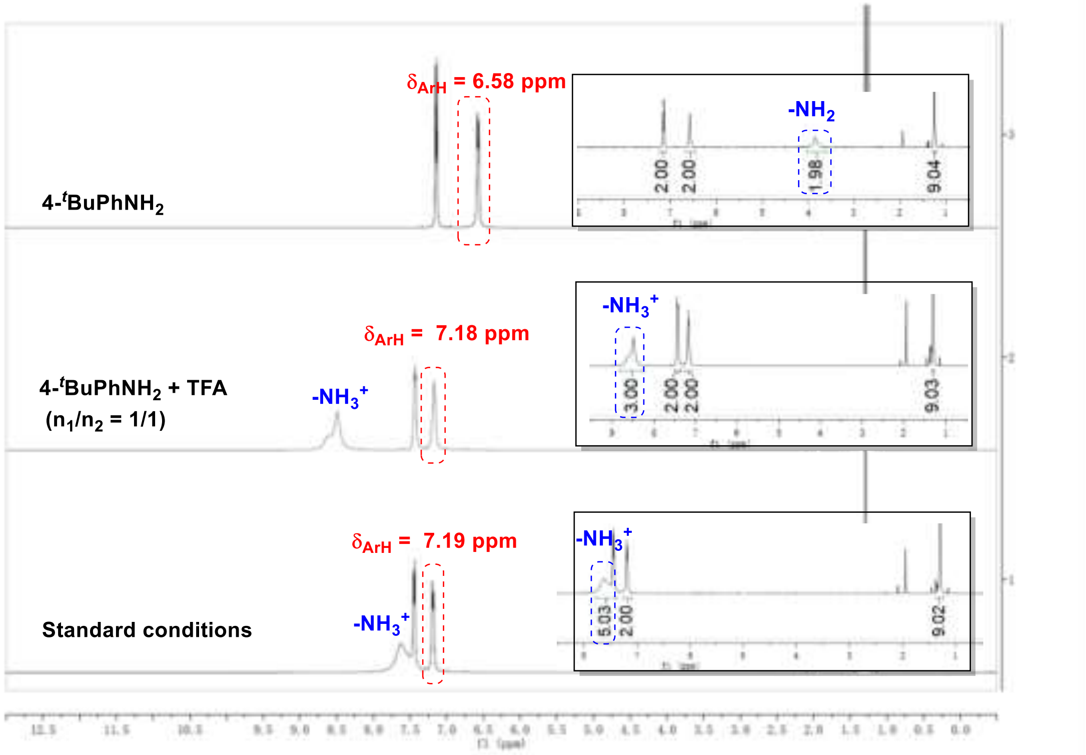

Figure S7. ^{1}H NMR Experiments with CD_3CN

^{1}H NMR experiments demonstrated that the amino group of anilines was fully protonated by TFA before C-N bond activation.

5) Oxygen Labelling Reactions

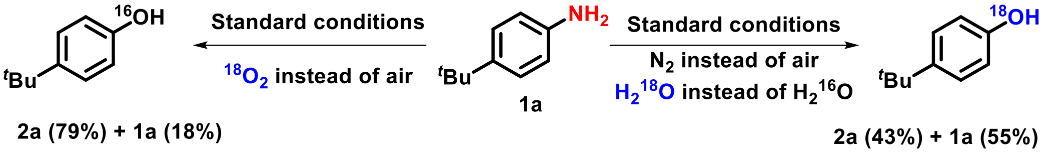

To a 25 mL Schlenk tube, 4-(*tert*-butyl)aniline **1a** (0.2 mmol, 29.9 mg), $UO_2(NO_3)_2{\cdot}6H_2O$ (4 mol%/0.008 mmol, 4 mg), TFA (0.2 mmol, 22.8 mg) and H_2O (0.6 mmol, 10.8 mg) were stirred in CH_3CN (2 mL) at room temperature for 24 h under blue light (460 nm) in a parallel reactor on $^{18}O_2$ atmosphere. The result of mass spectrometry (MS) indicated that there has no ^{18}O-labeled product **2a**.

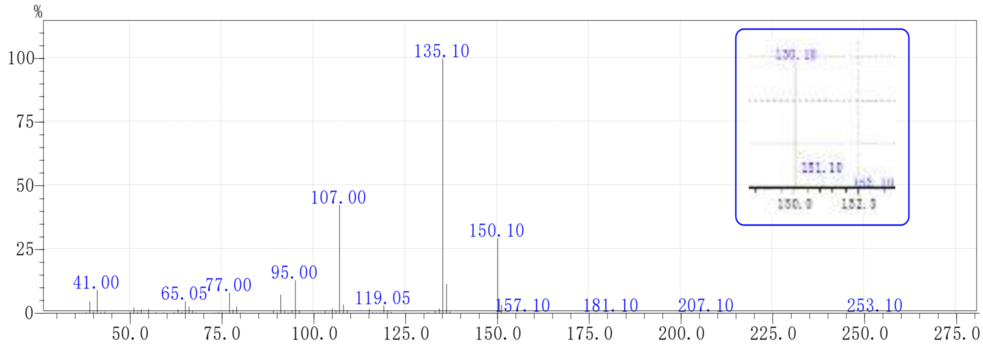

To a 50 mL Schlenk tube, 4-(*tert*-butyl)aniline **1a** (2 mmol, 299 mg), $UO_2(NO_3)_2{\cdot}6H_2O$ (4 mol%/0.008 mmol, 40.2 mg), TFA (2 mmol, 228 mg) and $H_2{}^{18}O$ (0.4 mmol, 80 mg) were stirred in CH_3CN (10 mL) at room temperature for 2.5 days under blue light in a parallel reactor under N_2 atmosphere. The result of mass spectrometry (MS) indicated that most of the product **2a** was labeled with $H_2{}^{18}O$.

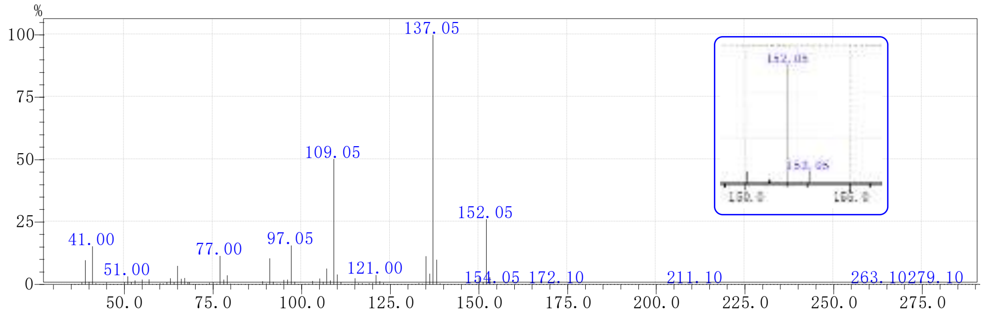

Labeling experiments with $H_2{}^{18}O$ and $^{18}O_2$ unambiguously confirmed that the oxygen atoms on the phenols originated from water instead of oxygen atmosphere.

6) ^{15}N NMR Experiments

To a 25 mL Schlenk tube, 4-(*tert*-butyl)aniline **1a** (1 mmol, 149.3 mg), $UO_2(NO_3)_2{\cdot}6H_2O$ (4 mol%/0.008 mmol, 20.1 mg), TFA (1 mmol, 114 mg) and H_2O (3 mmol, 54 mg) were stirred in HFIP (6 mL) at room temperature under blue light (460 nm) in a parallel reactor in the air.

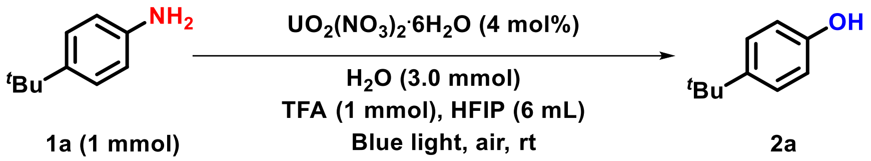

Entry	Time/h	Yields of 2a (%)
1	8	21
2	24	51
3	48	88

Yields by ^{1}H NMR with CH_2Br_2 as the internal standard.

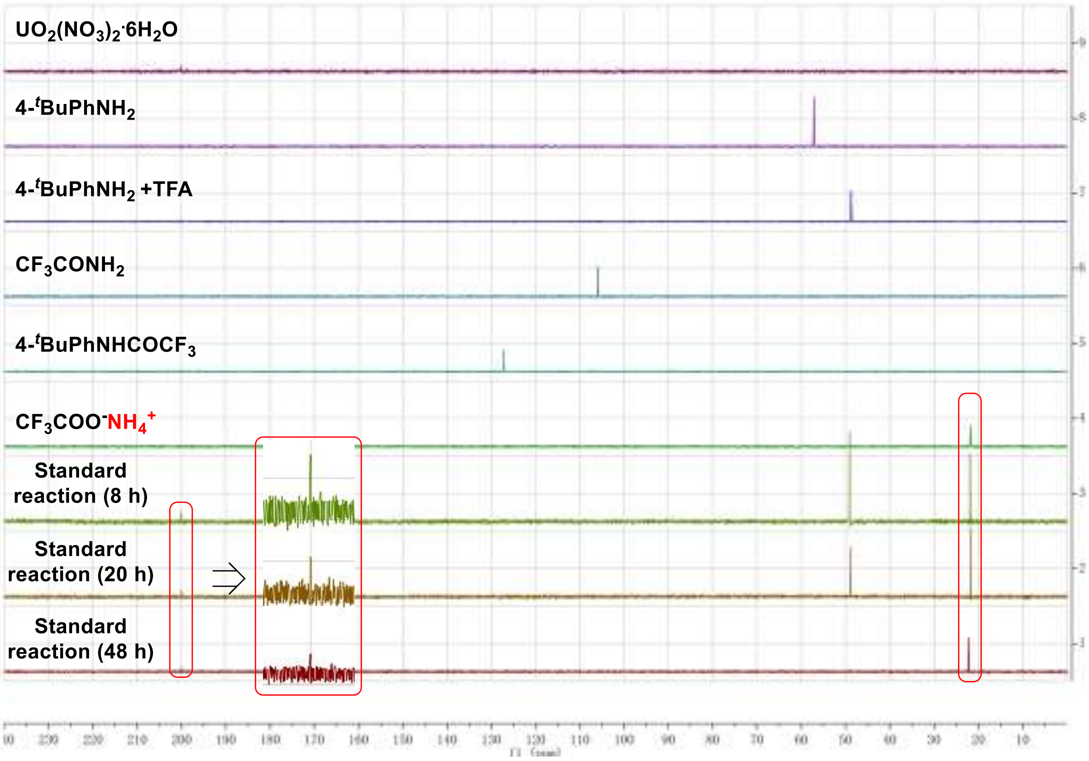

Figure S8: ^{15}N NMR Experiments with Various Nitrogenous Substances.

The result of ^{15}N NMR experiments elucidated that the nitrogen atoms on the anilines leaving in the form of ammonium trifluoroacetate (CF_3COONH_4). In addition, there had no other nitrogen-species detected by tracking experiments with ^{15}N NMR experiments.

7) On-off Experiments

All the reactions were conducted under standard conditions and stirred for certain hours with light and without light. The corresponding yields were calculated by NMR with CH_2Br_2 as internal standard.

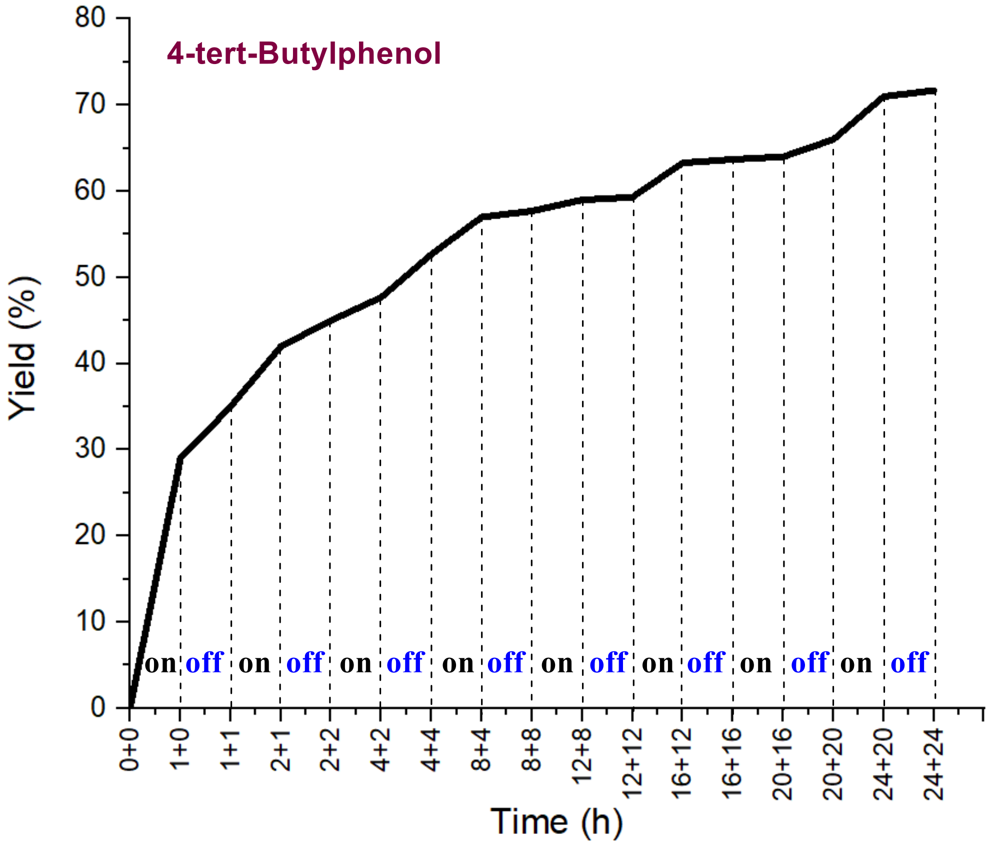

Figure S9: On-Off Experiments of 4-^{t}BuPhNH$_2$ Procedure.

The results shown that the C-N bond hydroxylation of anilines proceeded even during the period without light irradiation.

8) Quantum Yield Measurements[4-5]

A. Photon Flux Detection

A ferrioxalate actinometer solution was prepared by following the Hammond variation of the Hatchard and Parker procedure outlined in *Handbook of Photochemistry.*[6] The ferrioxalate actinometer solution measures the decomposition of ferric ions to ferrous ions, which are complexed by 1,10-phenanthroline and monitored by UV/Vis absorbance at 510 nm. The moles of iron-phenanthroline complex formed are related to moles of photons absorbed.

The solutions were prepared and stored in a dark laboratory:

1. Potassium ferrioxalate solution: 589.5 mg of potassium ferrioxalate (commercially available from Alfa Aesar) and 278 µL of sulfuric acid (96%) were added to a 100 mL volumetric flask, and filled to the mark with water (HPLC grade).

2. Phenantroline solution: 0.2% by weight of 1,10-phenanthroline in water (200 mg in 100 mL volumetric flask).

3. Buffer solution: to a 100 mL volumetric flask, 4.94 g of NaOAc and 1 mL of sulfuric acid (96%) were added and filled to the mark with water (HPLC grade).

Procedure: 1 mL of the actinometer solution, whose quantum yield is being measured, were added to a standard 25 mL Schlenk tube bought from Synthware. The tube was placed in parallel photoreactor irradiated without stirring by blue light (460 nm) who worked at 0.1 A and 0.28 V. This procedure was repeated 2 times, quenching the reactions after different time intervals: 5, 10, 15, 20, and 25 seconds.

The actinometer measurements were done as follows: 1) After irradiation, the actinometer solution was removed and placed in a 10 mL volumetric flask containing 0.5 mL of 1,10-phenanthroline solution and 2 mL of buffer solution. This flask was filled to the mark with water (HPLC grade). 2) The UV-vis spectra of the complexed actinometer samples were recorded for each time interval. The absorbance of the complexed actinometer solution was monitored at 510 nm. The moles of Fe^{2+} formed for each sample are determined according to the Beer's Law (eq. S1):

$$moles\ (Fe^{2+}) = \frac{V_1 * V_3 * \Delta A\ (510\ nm)}{10^3 * V_2 * l * \varepsilon\ (510\ nm)} \quad \text{(eq S1)}$$

where V_1 is the irradiated volume (1 mL), V_2 is the aliquot of the irradiated solution taken for the determination of the ferrous ions (1 mL), V_3 is the final volume after complexation with phenanthroline (10 mL), *l* is the optical path-length of the irradiation cell (1 cm), ΔA (510 nm) the optical difference in absorbance between the irradiated solution and the one stored in the dark, ε (510 nm) is that of the complex $Fe(phen)_3^{2+}$ (11100 $L{\cdot}mol^{-1}{\cdot}cm^{-1}$). The moles of Fe^{2+} formed (x) are plotted as a function of time (t). The slope of this line was correlated to the moles of incident photons by unit of time (q^0n,p) by the use of the following Equation S2:

$$\Phi\ (\lambda) = \frac{dx/dt}{q^{0}_{n,p}\ [1\text{-}10^{-A(\lambda)}]} \qquad \text{(eq S2)}$$

where dx/dt is the rate of change of a measurable quantity (spectral or any other property), the quantum yield (Φ) for Fe^{2+} at 460 nm is 0.9, and A (λ) is the absorbance of the actinometer at the wavelength used to carry out the experiments (460 nm). The absorbance at 460 nm A(460) was measure using a Shimadzu UV-2700 UV-Vis spectrophotometer in 1 mm path quartz cuvettes in the presence of the bandpass filter of 450 nm employed to run the measurements, obtaining an absorbance of 0.14.

q^0n,p, which is the photon flux, was determined to be $2.4{*}10^{-10}$ einstein s^{-1}.

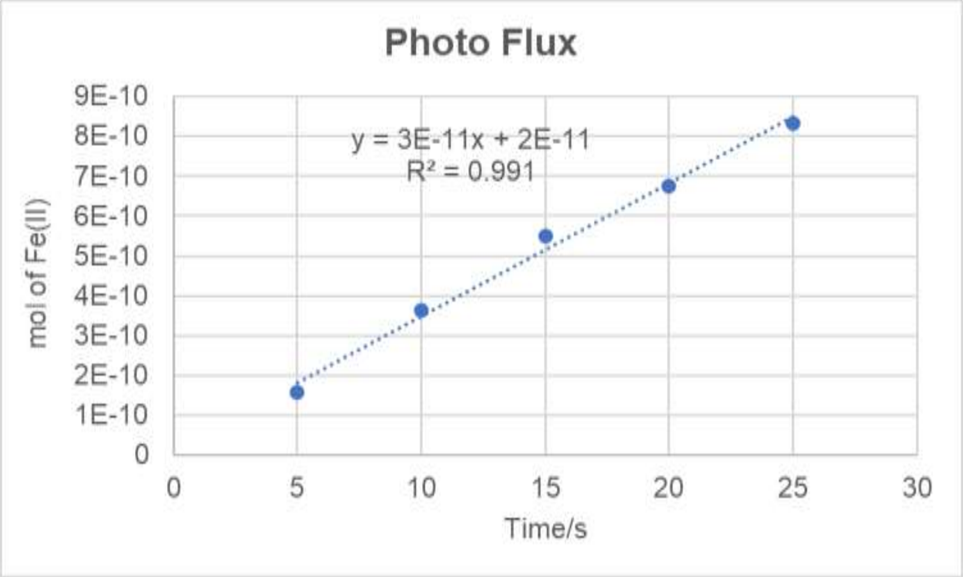

Figure S10: Photon Flux Detection Experiments.

B. Quantum Yields Calculation

a) Quantum Yield of Standard Conditions as eq S3

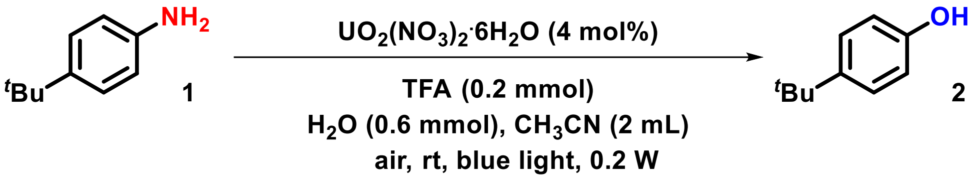

(eq S3)

To a 50 mL Schlenk tube, aniline **1a** (0.2 mmol, 29.9 mg), $UO_2(NO_3)_2{\cdot}6H_2O$ (4 mol%/0.008 mmol, 4 mg), TFA (0.2 mmol, 24.8 mg) and H_2O (0.6 mmol, 10.8 mg) were stirred in CH_3CN (2 mL) at room temperature in a photoreactor in the air, which worked at 0.2 W. Reactions were separately stopped at 10, 30, 40, 70, 100 minutes. The moles of product **2a** formed were determined by LC measurement using external standard method.

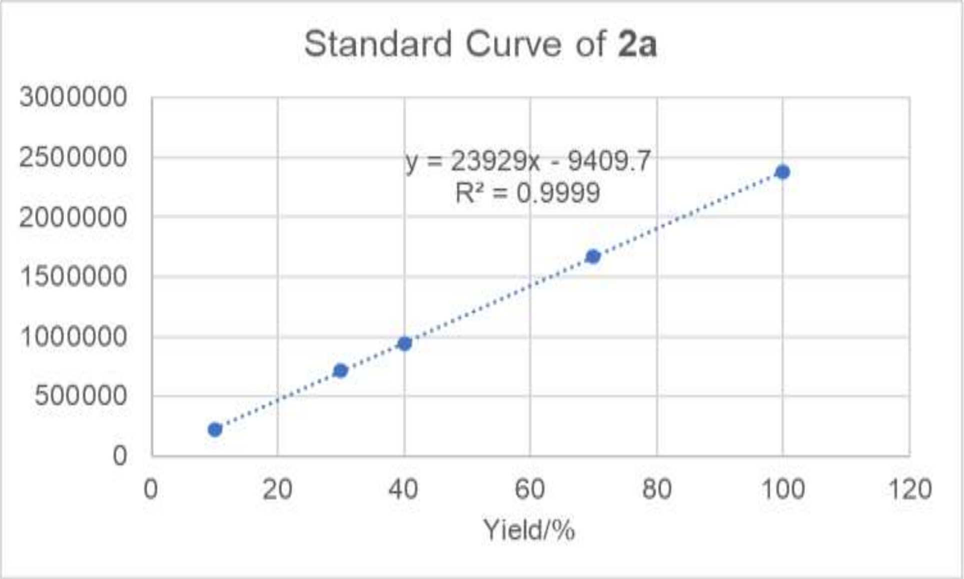

Figure S11: Standard Curve of phenol 2a on LC.

The moles of product per unit of time are related to the number of photons absorbed. The photons absorbed are correlated to the number of incident photons by the use of the equation displayed in the previous point. According to Equation S2, if we plot the moles of product (x) versus the moles of incident photons (q^0n,p*dt), the slope is equal to: **Φ·(1-10-A(460 nm))**, where Φ is the quantum yield to be determined and *A*(460 nm) is the absorption of the reaction under study. *A*(460 nm) was measured using a Shimadzu UV-2700 UV-Vis spectrophotometer in 1 mm path

quartz in the presence of the bandpass filter of 460 nm employed to carry out the measurements. An absorbance of 0.032 was determined for the reaction with **2a**. The quantum yield (Φ) of the photochemical reaction S3 was calculated to be **8.4**.

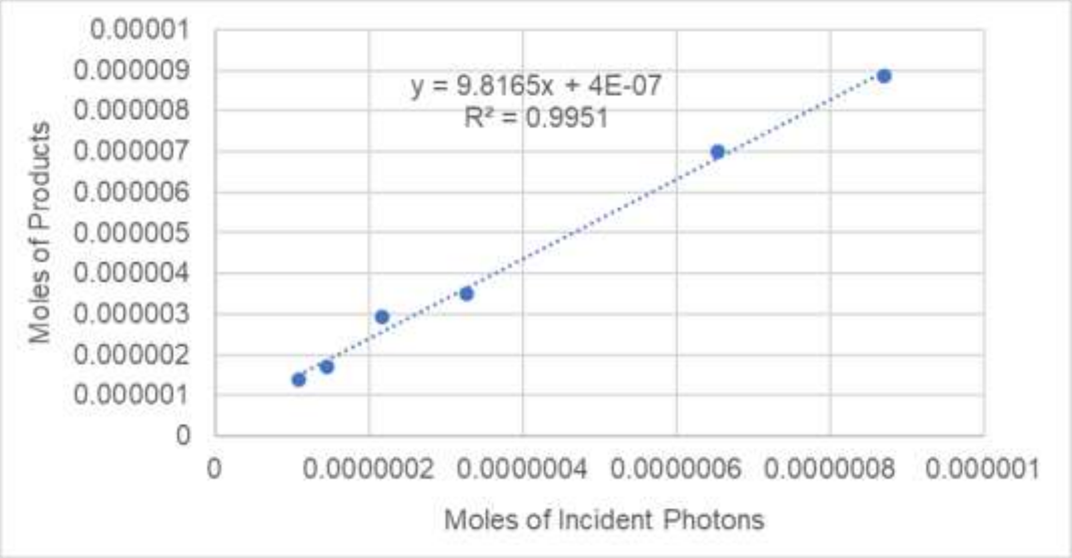

Figure S12: Quantum Yield Calculation of Phenol 2a under Standard Conditions.

Quantum Yield Calculation Experiments Photoreactor:

The photoreactor used in this research from Shanghai 3S Technology Co. Ltd.

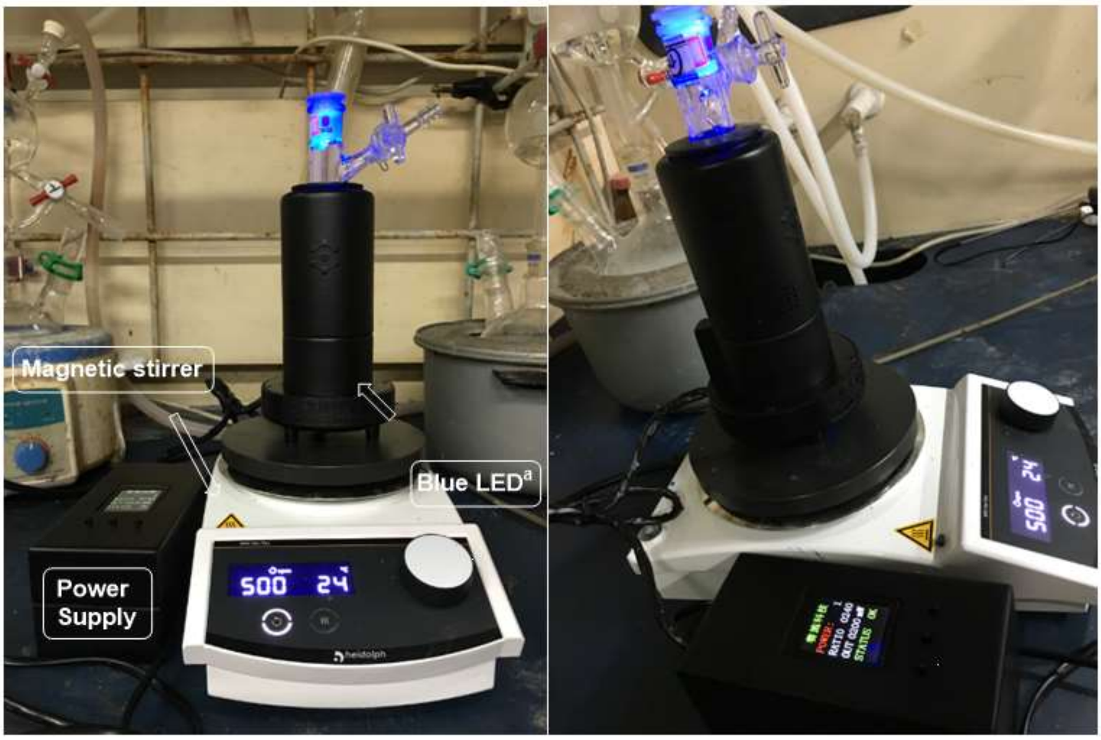

Figure S13: Quantum Yields Experiments Photoreactor

9) Cyclic voltammetry studies

Cyclic voltammograms were collected with a CHI600E electrochemistry station from CH Instruments Ins. Samples were prepared with 0.004 mmol of substrate [anilines, or anilines and trifluoroacetic acid (TFA)] in 5 mL of 0.1 M tetrabutylammonium perchlorate (TBAP) in solvent [acetonitrile (CH_3CN), 1,1,1,3,3,3-hexafluoro-2-propanol (HFIP), or nitromethane (CH_3NO_2)]. Measurements employed a glassy carbon working electrode, platinum wire counter electrode, saturated calomel electrode as reference electrode, and a scan rate of 100 mV/s. Reductions were measured by scanning potentials in the negative direction and oxidations in the positive direction; the glassy carbon electrode was polished between each scan.

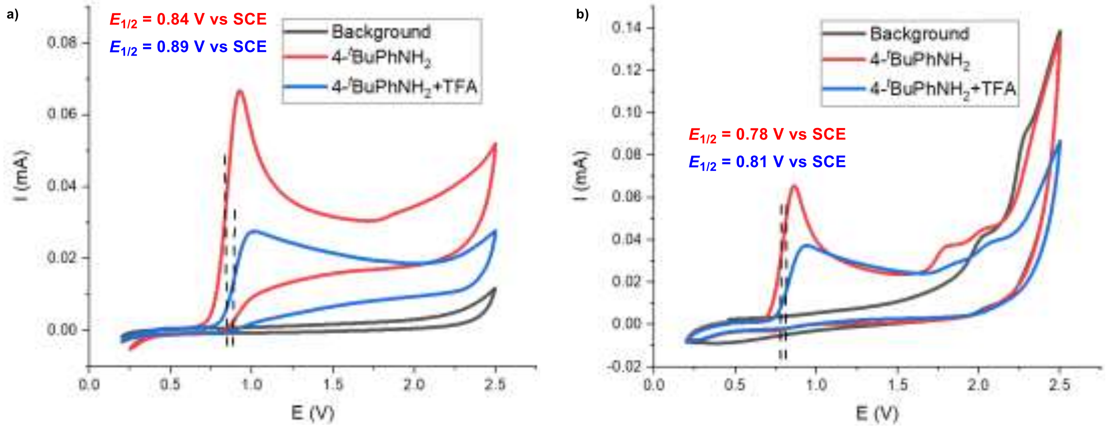

Figure S14: Cyclic Voltammogram of 4-^{t}BuPhNH$_2$ in a), CH_3CN and b), CH_3NO_2.

The oxidation potential of protonated aniline is larger than the free aniline, which was similar with previous work.[7] The oxidation potential of 4-^{t}BuPhNH$_2$ is lower in CH_3NO_2 than CH_3CN.

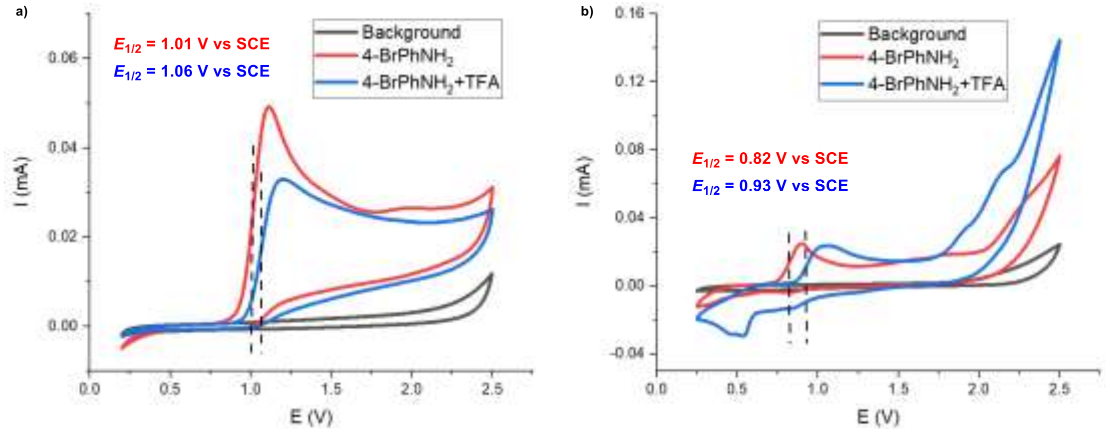

Figure S15: Cyclic Voltammogram of 4-BrPhNH$_2$ in a), CH$_3$CN and b), HFIP.

The oxidation potential of 4-BrPhNH$_2$ is lower in HFIP than CH$_3$CN.

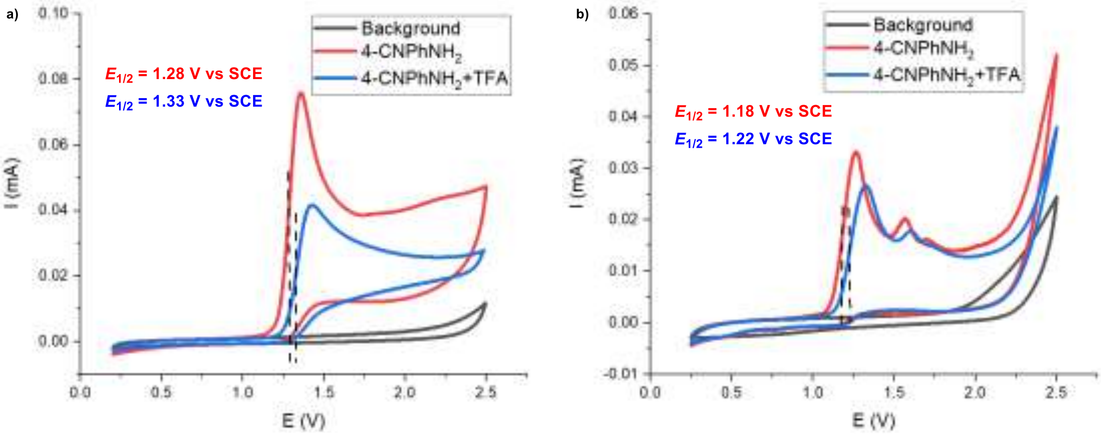

Figure S16: Cyclic Voltammogram of 4-CNPhNH$_2$ in a), CH$_3$CN and b), HFIP.

The oxidation potential of 4-CNPhNH$_2$ is lower in HFIP than CH$_3$CN.

V. Procedures and Datas for Starting Materials in Figure 2.

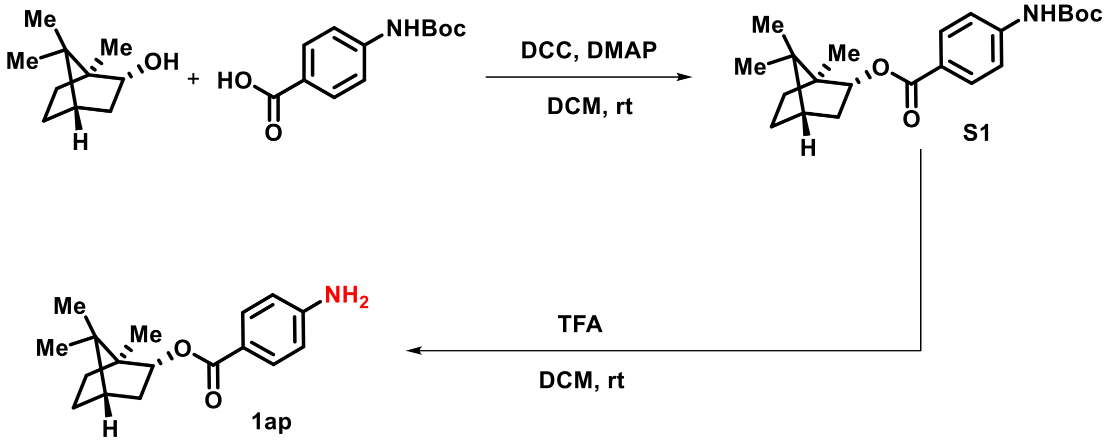

(1*S*,2*R*,4*S*)-1,7,7-trimethylbicyclo[2.2.1]heptan-2-yl 4-((*tert*-butoxycarbonyl)amino)benzoate S1: A solution of L(-)-Borneol (5 mmol, 772 mg), 4-((*tert*-butoxycarbonyl)amino)benzoic acid (5 mmol, 1.19 g), *N*,*N*-dimethylpyridin-4-amine (DMAP, 1 mmol, 123 mg) and DCM (20 mL) were stirred at room temperature. Dicyclohexylmethanediimine (DCC, 10 mmol, 2.06 g) was added in one porton manner. After the reaction by TLC, removing dichloromethane (DCM) by rotary evaporation and dissolving the residue with EA (30 mL). Then filtered, the filtrate was extracted with citric acid (10 wt%), saturated sodium chloride respectively. Dry the combined organic phase with anhydrous $MgSO_4$ and then evaporate under reduced pressure. The residue was chromatographed on silica gel using petroleum ether/ethyl acetate (V_{PE}/V_{EA} = 40/1 to 20/1) as eluent to give **S1** (1.34 g, 72%) with offwhite solid.

(1*S*,2*R*,4*S*)-1,7,7-trimethylbicyclo[2.2.1]heptan-2-yl 4-aminobenzoate 1ap: A mixture of **S1** (2.5 mmol, 933 mg), trifluoroacetic acid (TFA, 0.75 mmol, 86 mg) was stirred in DCM (8 mL) at room temperature. After the reaction by TLC, dichloromethane (DCM) was removed by rotary evaporation. The residue was chromatographed on silica gel using petroleum ether/ethyl acetate (V_{PE}/V_{EA} = 10/1) as eluent to give **1ap** (588 mg, 86%) with white solid. **^{1}H NMR** (400 MHz, DMSO-*d6*) δ 7.66 (d, *J* = 8.0 Hz, 2H), 6.61 (d, *J* = 8.0 Hz, 2H), 6.11 (brs, 2H), 4.93 (d, *J* = 8.0 Hz, 1H), 2.37-2.29 (m, 1H), 2.08-2.02 (m, 1H), 1.78-1.68 (m, 2H), 1.38-1.32 (m, 1H), 1.28-1.22 (m, 1H), 0.99 (dd, J_1 = 8.0 Hz, J_2 = 4.0 Hz, 1H), 0.91 (s, 3H), 0.87 (s, 3H),

0.83 (s, 3H). **^{13}C NMR** (100 MHz, DMSO-*d6*) δ 165.9, 152.8, 131.0, 116.9, 113.2, 78.4, 48.6, 47.5, 44.4, 36.6, 27.7, 27.0, 19.6, 18.7, 13.6. **IR** (neat) 2912, 1715, 1664, 1613, 1275, 1148, 1117 cm^{-1}. **MS** (ESI) Calculated for $C_{17}H_{24}NO_2$ $(M+H)^+$: 274.2; Found 274.3.

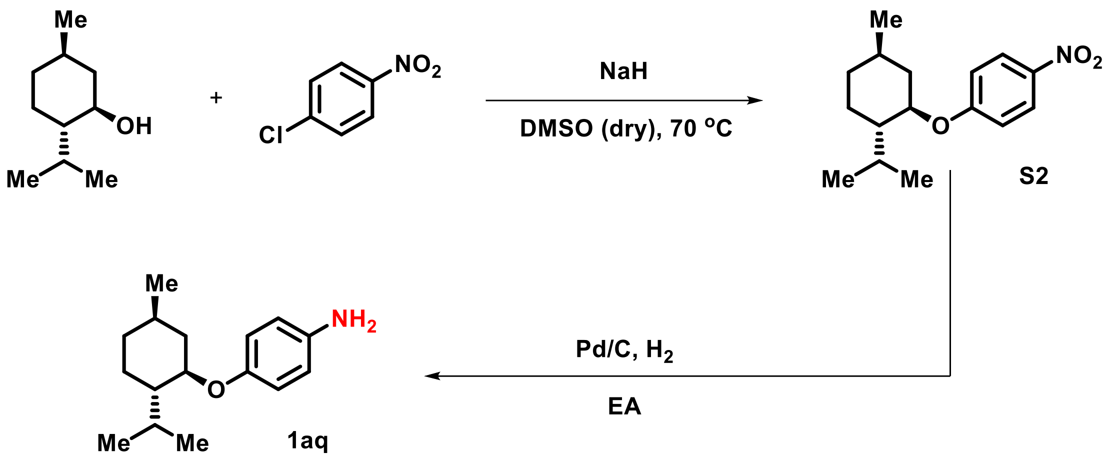

1-(((*1R,2S,5R*)-2-isopropyl-5-methylcyclohexyl)oxy)-4-nitrobenzene S2 was synthesized according to previous method.[8]

4-(((*1R,2S,5R*)-2-isopropyl-5-methylcyclohexyl)oxy)aniline 1aq: A mixture of **S2** (7 mmol, 2.38 g), Pd/C (10 wt%, 238 mg) was stirred in EA (5 mL) under H_2 atmosphere (> 4 atm) at 50 °C about 5 hours. The mixture was filtered and the filtrate evaporated to dryness. The residue was chromatographed on silica gel using petroleum ether/ethyl acetate (V_{PE}/V_{EA} = 10/1) as eluent to give **1aq** (1.42 g, 82%) with white solid. **^{1}H NMR** (400 MHz, DMSO-*d6*) δ 6.63 (d, *J* = 8.0 Hz, 2H), 6.49 (d, *J* = 8.0 Hz, 2H), 4.69 (brs, 2H), 3.81-3.75 (m, 1H), 2.22-2.15 (m, 1H), 2.02-1.99 (m, 1H), 1.66-1.59 (m, 2H), 1.43-1.31 (m, 2H), 1.09-0.98 (m, 1H), 0.89-0.80 (m, 8H), 0.76 (d, *J* = 4.0 Hz, 3H). **^{13}C NMR** (100 MHz, DMSO-*d6*) δ 149.0, 142.4, 117.4, 115.1, 77.8, 47.8, 40.4, 34.1, 30.7, 25.5, 23.1, 22.1, 20.7, 16.4. **IR** (neat) 2958, 2931, 1621, 1507, 1458, 1218, 820 cm^{-1}. **MS** (ESI) Calculated for $C_{16}H_{26}NO$ $(M+H)^+$: 248.2; Found 248.1.

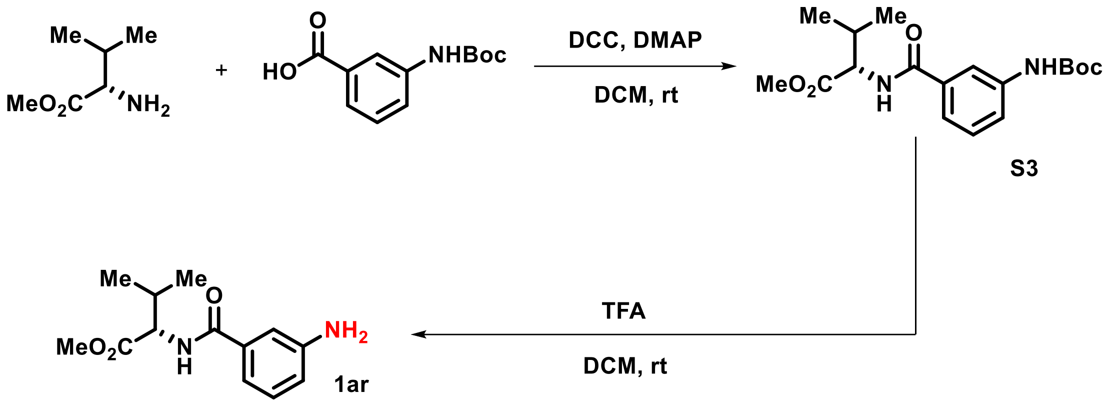

Methyl (3-((*tert*-butoxycarbonyl)amino)benzoyl)-L-valinate S3: A solution of L-Valine (5 mmol, 586 mg), 3-((tert-butoxycarbonyl)amino)benzoic acid (5 mmol, 1.19 g), *N*,*N*-dimethylpyridin-4-amine (DMAP, 1 mmol, 123 mg) and DCM (20 mL) were stirred at 0 °C. Dicyclohexylmethanediimine (DCC, 10 mmol, 2.06 g) was added in one portion manner. Subsequently, the temperature from 0 °C to rt. After the reaction by TLC, removing dichloromethane (DCM) by rotary evaporation and dissolving the residue with EA (30 mL). Then filtered, the filtrate was extracted with citric acid (10 wt%), saturated sodium chloride respectively. Dry the combined organic phase with anhydrous $MgSO_4$ and then evaporate under reduced pressure. The residue was chromatographed on silica gel using petroleum ether/ethyl acetate (V_{PE}/V_{EA} = 5/1) as eluent to give **S3** (1.32 g, 75%) with white solid.

Methyl (3-aminobenzoyl)-*L*-valinate 1ar: A mixture of **S3** (2.5 mmol, 875 mg), trifluoroacetic acid (TFA, 0.75 mmol, 86 mg) was stirred in DCM (8 mL) at room temperature. After the reaction by TLC, dichloromethane (DCM) was removed by rotary evaporation. The residue was chromatographed on silica gel using petroleum ether/ethyl acetate (V_{PE}/V_{EA} = 5/1) as eluent to give **1ar** (569 mg, 91%) with white solid. **^{1}H NMR** (400 MHz, DMSO-*d6*) δ 8.57 (d, *J* = 8.0 Hz, 1H), 7.42-7.39 (m, 2H), 7.31 (t, *J* = 8.0 Hz, 1H), 7.07 (d, *J* = 8.0 Hz, 1H), 5.97 (brs, 2H), 4.27 (t, *J* = 8.0 Hz, 1H), 3.65 (s, 3H), 2.22-2.13 (m, 1H), 0.97 (d, *J* = 8.0 Hz, 3H), 0.92 (d, *J* = 8.0 Hz, 3H). **^{13}C NMR** (100 MHz, DMSO-*d6*) δ 172.3, 167.1, 141.6, 135.2, 129.2, 120.8, 120.2, 117.3, 58.7, 51.7, 29.6, 19.2, 19.1. **IR** (neat) 2968, 2878, 1638, 1532, 1528, 1201, 1132 cm^{-1}. **MS** (ESI) Calculated for $C_{13}H_{17}N_2O_3$ $(M-H)^-$: 249.1; Found 249.1.

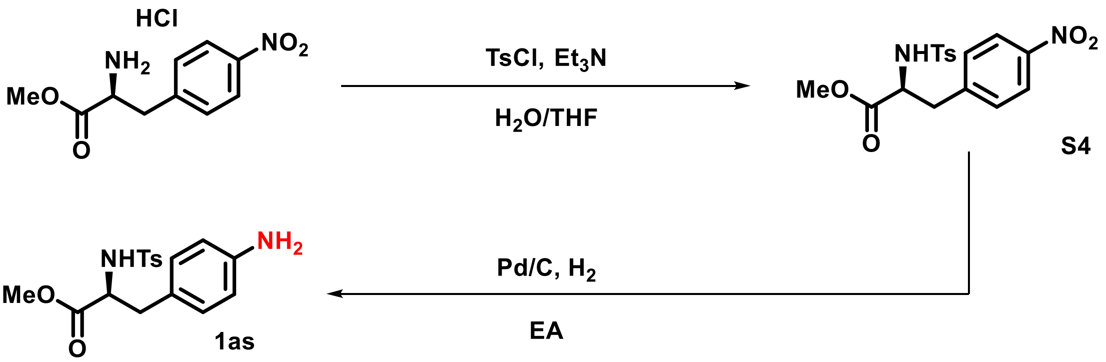

Methyl (*S*)-2-((4-methylphenyl)sulfonamido)-3-(4-nitrophenyl)propanoate S4: Tosyl chloride (TsCl, 2 mmol, 380 mg) was added to a solution of L-4-Nitrophenylalanine methyl ester hydrochloride (2 mmol, 520 mg), Et_3N (6 mmol, 608 mg) and H_2O/THF (V/V = 2/1, 15 mL) at 0 °C. Subsequently, the temperature from 0 °C to rt. After the reaction by TLC, DCM (30 mL) was added to quench it. The mixture was extracted with saturated sodium chloride. Dry the combined organic phase with anhydrous $MgSO_4$ and then evaporate under reduced pressure. The residue was chromatographed on silica gel using petroleum ether/ethyl acetate (V_{PE}/V_{EA} = 2/1) as eluent to give **S4** (650 mg, 86%) with white solid.

Methyl (*S*)-3-(4-aminophenyl)-2-((4-methylphenyl)sulfonamido)propanoate 1as: A mixture of **S4** (1 mmol, 378 mg), Pd/C (10 wt%, 38 mg) was stirred in EA (3 mL) under H_2 atmosphere (> 4 atm) at 50 °C about 5 hours. The mixture was filtered and the filtrate evaporated to dryness. The residue was chromatographed on silica gel using petroleum ether/ethyl acetate (V_{PE}/V_{EA} = 1/1) as eluent to give **1as** (324 mg, 93%) with white solid. **^{1}H NMR** (400 MHz, DMSO-*d6*) δ 8.33 (d, *J* = 8.0 Hz, 1H), 7.48 (d, *J* = 8.0 Hz, 2H), 7.29 (d, *J* = 8.0 Hz, 2H), 6.72 (d, *J* = 8.0 Hz, 2H), 6.43 (d, *J* = 8.0 Hz, 2H), 5.05 (brs, 1H), 3.80-3.74 (m, 1H), 3.30 (s, 3H), 2.72-2.66 (m, 1H), 2.59-2.54 (m, 1H), 2.36 (s, 3H). **^{13}C NMR** (100 MHz, DMSO-*d6*) δ 171.3, 147.1, 142.5, 137.8, 129.5, 129.3, 126.4, 122.9, 113.9, 57.9, 51.6, 37.2, 21.0. **IR** (neat) 3452, 3372, 1740, 1627, 1515, 1337, 1294 cm^{-1}. **MS** (ESI) Calculated for $C_{17}H_{21}N_2O_4S$ $(M+H)^+$: 349.1; Found 349.3.

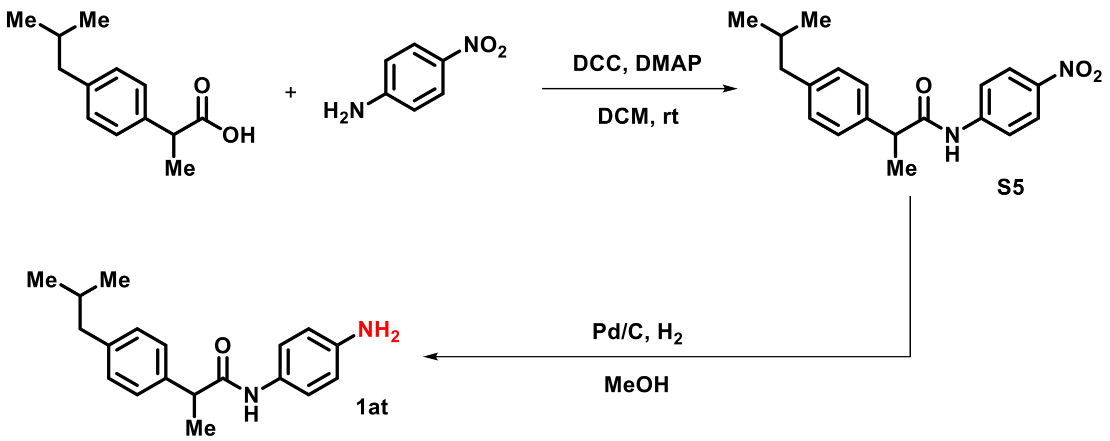

2-(4-Isobutylphenyl)-*N*-(4-nitrophenyl)propanamide S5: A solution of Ibuprofen (5 mmol, 1.03 g), 4-Nitroaniline (6 mmol, 830 mg), *N*,*N*-dimethylpyridin-4-amine (DMAP, 1 mmol, 123 mg) and DCM (20 mL) were stirred at room temperature (rt). Dicyclohexylmethanediimine (DCC, 10 mmol, 2.06 g) was added in one portion manner. After the reaction by TLC, removing dichloromethane (DCM) by rotary evaporation and dissolving the residue with EA (30 mL). Then filtered, the filtrate was extracted with citric acid (10 wt%), saturated sodium chloride respectively. Dry the combined organic phase with anhydrous $MgSO_4$ and then evaporate under reduced pressure. The residue was chromatographed on silica gel using petroleum ether/ethyl acetate (V_{PE}/V_{EA} = 5/1) as eluent to give **S5** (897 mg, 55%) with white solid.

***N*-(4-aminophenyl)-2-(4-isobutylphenyl)propanamide 1at:** A mixture of **S5** (2 mmol, 652 mg), Pd/C (10 wt%, 66 mg) was stirred in MeOH (6 mL) under H_2 atmosphere (> 4 atm) at 50 ^oC about 5 hours. The mixture was filtered and the filtrate evaporated to dryness. The residue was chromatographed on silica gel using petroleum ether/ethyl acetate (V_{PE}/V_{EA} = 2/1) as eluent to give **1at** (421 mg, 71%) with white solid. **^{1}H NMR** (400 MHz, DMSO-*d6*) δ 9.58 (brs, 1H), 7.27 (d, *J* = 8.0 Hz, 2H), 7.20 (d, *J* = 12.0 Hz, 2H), 7.09 (d, *J* = 8.0 Hz, 2H), 6.47 (d, *J* = 8.0 Hz, 2H), 4.81 (brs, 2H), 3.70 (q, *J* = 8.0 Hz, 1H), 2.39 (d, *J* = 4.0 Hz, 2H), 1.83-1.76 (m, 1H), 1.36 (d, *J* = 8.0 Hz, 3H), 0.84 (d, *J* = 4.0 Hz, 6H). **^{13}C NMR** (100 MHz, DMSO-*d6*) δ 171.3, 144.6, 139.6, 139.3, 128.8, 128.5, 126.9, 120.8, 113.7, 45.3, 44.2, 29.6, 22.2, 18.7. **IR** (neat) 2969, 1654, 1611, 1528, 1516, 1424, 1246 cm^{-1}. **MS** (ESI) Calculated

for $C_{19}H_{25}N_2O$ $(M+H)^+$: 297.2; Found 297.0.

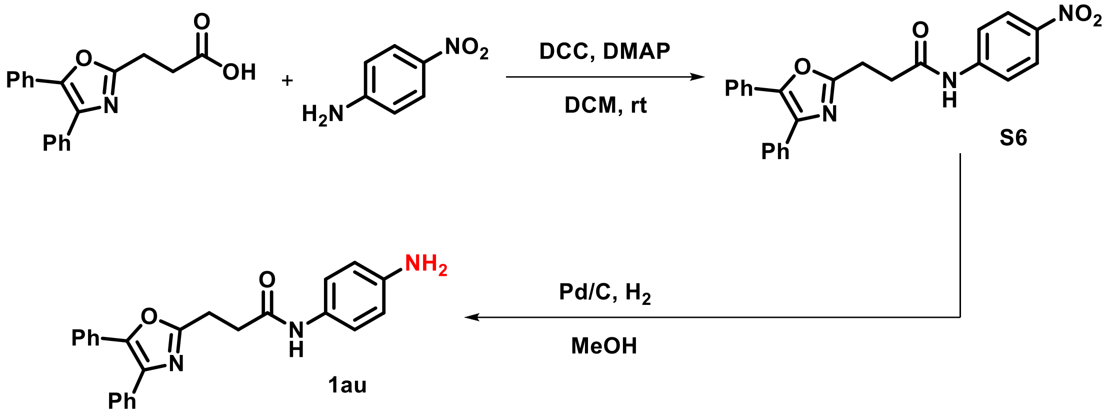

3-(4,5-Diphenyloxazol-2-yl)-*N*-(4-nitrophenyl)propanamide S6: A solution of Oxaprozin (5 mmol, 1.47 g), 4-Nitroaniline (6 mmol, 830 mg), *N*,*N*-dimethylpyridin-4-amine (DMAP, 1 mmol, 123 mg) and DCM (20 mL) were stirred at room temperature (rt). Dicyclohexylmethanediimine (DCC, 10 mmol, 2.06 g) was added in one portion manner. After the reaction by TLC, removing dichloromethane (DCM) by rotary evaporation and dissolving the residue with EA (30 mL). Then filtered, the filtrate was extracted with citric acid (10 wt%), saturated sodium chloride respectively. Dry the combined organic phase with anhydrous $MgSO_4$ and then evaporate under reduced pressure. The residue was chromatographed on silica gel using petroleum ether/ethyl acetate (V_{PE}/V_{EA} = 2/1) as eluent to give **S6** (1.35 g, 65%) with white solid.

***N*-(4-aminophenyl)-3-(4,5-diphenyloxazol-2-yl)propanamide 1au:** A mixture of **S6** (3 mmol, 1.24 g), Pd/C (10 wt%, 124 mg) was stirred in MeOH (6 mL) under H_2 atmosphere (> 4 atm) at 50 ^oC about 5 hours. The mixture was filtered and the filtrate evaporated to dryness. The residue was chromatographed on silica gel using petroleum ether/ethyl acetate (V_{PE}/V_{EA} = 1/1) as eluent to give **1au** (908 mg, 79%) with white solid. **^{1}H NMR** (400 MHz, DMSO-*d6*) δ 9.65 (brs, 1H), 7.57-7.51 (m, 4H), 7.44-7.35 (m, 6H), 7.22 (d, *J* = 8.0 Hz, 2H), 6.49 (d, *J* = 8.0 Hz, 2H), 4.83 (brs, 2H), 3.12 (t, *J* = 7.2 Hz, 2H), 2.80 (t, *J* = 7.2 Hz, 2H). **^{13}C NMR** (100 MHz, DMSO-*d6*) δ 168.4, 162.7, 144.7, 144.5, 134.3, 132.1, 128.9, 128.8, 128.6, 128.5, 128.4, 128.1, 127.3, 126.3, 120.9, 113.8, 32.5, 23.3. **IR** (neat) 3334, 1648, 1531, 1516, 1288, 1244,

1059 cm^{-1}. **MS** (ESI) Calculated for $C_{24}H_{22}N_3O_2$ $(M+H)^+$: 384.2; Found 384.4.

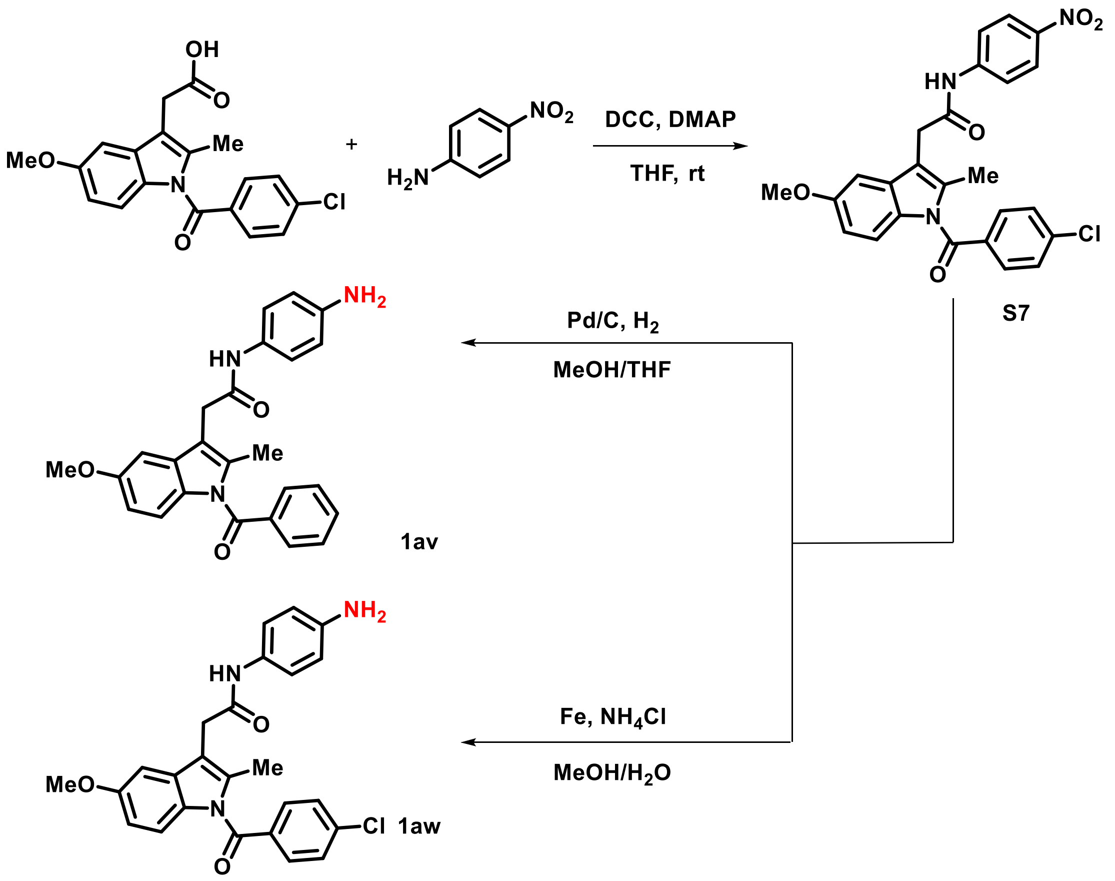

2-(1-(4-chlorobenzoyl)-5-methoxy-2-methyl-1H-indol-3-yl)-*N*-(4-nitrophenyl)acetamide S7: A solution of Indometacin (3 mmol, 1.07 g), 4-Nitroaniline (3.6 mmol, 500 mg), N,N-dimethylpyridin-4-amine (DMAP, 0.6 mmol, 73 mg) and DCM (20 mL) were stirred at room temperature (rt). Dicyclohexylmethanediimine (DCC, 10 mmol, 2.06 g) was added in one portion manner. After the reaction by TLC, removing dichloromethane (DCM) by rotary evaporation and dissolving the residue with EA (30 mL). Then filtered, the filtrate was extracted with citric acid (10 wt%), saturated sodium chloride respectively. Dry the combined organic phase with anhydrous $MgSO_4$ and then evaporate under reduced pressure. The residue was chromatographed on silica gel using petroleum ether/ethyl acetate (V_{PE}/V_{EA} = 5/1 to 2/1) as eluent to give **S7** (815 mg, 57%) with white solid.

***N*-(4-aminophenyl)-2-(1-benzoyl-5-methoxy-2-methyl-1H-indol-3-yl)acetamide 1av:** A mixture of **S7** (1 mmol, 478 g), Pd/C (10 wt%, 48 mg) was stirred in MeOH/THF (3 mL/2 mL) under H_2 atmosphere (> 10 atm) at 50 °C about 10 hours.

The mixture was filtered and the filtrate evaporated to dryness. The residue was chromatographed on silica gel using petroleum ether/ethyl acetate (V_{PE}/V_{EA} = 1/1) as eluent to give **1av** (314 mg, 76%) with white solid. **^{1}H NMR** (500 MHz, DMSO-*d6*) δ 9.77 (brs, 1H), 7.70 (t, *J* = 7.5 Hz, 1H), 7.67 (d, *J* = 10.0 Hz, 2H), 7.57 (t, *J* = 7.5 Hz, 2H), 7.22-7.19 (m, 3H), 6.88 (d, *J* = 9.0 Hz, 1H), 6.67 (dd, J_1 = 9.0 Hz, J_2 = 2.5 Hz, 1H), 6.49 (d, *J* = 8.5 Hz, 2H), 4.85 (brs, 2H), 3.75 (s, 3H), 3.65 (s, 2H), 2.27 (s, 3H). **^{13}C NMR** (125 MHz, DMSO-*d6*) δ 168.9, 167.5, 156.6, 155.5, 144.9, 135.6, 135.3, 132.8, 130.4, 129.2, 128.9, 128.3, 121.1, 114.5, 114.3, 113.8, 111.0, 101.9, 55.4, 31.9, 13.4. **IR** (neat) 3421, 3315, 2944, 2831, 1449, 1024, 826 cm^{-1}. **MS** (ESI) Calculated for $C_{25}H_{24}N_3O_3$ $(M+H)^+$: 414.2; Found 414.2.

***N*-(4-aminophenyl)-2-(1-(4-chlorobenzoyl)-5-methoxy-2-methyl-1H-indol-3-yl)acetamide 1aw:** A mixture of **S7** (1 mmol, 478 g), Fe (5 mmol, 280 mg) and NH_4Cl (5 mmol, 268 mg) was stirred in MeOH/H_2O (4 mL/2 mL) at 60 ^{o}C about 24 hours. The mixture was filtered and the residue washed by DCM. The filtrate was extracted with saturated sodium chloride and then evaporated to dryness. The residue was chromatographed on silica gel using petroleum ether/ethyl acetate (V_{PE}/V_{EA} = 1/1) as eluent to give **1aw** (260 mg, 58%) with white solid. **^{1}H NMR** (400 MHz, DMSO-*d6*) δ 9.79 (brs, 1H), 7.68 (d, *J* = 8.0 Hz, 2H), 7.64 (d, *J* = 8.0 Hz, 2H), 7.21-7.19 (m, 3H), 6.92 (d, *J* = 8.0 Hz, 1H), 6.70 (dd, J_1 = 8.8 Hz, J_2 = 2.4 Hz, 1H), 6.48 (d, *J* = 8.0 Hz, 2H), 4.90 (brs, 2H), 3.74 (s, 3H), 3.64 (s, 2H), 2.27 (s, 3H). **^{13}C NMR** (100 MHz, DMSO-*d6*) δ 167.9, 167.5, 156.6, 155.6, 137.6, 135.3, 134.3, 131.2, 130.9, 130.3, 129.1, 128.3, 121.3, 114.6, 114.5, 113.8, 111.1, 102.1, 55.5, 31.9, 13.5. **IR** (neat) 3470, 3254, 2255, 1668, 1052, 1025, 1005 cm^{-1}. **MS** (ESI) Calculated for $C_{25}H_{21}ClN_3O_3$ $(M-H)^-$: 446.1; Found 446.3.

VI. General Procedures

A. Parallel Photoreactor

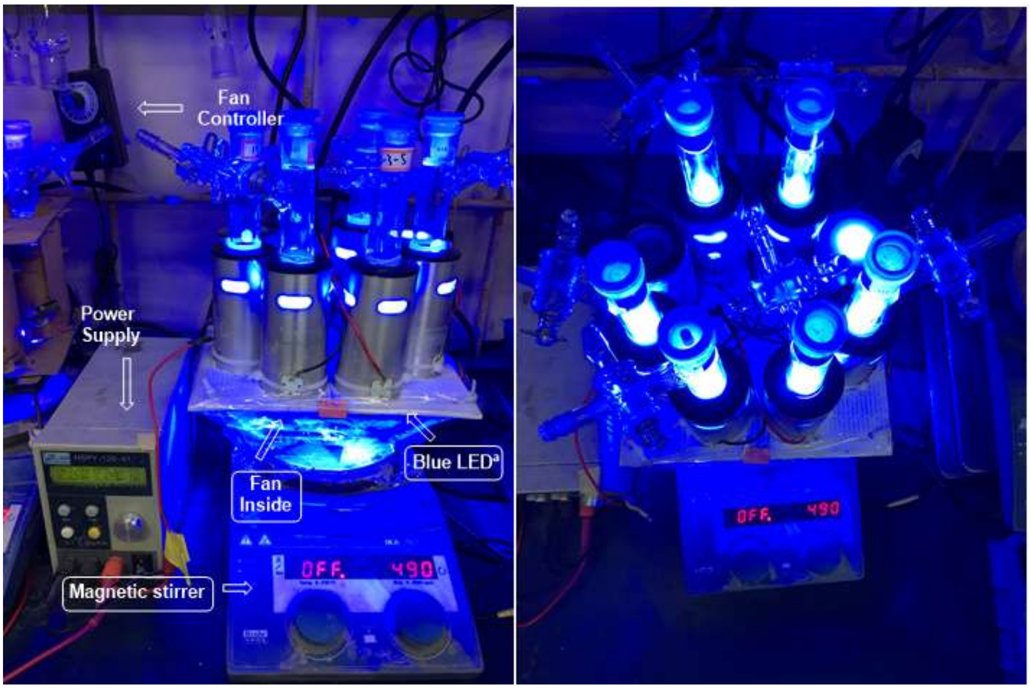

Figure S17: Parallel Photoreactor

[a]There are three LEDs in each cell. The second LED is on the opposite side of shown LED, the third one is on the bottom part of the cell.

B. General Procedures for Phenols

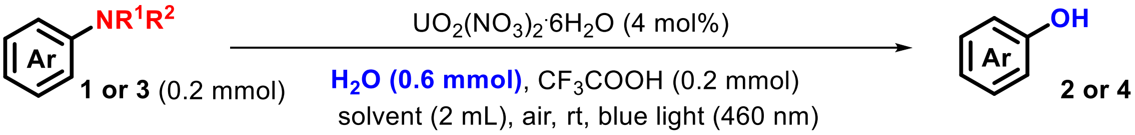

To a 50 mL Schlenk tube, CF_3COOH (TFA) (0.2 mmol, 22.9 mg/15 μL) was added to the mixture of **1 or 3** (0.2 mmol), $UO_2(NO_3)_2\cdot6H_2O$ (4 mol%, 4.0 mg) and solvent (2 mL). Subsequently, the reaction was irradiated by blue light (460 nm) in parallel reactor. Ethyl acetate (EA) was added to the reaction mixture to quench it. The pH of mixture was adjusted to 10~11 by 2*N* NaOH (aq), then extracting with saturated NaCl (aq) (10 mL, three times) and collecting the gathered aqueous phase. Subsequently,

the pH of gathered aqueous phase was adjusted to 4~5 by 2*N* HCl (aq), then extracting with EA (30 mL, three times) and collecting the gathered organic phase. Drying the organic layer over $MgSO_4$ (dry) and Evaporating under reduced pressure. Purify the crude residue by column chromatography on silica gel using PE/EA to afford **2** or **4**. For detailed modification, please see the corresponding procedure.

VII. Procedures and Data for Fig. 3, 4, and 5.

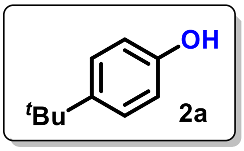

4-(*Tert*-butyl)phenol 2a: 4-(*tert*-butyl)aniline **1a** (0.2 mmol, 29.9 mg), $UO_2(NO_3)_2{\cdot}6H_2O$ (4 mol%/0.008 mmol, 4 mg), TFA (0.2 mmol, 22.8 mg) and H_2O (0.6 mmol, 10.8 mg) were stirred in CH_3CN (2 mL) at room temperature for 24 h under blue light (460 nm, 6 W) in a parallel reactor in the air. **2a** (25.5 mg, 85%) was obtained through column chromatography (V_{PE}/V_{EA} = 10/1) as a white solid. **^{1}H NMR** (400 MHz, $CDCl_3$) δ 7.27 (d, *J* = 8.0 Hz, 2H), 6.78 (d, *J* = 8.0 Hz, 2H), 4.09 (brs, 1H), 1.30 (s, 9H). **^{13}C NMR** (100 MHz, $CDCl_3$) δ 153.1, 143.6, 126.4, 114.8, 34.1, 31.5. **IR** (neat) 3245, 1599, 1512, 1361, 1236, 1182, 825 cm^{-1}. GCMS (EI) m/z, $[M]^+$ = 150.[9]

10 mmol Scale Parallel Photoreactor

The photoreactor used in this research from Shanghai 3S Technology Co. Ltd.

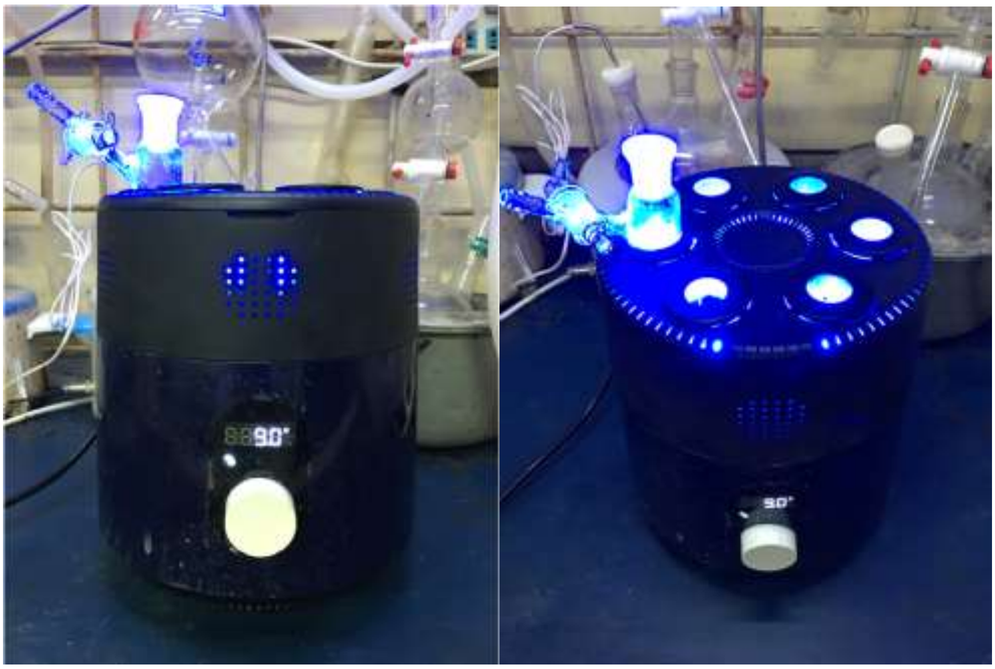

10 mmol scale: 4-(*tert*-butyl)aniline **1a** (10.0 mmol, 1.49 g), $UO_2(NO_3)_2{\cdot}6H_2O$ (3 mol%/0.3 mmol, 150 mg), TFA (25 mmol, 2.85 g) and H_2O (30 mmol, 540 mg) were stirred in HFIP (20 mL) at room temperature for 6 days under blue light (460 nm) in a parallel reactor in the air. Ethyl acetate (EA, 50 mL) was added to the reaction mixture to quench it. The pH of mixture was adjusted to 10~11 by 2*N* NaOH (aq), then extracting with saturated NaCl (aq) (30 mL, three times) and collecting the

gathered aqueous phase. Subsequently, the pH of gathered aqueous phase was adjusted to 4~5 by 2*N* HCl (aq), then extracting with EA (30 mL, three times) and collecting the gathered organic phase. Drying the organic layer over $MgSO_4$ (dry) and Evaporating under reduced pressure. Purify the crude residue by column chromatography on silica gel using PE/EA (10/1) to afford **2a** (888 mg, 59%) with yellowish-white solid.

Flow Photoreactor

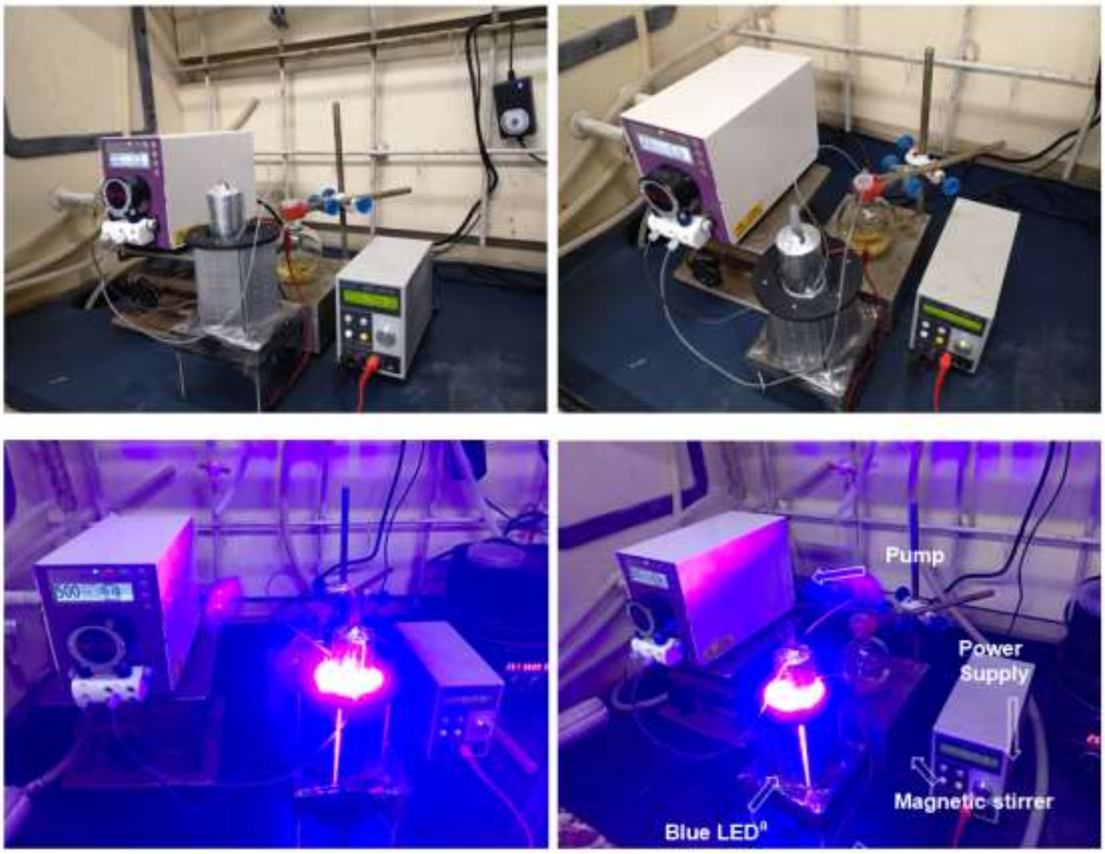

To a 250 mL bottle, a solution of 4-(*tert*-butyl)aniline **1a** (20.0 mmol, 2.98 g), $UO_2(NO_3)_2·6H_2O$ (2 mol%/0.4 mmol, 201 mg), TFA (40 mmol, 4.6 g) and H_2O (60 mmol, 1.08 g) in CH_3CN/HFIP (25 mL/25 mL) was stirred in the air. The solution was pumped into a flow micro tube by a pump (0.5 mL/min), which was made of PTFE tubing (O.D. = 2 mm, I.D. = 1 mm, length = 5.68 m, volume = 4.45 mL), and returned to Schlenk tube with the same pump. This circulatory system was irradiated by blue light (435 nm, 54 W) about 23 hours (the temperature was below 30 ^{o}C). After the reaction, DCM (10 mL) was pumped into a flow micro tube to quench it and wash

the tube. The solvent was removed in vacuo and the residue was dissolved in EA (30 mL). The pH of mixture was adjusted to 10~11 by 2*N* NaOH (aq), then extracting with saturated NaCl (aq) (30 mL, three times) and collecting the gathered aqueous phase. Subsequently, the pH of gathered aqueous phase was adjusted to 4~5 by 2*N* HCl (aq), then extracting with EA (30 mL, three times) and collecting the gathered organic phase. Drying the organic layer over $MgSO_4$ (dry) and Evaporating under reduced pressure. Purify the crude residue by column chromatography on silica gel using PE/EA (10/1) to afford **2a** (78%, 2.34 g) with pale brown liquid.

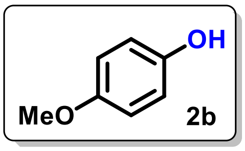

4-Methoxyphenol 2b: 4-methoxyaniline **1b** (0.2 mmol, 24.6 mg), $UO_2(NO_3)_2{\cdot}6H_2O$ (4 mol%/0.008 mmol, 4 mg), TFA (0.2 mmol, 22.8 mg) and H_2O (0.6 mmol, 10.8 mg) were stirred in CH_3CN (2 mL) at room temperature for 24 h under blue light (460 nm) in a parallel reactor in the air. **2b** (22.3 mg, 90%) was obtained through column chromatography (V_{PE}/V_{EA} = 10/1) as a white solid. **^{1}H NMR** (400 MHz, $CDCl_3$) δ 6.81-6.76 (m, 4H), 4.96 (brs, 1H), 3.77 (s, 3H). **^{13}C NMR** (100 MHz, $CDCl_3$) δ 153.5, 149.5, 116.1, 114.9, 55.9. **IR** (neat) 3350, 1510, 1443, 1371, 1232, 1032, 824 cm^{-1}. GCMS (EI) m/z, $[M]^+$ = 124.[9]

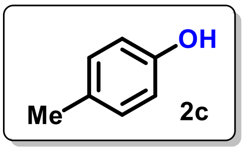

***p*-Cresol 2c**: *p*-toluidine **1c** (0.2 mmol, 21.4 mg), $UO_2(OAc)_2{\cdot}2H_2O$ (4 mol%/0.008 mmol, 3.4 mg), TFA (0.2 mmol, 22.8 mg) and H_2O (0.6 mmol, 10.8 mg) were stirred in CH_3CN (2 mL) at room temperature for 2 days under blue light (460 nm) in a parallel reactor on N_2 atmosphere. **2c** (13.6 mg, 63%) was obtained through column chromatography (V_{PE}/V_{EA} = 10/1) as a brown liquid. **^{1}H NMR** (400 MHz, $CDCl_3$) δ 7.05 (d, *J* = 8.0 Hz, 2H), 6.74 (d, *J* = 8.0 Hz, 2H), 4.53 (brs, 1H), 2.28 (s, 3H). **^{13}C NMR** (100 MHz, $CDCl_3$) δ 153.3, 130.0, 129.9, 115.1, 20.4. **IR** (neat) 3387, 2955, 2924, 1458, 1377, 1262, 1186 cm^{-1}. GCMS (EI) m/z, $[M]^+$ = 108.[9]

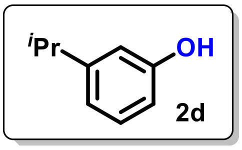

3-Isopropylphenol 2d: 3-isopropylaniline **1d** (0.2 mmol, 27.0 mg), $UO_2(OAc)_2{\cdot}2H_2O$ (4 mol%/0.008 mmol, 3.4 mg), TFA (0.2 mmol, 22.8 mg) and H_2O (0.6 mmol, 10.8 mg) were stirred in CH_3CN (2 mL) at room temperature for 2 days under blue light (460 nm) in a parallel reactor on N_2 atmosphere. **2d** (15.8 mg, 58%) was obtained through column chromatography (V_{PE}/V_{EA} = 10/1) as a brown liquid. **^{1}H NMR** (400 MHz, $CDCl_3$) δ 7.20 (t, J = 8.0 Hz, 1H), 6.86 (d, J = 8.0 Hz, 1H), 6.78 (s, 1H), 6.72 (ddd, J_1 = 8.0 Hz, J_2 = 2.4 Hz, J_3 = 0.8 Hz), 5.05 (brs, 1H), 2.92-2.85 (m, 1H), 1.26 (d, J = 4.0 Hz, 6H). **^{13}C NMR** (100 MHz, $CDCl_3$) δ 155.3, 150.9, 129.4, 118.9, 113.5, 112.7, 33.9, 23.8. **IR** (neat) 3383, 2961, 1589, 1454, 1231, 1179, 930 cm^{-1}. GCMS (EI) m/z, $[M]^+$ = 136.[10]

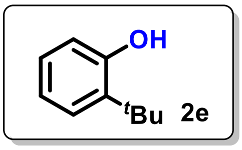

2-(Tert-butyl)phenol 2e: 2-(tert-butyl)aniline **1e** (0.2 mmol, 29.8 mg), $UO_2(NO_3)_2{\cdot}6H_2O$ (4 mol%/0.008 mmol, 4 mg), TFA (0.4 mmol, 45.6 mg) and H_2O (0.6 mmol, 10.8 mg) were stirred in CH_3CN (2 mL) at room temperature for 2 days under blue light (460 nm) in a parallel reactor in the air. **2e** (18.9 mg, 63%) was obtained through column chromatography (V_{PE}/V_{EA} = 10/1) as a brown liquid. **^{1}H NMR** (400 MHz, $CDCl_3$) δ 7.31 (d, J = 8.0 Hz, 1H), 7.10 (t, J = 8.0 Hz, 1H), 6.91 (t, J = 8.0 Hz, 1H), 6.68 (d, J = 8.0 Hz, 1H), 4.88 (brs, 1H), 1.44 (s, 9H). **^{13}C NMR** (100 MHz, $CDCl_3$) δ 154.2, 136.1, 127.0, 126.9, 120.6, 116.5, 34.5, 29.6. **IR** (neat) 3534, 2955, 1443, 1364, 1333, 1248, 1186 cm^{-1}. GCMS (EI) m/z, $[M]^+$ = 150.[11]

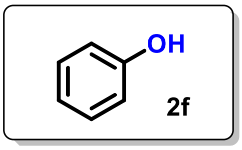

Phenol 2f: aniline **1f** (0.2 mmol, 18.6 mg), $UO_2(OAc)_2{\cdot}2H_2O$ (4 mol%/0.008 mmol, 3.4 mg), TFA (0.2 mmol, 22.8 mg) and H_2O (0.6 mmol, 10.8 mg) were stirred in CH_3CN (2 mL) at room temperature for 2 days under blue light (460 nm) in a parallel reactor on N_2 atmosphere. **2f** (11.5 mg, 61%) was obtained through column chromatography (V_{PE}/V_{EA} = 10/1) as a brown liquid. **^{1}H NMR** (400 MHz, $CDCl_3$) δ 7.25 (d, J = 8.0 Hz, 2H), 6.93 (t, J = 8.0 Hz, 1H), 6.83 (d, J = 8.0 Hz, 2H), 4.88 (brs, 1H). **^{13}C NMR**

(100 MHz, $CDCl_3$) δ 155.4, 129.7, 120.8, 115.3. **IR** (neat) 3360, 1477, 1449, 1379, 1241, 1089, 1046 cm^{-1}. GCMS (EI) m/z, $[M]^+$ = 94.[9]

Flow Chemistry: To a 250 mL bottle, a solution of aniline **1f** (20.0 mmol, 1.86 g), $UO_2(NO_3)_2·6H_2O$ (2 mol%/0.4 mmol, 201 mg), TFA (60 mmol, 6.9 g) and H_2O (60 mmol, 1.08 g) in HFIP (50 mL) was stirred in the air. The solution was pumped into a flow micro tube by a pump (0.5 mL/min), which was made of PTFE tubing (O.D. = 2 mm, I.D. = 1 mm, length = 5.68 m, volume = 4.45 mL), and returned to Schlenk tube with the same pump. This circulatory system was irradiated by blue light (430 nm, 54 W) about 2 days (the temperature was below 30 ^oC). After the reaction, DCM (10 mL) was pumped into a flow micro tube to quench it and wash the tube. The solvent was removed in vacuo and the residue was dissolved in EA (30 mL). The pH of mixture was adjusted to 10~11 by 2*N* NaOH (aq), then extracting with saturated NaCl (aq) (30 mL, three times) and collecting the gathered aqueous phase. Subsequently, the pH of gathered aqueous phase was adjusted to 4~5 by 2*N* HCl (aq), then extracting with EA (30 mL, three times) and collecting the gathered organic phase. Drying the organic layer over $MgSO_4$ (dry) and Evaporating under reduced pressure. Purify the crude residue by column chromatography on silica gel using PE/EA (20/1) to afford **2f** (60%, 1.13 g) with brown liquid.

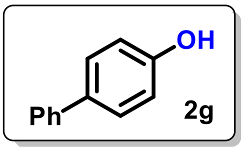

[1,1'-Biphenyl]-4-ol 2g: [1,1'-biphenyl]-4-amine **1g** (0.2 mmol, 33.8 mg), $UO_2(NO_3)_2·6H_2O$ (4 mol%/0.008 mmol, 4 mg), TFA (0.2 mmol, 22.8 mg) and H_2O (0.6 mmol, 10.8 mg) were stirred in CH_3CN (2 mL) at room temperature for 2 days under blue light (460 nm) in a parallel reactor in the air. **2g** (26.5 mg, 78%) was obtained through column chromatography (V_{PE}/V_{EA} = 10/1) as a white solid. **^{1}H NMR** (400 MHz, $CDCl_3$) δ 7.55 (d, *J* = 8.0 Hz, 2H), 7.49 (d, *J* = 8.0 Hz, 2H), 7.42 (t, *J* = 8.0 Hz, 2H), 7.31 (t, *J* = 8.0 Hz, 1H), 6.92 (d, *J* = 8.0 Hz, 2H), 4.80 (brs, 1H). **^{13}C NMR** (100 MHz, $CDCl_3$) δ 155.1, 140.8, 134.0, 128.7, 128.4, 126.71, 126.69, 115.6. **IR** (neat) 3345, 1458, 1422, 1236, 1112, 1080, 831 cm^{-1}. GCMS (EI) m/z, $[M]^+$ = 170.[12]

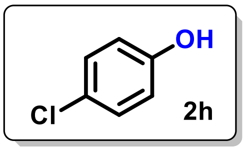

4-Chlorophenol 2h: 4-chloroaniline **1h** (0.2 mmol, 25.4 mg), $UO_2(NO_3)_2{\cdot}6H_2O$ (4 mol%/0.008 mmol, 4 mg), TFA (0.2 mmol, 22.8 mg) and H_2O (0.6 mmol, 10.8 mg) were stirred in HFIP (2 mL) at room temperature for 36 h under blue light (460 nm) in a parallel reactor in the air. **2h** (18.7 mg, 73%) was obtained through column chromatography (V_{PE}/V_{EA} = 10/1) as a white solid. **^{1}H NMR** (400 MHz, $CDCl_3$) δ 7.19 (d, *J* = 8.0 Hz, 2H), 6.77 (d, *J* = 8.0 Hz, 2H), 5.05 (brs, 1H). **^{13}C NMR** (100 MHz, $CDCl_3$) δ 154.1, 129.5, 125.7, 116.7. **IR** (neat) 3361, 1494, 1232, 1094, 1076, 1057, 826 cm^{-1}. GCMS (EI) m/z, $[M]^+$ = 128.[13]

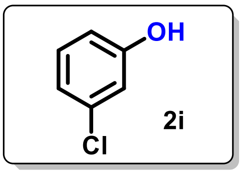

3-Chlorophenol 2i: 3-chloroaniline **1i** (0.2 mmol, 25.4 mg), $UO_2(NO_3)_2{\cdot}6H_2O$ (4 mol%/0.008 mmol, 4 mg), TFA (0.2 mmol, 22.8 mg) and H_2O (0.6 mmol, 10.8 mg) were stirred in HFIP (2 mL) at room temperature for 2 days under blue light (460 nm) in a parallel reactor in the air. **2i** (14.3 mg, 56%) was obtained through column chromatography (V_{PE}/V_{EA} = 10/1) as a white solid. **^{1}H NMR** (400 MHz, $CDCl_3$) δ 7.15 (t, *J* = 8.0 Hz, 1H), 6.92 (d, *J* = 8.0 Hz, 1H), 6.86 (t, *J* = 2.4 Hz, 1H), 6.72 (ddd, J_1 = 8.4 Hz, J_2 = 2.4 Hz, J_3 = 0.8 Hz, 1H), 5.07 (brs, 1H). **^{13}C NMR** (100 MHz, $CDCl_3$) δ 156.3, 134.9, 130.4, 121.0, 115.9, 113.8. **IR** (neat) 3341, 1589, 1473, 1445, 1248, 1215, 887 cm^{-1}. GCMS (EI) m/z, $[M]^+$ = 128.[14]

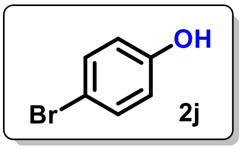

4-Bromophenol 2j: 4-bromoaniline **1j** (0.2 mmol, 34.0 mg), $UO_2(NO_3)_2{\cdot}6H_2O$ (4 mol%/0.008 mmol, 4 mg), TFA (0.2 mmol, 22.8 mg) and H_2O (0.6 mmol, 10.8 mg) were stirred in HFIP (2 mL) at room temperature for 36 h under blue light (460 nm) in a parallel reactor in the air. **2j** (26.1 mg, 76%) was obtained through column chromatography (V_{PE}/V_{EA} = 10/1) as a white solid. **^{1}H NMR** (400 MHz, $CDCl_3$) δ 7.33 (d, *J* = 8.0 Hz, 2H), 6.72 (d, *J* = 8.0 Hz, 2H), 5.22 (brs, 1H). **^{13}C NMR** (100 MHz, $CDCl_3$) δ 154.6, 132.5, 117.2, 112.9. **IR** (neat) 3336, 1489, 1433, 1240, 1169, 1070, 822 cm^{-1}. GCMS (EI) m/z, $[M]^+$ = 172.[9]

10 mmol scale, 4-bromoaniline **1j** (10.0 mmol, 1.71 g), $UO_2(NO_3)_2{\cdot}6H_2O$ (4 mol%/0.4 mmol, 200 mg), TFA (10 mmol, 1.14 g) and H_2O (30 mmol, 540 mg) were stirred in HFIP (40 mL) at room temperature for 7 days under blue light (460 nm) in a parallel reactor in the air. Ethyl acetate (EA, 50 mL) was added to the reaction mixture to quench it. The pH of mixture was adjusted to 10~11 by 2*N* NaOH (aq), then extracting with saturated NaCl (aq) (30 mL, three times) and collecting the gathered aqueous phase. Subsequently, the pH of gathered aqueous phase was adjusted to 4~5 by 2*N* HCl (aq), then extracting with EA (30 mL, three times) and collecting the gathered organic phase. Drying the organic layer over $MgSO_4$ (dry) and Evaporating under reduced pressure. Purify the crude residue by column chromatography on silica gel using PE/EA (10/1) to afford **2j** (883 mg, 51%) with white solid.

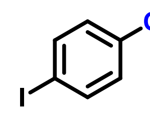
2k

4-Iodophenol 2k: 4-iodoaniline **1k** (0.2 mmol, 43.8 mg), $UO_2(NO_3)_2{\cdot}6H_2O$ (4 mol%/0.008 mmol, 4 mg), TFA (0.2 mmol, 22.8 mg) and H_2O (0.6 mmol, 10.8 mg) were stirred in HFIP (2 mL) at room temperature for 36 h under blue light (460 nm) in a parallel reactor in the air. **2k** (40.9 mg, 93%) was obtained through column chromatography (V_{PE}/V_{EA} = 10/1) as a white solid. **^{1}H NMR** (400 MHz, $CDCl_3$) δ 7.51 (d, *J* = 8.0 Hz, 2H), 6.63 (d, *J* = 8.0 Hz, 2H), 5.10 (brs, 1H). **^{13}C NMR** (100 MHz, $CDCl_3$) δ 155.3, 138.4, 117.8, 82.7. **IR** (neat) 3387, 2359, 1485, 1425, 1211, 1175, 822 cm^{-1}. GCMS (EI) m/z, $[M]^+$ = 220.[9]

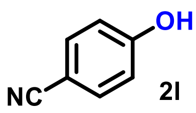
2l

4-Hydroxybenzonitrile 2l: 4-aminobenzonitrile **1l** (0.2 mmol, 23.6 mg), $UO_2(OAc)_2{\cdot}2H_2O$ (4 mol%/0.008 mmol, 3.4 mg), TFA (0.2 mmol, 22.8 mg) and H_2O (0.6 mmol, 10.8 mg) were stirred in HFIP (2 mL) at room temperature for 3.5 days under blue light (460 nm) in a parallel reactor on N_2 atmosphere. **2l** (16.2 mg, 68%) was obtained through column chromatography (V_{PE}/V_{EA} = 10/1) as a white solid. **^{1}H NMR** (400 MHz, $CDCl_3$) δ 7.55 (d, *J* = 8.0 Hz, 2H), 6.93 (d, *J* = 8.0 Hz, 2H), 6.45 (brs, 1H). **^{13}C NMR** (100

MHz, $CDCl_3$) δ 159.9, 134.3, 119.2, 116.4, 103.4. **IR** (neat) 3265, 1611, 1585, 1508, 1439, 1283, 1165 cm^{-1}. GCMS (EI) m/z, $[M]^+$ = 119.[9]

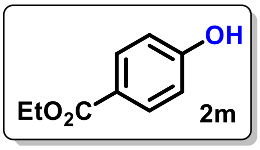

Ethyl 4-hydroxybenzoate 2m: ethyl 4-aminobenzoate **1m** (0.2 mmol, 33.0 mg), $UO_2(NO_3)_2·6H_2O$ (4 mol%/0.008 mmol, 4 mg), TFA (0.4 mmol, 45.6 mg) and H_2O (0.6 mmol, 10.8 mg) were stirred in HFIP (2 mL) at room temperature for 3.5 days under blue light (460 nm) in a parallel reactor on N_2 atmosphere. **2m** (21.9 mg, 66%) was obtained through column chromatography (V_{PE}/V_{EA} = 10/1) as a white solid. **^{1}H NMR** (400 MHz, $CDCl_3$) δ 7.96 (d, *J* = 8.0 Hz, 2H), 6.89 (d, *J* = 8.0 Hz, 2H), 6.46 (brs, 1H), 4.36 (q, *J* = 7.2 Hz, 2H), 1.38 (t, *J* = 7.2 Hz, 3H). **^{13}C NMR** (100 MHz, $CDCl_3$) δ 167.1, 160.3, 131.9, 122.5, 115.3, 61.0, 14.3. **IR** (neat) 3211, 3190, 2924, 1670, 1447, 1369, 1285 cm^{-1}. GCMS (EI) m/z, $[M]^+$ = 166.[13]

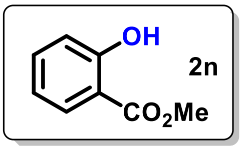

Methyl 2-hydroxybenzoate 2n: methyl 2-aminobenzoate **1n** (0.2 mmol, 30.2 mg), $UO_2(OAc)_2·2H_2O$ (4 mol%/0.008 mmol, 3.4 mg), TFA (0.2 mmol, 22.8 mg) and H_2O (0.6 mmol, 10.8 mg) were stirred in HFIP (2 mL) at room temperature for 3.5 days under blue light (460 nm) in a parallel reactor on N_2 atmosphere. **2n** (19.5 mg, 64%) was obtained through column chromatography (V_{PE}/V_{EA} = 10/1) as a brown liquid. **^{1}H NMR** (400 MHz, $CDCl_3$) δ 10.77 (brs, 1H), 7.83 (dd, J_1 = 8.0 Hz, J_2 = 1.6 Hz, 1H), 7.47-7.43 (m, 1H), 6.98 (dd, J_1 = 8.4 Hz, J_2 = 0.8 Hz, 1H), 6.90-6.86 (m, 1H), 3.94 (s, 3H). **^{13}C NMR** (100 MHz, $CDCl_3$) δ 170.6, 161.5, 135.7, 129.9, 119.1, 117.5, 112.3, 52.2. **IR** (neat) 3186, 1676, 1439, 1327, 1304, 1252, 1213 cm^{-1}. GCMS (EI) m/z, $[M]^+$ = 152.[15]

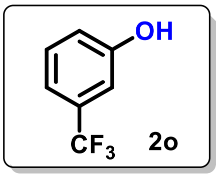

3-(Trifluoromethyl)phenol 2o: 3-(trifluoromethyl)aniline **1o** (0.2 mmol, 32.2 mg), $UO_2(NO_3)_2·6H_2O$ (4 mol%/0.008 mmol, 4 mg), TFA (0.4 mmol, 45.6 mg) and H_2O (0.6 mmol, 10.8 mg) were stirred in HFIP (2 mL) at room temperature for 2 days under blue light (460 nm) in a parallel reactor on N_2 atmosphere. **2o** (18.8 mg, 58%) was

obtained through column chromatography (V_{PE}/V_{EA} = 10/1) as a white solid. **^{1}H NMR** (400 MHz, $CDCl_3$) δ 7.36 (t, *J* = 8.0 Hz, 1H), 7.21 (d, *J* = 8.0 Hz, 1H), 7.09 (s, 1H), 7.01 (dd, J_1 = 8.0 Hz, J_2 = 2.4 Hz, 1H), 5.22 (brs, 1H). **^{13}C NMR** (100 MHz, $CDCl_3$) δ 155.6, 132.1 (q, *J* = 32 Hz), 130.3, 123.8 (q, *J* = 270 Hz), 118.8 (d, *J* = 1 Hz), 117.7 (q, *J* = 4 Hz), 112.3 (q, *J* = 4 Hz). **^{19}F NMR** (376 MHz, $CDCl_3$) δ -62.84. **IR** (neat) 3374, 1460, 1333, 1246, 1171, 1128, 980 cm^{-1}. GCMS (EI) m/z, $[M]^+$ = 162.[14]

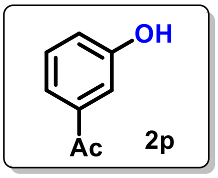

1-(3-Hydroxyphenyl)ethan-1-one 2p: 1-(3-aminophenyl)ethan-1-one **1p** (0.2 mmol, 27.0 mg), $UO_2(NO_3)_2 \cdot 6H_2O$ (4 mol%/0.008 mmol, 4 mg), TFA (0.4 mmol, 45.6 mg) and H_2O (0.6 mmol, 10.8 mg) were stirred in HFIP (2 mL) at room temperature for 2 days under blue light (460 nm) in a parallel reactor on N_2 atmosphere. **2p** (15.8 mg, 58%) was obtained through column chromatography (V_{PE}/V_{EA} = 10/1) as a white solid. **^{1}H NMR** (400 MHz, $CDCl_3$) δ 7.53-7.51 (m, 2H), 7.35 (t, *J* = 8.0 Hz, 1H), 7.12-7.09 (m, 1H), 6.29 (brs, 1H), 2.61 (s, 3H). **^{13}C NMR** (100 MHz, $CDCl_3$) δ 198.9, 156.2, 138.4, 129.9, 121.1, 120.7, 114.6, 26.8. **IR** (neat) 3173, 1578, 1490, 1367, 1296, 1259, 1221 cm^{-1}. GCMS (EI) m/z, $[M]^+$ = 136.[13]

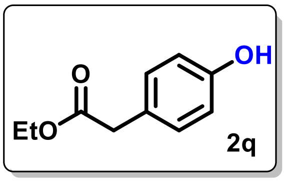

Ethyl 2-(4-hydroxyphenyl)acetate 2q: ethyl 2-(4-aminophenyl)acetate **1q** (0.2 mmol, 35.8 mg), $UO_2(NO_3)_2 \cdot 6H_2O$ (4 mol%/0.008 mmol, 4.0 mg), TFA (0.2 mmol, 22.8 mg) and H_2O (0.6 mmol, 10.8 mg) were stirred in HFIP (2 mL) at room temperature for 2 days under blue light (460 nm) in a parallel reactor on N_2 atmosphere. **2q** (28.8 mg, 80%) was obtained through column chromatography (V_{PE}/V_{EA} = 10/1) as a white solid. **^{1}H NMR** (400 MHz, $CDCl_3$) δ 7.10 (d, *J* = 8.0 Hz, 2H), 6.73 (d, *J* = 8.0 Hz, 2H), 4.70 (brs, 1H), 4.16 (q, *J* = 7.2 Hz, 2H), 3.54 (s, 2H), 1.25 (t, *J* = 7.2 Hz, 3H). **^{13}C NMR** (100 MHz, $CDCl_3$) δ 172.8, 154.9, 130.3, 125.7, 115.5, 61.1, 40.5, 14.1. **IR** (neat) 3351, 1705, 1701, 1445, 1298, 1221, 1155 cm^{-1}. **HRMS** (ESI) Calculated for $C_{10}H_{12}O_3Na$ $(M+Na)^+$: 203.0679;

Found 203.0680.

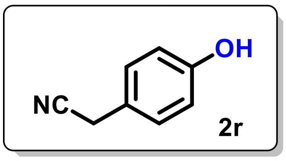

2-(4-Hydroxyphenyl)acetonitrile 2r: 2-(4-aminophenyl)acetonitrile **1r** (0.2 mmol, 26.4 mg), $UO_2(NO_3)_2 \cdot 6H_2O$ (4 mol%/0.008 mmol, 4.0 mg), TFA (0.2 mmol, 22.8 mg) and H_2O (0.6 mmol, 10.8 mg) were stirred in HFIP (2 mL) at room temperature for 2 days under blue light (460 nm) in a parallel reactor on N_2 atmosphere. **2r** (13.8 mg, 52%) was obtained through column chromatography (V_{PE}/V_{EA} = 10/1) as a white solid. **^{1}H NMR** (400 MHz, $CDCl_3$) δ 7.16 (d, *J* = 8.0 Hz, 2H), 6.83 (d, *J* = 8.0 Hz, 2H), 4.75 (brs, 1H), 3.67 (s, 2H). **^{13}C NMR** (100 MHz, $CDCl_3$) δ 155.7, 129.3, 121.6, 118.3, 116.1, 22.8. **IR** (neat) 3337, 1514, 1444, 1359, 1261, 1217, 1175 cm^{-1}. GCMS (EI) m/z, $[M]^+$ = 133.[13]

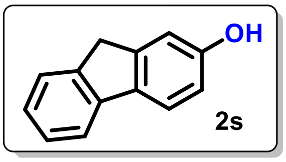

9*H*-fluoren-2-ol 2s: 9*H*-fluoren-2-amine **1s** (0.2 mmol, 36.2 mg), $UO_2(NO_3)_2 \cdot 6H_2O$ (4 mol%/0.008 mmol, 4.0 mg), TFA (0.2 mmol, 22.8 mg) and H_2O (0.6 mmol, 10.8 mg) were stirred in CH_3CN (2 mL) at room temperature for 2.5 days under blue light (460 nm) in a parallel reactor on N_2 atmosphere. **2s** (21.8 mg, 60%) was obtained through column chromatography (V_{PE}/V_{EA} = 10/1) as a white solid. **^{1}H NMR** (400 MHz, $CDCl_3$) δ 7.69 (d, *J* = 7.6 Hz, 1H), 7.64 (d, *J* = 8.0 Hz, 1H), 7.50 (d, *J* = 7.2 Hz, 1H), 7.35 (t, *J* = 7.2 Hz, 1H), 7.26-7.22 (m, 1H), 7.03-7.02 (m, 1H), 6.86 (dd, J_1 = 8.0 Hz, J_2 = 2.0 Hz, 1H), 4.96 (brs, 1H), 3.85 (s, 2H). **^{13}C NMR** (100 MHz, $CDCl_3$) δ 154.9, 145.3, 142.6, 141.5, 134.9, 126.7, 125.6, 124.8, 120.7, 119.0, 114.1, 112.2, 36.9. **IR** (neat) 3263, 1578, 1483, 1381, 1302, 1244, 1175 cm^{-1}. GCMS (EI) m/z, $[M]^+$ = 182.[13]

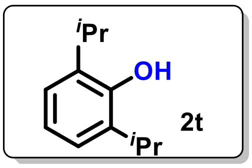

2,6-Diisopropylphenol 2t: 2,6-diisopropylaniline **1t** (0.2 mmol, 35.4 mg), $UO_2(NO_3)_2 \cdot 6H_2O$ (4 mol%/0.008 mmol, 4 mg), TFA (0.4 mmol, 45.6 mg) and H_2O (0.6 mmol, 10.8 mg) were stirred in HFIP (2 mL) at room temperature for 2 days under blue light (460 nm) in a parallel reactor on N_2 atmosphere. **2t** (17.1 mg, 48%) was obtained

through column chromatography (V_{PE}/V_{EA} = 10/1) as a white solid. **^{1}H NMR** (400 MHz, $CDCl_3$) δ 7.08 (d, *J* = 8.0 Hz, 1H), 6.92 (t, *J* = 7.6 Hz, 1H), 4.81 (brs, 1H), 3.21-3.14 (m, 2H), 1.29 (d, *J* = 8.0 Hz, 12H). **^{13}C NMR** (100 MHz, $CDCl_3$) δ 149.9, 133.6, 123.4, 120.6, 27.1, 22.7. **IR** (neat) 3556, 2961, 1458, 1309, 1258, 1199, 1148 cm^{-1}. GCMS (EI) m/z, $[M]^+$ = 178.[16]

Flow Chemistry: To a 250 mL bottle, a solution of aniline **1t** (20.0 mmol, 3.55 g), $UO_2(NO_3)_2{\cdot}6H_2O$ (2 mol%/0.4 mmol, 201 mg), TFA (40 mmol, 4.6 g) and H_2O (60 mmol, 1.08 g) in HFIP/CH_3CN (25 mL/25 mL) was added in the air. The solution was pumped into a flow micro tube by a pump (0.5 mL/min), which was made of PTFE tubing (O.D. = 2 mm, I.D. = 1 mm, length = 5.68 m, volume = 4.45 mL), and returned to Schlenk tube with the same pump. This circulatory system was irradiated by blue LED (430 nm, 54 W) about 24 hours (the temperature was below 30 ^{o}C). After the reaction, DCM (10 mL) was pumped into a flow micro tube to quench it and wash the tube. The solvent was removed in vacuo and the residue was dissolved in EA (30 mL). The pH of mixture was adjusted to 10~11 by 2*N* NaOH (aq), then extracting with saturated NaCl (aq) (30 mL, three times) and collecting the gathered aqueous phase. Subsequently, the pH of gathered aqueous phase was adjusted to 4~5 by 2*N* HCl (aq), then extracting with EA (30 mL, three times) and collecting the gathered organic phase. Drying the organic layer over $MgSO_4$ (dry) and Evaporating under reduced pressure. Purify the crude residue by column chromatography on silica gel using PE/EA (10/1) to afford **2t** (76%, 2.70 g) with brown liquid.

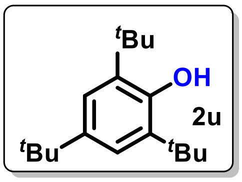

2,4,6-Tri-tert-butylphenol 2u: 2,4,6-tri-tert-butylaniline **1u** (0.2 mmol, 52.3 mg), $UO_2(NO_3)_2{\cdot}6H_2O$ (4 mol%/0.008 mmol, 4.0 mg), TFA (0.2 mmol, 22.8 mg) and H_2O (0.6 mmol, 10.8 mg) were stirred in CH_3NO_2 (2 mL) at room temperature for 2.5 days under blue light (460 nm) in a parallel reactor in the air. **2u** (28.8 mg, 55%) was obtained through column chromatography (V_{PE}/V_{EA} = 10/1) as a white solid. **^{1}H NMR** (400 MHz, $CDCl_3$) δ 7.21 (s, 2H), 5.05 (brs, 1H), 1.46 (s, 18H), 1.31 (s, 9H).

^{13}C NMR (100 MHz, $CDCl_3$) δ 151.3, 141.4, 134.9, 121.9, 34.6, 31.7, 30.4. **IR** (neat) 3643, 2928, 1435, 1359, 1234, 1204, 1159 cm^{-1}. GCMS (EI) m/z, $[M]^+$ = 262.[17]

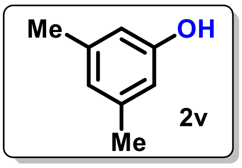

3,5-Dimethylphenol 2v: 3,5-dimethylaniline **1v** (0.2 mmol, 24.2 mg), $UO_2(NO_3)_2 \cdot 6H_2O$ (4 mol%/0.008 mmol, 4 mg), TFA (0.2 mmol, 22.8 mg) and H_2O (0.6 mmol, 10.8 mg) were stirred in CH_3CN (2 mL) at room temperature for 2.5 days under blue light (460 nm) in a parallel reactor on N_2 atmosphere. **2v** (13.4 mg, 55%) was obtained through column chromatography (V_{PE}/V_{EA} = 10/1) as a brown liquid. **^{1}H NMR** (400 MHz, $CDCl_3$) δ 6.58 (s, 1H), 6.46 (s, 2H), 4.57 (brs, 1H), 2.27 (s, 6H). **^{13}C NMR** (100 MHz, $CDCl_3$) δ 155.4, 139.5, 122.5, 113.0, 21.2. **IR** (neat) 2959, 2928, 1742, 1491, 1449, 1373, 1238 cm^{-1}. GCMS (EI) m/z, $[M]^+$ = 122.[9]

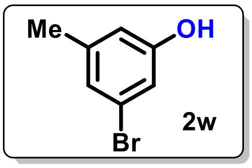

3-Bromo-5-methylphenol 2w: 3-bromo-5-methylaniline **1w** (0.2 mmol, 37.0 mg), $UO_2(OAc)_2 \cdot 2H_2O$ (4 mol%/0.008 mmol, 3.4 mg), TFA (0.2 mmol, 22.8 mg) and H_2O (0.6 mmol, 10.8 mg) were stirred in HFIP (2 mL) at room temperature for 2 days under blue light (460 nm) in a parallel reactor on N_2 atmosphere. **2w** (17.9 mg, 48%) was obtained through column chromatography (V_{PE}/V_{EA} = 10/1) as a brown solid. **^{1}H NMR** (400 MHz, $CDCl_3$) δ 6.91 (s, 1H), 6.82 (s, 1H), 6.58 (s, 1H), 4.92 (brs, 1H), 2.27 (s, 3H). **^{13}C NMR** (100 MHz, $CDCl_3$) δ 156.1, 141.4, 124.8, 122.4, 115.8, 115.0, 21.1. **IR** (neat) 3325, 1603, 1578, 1458, 1452, 1275, 1155 cm^{-1}. GCMS (EI) m/z, $[M]^+$ = 186.[18]

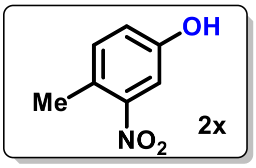

4-Methyl-3-nitrophenol 2x: 4-methyl-3-nitroaniline **1x** (0.2 mmol, 30.4 mg), $UO_2(OAc)_2 \cdot 2H_2O$ (8 mol%/0.016 mmol, 6.8 mg), TFA (0.4 mmol, 45.6 mg) and H_2O (0.6 mmol, 10.8 mg) were stirred in HFIP (2 mL) at room temperature for 3.5 days under blue light (460 nm) in a parallel reactor on N_2 atmosphere. **2x** (19.3 mg, 63%) was obtained through column chromatography (V_{PE}/V_{EA} = 5/1) as a white solid. **^{1}H**

NMR (400 MHz, DMSO-*d6*) δ 10.13 (brs, 1H), 7.33 (d, *J* = 2.8 Hz, 1H), 7.28 (d, *J* = 8.0 Hz, 2H), 7.03 (dd, J_1 = 8.4 Hz, J_2 = 2.4 Hz, 1H), 2.38 (s, 3H). **^{13}C NMR** (100 MHz, DMSO-*d6*) δ 156.0, 149.1, 133.6, 122.6, 120.9, 110.4, 18.7. **IR** (neat) 3399, 1514, 1491, 1327, 1294, 1215, 1192 cm^{-1}. GCMS (EI) m/z, $[M]^+$ = 153.[19]

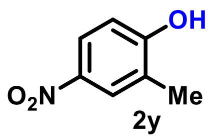

2y

2-Methyl-4-nitrophenol 2y: 2-methyl-4-nitrophenol **1y** (0.2 mmol, 30.4 mg), $UO_2(OAc)_2 \cdot 2H_2O$ (8 mol%/0.016 mmol, 6.8 mg), TFA (0.4 mmol, 45.6 mg) and H_2O (0.6 mmol, 10.8 mg) were stirred in HFIP (2 mL) at room temperature for 4 days under blue light (460 nm) in a parallel reactor on N_2 atmosphere. **2y** (12.5 mg, 41%) was obtained through column chromatography (V_{PE}/V_{EA} = 5/1) as a white solid. **^{1}H NMR** (400 MHz, $CDCl_3$) δ 8.07 (d, *J* = 2.8 Hz, 1H), 8.01 (dd, J_1 = 8.8 Hz, J_2 = 2.8 Hz, 1H), 6.86 (d, *J* = 8.8 Hz, 1H), 5.95 (brs, 1H), 2.32 (s, 3H). **^{13}C NMR** (100 MHz, $CDCl_3$) δ 159.7 (d, *J* = 7 Hz), 141.3, 126.9, 125.1 (d, *J* = 4 Hz), 123.7, 114.7, 15.8. **IR** (neat) 3236, 1659, 1608, 1454, 1298, 1223, 1209. GCMS (EI) m/z, $[M]^+$ = 153.[20]

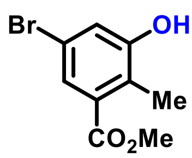

2z

Methyl 5-bromo-3-hydroxy-2-methylbenzoate 2z: methyl 3-amino-5-bromo-2-methylbenzoate **1z** (0.2 mmol, 48.6 mg), $UO_2(OAc)_2 \cdot 2H_2O$ (8 mol%/0.016 mmol, 6.8 mg), TFA (0.4 mmol, 45.6 mg) and H_2O (0.6 mmol, 10.8 mg) were stirred in HFIP (2 mL) at room temperature for 4 days under blue light (460 nm) in a parallel reactor on N_2 atmosphere. **2z** (21.0 mg, 43%) was obtained through column chromatography (V_{PE}/V_{EA} = 5/1) as a white solid. **^{1}H NMR** (400 MHz, DMSO-*d6*) δ 10.26 (brs, 1H), 7.30 (d, *J* = 2.0 Hz, 1H), 7.14 (d, *J* = 2.0 Hz, 2H), 3.81 (s, 3H), 2.49 (s, 3H). **^{13}C NMR** (100 MHz, DMSO-*d6*) δ 166.6, 157.2, 133.1, 124.8, 122.3, 120.1, 117.9, 52.2, 12.4. **IR** (neat) 3310, 1695, 1574, 1352, 1265, 1233, 1053 cm^{-1}. **HRMS** (ESI) Calculated for $C_9H_8O_3Br$ $(M-H)^-$: 242.9662; Found 242.9628.

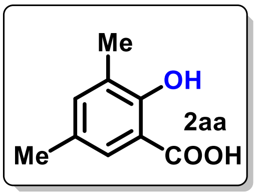

2-Hydroxy-3,5-dimethylbenzoic acid 2aa: 2-amino-3,5-dimethylbenzoic acid **1aa** (0.2 mmol, 33.0 mg), $UO_2(OAc)_2{\cdot}2H_2O$ (8 mol%/0.016 mmol, 6.8 mg), TFA (0.4 mmol, 45.6 mg) and H_2O (0.6 mmol, 10.8 mg) were stirred in HFIP (2 mL) at room temperature for 3.5 days under blue light (460 nm) in a parallel reactor on N_2 atmosphere. **2aa** (14.9 mg, 45%) was obtained through column chromatography (V_{PE}/V_{EA} = 2/1) as a white solid. **^{1}H NMR** (400 MHz, DMSO-*d6*) δ 13.77 (brs, 1H), 11.37 (brs, 1H), 7.43 (s, 1H), 7.21 (s, 1H), 2.20 (s, 3H), 2.14 (s, 3H). **^{13}C NMR** (100 MHz, DMSO-*d6*) δ 172.5, 157.6, 137.3, 127.3, 127.0, 125.3, 111.5, 19.9, 15.2. **IR** (neat) 3493, 3420, 1281, 1053, 1024, 1005, 758 cm^{-1}. **HRMS** (ESI) Calculated for $C_9H_9O_3$ $(M-H)^-$: 165.0557; Found 165.0525.

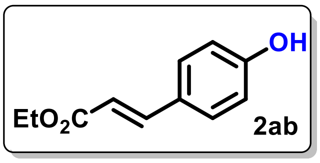

Ethyl (*E*)-3-(4-hydroxyphenyl)acrylate 2ab: ethyl (*E*)-3-(4-aminophenyl)acrylate **1ab** (0.2 mmol, 38.2 mg), $UO_2(OAc)_2{\cdot}2H_2O$ (4 mol%/0.008 mmol, 3.4 mg), TFA (0.2 mmol, 22.8 mg) and H_2O (0.6 mmol, 10.8 mg) were stirred in HFIP (2 mL) at room temperature for 2 days under blue light (460 nm) in a parallel reactor on N_2 atmosphere. **2ab** (23.0 mg, 60%) was obtained through column chromatography (V_{PE}/V_{EA} = 10/1) as a white solid. **^{1}H NMR** (400 MHz, $CDCl_3$) δ 7.64 (d, *J* = 16.0 Hz, 1H), 7.41 (d, *J* = 8.0 Hz, 2H), 6.87 (d, *J* = 16.0 Hz, 2H), 6.29 (d, *J* = 16.0 Hz, 1H), 4.27 (q, *J* = 8.0 Hz, 2H), 1.34 (t, *J* = 8.0 Hz, 3H). **^{13}C NMR** (100 MHz, $CDCl_3$) δ 167.9, 158.1, 144.8, 129.9, 126.9, 115.9, 115.3, 60.6, 14.3. **IR** (neat) 3358, 1707, 1686, 1605, 1325, 1275, 1171 cm^{-1}. **HRMS** (ESI) Calculated for $C_{11}H_{13}O_3$ $(M+H)^+$: 193.0859; Found 193.0860.

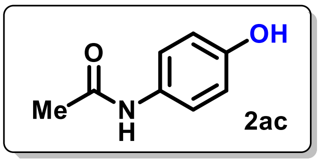

***N*-(4-hydroxyphenyl)acetamide 2ac**: *N*-(4-aminophenyl)acetamide **1ac** (0.2 mmol, 30.0 mg), $UO_2(NO_3)_2{\cdot}6H_2O$ (4 mol%/0.008 mmol, 4.0 mg), TFA (0.2 mmol, 22.8 mg) and H_2O (0.6 mmol, 10.8 mg) were stirred in HFIP (2 mL) at room temperature for 3 days under blue light (460 nm) in a parallel reactor in the air. **2ac**

(24.8 mg, 82%) was obtained through column chromatography (V_{PE}/V_{EA} = 5/1) as a white solid. **^{1}H NMR** (400 MHz, DMSO-*d6*) δ 9.63 (brs, 1H), 9.13 (brs, 1H), 7.32 (d, *J* = 12.0 Hz, 2H), 6.67 (d, *J* = 8.0 Hz, 2H), 1.97 (s, 3H). **^{13}C NMR** (100 MHz, DMSO-*d6*) δ 167.6, 153.1, 131.0, 120.9, 115.0, 23.7. **IR** (neat) 3328, 1653, 1558, 1437, 1327, 1242, 1015 cm^{-1}. GCMS (EI) m/z, $[M]^+$ = 151.[21]

Flow Chemistry: To a 250 mL bottle, a solution of aniline **1ac** (20.0 mmol, 3.06 g), $UO_2(NO_3)_2·6H_2O$ (3 mol%/0.6 mmol, 302 mg), TFA (60 mmol, 6.9 g) and H_2O (60 mmol, 1.08 g) in HFIP/CH_3CN (45 mL/25 mL) was stirred in the air. The solution was pumped into a flow micro tube by a pump (0.5 mL/min), which was made of PTFE tubing (O.D. = 2 mm, I.D. = 1 mm, length = 5.68 m, volume = 4.45 mL), and returned to Schlenk tube with the same pump. This circulatory system was irradiated by blue LED (430 nm, 54 W) about 3 days (the temperature was below 30 ^oC). After the reaction, DCM (10 mL) was pumped into a flow micro tube to quench it and wash the tube. The solvent was removed in vacuo and the residue was dissolved in EA (30 mL). The pH of mixture was adjusted to 10~11 by 2*N* NaOH (aq), then extracting with saturated NaCl (aq) (30 mL, three times) and collecting the gathered aqueous phase. Subsequently, the pH of gathered aqueous phase was adjusted to 4~5 by 2*N* HCl (aq), then extracting with EA (30 mL, three times) and collecting the gathered organic phase. Drying the organic layer over $MgSO_4$ (dry) and Evaporating under reduced pressure. Purify the crude residue by column chromatography on silica gel using PE/EA (1/1) to afford **2ac** (71%, 2.15 g) with light brown solid.

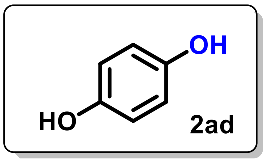

Hydroquinone 2ad: 4-Aminophenol **1ad** (0.2 mmol, 21.8 mg), $UO_2(NO_3)_2·6H_2O$ (4 mol%/0.008 mmol, 4.0 mg), TFA (0.2 mmol, 22.8 mg) and H_2O (0.6 mmol, 10.8 mg) were stirred in HFIP (2 mL) at room temperature for 2 days under blue light (460 nm) in a parallel reactor on N_2 atmosphere. **2ad** (14.1 mg, 64%) was obtained through column chromatography (V_{PE}/V_{EA} = 5/1) as a brown solid. **^{1}H NMR** (400 MHz, DMSO-*d6*) δ 8.62 (brs, 2H), 6.55 (s, 4H). **^{13}C NMR** (100 MHz, DMSO-*d6*) δ 149.8, 115.7. **IR**

(neat) 3395, 1651, 1277, 1049, 1024, 1003, 822 cm^{-1}. GCMS (EI) m/z, $[M]^+$ = 110.[22]

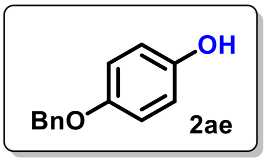

4-(Benzyloxy)phenol 2ae: 4-(benzyloxy)aniline **1ae** (0.2 mmol, 39.8 mg), $UO_2(NO_3)_2 \cdot 6H_2O$ (4 mol%/0.008 mmol, 4.0 mg), TFA (0.2 mmol, 22.8 mg) and H_2O (0.6 mmol, 10.8 mg) were stirred in CH_3CN (2 mL) at room temperature for 2 days under blue light (460 nm) in a parallel reactor on N_2 atmosphere. **2ae** (34.0 mg, 85%) was obtained through column chromatography (V_{PE}/V_{EA} = 10/1) as a white solid. **^{1}H NMR** (400 MHz, $CDCl_3$) δ 7.44-7.37 (m, 4H), 7.35-7.30 (m, 1H), 6.89-6.85 (m, 2H), 6.78-6.74 (m, 2H), 5.02 (s, 2H). **^{13}C NMR** (100 MHz, $CDCl_3$) δ 152.9, 149.7, 137.2, 128.5, 127.9, 127.5, 116.1, 116.0, 70.8. **IR** (neat) 3387, 1506, 1454, 1362, 1209, 1167, 1014 cm^{-1}. **HRMS** (ESI) Calculated for $C_{13}H_{11}O_2$ $(M-H)^-$: 199.0765; Found 199.0747.

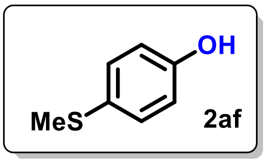

4-(Methylthio)phenol 2af: 4-(methylthio)aniline **1af** (0.2 mmol, 27.8 mg), $UO_2(NO_3)_2 \cdot 6H_2O$ (4 mol%/0.008 mmol, 4.0 mg), TFA (0.2 mmol, 22.8 mg) and H_2O (0.6 mmol, 10.8 mg) were stirred in CH_3CN (2 mL) at room temperature for 2.5 days under blue light (460 nm) in a parallel reactor on N_2 atmosphere. **2af** (11.5 mg, 41%) was obtained through column chromatography (V_{PE}/V_{EA} = 10/1) as a brown liquid. **^{1}H NMR** (400 MHz, $CDCl_3$) δ 7.22 (d, *J* = 8.0 Hz, 2H), 6.78 (d, *J* = 8.0 Hz, 2H), 2.44 (s, 3H). **^{13}C NMR** (100 MHz, $CDCl_3$) δ 151.1, 130.4, 128.9, 116.1, 18.1. **IR** (neat) 3316, 2916, 1582, 1427, 1362, 1219, 1175. GCMS (EI) m/z, $[M]^+$ = 140.[22]

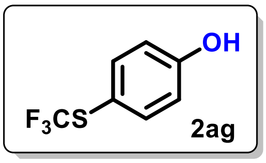

4-((Trifluoromethyl)thio)phenol 2ag: 4-((trifluoromethyl)thio)aniline **1ag** (0.2 mmol, 38.6 mg), $UO_2(OAc)_2 \cdot 2H_2O$ (4 mol%/0.008 mmol, 3.4 mg), TFA (0.2 mmol, 22.8 mg) and H_2O (0.6 mmol, 10.8 mg) were stirred in HFIP (2 mL) at room temperature for 2.5 days under blue light (460 nm) in a parallel reactor on N_2 atmosphere. **2ag** (28.3 mg, 73%) was obtained through column chromatography (V_{PE}/V_{EA} = 5/1) as a red solid. **^{1}H NMR** (400 MHz, $CDCl_3$) δ 7.54 (d, *J* = 8.0 Hz, 2H),

6.87 (d, J = 8.0 Hz, 2H), 5.08 (brs, 1H). **^{13}C NMR** (100 MHz, $CDCl_3$) δ 157.9, 138.6, 129.6 (q, J = 245 Hz), 116.5, 115.2. **^{19}F NMR** (376 MHz, $CDCl_3$) δ -43.91. **IR** (neat) 3260, 1584, 1435, 1248, 1225, 1111, 1088 cm^{-1}. GCMS (EI) m/z, $[M]^+$ = 194.[23]

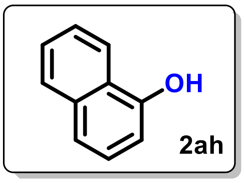

Naphthalen-1-ol 2ah: naphthalen-1-amine **1ah** (0.2 mmol, 28.6 mg), $UO_2(NO_3)_2 \cdot 6H_2O$ (4 mol%/0.008 mmol, 4.0 mg), TFA (0.2 mmol, 22.8 mg) and H_2O (0.6 mmol, 10.8 mg) were stirred in CH_3CN (2 mL) at room temperature for 3 days under blue light (460 nm) in a parallel reactor on N_2 atmosphere. **2ah** (15.6 mg, 54%) was obtained through column chromatography (V_{PE}/V_{EA} = 10/1) as a white solid. **^{1}H NMR** (400 MHz, $CDCl_3$) δ 8.21-8.18 (m, 1H), 7.84-7.81 (m, 1H), 7.53-7.44 (m, 3H), 7.32 (t, J = 7.6 Hz, 1H), 6.82 (dd, J_1 = 7.2 Hz, J_2 = 0.8 Hz, 1H), 5.36 (brs, 1H). **^{13}C NMR** (100 MHz, $CDCl_3$) δ 151.4, 134.8, 127.7, 126.4, 125.8, 125.2, 124.4, 121.5, 120.7, 108.6. **IR** (neat) 3284, 1578, 1362, 1303, 1267, 1238, 1148 cm^{-1}. GCMS (EI) m/z, $[M]^+$ = 144.[22]

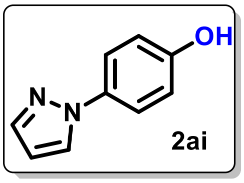

4-(1H-pyrazol-1-yl)phenol 2ai: 4-(1H-pyrazol-1-yl)aniline **1ai** (0.2 mmol, 31.8 mg), $UO_2(NO_3)_2 \cdot 6H_2O$ (8 mol%/0.016 mmol, 8.0 mg), TFA (0.4 mmol, 45.6 mg) and H_2O (0.6 mmol, 10.8 mg) were stirred in HFIP (2 mL) at room temperature for 3.5 days under blue light (460 nm) in a parallel reactor on N_2 atmosphere. **2ai** (25.6 mg, 80%) was obtained through column chromatography (V_{PE}/V_{EA} = 5/1) as a brown solid. **^{1}H NMR** (400 MHz, $CDCl_3$) δ 8.78 (brs, 1H), 7.75-7.73 (m, 2H), 7.37 (d, J = 8.0 Hz, 2H), 6.80 (d, J = 8.0 Hz, 2H), 6.44 (t, J = 2.4 Hz, 1H). **^{13}C NMR** (100 MHz, $CDCl_3$) δ 156.0, 140.2, 132.6, 128.2, 122.1, 116.3, 107.1. **IR** (neat) 3325, 3124, 1522, 1460, 1234, 1055, 1036 cm^{-1}. **HRMS** (ESI) Calculated for $C_9H_9N_2O$ $(M+H)^+$: 161.0709; Found 161.0709.

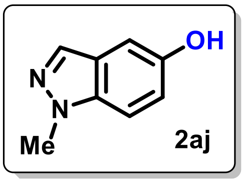

1-Methyl-1H-indazol-5-ol 2aj: 1-methyl-1H-indazol-5-amine

1aj (0.2 mmol, 29.4 mg), $UO_2(NO_3)_2{\cdot}6H_2O$ (8 mol%/0.016 mmol, 8.0 mg), TFA (0.4 mmol, 45.6 mg) and H_2O (0.6 mmol, 10.8 mg) were stirred in HFIP (2 mL) at room temperature for 3.5 days under blue light (460 nm) in a parallel reactor on N_2 atmosphere. **2aj** (12.1 mg, 41%) was obtained through column chromatography (V_{PE}/V_{EA} = 5/1) as a brown solid. **^{1}H NMR** (400 MHz, DMSO-*d6*) δ 9.12 (brs, 1H), 7.79 (s, 1H), 7.42 (d, *J* = 12.0 Hz, 1H), 6.97-6.92 (m, 2H), 3.96 (s, 3H). **^{13}C NMR** (100 MHz, DMSO-*d6*) δ 151.4, 135.1, 130.8, 124.2, 117.7, 110.2, 102.4, 35.3. **IR** (neat) 3160, 1508, 1450, 1308, 1211, 1146, 1003 cm^{-1}. **HRMS** (ESI) Calculated for $C_8H_9N_2O$ $(M+H)^+$: 149.0709; Found 149.0709.

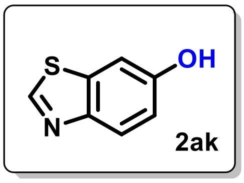

Benzo[d]thiazol-6-ol 2ak: benzo[d]thiazol-6-amine **1ak** (0.2 mmol, 30.0 mg), $UO_2(NO_3)_2{\cdot}6H_2O$ (8 mol%/0.016 mmol, 8.0 mg), TFA (0.4 mmol, 45.6 mg) and H_2O (0.6 mmol, 10.8 mg) were stirred in HFIP (2 mL) at room temperature for 3.5 days under blue light (460 nm) in a parallel reactor on N_2 atmosphere. **2ak** (18.1 mg, 60%) was obtained through column chromatography (V_{PE}/V_{EA} = 5/1) as a brown solid. **^{1}H NMR** (400 MHz, DMSO-*d6*) δ 9.74 (brs, 1H), 9.08 (s, 1H), 7.87 (d, *J* = 8.0 Hz, 1H), 7.43 (s, 1H), 7.00 (d, *J* = 8.0 Hz, 1H). **^{13}C NMR** (100 MHz, DMSO-*d6*) δ 155.7, 152.1, 146.6, 134.9, 123.4, 115.9, 106.7. **IR** (neat) 3067, 2671, 1601, 1435, 1373, 1246, 1188. **HRMS** (ESI) Calculated for C_7H_6NOS $(M+H)^+$: 152.0165; Found 156.0165.

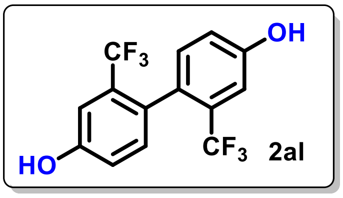

2,2'-Bis(trifluoromethyl)-[1,1'-biphenyl]-4,4'-diol 2al: 2,2'-bis(trifluoromethyl)-[1,1'-biphenyl]-4,4'-diamine **1al** (0.2 mmol, 64.0 mg), $UO_2(NO_3)_2{\cdot}6H_2O$ (4 mol%/0.008 mmol, 4.0 mg), TFA (0.4 mmol, 45.6 mg) and H_2O (0.6 mmol, 10.8 mg) were stirred in HFIP (2 mL) at room temperature for 4 days under blue light (460 nm) in a parallel reactor on N_2 atmosphere. **2al** (48.3 mg, 75%) was obtained through column chromatography (V_{PE}/V_{EA} = 5/1) as a brown solid. **^{1}H NMR** (400 MHz, DMSO-*d6*) δ 10.16 (brs, 2H), 7.12-7.08 (m, 4H), 7.02 (dd, J_1 = 8.0 Hz, J_2

= 4.0 Hz, 2H). **^{13}C NMR** (100 MHz, DMSO-*d6*) δ 157.1, 133.6, 128.7 (q, *J* = 29 Hz), 127.3, 123.8 (q, *J* = 272 Hz), 118.0, 112.3. **^{19}F NMR** (376 MHz, DMSO-*d6*) δ -57.41. **IR** (neat) 3287, 1591, 1489, 1476, 1364, 1315, 1217 cm^{-1}. **HRMS** (ESI) Calculated for $C_{14}H_7O_2F_6$ $(M-H)^-$: 321.0356; Found 321.0318.

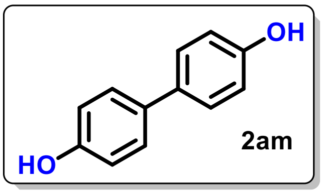

[1,1'-Biphenyl]-4,4'-diol 2am: benzidine **1am** (0.2 mmol, 36.8 mg), $UO_2(NO_3)_2{\cdot}6H_2O$ (4 mol%/0.008 mmol, 4.0 mg), TFA (0.4 mmol, 45.6 mg) and H_2O (0.6 mmol, 10.8 mg) were stirred in CH_3CN (2 mL) at room temperature for 2 days under blue light (460 nm) in a parallel reactor on N_2 atmosphere. **2am** (20.2 mg, 55%) was obtained through column chromatography (V_{PE}/V_{EA} = 5/1) as a brown solid. **^{1}H NMR** (400 MHz, DMSO-*d6*) δ 9.21 (brs, 2H), 6.77 (d, *J* = 8.0 Hz, 4H), 6.71 (d, *J* = 8.0 Hz, 4H). **^{13}C NMR** (100 MHz, DMSO-*d6*) δ 152.9, 150.0, 119.4, 116.1. **IR** (neat) 3391, 1591, 1503, 1425, 1383, 1254, 1175 cm^{-1}. GCMS (EI) m/z, $[M]^+$ = 186.[24]

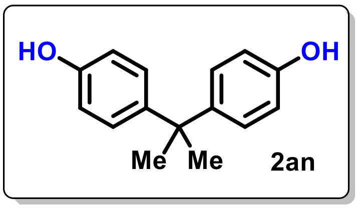

4,4'-(Propane-2,2-diyl)diphenol 2an: 4,4'-(propane-2,2-diyl)dianiline **1an** (0.2 mmol, 45.2 mg), $UO_2(NO_3)_2{\cdot}6H_2O$ (4 mol%/0.008 mmol, 4.0 mg), TFA (0.4 mmol, 45.6 mg) and H_2O (0.6 mmol, 10.8 mg) were stirred in CH_3NO_2 (2 mL) at room temperature for 2 days under blue light (460 nm) in a parallel reactor on N_2 atmosphere. **2an** (27.4 mg, 60%) was obtained through column chromatography (V_{PE}/V_{EA} = 5/1) as a white solid. **^{1}H NMR** (400 MHz, DMSO-*d6*) δ 9.13 (brs, 2H), 6.98 (d, *J* = 8.0 Hz, 4H), 6.64 (d, *J* = 8.0 Hz, 4H), 1.53 (s, 6H). **^{13}C NMR** (100 MHz, DMSO-*d6*) δ 154.9, 141.1, 127.3, 114.6, 40.9, 30.9. **IR** (neat) 3260, 1659, 1271, 1233, 1051, 1024, 1005 cm^{-1}. GCMS (EI) m/z, $[M]^+$ = 228.[25]

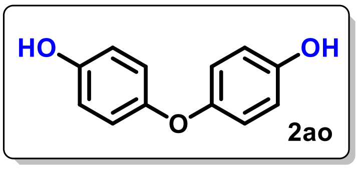

4,4'-Oxydiphenol 2ao: 4,4'-oxydianiline **1ao** (0.2 mmol, 40.0 mg), $UO_2(NO_3)_2{\cdot}6H_2O$ (4 mol%/0.008

mmol, 4.0 mg), TFA (0.4 mmol, 45.6 mg) and H_2O (0.6 mmol, 10.8 mg) were stirred in CH_3CN (2 mL) at room temperature for 2 days under blue light (460 nm) in a parallel reactor on N_2 atmosphere. **2ao** (17.8 mg, 44%) was obtained through column chromatography (V_{PE}/V_{EA} = 5/1) as a white solid. **^{1}H NMR** (400 MHz, DMSO-*d6*) δ 9.21 (brs, 2H), 6.77 (d, *J* = 8.0 Hz, 4H), 6.71 (d, *J* = 8.0 Hz, 4H). **^{13}C NMR** (100 MHz, DMSO-*d6*) δ 152.9, 150.0, 119.4, 116.1. **IR** (neat) 3338, 1497, 1449, 1368, 1209, 1192, 827 cm^{-1}. **HRMS** (ESI) Calculated for $C_{12}H_9O_3$ $(M-H)^-$: 201.0557; Found 201.0525.

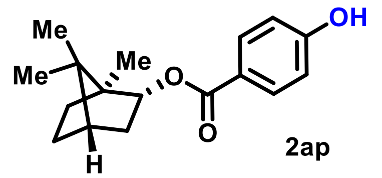

2ap

(*1S,2R,4S*)-1,7,7-trimethylbicyclo[2.2.1]heptan-2-yl 4-hydroxybenzoate 2ap: (*1S,2R,4S*)-1,7,7-trimethylbicyclo[2.2.1]heptan-2-yl 4-aminobenzoate **1ap** (0.1 mmol, 27.3 mg), $UO_2(NO_3)_2{\cdot}6H_2O$ (4 mol%/0.008 mmol, 2.0 mg), TFA (0.1 mmol, 11.4 mg) and H_2O (0.3 mmol, 5.4 mg) were stirred in HFIP (2 mL) at room temperature for 2 days under blue light (460 nm) in a parallel reactor on N_2 atmosphere. **2ap** (11.2 mg, 41%) was obtained through column chromatography (V_{PE}/V_{EA} = 5/1) as a white solid. **^{1}H NMR** (400 MHz, $CDCl_3$) δ 7.97 (d, *J* = 8.0 Hz, 2H), 6.87 (d, *J* = 8.0 Hz, 2H), 5.67 (brs, 1H), 5.11-5.07 (m, 1H), 2.50-2.42 (m, 1H), 2.15-2.09 (m, 1H), 1.84-1.76 (m, 1H), 1.74-1.72 (m, 1H), 1.44-1.36 (m, 1H), 1.32-1.27 (m, 1H), 1.13-1.09 (m, 1H), 0.96 (s, 3H), 0.91 (s, 3H), 0.90 (s, 3H). **^{13}C NMR** (100 MHz, $CDCl_3$) δ 166.7, 159.7, 131.8, 123.5, 115.1, 80.3, 49.1, 47.9, 45.0, 36.9, 28.1, 27.4, 19.7, 18.9, 13.6. **IR** (neat) 3362, 1682, 1314, 1285, 1233, 1165, 1126 cm^{-1}. **HRMS** (ESI) Calculated for $C_{17}H_{21}O_3$ $(M-H)^-$: 273.1496; Found 273.1459.

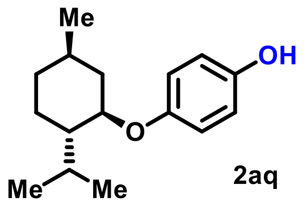

2aq

4-(((*1R,2S,5R*)-2-isopropyl-5-methylcyclohexyl)oxy)phenol 2aq: 4-(((*1R,2S,5R*)-2-isopropyl-5-methylcyclohexyl)oxy)aniline **1aq** (0.1 mmol, 24.7 mg), $UO_2(NO_3)_2{\cdot}6H_2O$ (4

mol%/0.008 mmol, 2.0 mg), TFA (0.1 mmol, 11.4 mg) and H_2O (0.3 mmol, 5.4 mg) were stirred in HFIP (1 mL) at room temperature for 24 hours under blue light (460 nm) in a parallel reactor on N_2 atmosphere. **2aq** (18.6 mg, 75%) was obtained through column chromatography (V_{PE}/V_{EA} = 10/1) as a white solid. **^{1}H NMR** (400 MHz, $CDCl_3$) δ 6.80 (d, *J* = 8.0 Hz, 2H), 6.74 (d, *J* = 8.0 Hz, 2H), 4.72 (brs, 1H), 3.89-3.83 (m, 1H), 2.30-2.23 (m, 1H), 2.14-2.08 (m, 1H), 1.71-1.68 (m, 1H), 1.50-1.39 (m, 1H), 1.09-0.98 (m, 2H), 0.96-0.86 (m, 7H), 0.79 (d, *J* = 8.0 Hz, 3H). **^{13}C NMR** (100 MHz, $CDCl_3$) δ 152.5, 149.5, 117.8, 116.1, 79.1, 48.2, 40.5, 34.5, 31.5, 25.9, 23.6, 22.1, 20.8, 16.5. **IR** (neat) 3371, 2924, 1506, 1449, 1369, 1211, 1099. **HRMS** (ESI) Calculated for $C_{16}H_{23}O_2$ $(M-H)^-$: 247.1704; Found 247.1670.

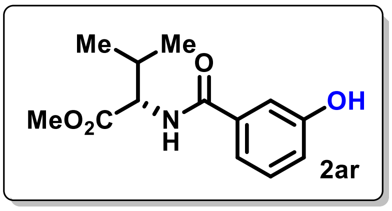

Methyl (3-hydroxybenzoyl)-*L*-valinate 2ar: methyl (3-aminobenzoyl)-*L*-valinate **1ar** (0.1 mmol, 25.0 mg), $UO_2(NO_3)_2 \cdot 6H_2O$ (4 mol%/0.008 mmol, 2.0 mg), TFA (0.1 mmol, 11.4 mg) and H_2O (0.6 mmol, 5.4 mg) were stirred in HFIP (2 mL) at room temperature for 24 hours under blue light (460 nm) in a parallel reactor on N_2 atmosphere. **2ai** (13.1 mg, 52%) was obtained through column chromatography (V_{PE}/V_{EA} = 2/1) as a white solid. **^{1}H NMR** (400 MHz, $CDCl_3$) δ 7.49 (brs, 1H), 7.24-7.22 (m, 2H), 7.02-6.99 (m, 1H), 6.87 (d, *J* = 8.4 Hz, 1H), 6.12 (brs, 1H), 4.74-4.71 (m, 1H), 3.76 (s, 3H), 2.30-2.22 (m, 1H), 0.98 (t, *J* = 7.2 Hz, 6H). **^{13}C NMR** (100 MHz, $CDCl_3$) δ 172.7, 168.0, 157.1, 134.8, 129.8, 119.4, 117.9, 114.7, 57.8, 52.3, 31.5, 18.9, 17.9. **IR** (neat) 3318, 2963, 1736, 1643, 1529, 1314, 1213 cm^{-1}. **HRMS** (ESI) Calculated for $C_{13}H_{18}NO_4$ $(M+H)^+$: 252.1230; Found 252.1228.

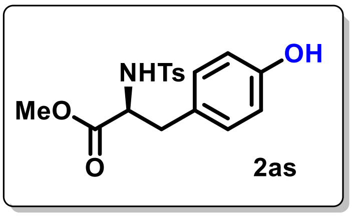

Methyl tosyl-*L*-tyrosinate 2as: methyl (*S*)-3-(4-aminophenyl)-2-((4-methylphenyl)sulfonamido)propanoate **1as** (0.1 mmol, 34.8 mg), $UO_2(NO_3)_2 \cdot 6H_2O$ (4 mol%/0.008 mmol, 2.0 mg), TFA (0.1 mmol, 11.4 mg) and H_2O (0.3 mmol, 5.4 mg) were stirred in CH_3NO_2 (2 mL) at room temperature for

1.5 days under blue light (460 nm) in a parallel reactor on N_2 atmosphere. **2as** (21.6 mg, 62%) was obtained through column chromatography (V_{PE}/V_{EA} = 2/1) as a brown solid. **^{1}H NMR** (400 MHz, $CDCl_3$) δ 7.63 (d, *J* = 8.0 Hz, 2H), 7.24 (d, *J* = 8.0 Hz, 2H), 6.92 (d, *J* = 8.0 Hz, 2H), 6.68 (d, *J* = 8.0 Hz, 2H), 5.35 (brs, 1H), 5.12 (d, *J* = 12.0 Hz, 1H), 4.18-4.13 (m, 1H), 3.49 (s, 3H), 2.96-2.94 (m, 2H), 2.40 (s, 3H). **^{13}C NMR** (100 MHz, $CDCl_3$) δ 171.4, 155.0, 143.7, 136.6, 130.6, 129.6, 127.2, 126.7, 115.5, 56.8, 52.4, 38.5, 21.5. **IR** (neat) 3426, 1751, 1730, 1445, 1339, 1219, 1150 cm^{-1}. **HRMS** (ESI) Calculated for $C_{17}H_{20}NO_5S$ $(M+H)^+$: 350.1057; Found 350.1052.

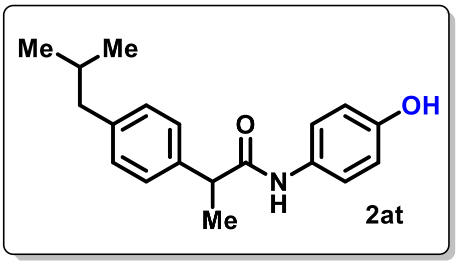

***N*-(4-hydroxyphenyl)-2-(4-isobutylphenyl)propanamide 2at**: *N*-(4-aminophenyl)-2-(4-isobutylphenyl)propanamide **1at** (0.1 mmol, 29.6 mg), $UO_2(NO_3)_2{\cdot}6H_2O$ (4 mol%/0.008 mmol, 2.0 mg), TFA (0.1 mmol, 11.4 mg) and H_2O (0.3 mmol, 5.4 mg) were stirred in HFIP (2 mL) at room temperature for 2.5 days under blue light (460 nm) in a parallel reactor on N_2 atmosphere. **2at** (20.8 mg, 70%) was obtained through column chromatography (V_{PE}/V_{EA} = 5/1) as a white solid. **^{1}H NMR** (400 MHz, DMSO-*d6*) δ 9.75 (brs, 1H), 9.13 (brs, 1H), 7.36 (d, *J* = 8.0 Hz, 2H), 7.28 (d, *J* = 8.0 Hz, 2H), 7.09 (d, *J* = 8.0 Hz, 2H), 6.66 (d, *J* = 8.0 Hz, 2H), 3.75-3.70 (m, 1H), 2.40 (d, *J* = 4.0 Hz, 2H), 1.84-1.74 (m, 1H), 1.38 (d, *J* = 4.0 Hz, 3H), 0.84 (d, *J* = 8.0 Hz, 6H). **^{13}C NMR** (100 MHz, DMSO-*d6*) δ 171.6, 153.2, 139.39, 139.33, 130.9, 128.8, 126.9, 120.8, 114.9, 45.4, 44.2, 29.6, 22.2, 18.7. **IR** (neat) 3285, 1653, 1539, 1508, 1449, 1234, 1163 cm^{-1}. **HRMS** (ESI) Calculated for $C_{19}H_{24}NO_5$ $(M+H)^+$: 298.1802; Found 298.1799.

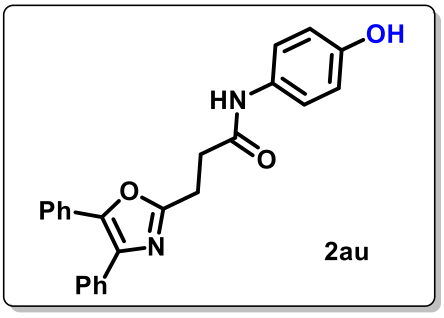

3-(4,5-Diphenyloxazol-2-yl)-*N*-(4-hydroxyphenyl)propanamide 2au: *N*-(4-aminophenyl)-3-(4,5-diphenyloxazol-2-yl)propanamide **1au** (0.1 mmol, 38.3 mg),

$UO_2(NO_3)_2 \cdot 6H_2O$ (4 mol%/0.008 mmol, 2.0 mg), TFA (0.1 mmol, 11.4 mg) and H_2O (0.3 mmol, 5.4 mg) were stirred in HFIP (2 mL) at room temperature for 24 hours under blue light (460 nm) in a parallel reactor on N_2 atmosphere. **2au** (23.0 mg, 60%) was obtained through column chromatography (V_{PE}/V_{EA} = 2/1) as a white solid. **^{1}H NMR** (400 MHz, DMSO-*d6*) δ 9.81 (brs, 1H), 9.13 (brs, 1H), 7.57-7.51 (m, 4H), 7.44-7.35 (m, 8H), 6.68 (d, *J* = 8.0 Hz, 2H), 3.14 (t, *J* = 7.6 Hz, 2H), 2.83 (t, *J* = 7.6 Hz, 2H). **^{13}C NMR** (100 MHz, DMSO-*d6*) δ 168.7, 162.7, 153.2, 144.5, 134.3, 132.0, 130.9, 128.9, 128.8, 128.6, 128.4, 128.1, 127.3, 126.3, 120.8, 115.0, 32.5, 23.2. **IR** (neat) 3414, 3264, 2126, 1279, 1053, 1024, 1005 cm^{-1}. **HRMS** (ESI) Calculated for $C_{24}H_{21}N_2O_3$ $(M+H)^+$: 385.1547; Found 385.1540.

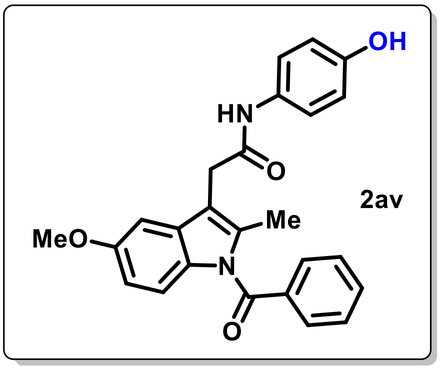

2-(1-Benzoyl-5-methoxy-2-methyl-1*H*-indol-3-yl)-*N*-(4-hydroxyphenyl)acetamide 2av: *N*-(4-aminophenyl)-2-(1-benzoyl-5-methoxy-2-methyl-1*H*-indol-3-yl)acetamide **1av** (0.1 mmol, 41.3 mg), $UO_2(NO_3)_2 \cdot 6H_2O$ (4 mol%/0.008 mmol, 2.0 mg), TFA (0.1 mmol, 11.4 mg) and H_2O (0.3 mmol, 5.4 mg) were stirred in CH_3CN (2 mL) at room temperature for 2 days under blue light (460 nm) in a parallel reactor on N_2 atmosphere. **2av** (15.7 mg, 38%) was obtained through column chromatography (V_{PE}/V_{EA} = 1/1) as a white solid. **^{1}H NMR** (400 MHz, DMSO-*d6*) δ 9.93 (brs, 1H), 9.17 (brs, 1H), 7.72-7.65 (m, 3H), 7.57 (t, *J* = 8.0 Hz, 2H), 7.37 (d, *J* = 8.0 Hz, 2H), 7.19 (d, *J* = 2.4 Hz, 1H), 6.89 (d, *J* = 12.0 Hz, 1H), 6.70-6.66 (m, 3H), 3.75 (s, 3H), 3.68 (s, 2H), 2.27 (s, 3H). **^{13}C NMR** (100 MHz, DMSO-*d6*) δ 168.9, 167.8, 155.5, 153.4, 135.5, 135.4, 132.8, 130.8, 130.7, 130.4, 129.1, 128.9, 121.1, 115.0, 114.4, 114.1, 111.0, 101.9, 55.4, 31.9, 13.3. **IR** (neat) 3316, 1655, 1605, 1514, 1358, 1325, 1225 cm^{-1}. **HRMS** (ESI) Calculated for $C_{25}H_{23}N_2O_4$ $(M+H)^+$: 415.1652; Found 415.1649.

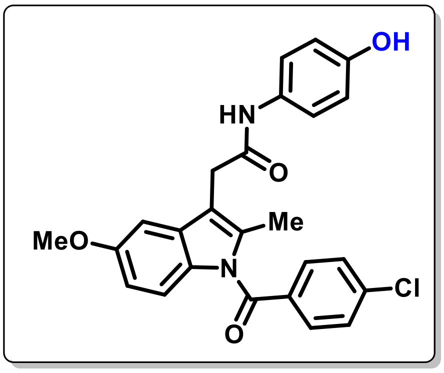

2-(1-(4-chlorobenzoyl)-5-methoxy-2-methyl-1*H*-indol-3-yl)-*N*-(4-hydroxyphenyl)acetamide 2aw: *N*-(4-aminophenyl)-2-(1-(4-chlorobenzoyl)-5-methoxy-2-methyl-1*H*-indol-3-yl)acetamide **1aw** (0.1 mmol, 44.7 mg), $UO_2(NO_3)_2{\cdot}6H_2O$ (4 mol%/0.008 mmol, 2.0 mg), TFA (0.1 mmol, 11.4 mg) and H_2O (0.3 mmol, 5.4 mg) were stirred in CH_3CN (2 mL) at room temperature for 2 days under blue light (460 nm) in a parallel reactor on N_2 atmosphere. **2au** (15.2 mg, 34%) was obtained through column chromatography (V_{PE}/V_{EA} = 1/1) as a white solid. **^{1}H NMR** (500 MHz, DMSO-*d6*) δ 9.94 (brs, 1H), 9.18 (brs, 1H), 7.69 (d, *J* = 9.0 Hz, 2H), 7.64 (d, *J* = 8.5 Hz, 2H), 7.36 (d, *J* = 8.5 Hz, 2H), 7.19 (d, *J* = 2.5 Hz, 1H), 6.93 (d, *J* = 9.0 Hz, 1H), 6.71 (dd, J_1 = 9.0 Hz, J_2 = 2.5 Hz, 1H), 6.68 (d, *J* = 9.0 Hz, 2H), 3.75 (s, 3H), 3.68 (s, 2H), 2.28 (s, 3H). **^{13}C NMR** (125 MHz, DMSO-*d6*) δ 167.9, 167.8, 155.6, 153.4, 137.6, 135.3, 134.2, 131.2, 130.9, 130.8, 130.3, 129.1, 121.1, 115.1, 114.6, 114.3, 111.1, 102.0, 55.5, 31.9, 13.4. **IR** (neat) 3283, 1661, 1518, 1359, 1255, 1216, 1150 cm^{-1}. **HRMS** (ESI) Calculated for $C_{25}H_{22}N_2O_4Cl$ $(M+H)^+$: 449.1263; Found 449.1260.

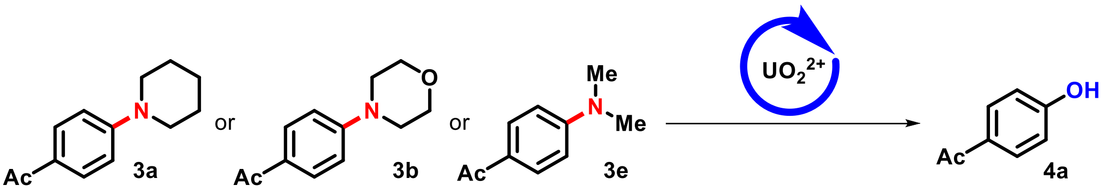

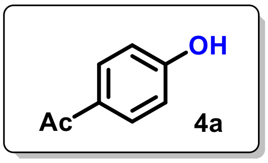

Procedures for 4a from 3a: 1-(4-Hydroxyphenyl)ethan-1-one 4a: 1-(4-(piperidin-1-yl)phenyl)ethan-1-one **3a** (0.2 mmol, 40.6 mg), $UO_2(NO_3)_2{\cdot}6H_2O$ (8 mol%/0.016 mmol, 8.0 mg), TFA (0.4 mmol, 45.6 mg) and H_2O (0.6 mmol, 10.8 mg) were stirred in HFIP (2 mL) at room temperature for 3 days under blue light (460 nm) in a parallel reactor on N_2 atmosphere. **4a** (15.0 mg, 55%)

was obtained through column chromatography (V_{PE}/V_{EA} = 5/1) as a brownish white solid. **^{1}H NMR** (400 MHz, $CDCl_3$) δ 8.14 (brs, 1H), 7.91 (d, *J* = 8.0 Hz, 2H), 6.96 (d, *J* = 8.0 Hz, 2H), 2.59 (s, 3H). **^{13}C NMR** (100 MHz, $CDCl_3$) δ 198.8, 161.5, 131.2, 129.4, 115.6, 26.2. **IR** (neat) 3271, 2918, 2851, 1661, 1358, 1275, 1213 cm^{-1}. GCMS (EI) m/z, $[M]^+$ = 136.[13]

Procedures for 4a from 3b: 1-(4-Morpholinophenyl)ethan-1-one **3b** (0.2 mmol, 41.0 mg), $UO_2(OAc)_2{\cdot}2H_2O$ (4 mol%/0.008 mmol, 3.4 mg), TFA (0.4 mmol, 45.6 mg) and H_2O (0.6 mmol, 10.8 mg) were stirred in HFIP (2 mL) at room temperature for 3 days under blue light (460 nm) in a parallel reactor on N_2 atmosphere. **4a** (11.2 mg, 41%) was obtained through column chromatography (V_{PE}/V_{EA} = 5/1) as a brownish white solid.

Procedures for 4a from 3e: 1-(4-(Dimethylamino)phenyl)ethan-1-one **3c** (0.2 mmol, 32.7 mg), $UO_2(NO_3)_2{\cdot}6H_2O$ (4 mol%/0.008 mmol, 4.0 mg), TFA (0.2 mmol, 22.8 mg) and H_2O (0.6 mmol, 10.8 mg) were stirred in HFIP (2 mL) at room temperature for 3 days under blue light (460 nm) in a parallel reactor on N_2 atmosphere. **4a** (14.4 mg, 53%) was obtained through column chromatography (V_{PE}/V_{EA} = 5/1) as a brownish white solid.

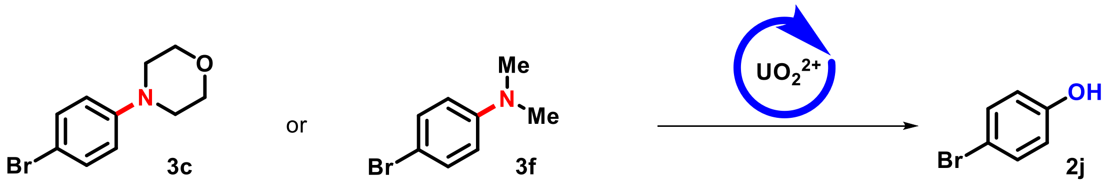

Procedures for 2j from 3c: 4-(4-Bromophenyl)morpholine **3c** (0.2 mmol, 48.2 mg), $UO_2(NO_3)_2{\cdot}6H_2O$ (8 mol%/0.016 mmol, 8.0 mg), TFA (0.2 mmol, 22.8 mg) and H_2O (0.6 mmol, 10.8 mg) were stirred in HFIP (2 mL) at room temperature for 2.5 days under blue light (460 nm) in a parallel reactor in the air. **2j** (20.7 mg, 60%) was obtained through column chromatography (V_{PE}/V_{EA} = 10/1) as a brown solid.

Procedures for 2j from 3f: 4-Bromo-N,N-dimethylaniline **3f** (0.2 mmol, 39.8 mg), $UO_2(NO_3)_2 \cdot 6H_2O$ (4 mol%/0.008 mmol, 4.0 mg), TFA (0.2 mmol, 22.8 mg) and H_2O (0.6 mmol, 10.8 mg) were stirred in HFIP (2 mL) at room temperature for 2 days under blue light (460 nm) in a parallel reactor in the air. **2j** (25.8 mg, 75%) was obtained through column chromatography (V_{PE}/V_{EA} = 10/1) as a brown solid.

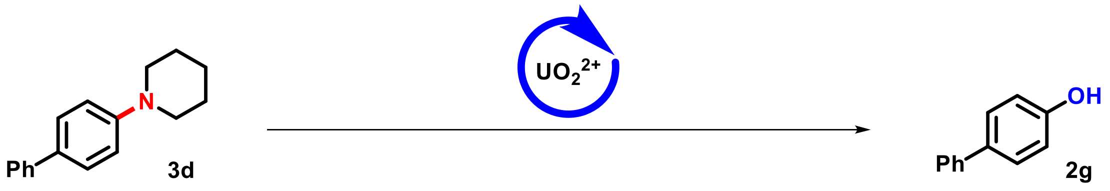

Procedures for 2g from 3d: 1-([1,1'-biphenyl]-4-yl)piperidine **3d** (0.2 mmol, 47.5 mg), $UO_2(NO_3)_2 \cdot 6H_2O$ (8 mol%/0.016 mmol, 8.0 mg), TFA (0.4 mmol, 45.6 mg) and H_2O (0.6 mmol, 10.8 mg) were stirred in HFIP (2 mL) at room temperature for 3 days under blue Llight (460 nm) in a parallel reactor on N_2 atmosphere. **2g** (24.2 mg, 71%) was obtained through column chromatography (V_{PE}/V_{EA} = 10/1) as a white solid.

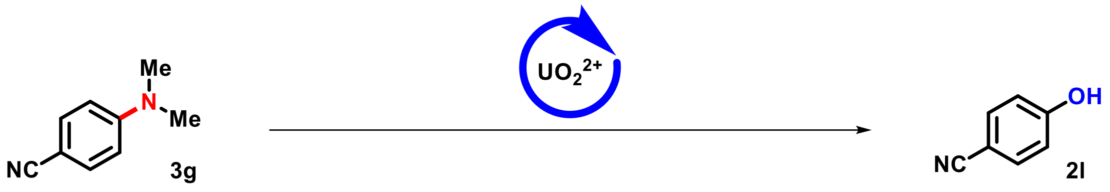

Procedures for 2l from 3g: 4-(Dimethylamino)benzonitrile **3g** (0.2 mmol, 29.2 mg), $UO_2(OAc)_2 \cdot 2H_2O$ (8 mol%/0.016 mmol, 6.8 mg), TFA (0.6 mmol, 68.4 mg) and H_2O (0.6 mmol, 10.8 mg) were stirred in HFIP (2 mL) at room temperature for 4 days under blue light (460 nm) in a parallel reactor in the air. **2l** (14.3 mg, 60%) was obtained through column chromatography (V_{PE}/V_{EA} = 10/1) as a white solid.

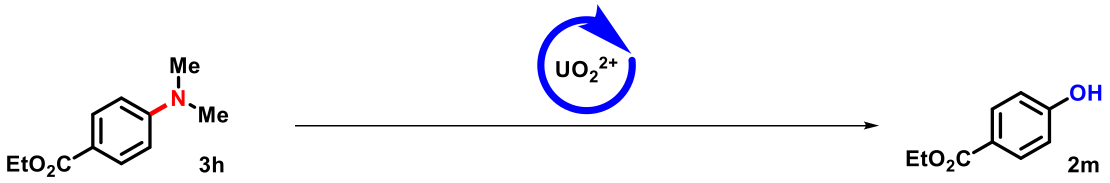

Procedures for 2m from 3h: Ethyl 4-(dimethylamino)benzoate **3h** (0.2 mmol, 38.6 mg), $UO_2(NO_3)_2 \cdot 6H_2O$ (4 mol%/0.016 mmol, 4.0 mg), TFA (0.2 mmol, 22.8 mg) and H_2O (0.6 mmol, 10.8 mg) were stirred in HFIP (2 mL) at room temperature for 4 days under blue light (460 nm) in a parallel reactor in the air. **2m** (15.6 mg, 47%) was obtained through column chromatography (V_{PE}/V_{EA} = 4/1) as a white solid.

VIII. X-ray Crystallography Analysis of Compounds

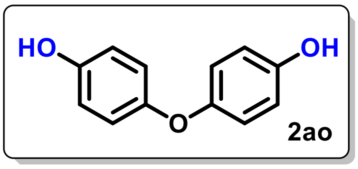

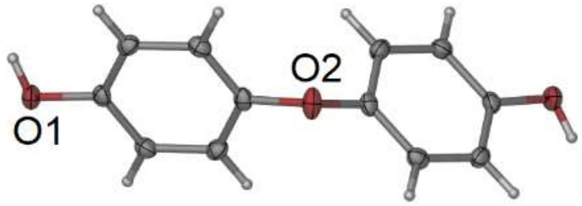

Table S1. Crystal Data and Structure Refinement for Complex **2ao** **(CCDC2043527)**

Complex	**2ao**
Empirical formula	$C_{12}H_{10}O_3$
Formula weight	202.20
Crystal system	monoclinic
Space group	Pbcn
a, Å	5.27850(10)
b, Å	8.2544(2)
c, Å	22.0985(5)
α, °	90
β, °	90
γ, °	90
V, $Å^3$	962.85(4)
Z	4
ρ_{calcd} (g cm^{-3})	1.395
μ/mm^{-1}	0.830
F(000)	424.0
2θ range for data collection/°	8.002 to 134.118
R_{int}	0.0338
Final *R* indices [$I > 2\sigma(I)$]	R_1 = 0.0301 wR_2 = 0.0793
Final *R* indexes [all data]	R_1 = 0.0319, wR_2 = 0.0808

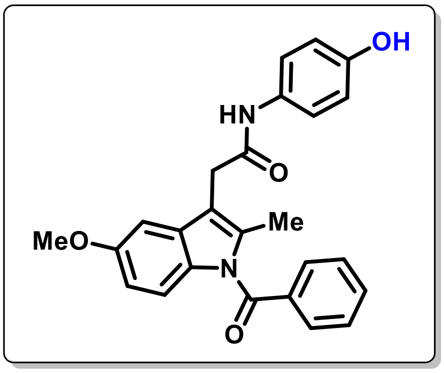

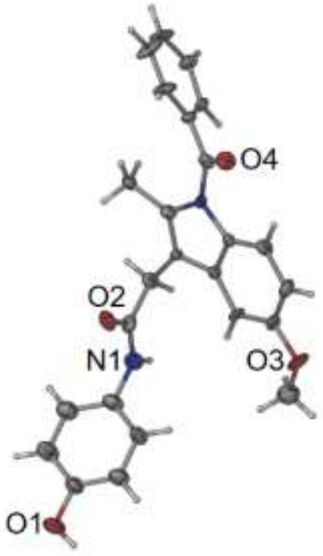

Table S2. Crystal Data and Structure Refinement for Complex **2av** **(CCDC2050763)**

Complex	**2av**
Empirical formula	$C_{25}H_{22}N_2O_4$
Formula weight	414.44
Crystal system	monoclinic
Space group	$P2_1$
a, Å	9.4784(3)
b, Å	10.4081(3)
c, Å	21.5648(7)
α, °	90
β, °	93.555(3)
γ, °	90
V, Å^3	2123.32(11)
Z	4
ρ_{calc} (g cm^{-3})	1.296
μ/mm^{-1}	0.720
F(000)	872.0
2θ range for data collection/°	8.216 to 134.072
R_{int}	0.0567
Final *R* indices [$I > 2\sigma(I)$]	R_1 = 0.1226 wR_2 = 0.3235
Final *R* indexes [all data]	R_1 = 0.1298, wR_2 = 0.3296

IX. NMR Spectra

^{1}H NMR of 2a

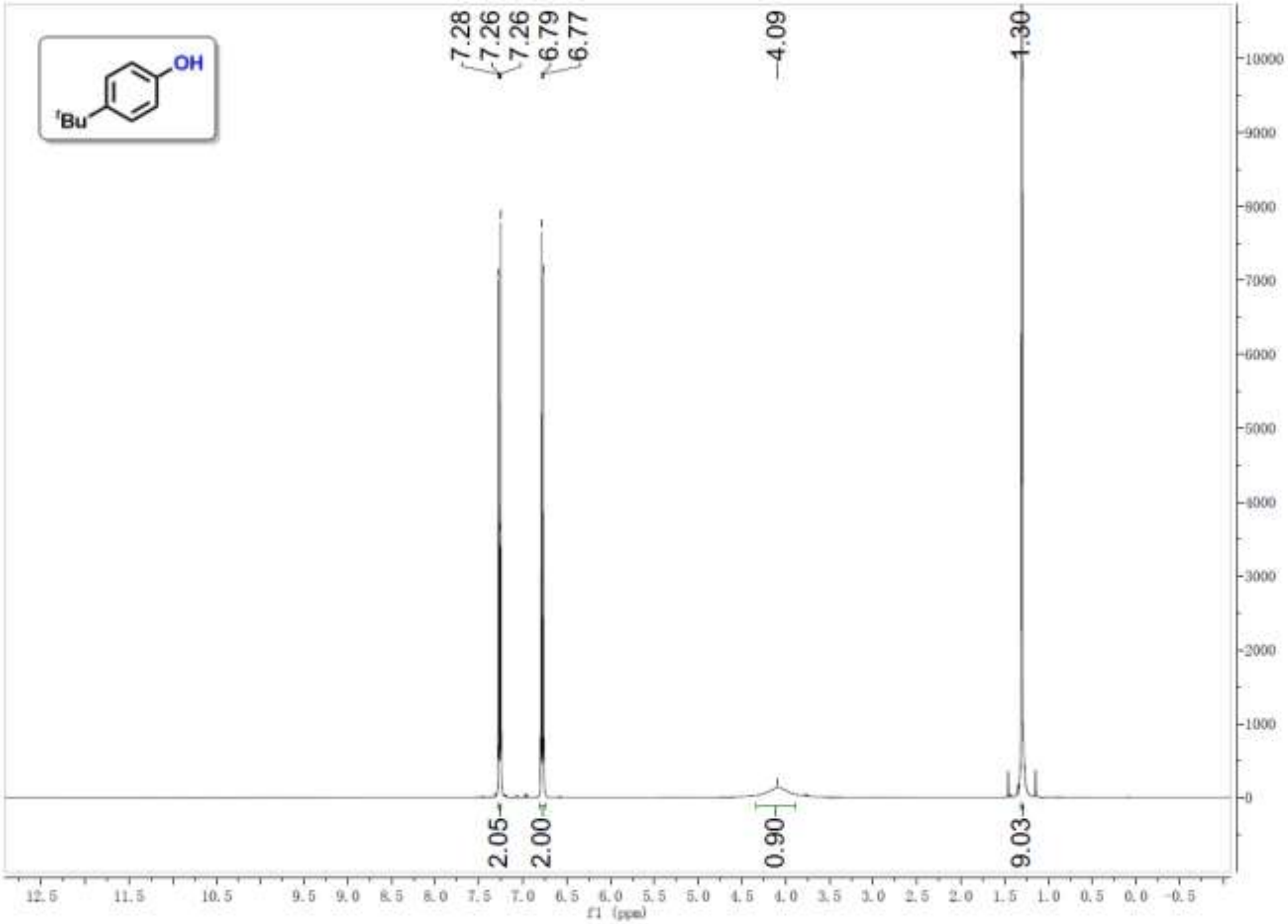

^{13}C NMR of 2a

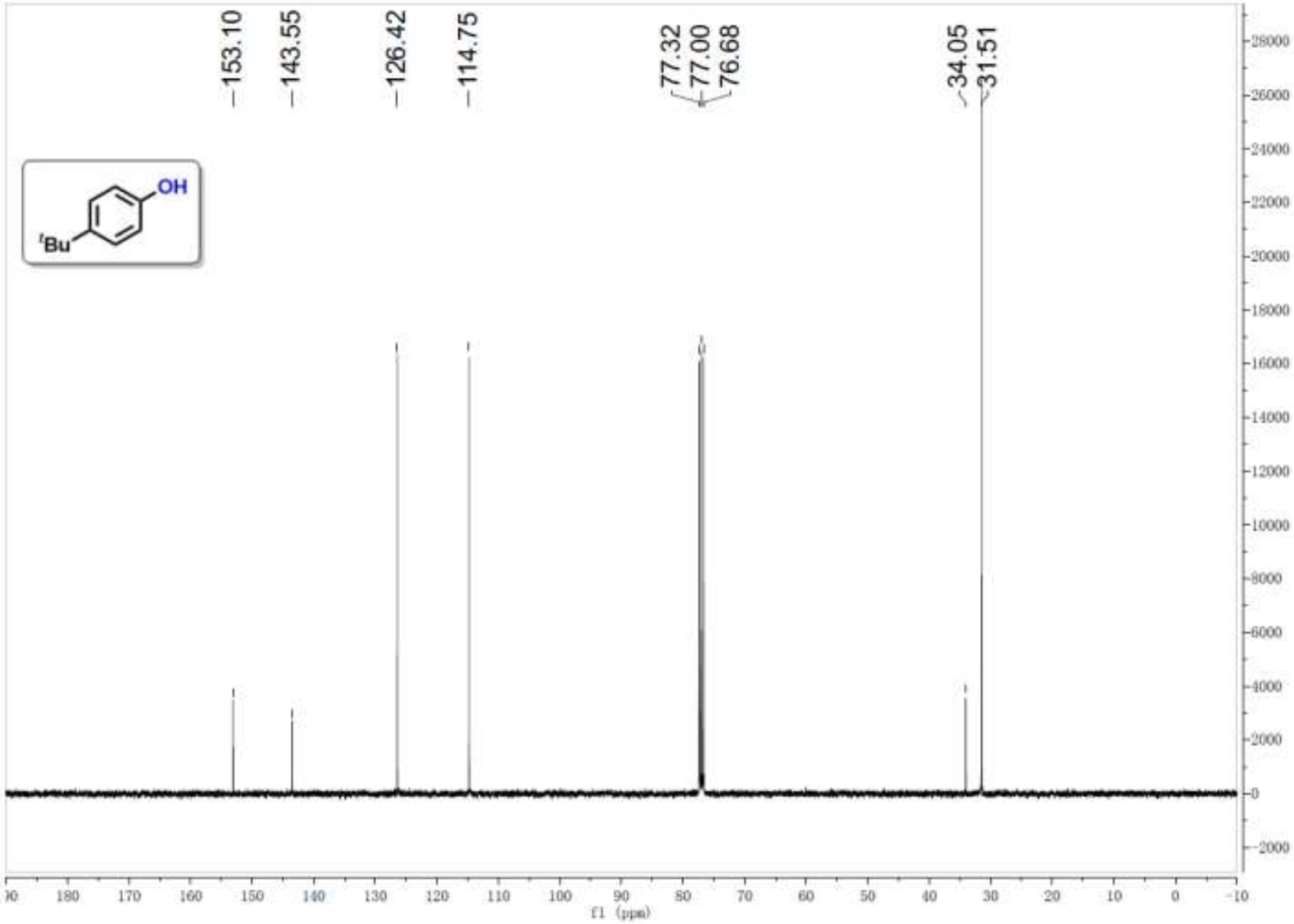

153.10
143.55
126.42
114.75
77.32
77.00
76.68
34.05
31.51
OH
tBu
f1 (ppm)

^{1}H NMR of 2b

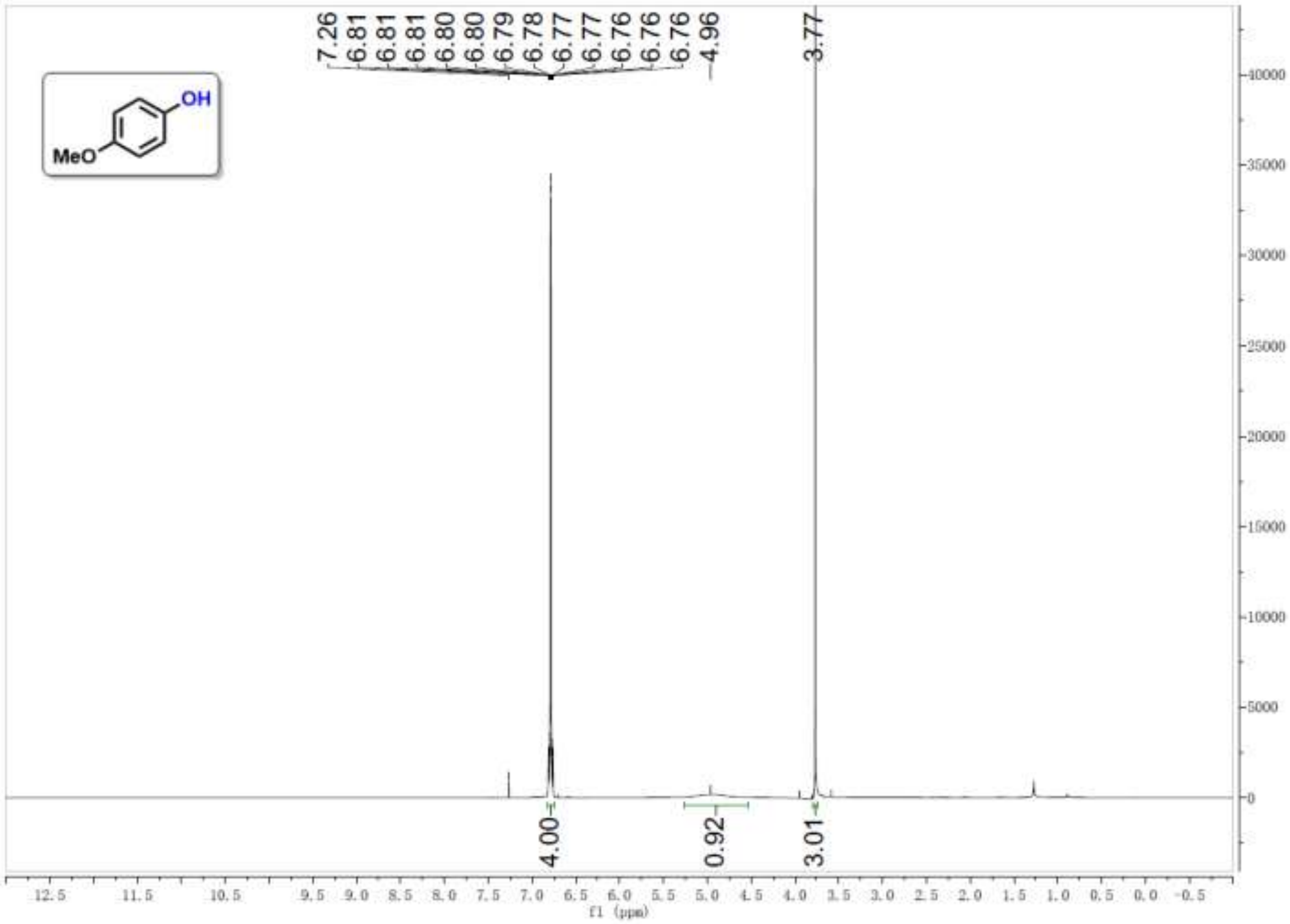

OH
MeO
7.26
6.81
6.81
6.81
6.80
6.80
6.79
6.78
6.77
6.77
6.77
6.76
6.76
6.76
4.96
3.77
4.00
0.92
3.01
f1 (ppm)

^{13}C NMR of 2b

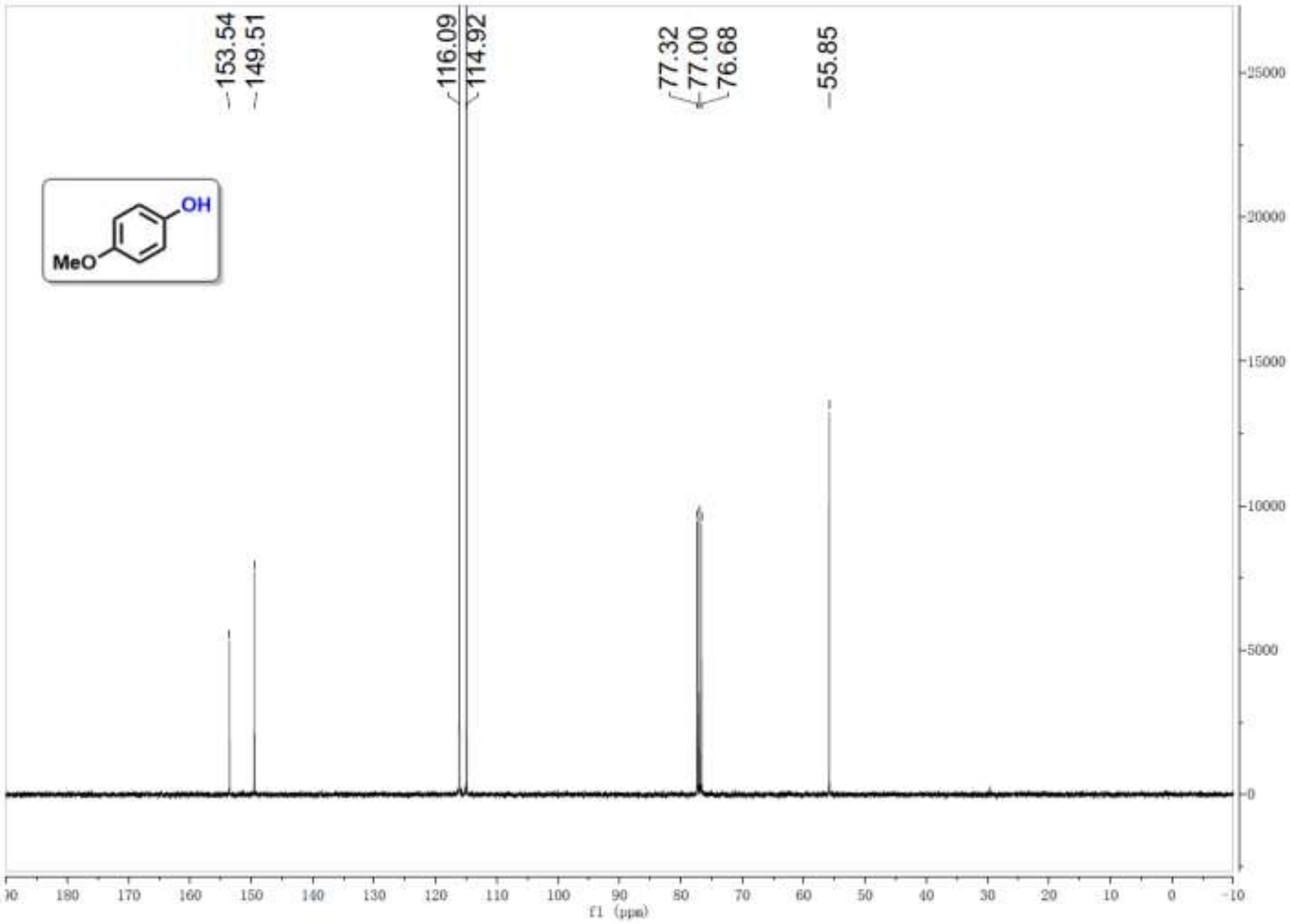

153.54
149.51
116.09
114.92
77.32
77.00
76.68
55.85
OH
MeO
25000
20000
15000
10000
5000
0
f1 (ppm)

^{1}H NMR of 2c

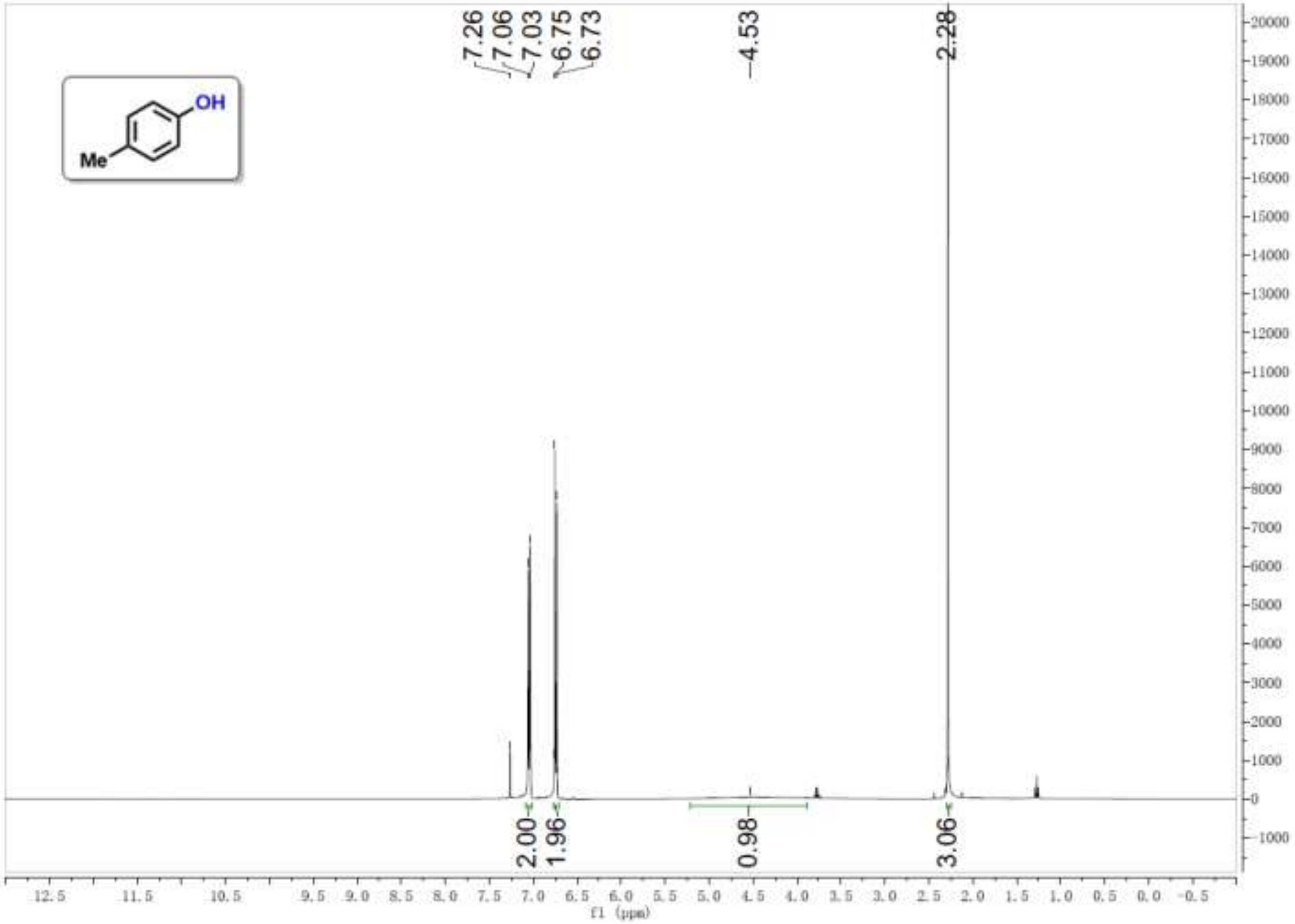

^{13}C NMR of 2c

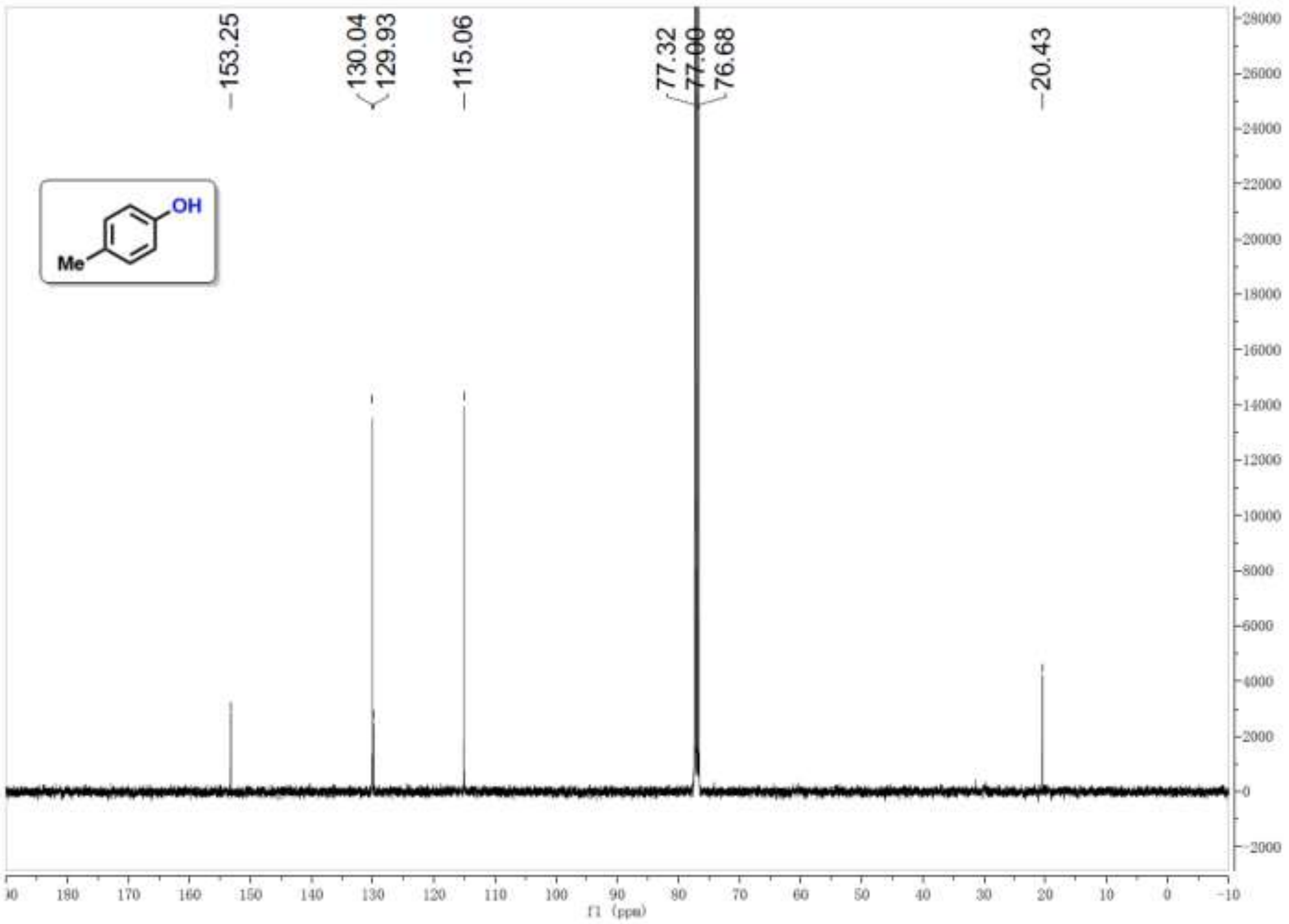

^{1}H NMR of 2d

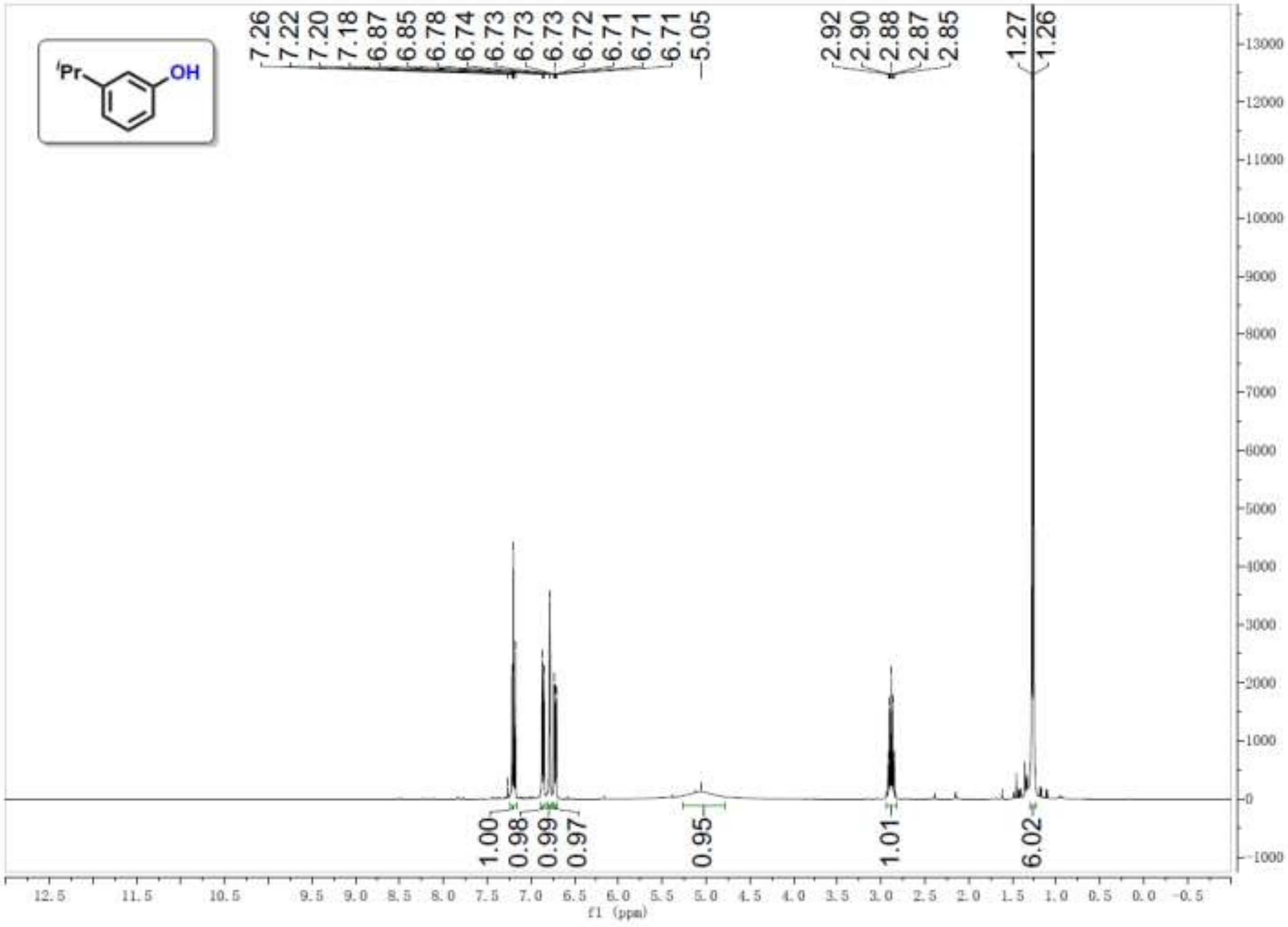

iPr
OH
7.26
7.22
7.20
7.18
6.87
6.85
6.78
6.74
6.73
6.73
6.73
6.72
6.71
6.71
6.71
5.05
2.92
2.90
2.88
2.87
2.85
1.27
1.26
1.00
0.98
0.99
0.97
0.95
1.01
6.02
f1 (ppm)

^{13}C NMR of 2d

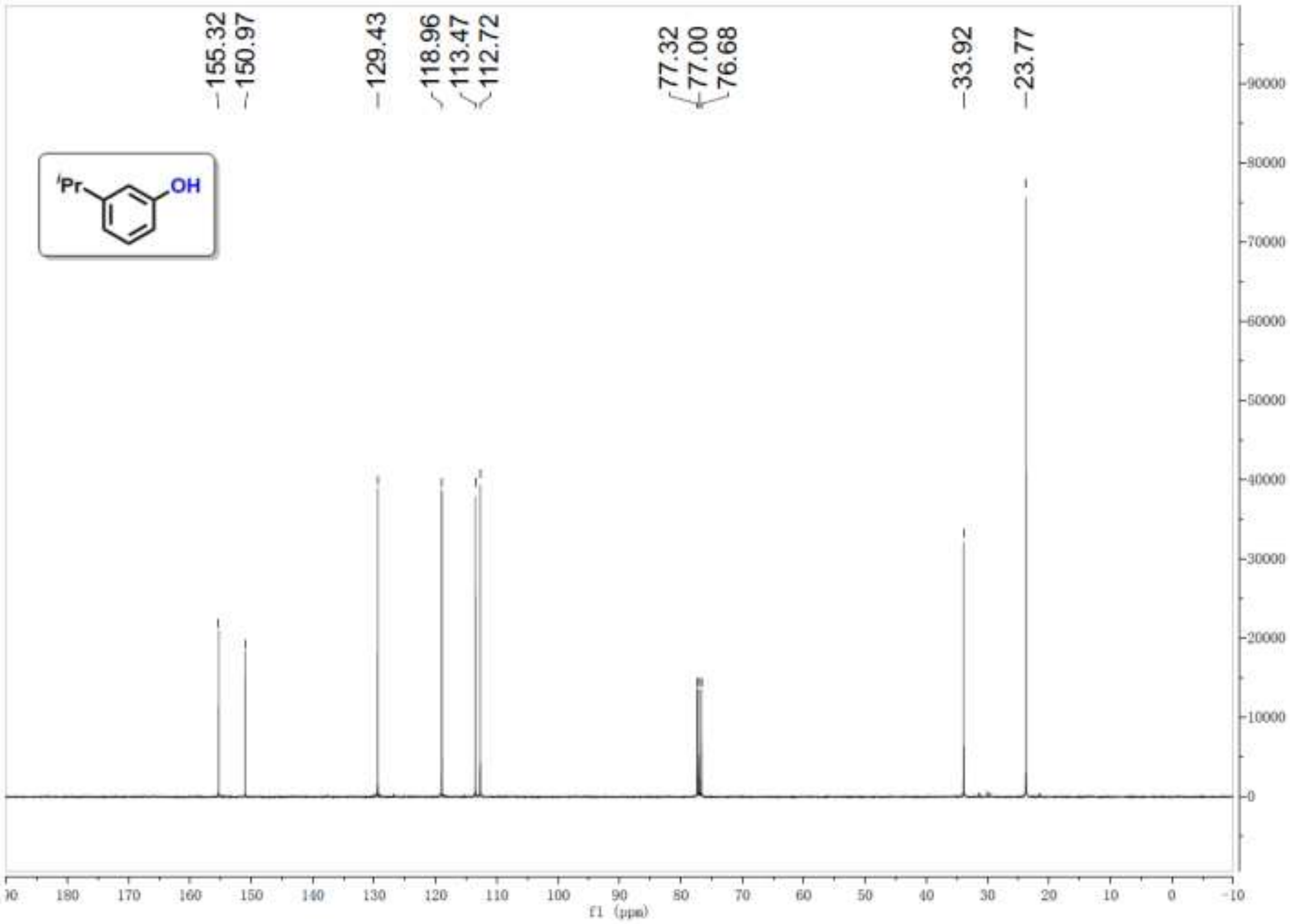

155.32
150.97
129.43
118.96
113.47
112.72
77.32
77.00
76.68
33.92
23.77
ⁱPr
OH
f1 (ppm)

^{1}H NMR of 2e

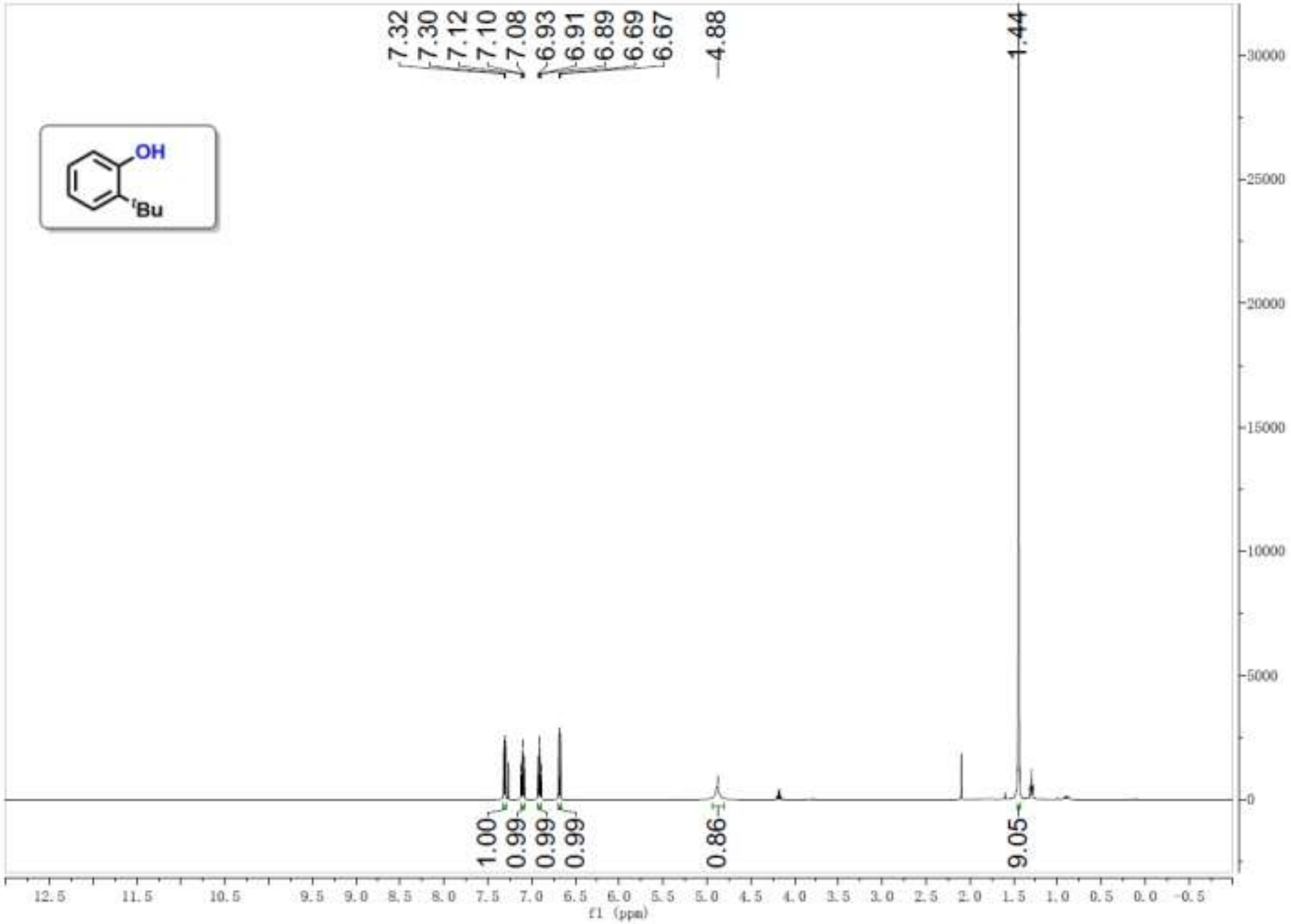

OH
tBu
7.32
7.30
7.12
7.10
7.08
6.93
6.91
6.89
6.69
6.67
4.88
1.44
1.00
0.99
0.99
0.99
0.86
9.05
30000
25000
20000
15000
10000
5000
0
12.5
11.5
10.5
9.5
9.0
8.5
8.0
7.5
7.0
6.5
6.0
5.5
5.0
4.5
4.0
3.5
3.0
2.5
2.0
1.5
1.0
0.5
0.0
-0.5
f1 (ppm)

^{13}C NMR of 2e

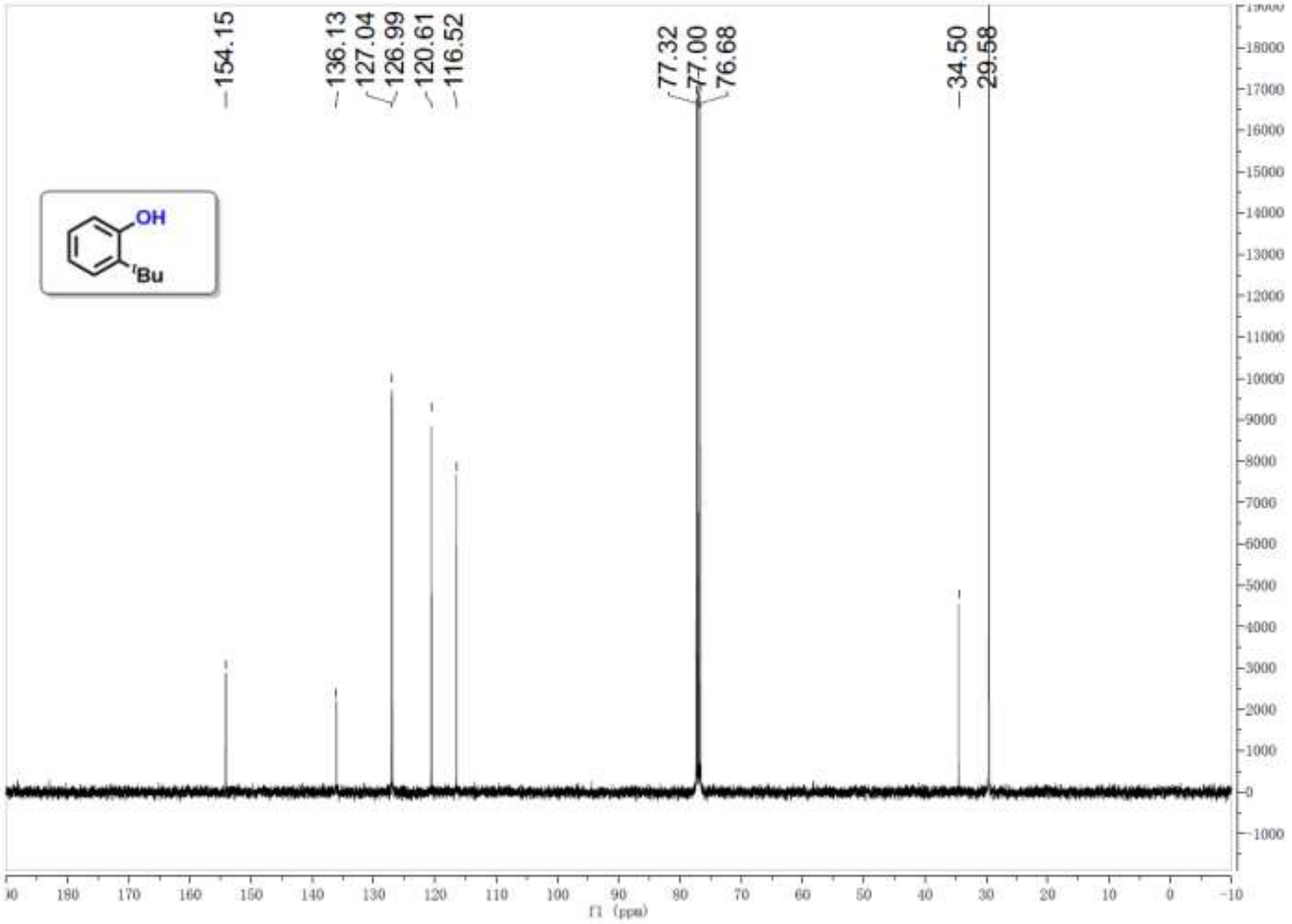

154.15
136.13
127.04
126.99
120.61
116.52
77.32
77.00
76.68
34.50
29.58
OH
ᵗBu
f1 (ppm)

^{1}H NMR of 2f

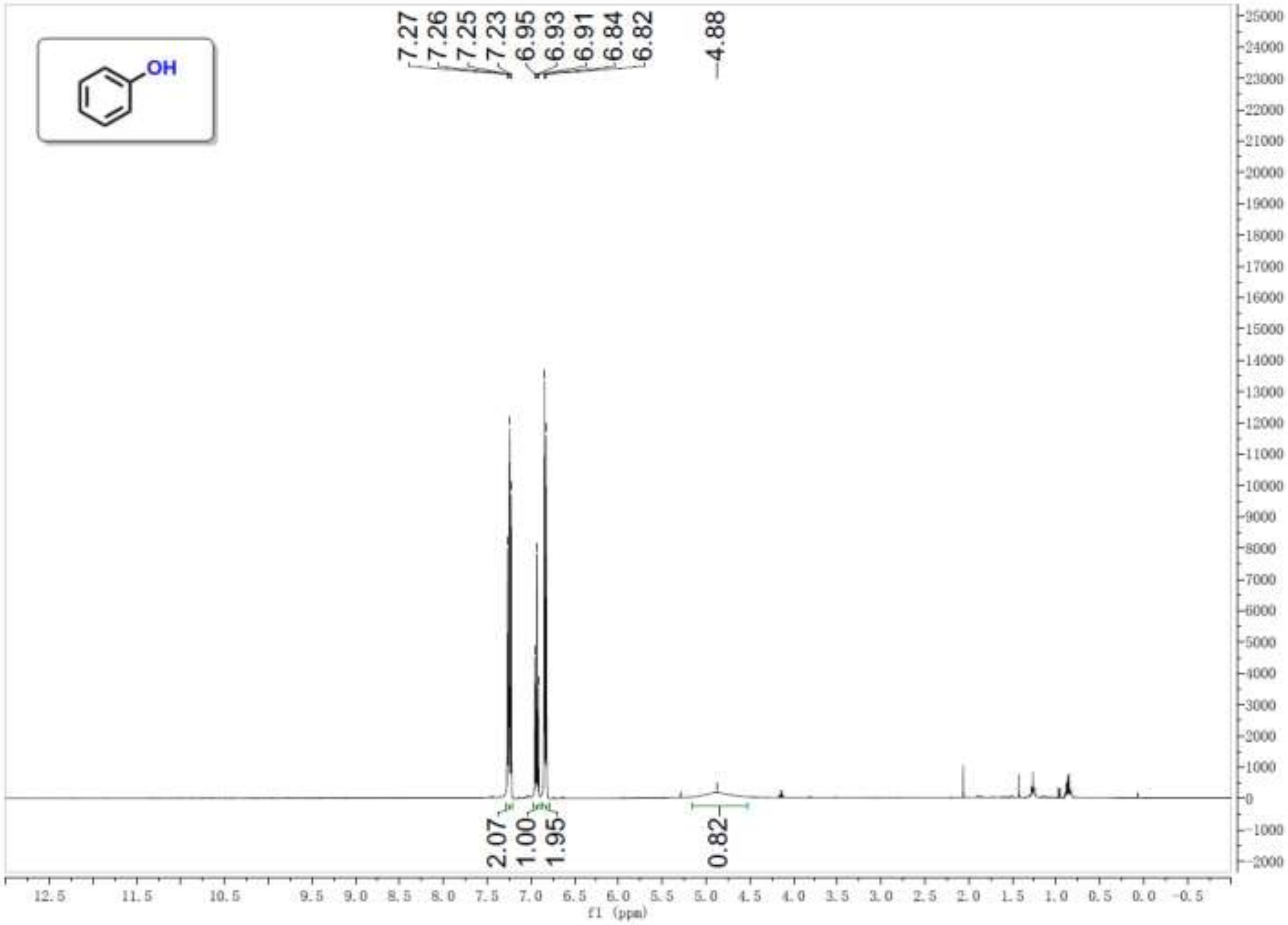

^{13}C NMR of 2f

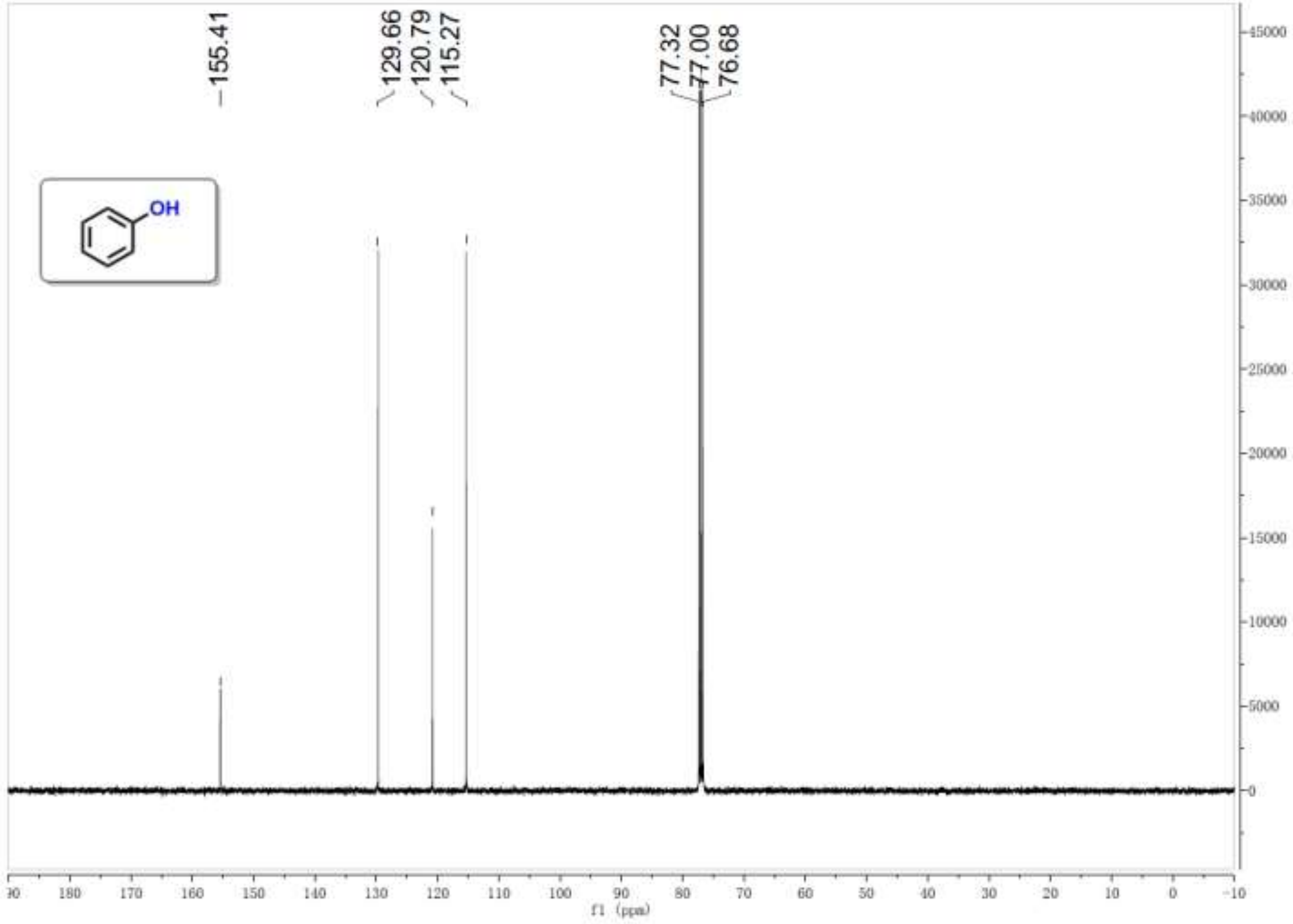

^{1}H NMR of 2g

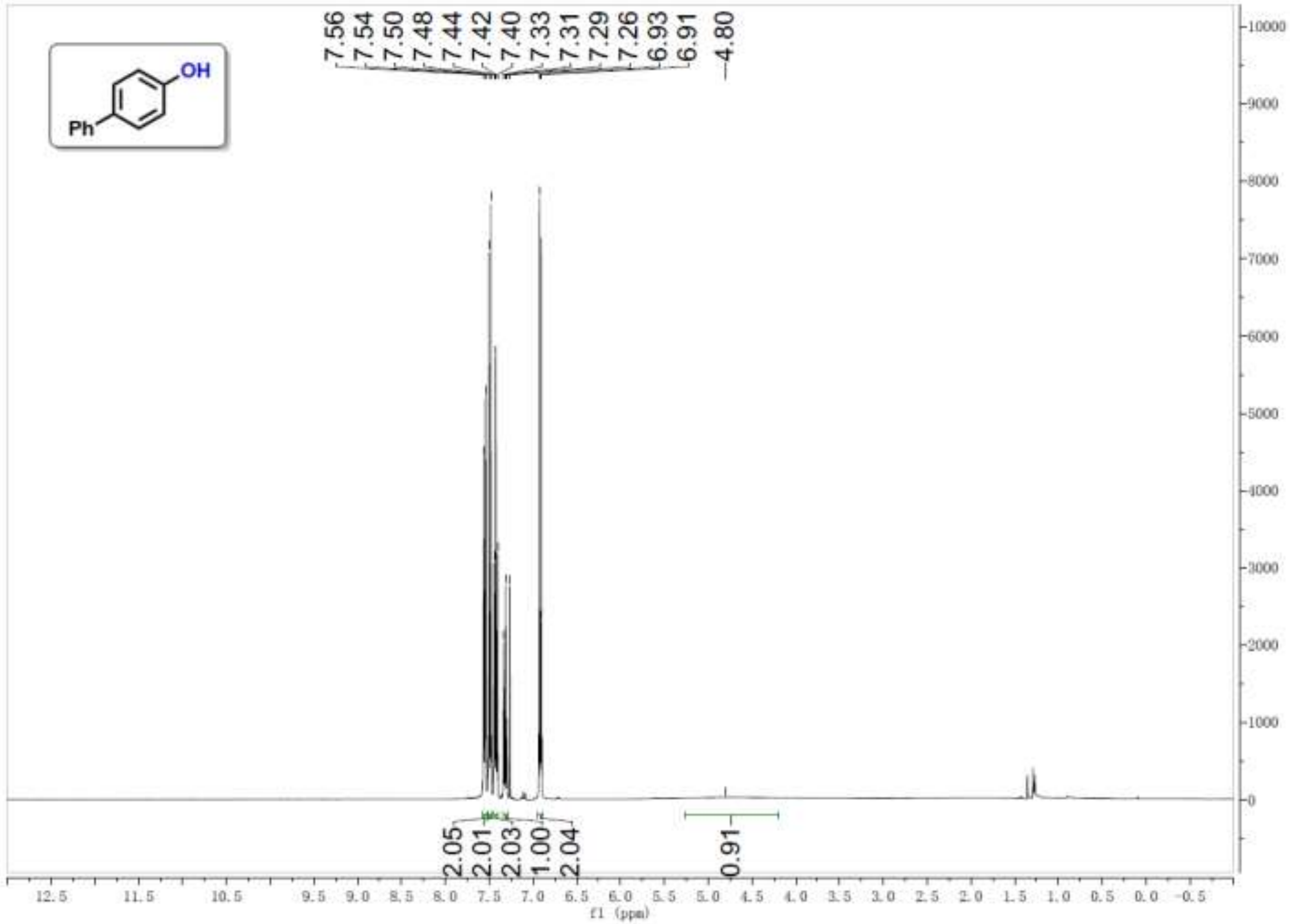

OH
Ph
7.56
7.54
7.50
7.48
7.44
7.42
7.40
7.33
7.31
7.29
7.26
6.93
6.91
4.80
2.05
2.01
2.03
1.00
2.04
0.91
f1 (ppm)

^{13}C NMR of 2g

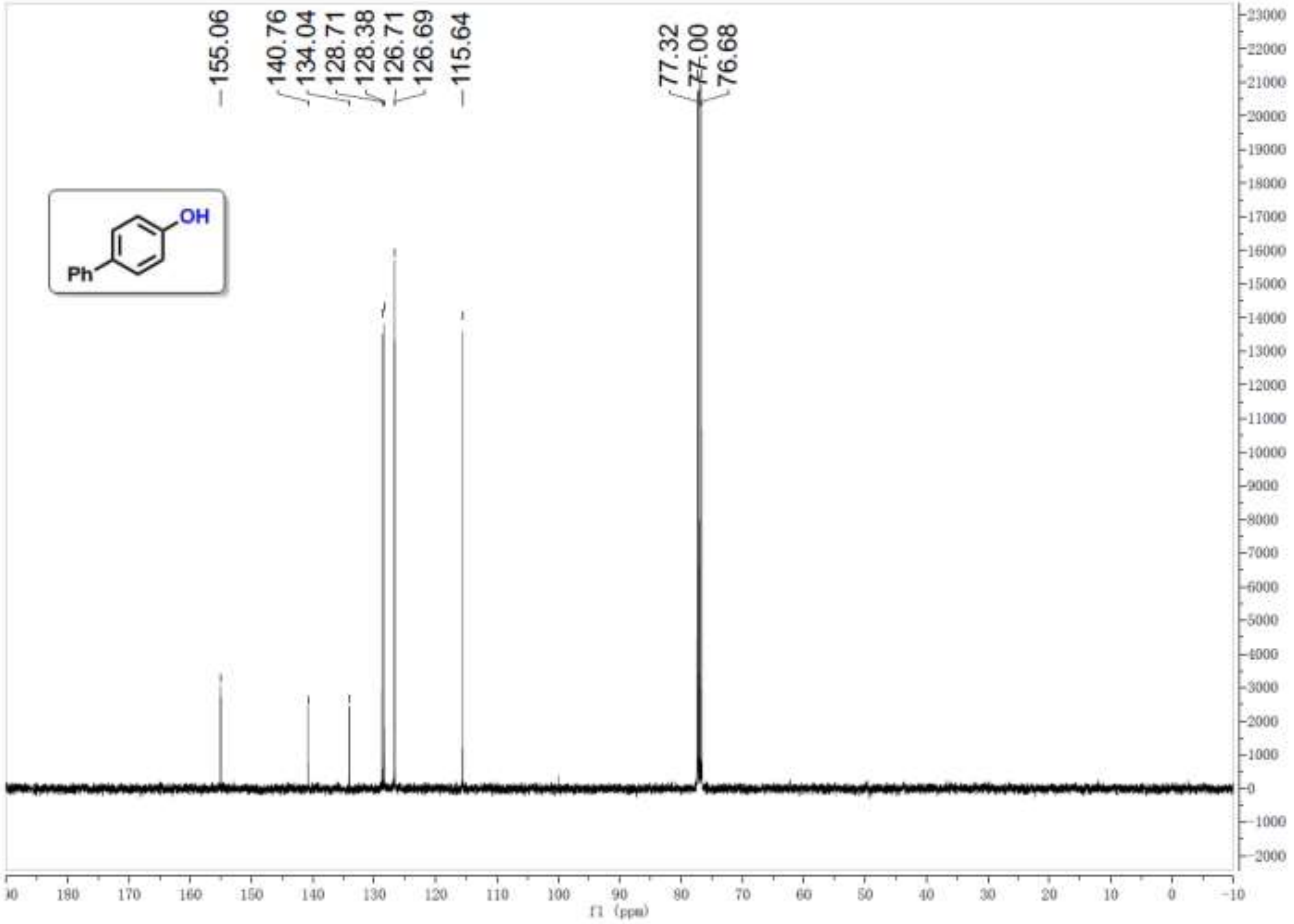
155.06
140.76
134.04
128.71
128.38
126.71
126.69
115.64
77.32
77.00
76.68
OH
Ph
f1 (ppm)

^{1}H NMR of 2h

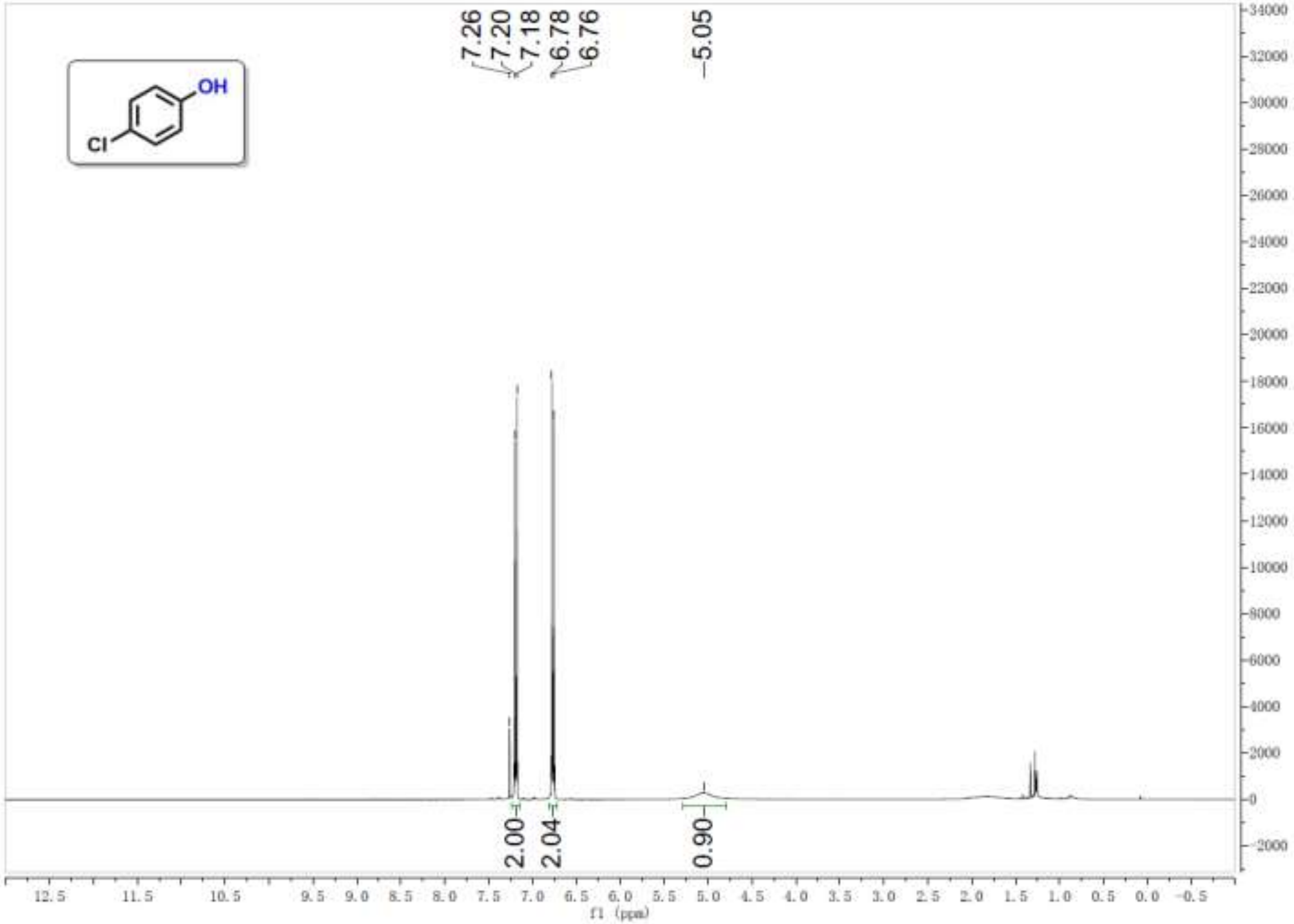

^{13}C NMR of 2h

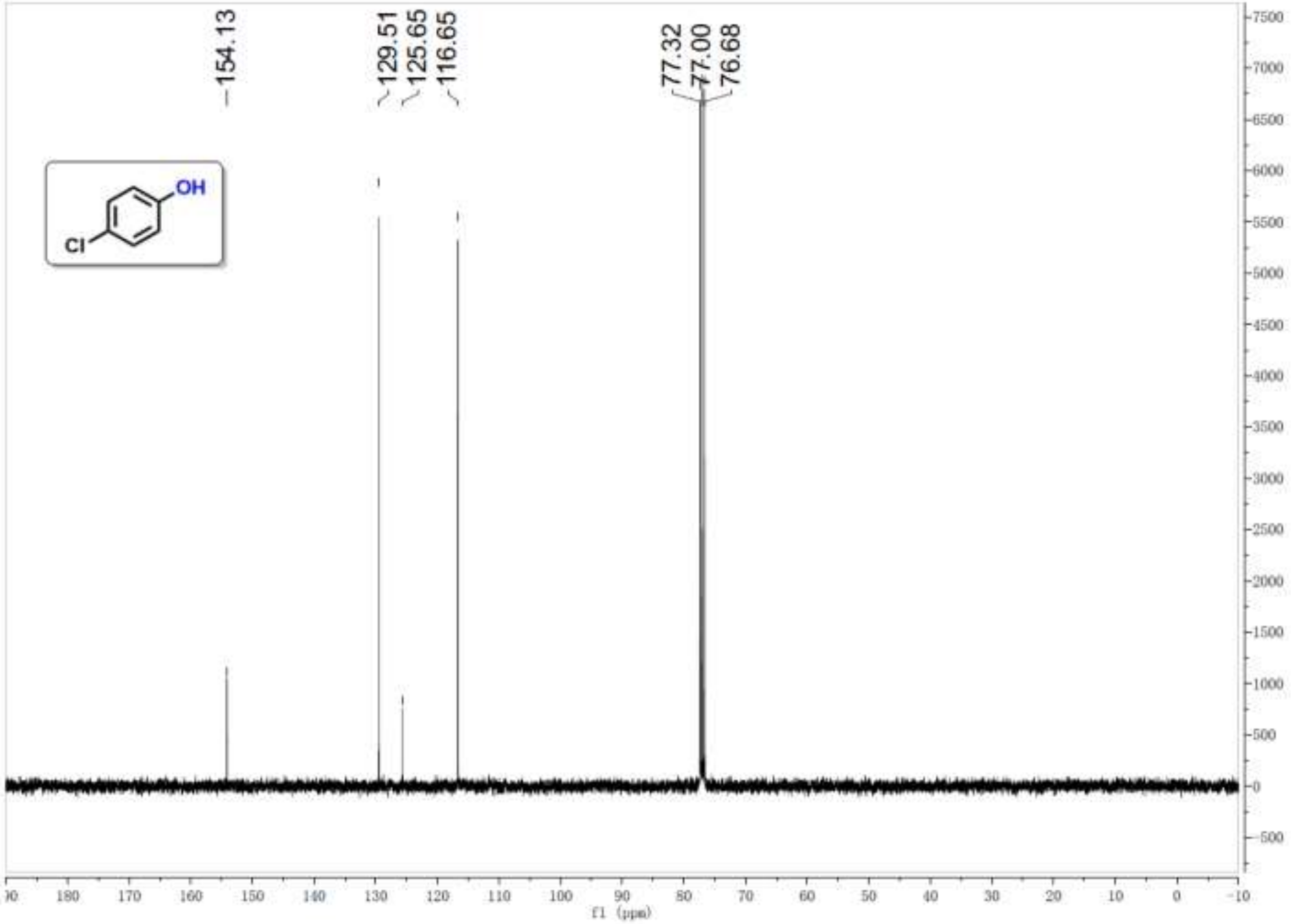

^{1}H NMR of 2i

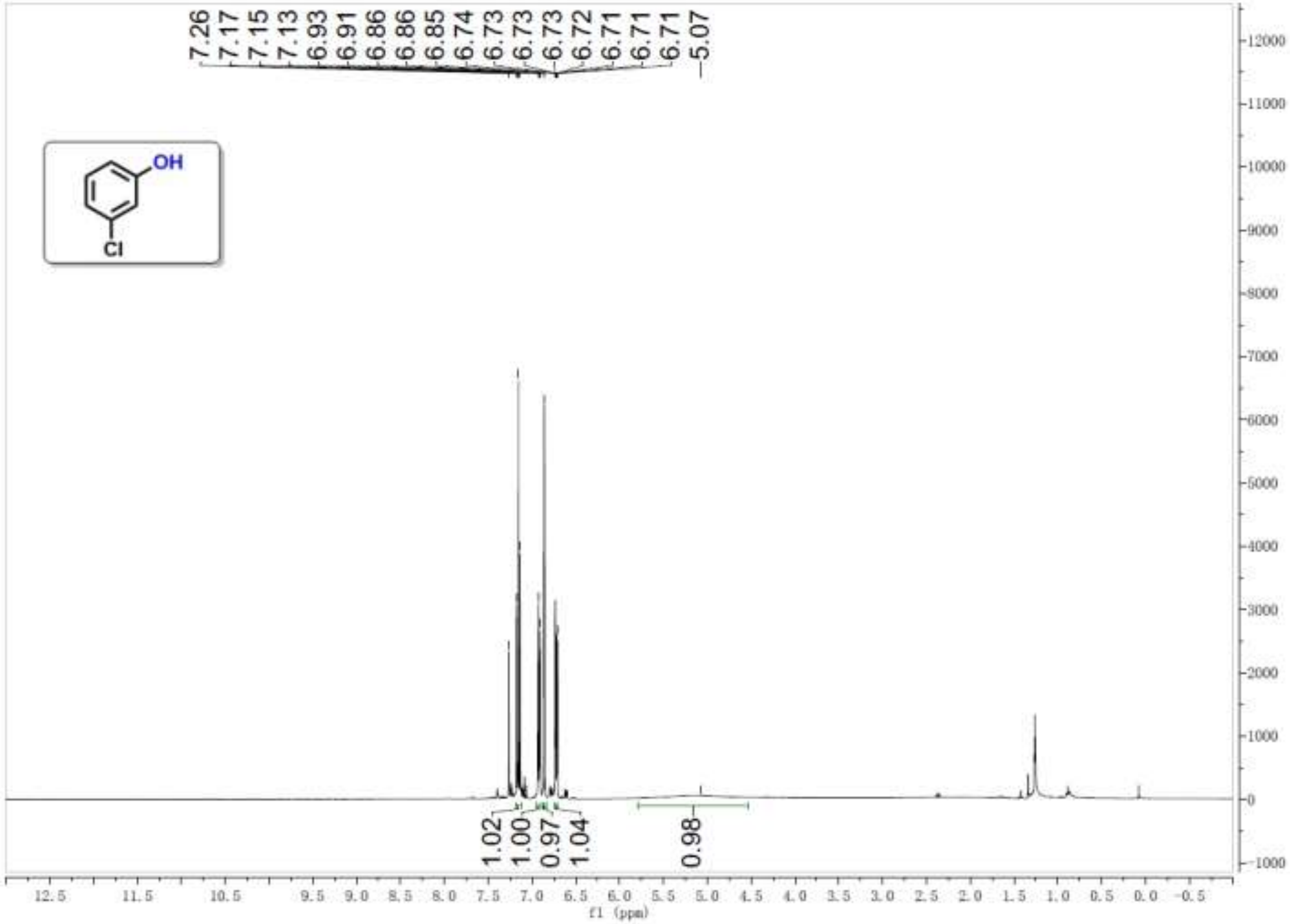

7.26
7.17
7.15
7.13
6.93
6.91
6.86
6.86
6.85
6.74
6.73
6.73
6.73
6.72
6.71
6.71
6.71
6.71
5.07
OH
Cl
1.02
1.00
0.97
1.04
0.98
f1 (ppm)

^{13}C NMR of 2i

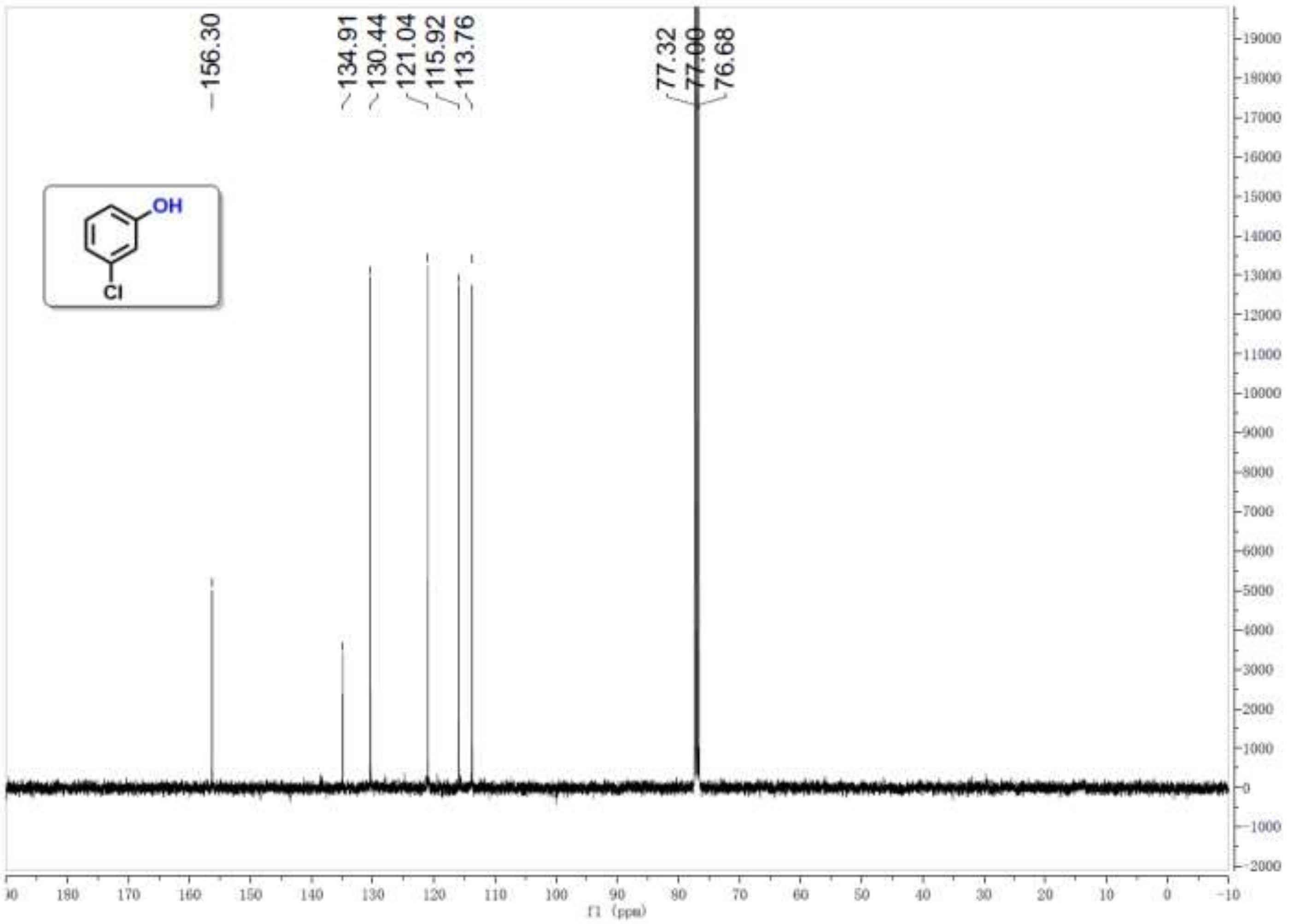
156.30
134.91
130.44
121.04
115.92
113.76
77.32
77.00
76.68
OH
Cl
f1 (ppm)

^{1}H NMR of 2j

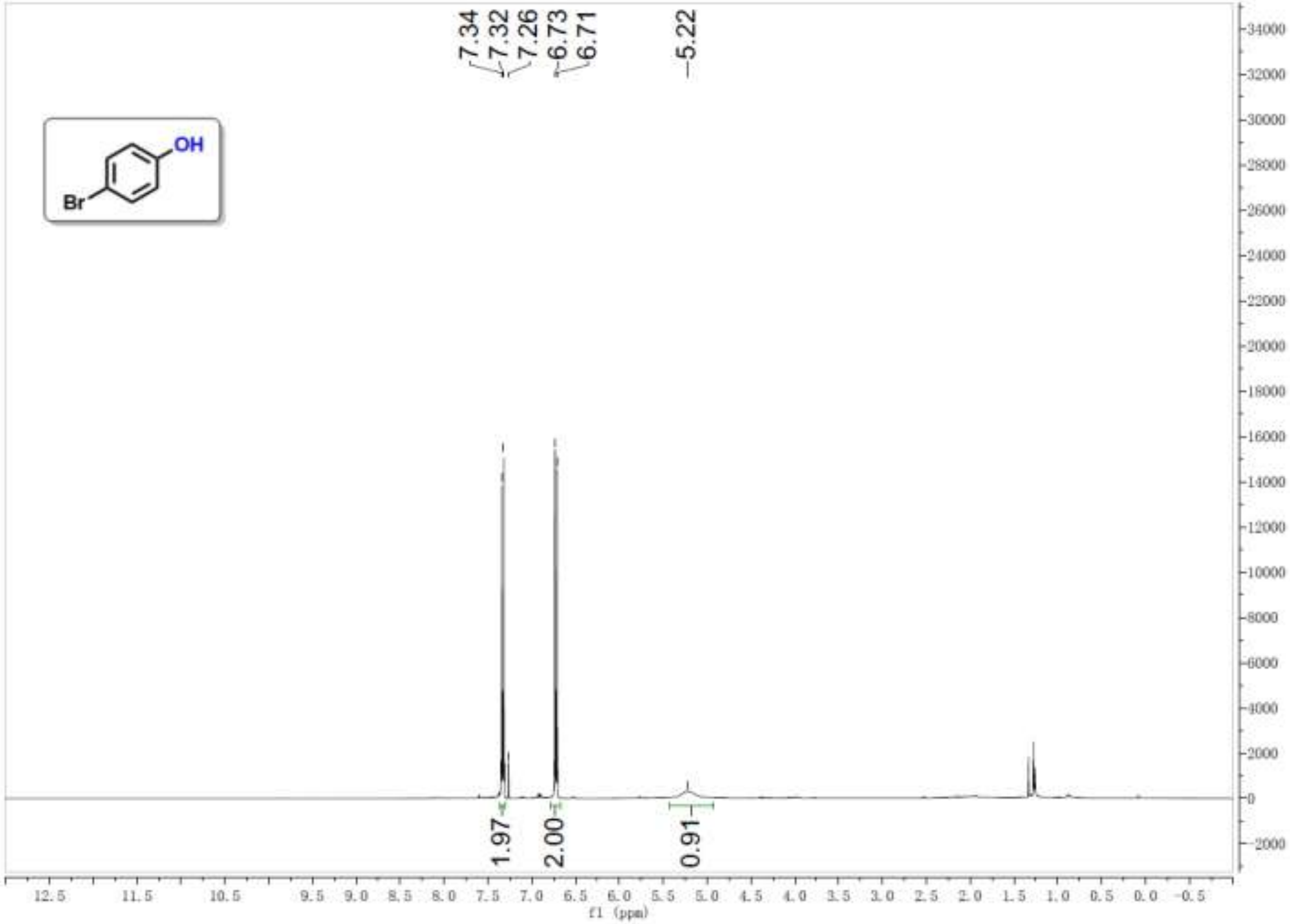

^{13}C NMR of 2j

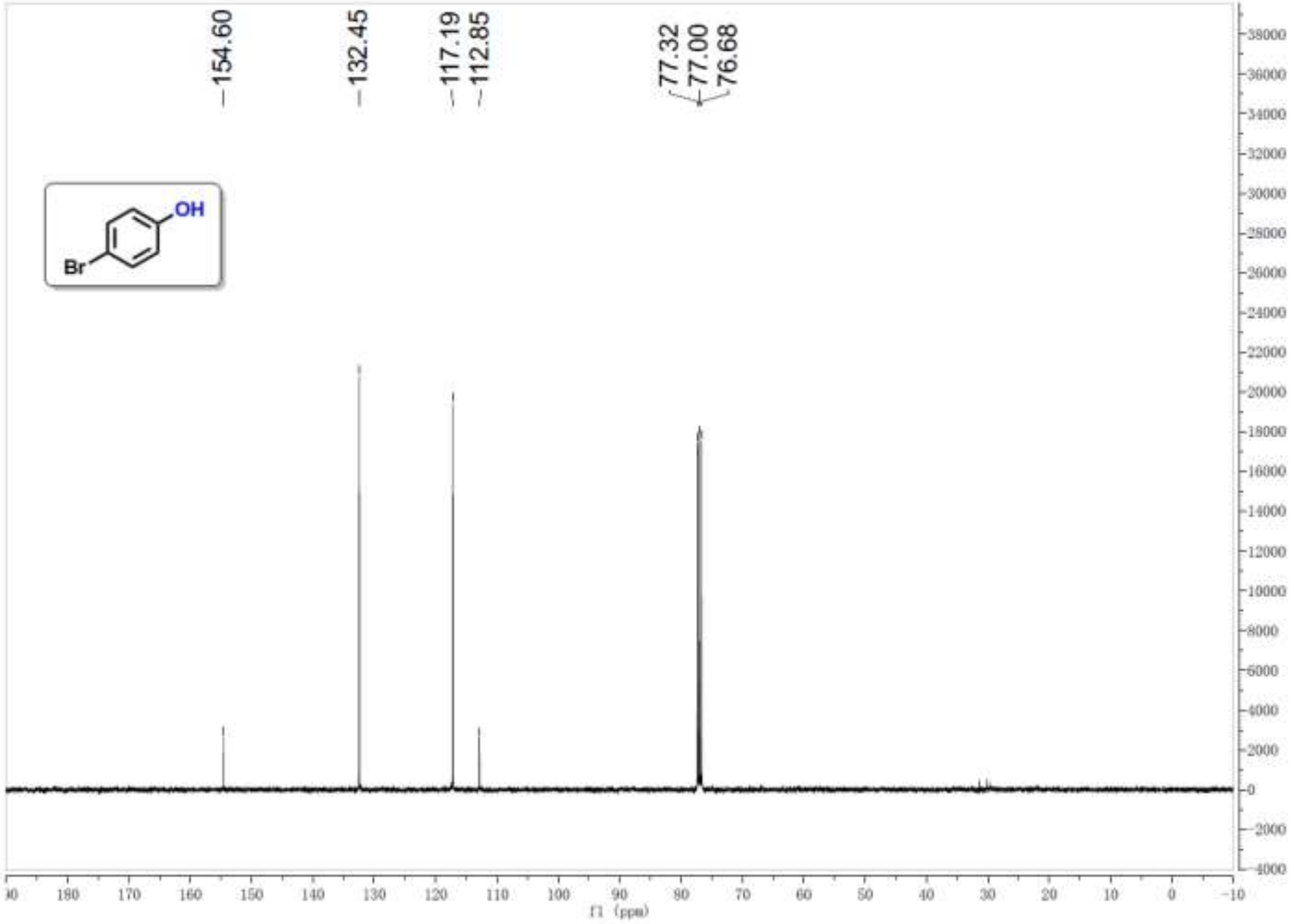

^{1}H NMR of 2k

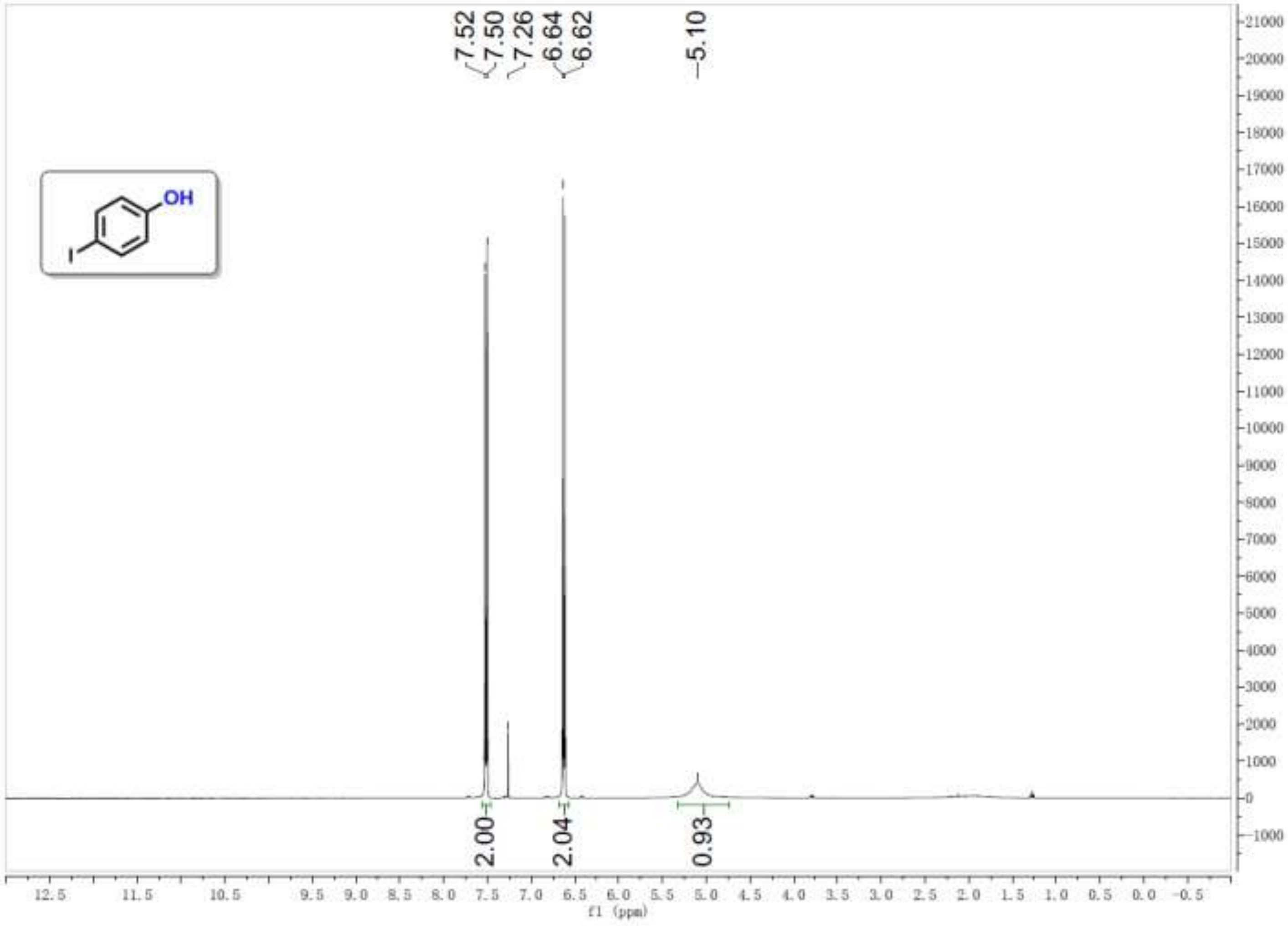

^{13}C NMR of 2k

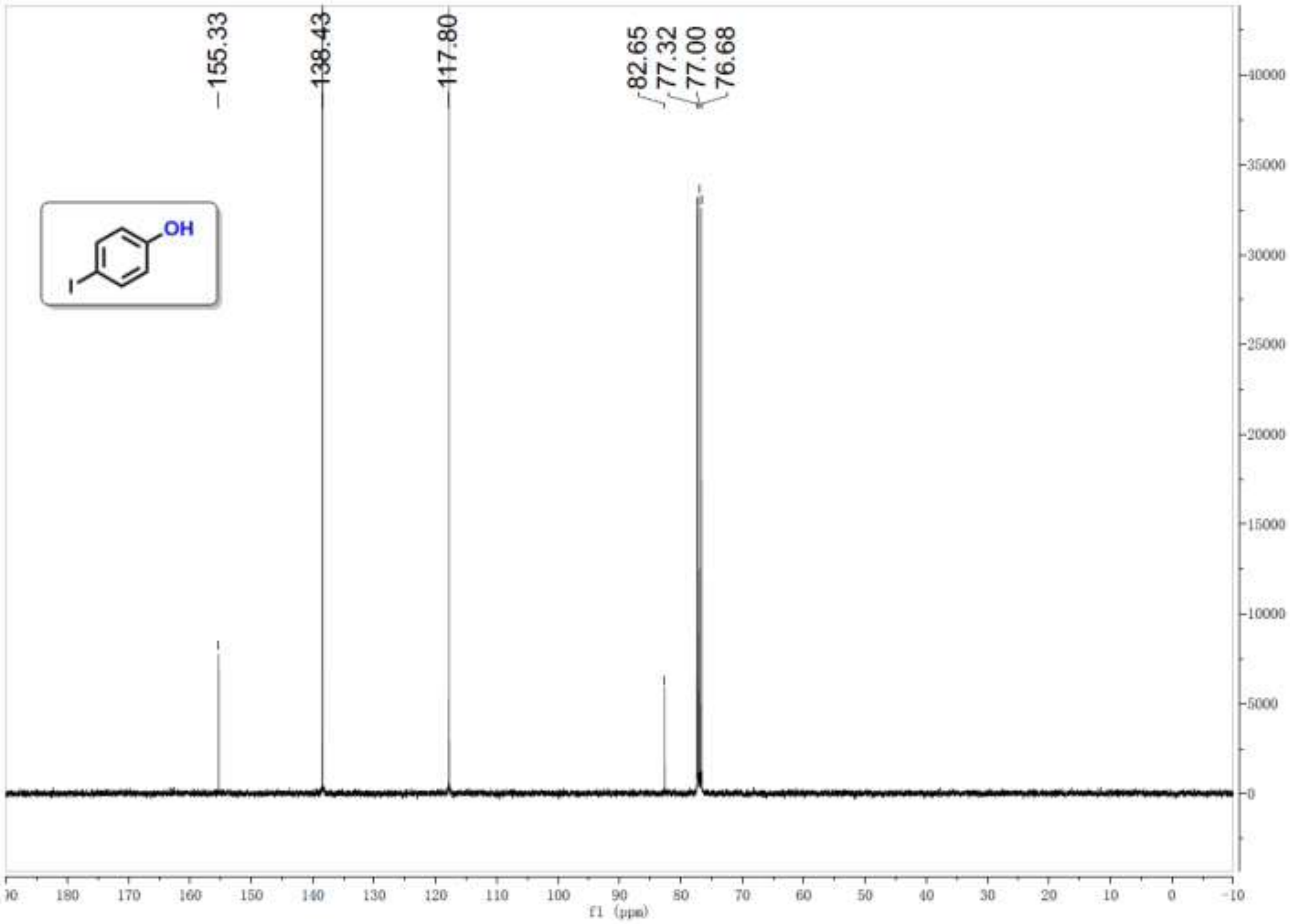

155.33
138.43
117.80
82.65
77.32
77.00
76.68
OH
I
40000
35000
30000
25000
20000
15000
10000
5000
0
180
170
160
150
140
130
120
110
100
90
80
70
60
50
40
30
20
10
0
-10
f1 (ppm)

^{1}H NMR of 2l

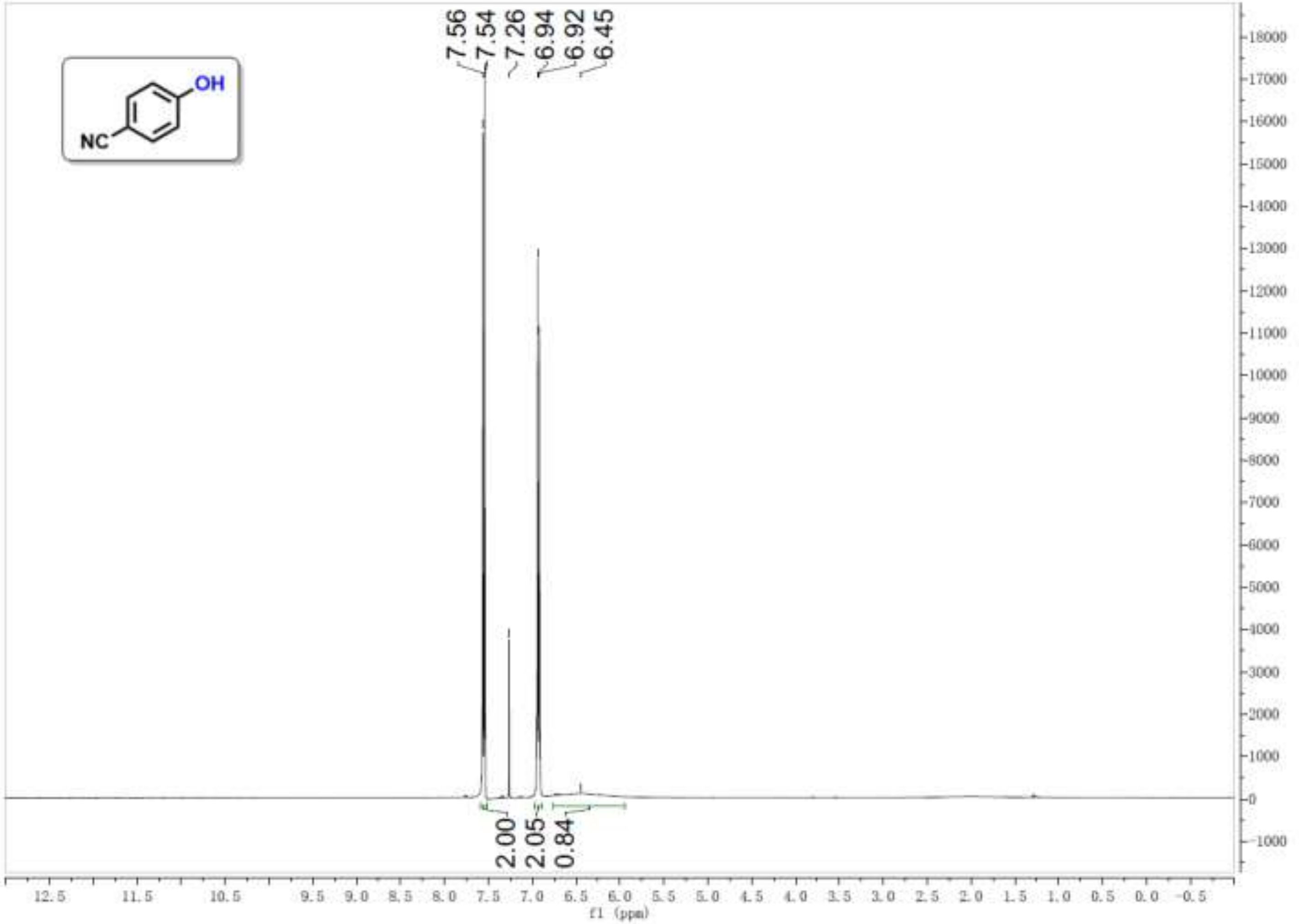
OH
NC
7.56
7.54
7.26
6.94
6.92
6.45
2.00
2.05
0.84
18000
17000
16000
15000
14000
13000
12000
11000
10000
9000
8000
7000
6000
5000
4000
3000
2000
1000
0
-1000
12.5
11.5
10.5
9.5
9.0
8.5
8.0
7.5
7.0
6.5
6.0
5.5
5.0
4.5
4.0
3.5
3.0
2.5
2.0
1.5
1.0
0.5
0.0
-0.5
f1 (ppm)

^{13}C NMR of 2l

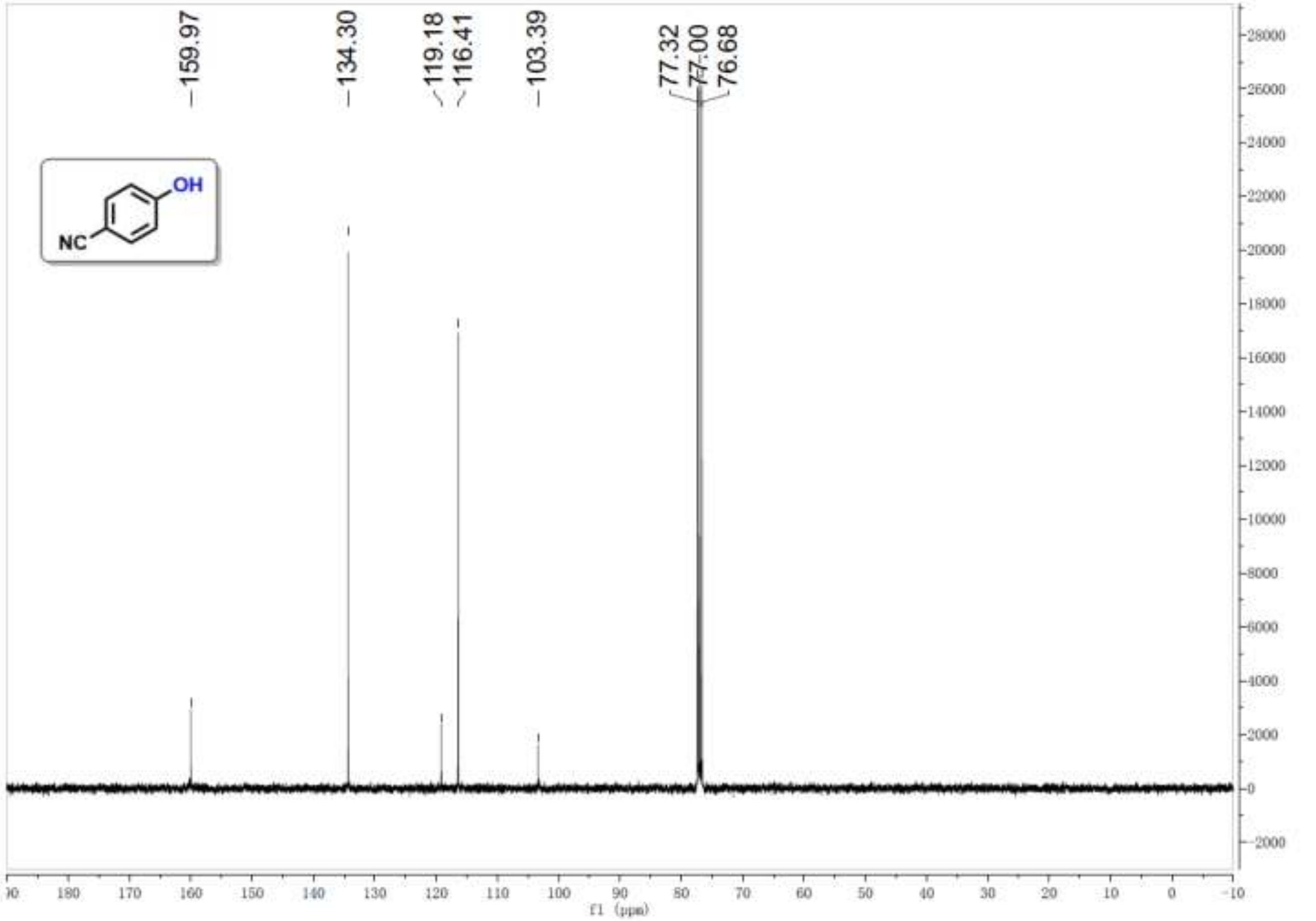

^{1}H NMR of 2m

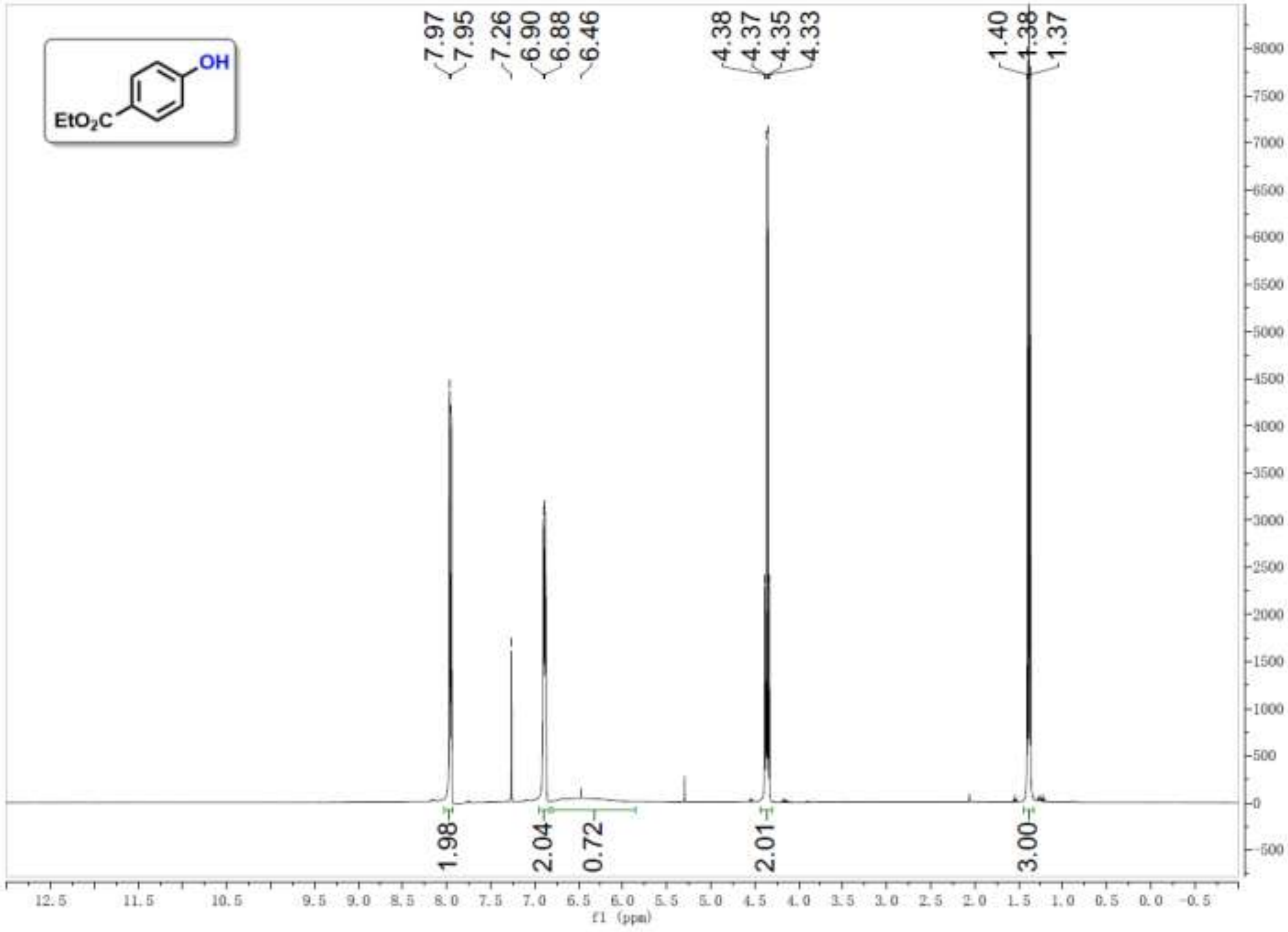

OH
EtO_2C
7.97
7.95
7.26
6.90
6.88
6.46
4.38
4.37
4.35
4.33
1.40
1.38
1.37
1.98
2.04
0.72
2.01
3.00
f1 (ppm)

^{13}C NMR of 2m

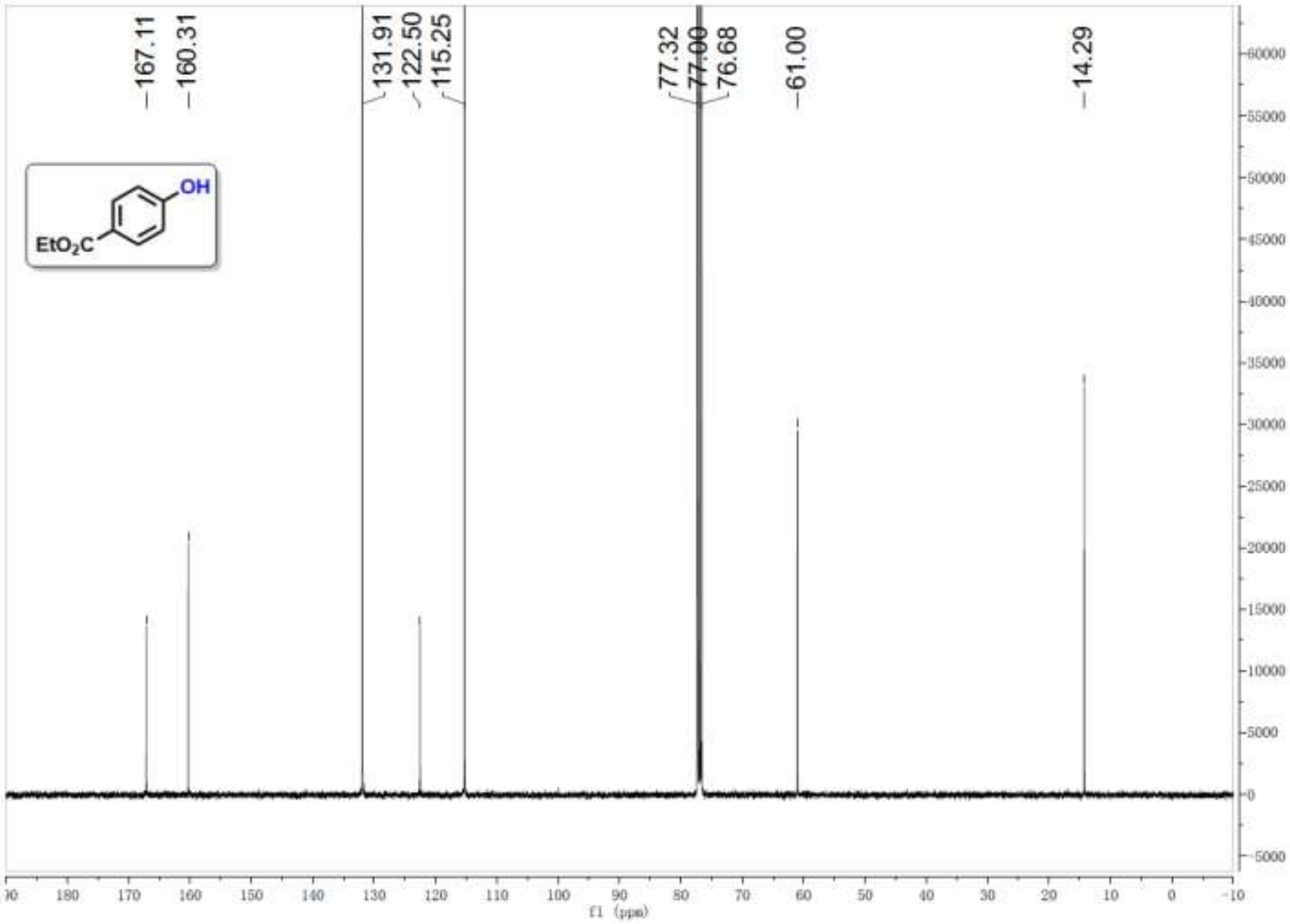
167.11
160.31
131.91
122.50
115.25
77.32
77.00
76.68
61.00
14.29
OH
EtO2C
60000
55000
50000
45000
40000
35000
30000
25000
20000
15000
10000
5000
0
-5000
180
170
160
150
140
130
120
110
100
90
80
70
60
50
40
30
20
10
0
-10
f1 (ppm)

^{1}H NMR of 2n

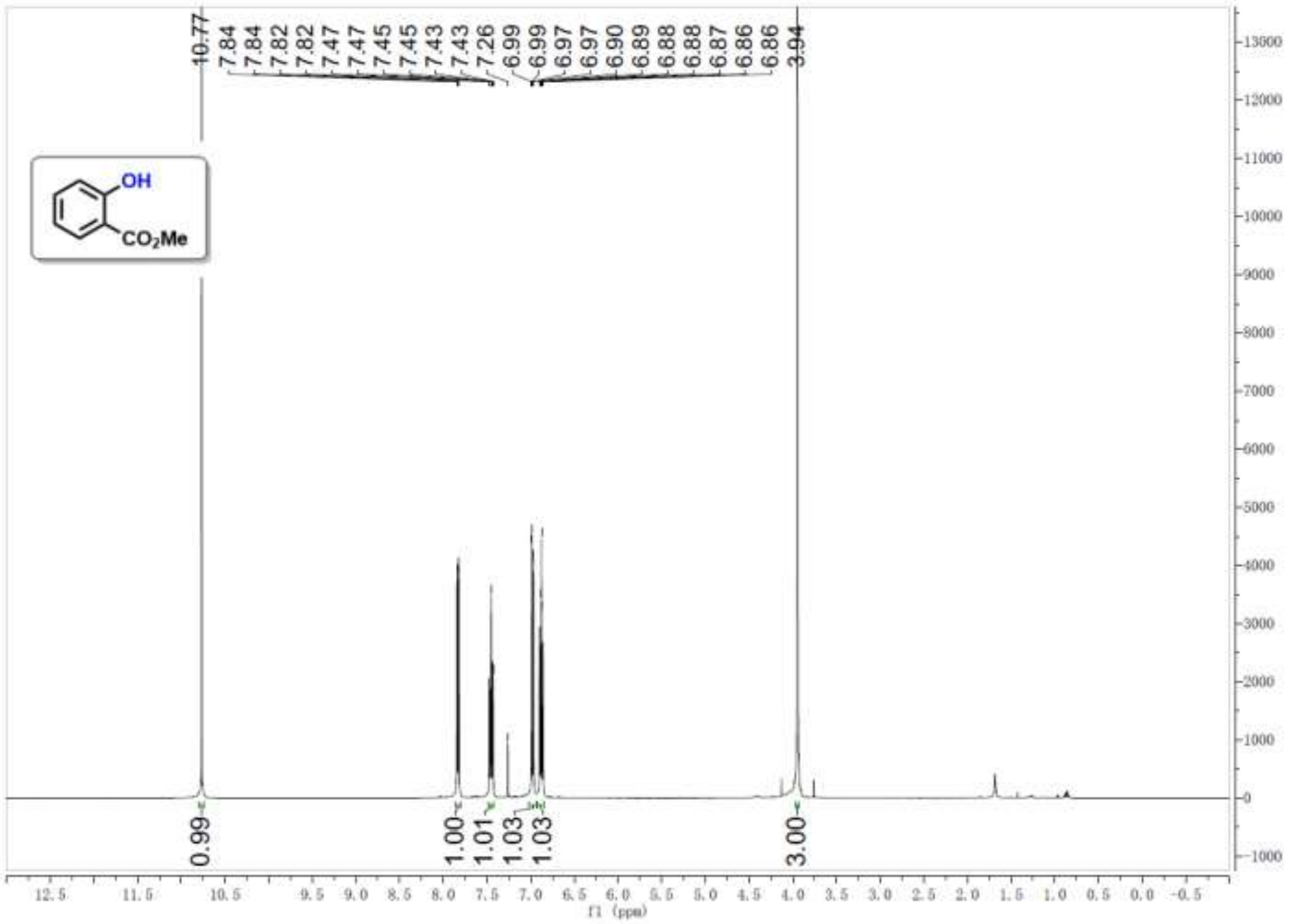
OH
CO2Me
10.77
7.84
7.84
7.82
7.82
7.47
7.47
7.45
7.45
7.43
7.43
7.26
6.99
6.99
6.97
6.97
6.90
6.89
6.88
6.88
6.87
6.86
6.86
3.94
0.99
1.00
1.01
1.03
1.03
3.00
f1 (ppm)

^{13}C NMR of 2n

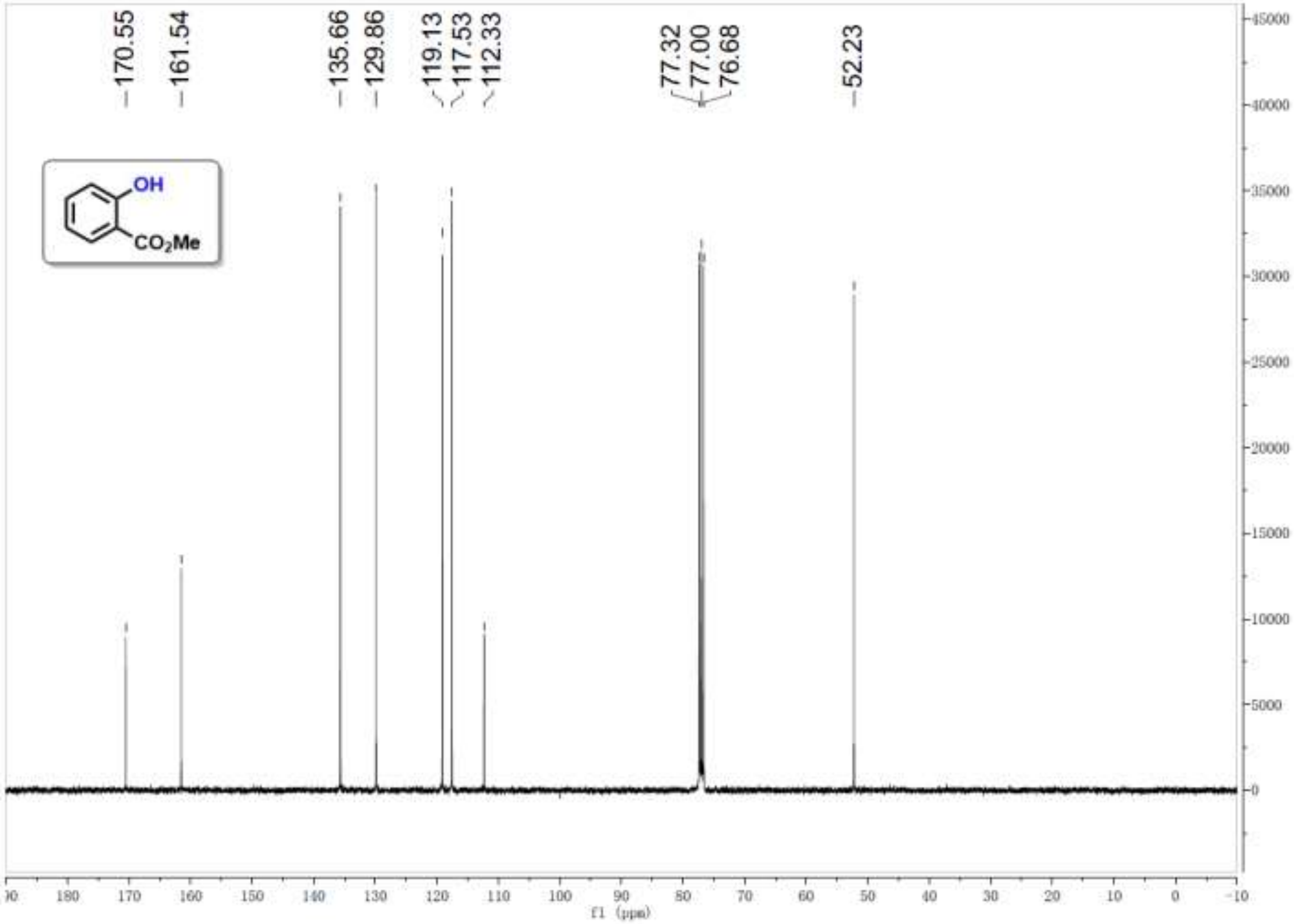

170.55
161.54
135.66
129.86
119.13
117.53
112.33
77.32
77.00
76.68
52.23
OH
CO2Me
f1 (ppm)

^{1}H NMR of 2o

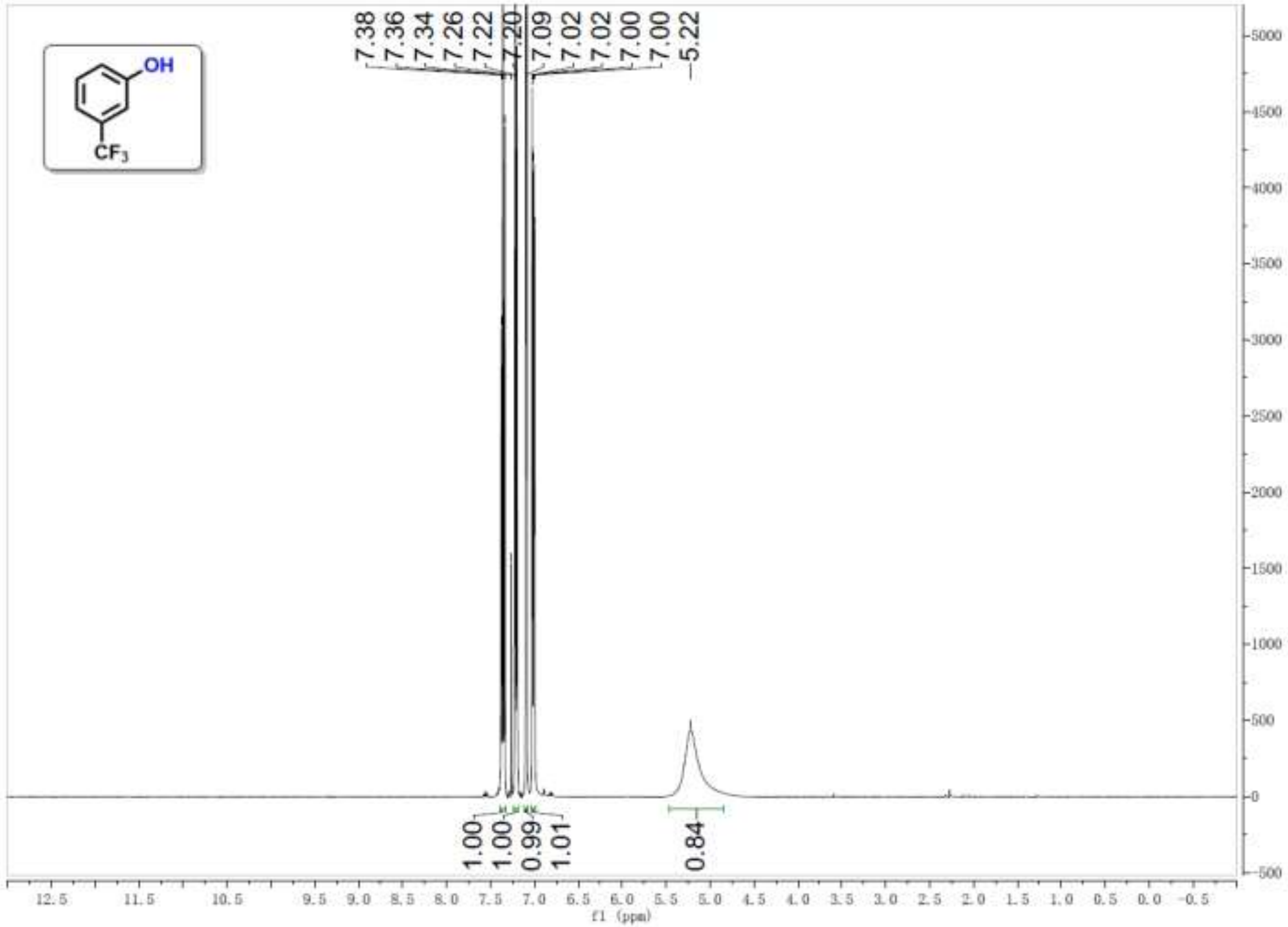
OH
CF3
7.38
7.36
7.34
7.26
7.22
7.20
7.09
7.02
7.02
7.00
7.00
5.22
1.00
1.00
0.99
1.01
0.84
f1 (ppm)

^{13}C NMR of 2o

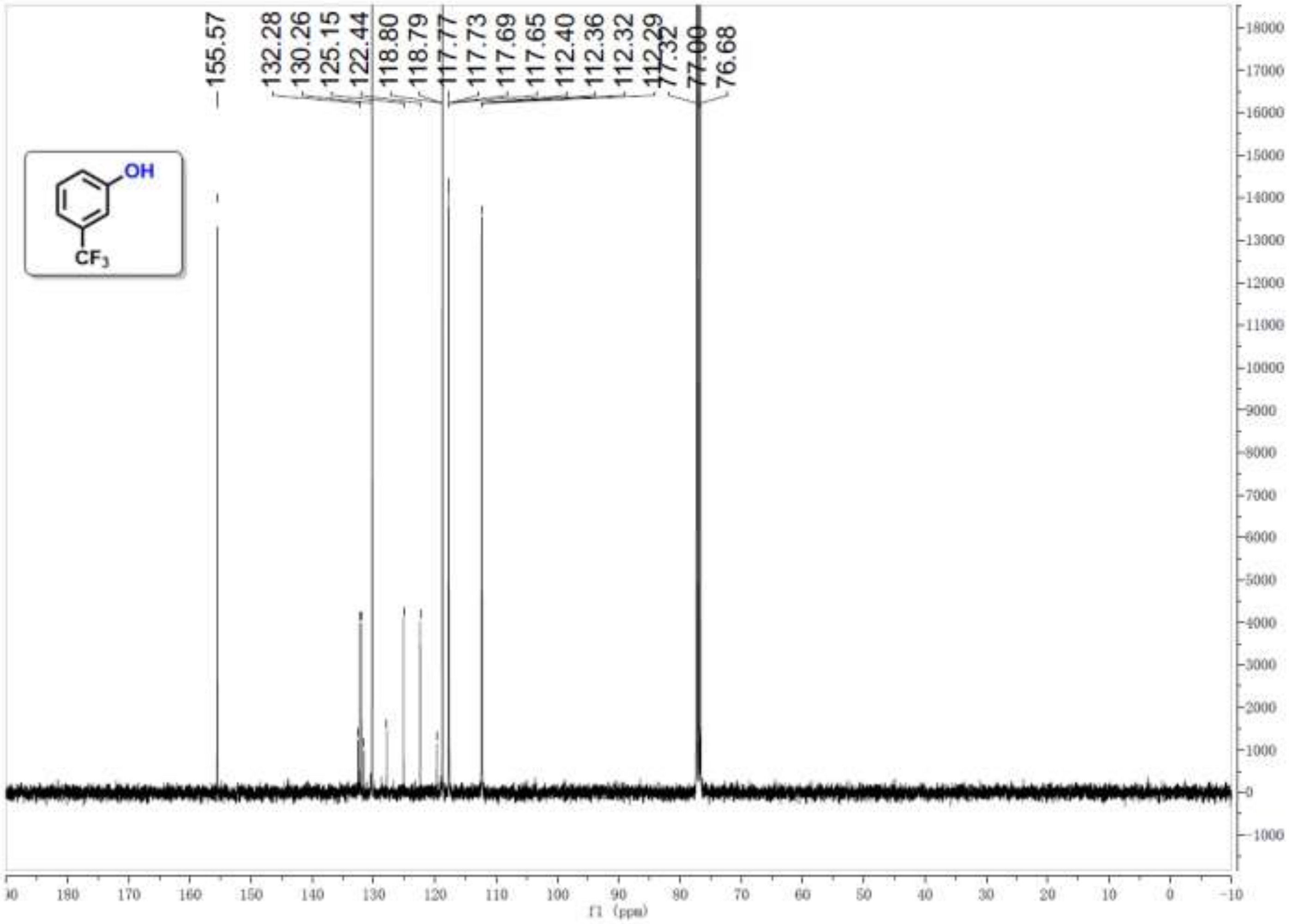

155.57
132.28
130.26
125.15
122.44
118.80
118.79
117.77
117.73
117.69
117.65
112.40
112.36
112.32
112.29
77.32
77.00
76.68
OH
CF3
f1 (ppm)

^{19}F NMR of 2o

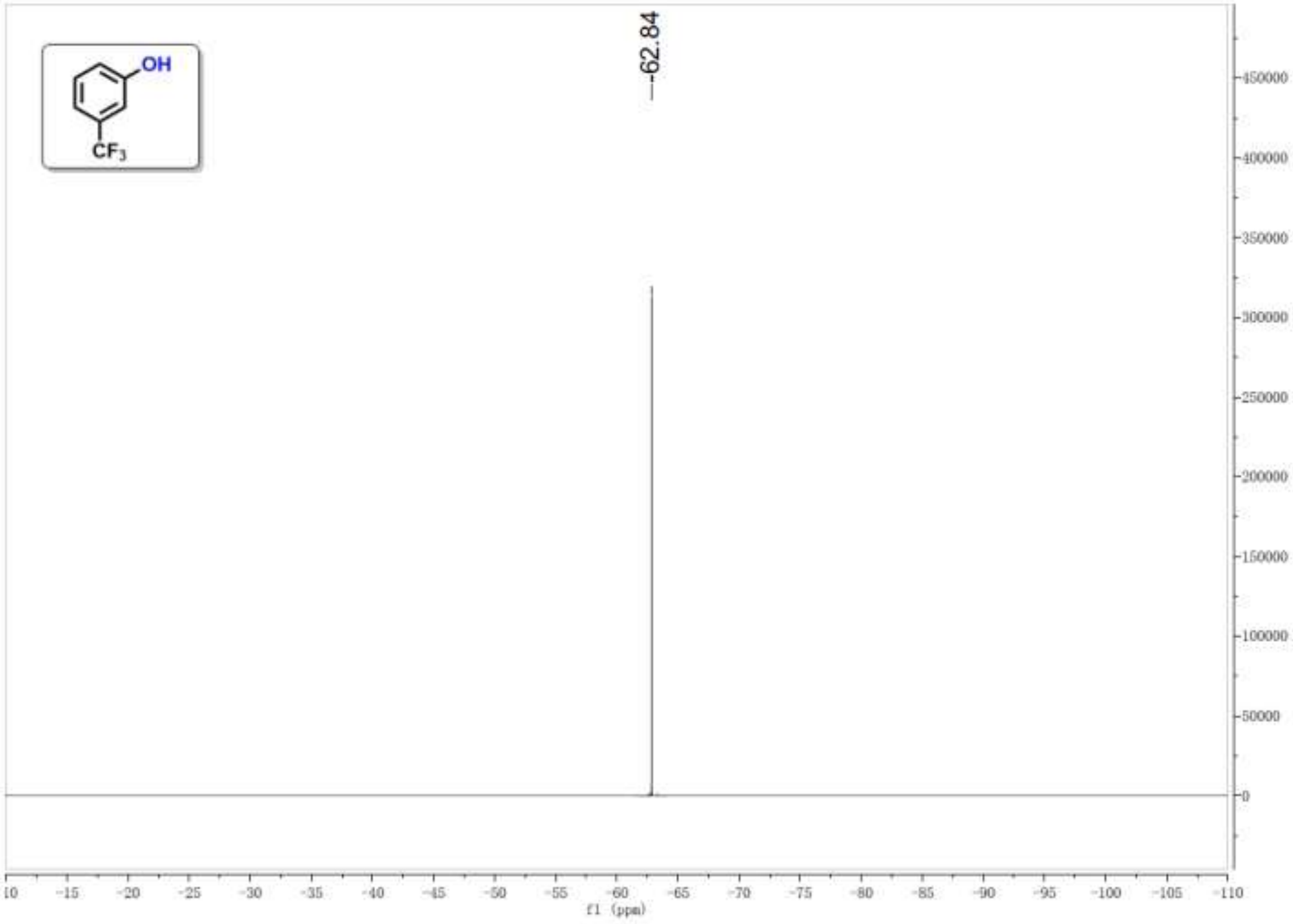

OH
CF3
-62.84
450000
400000
350000
300000
250000
200000
150000
100000
50000
0
10
-15
-20
-25
-30
-35
-40
-45
-50
-55
-60
-65
-70
-75
-80
-85
-90
-95
-100
-105
-110
f1 (ppm)

^{1}H NMR of 2p

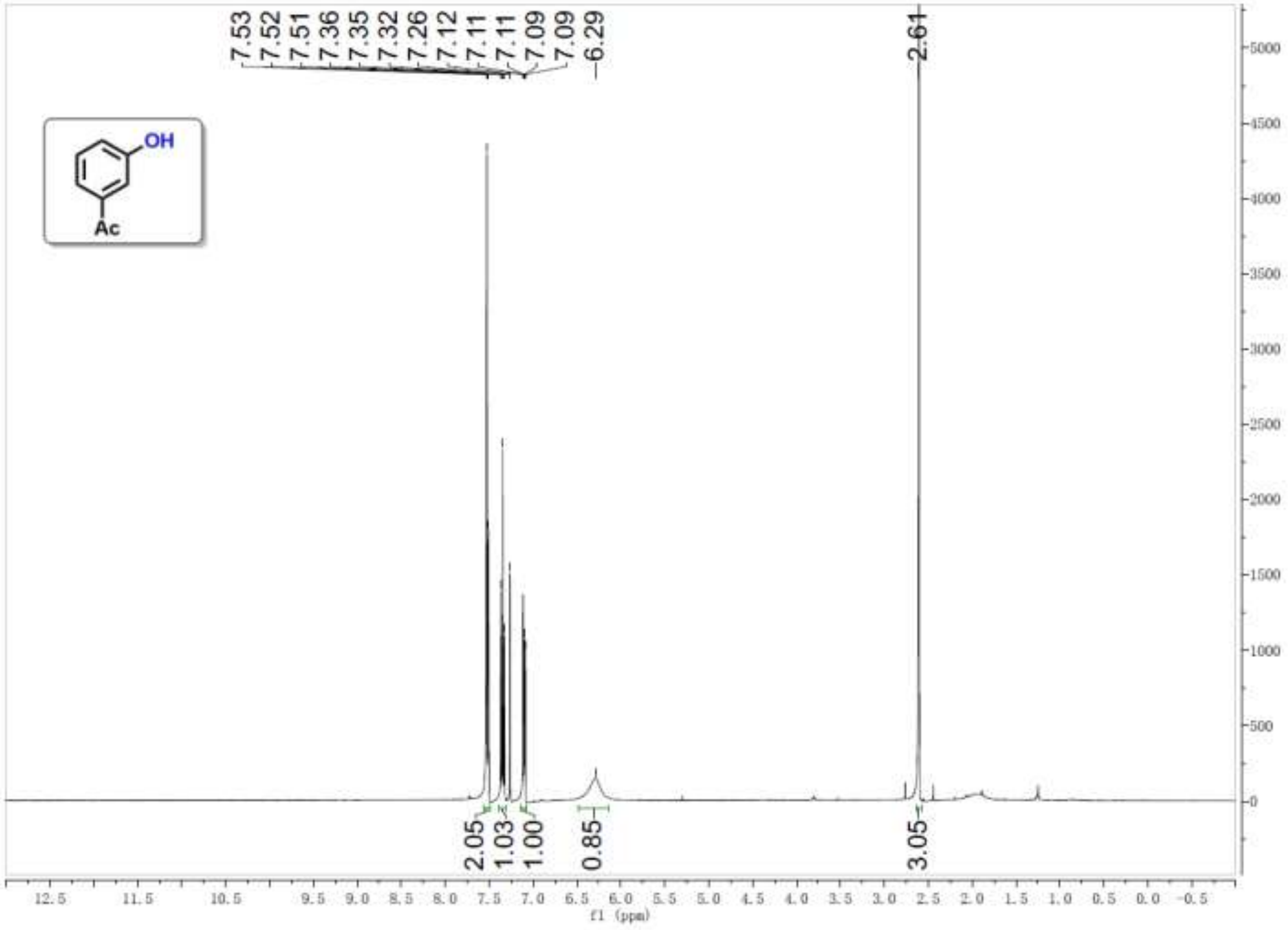

7.53
7.52
7.51
7.36
7.35
7.32
7.26
7.12
7.11
7.11
7.09
7.09
6.29
2.61
OH
Ac
2.05
1.03
1.00
0.85
3.05
f1 (ppm)

^{13}C NMR of 2p

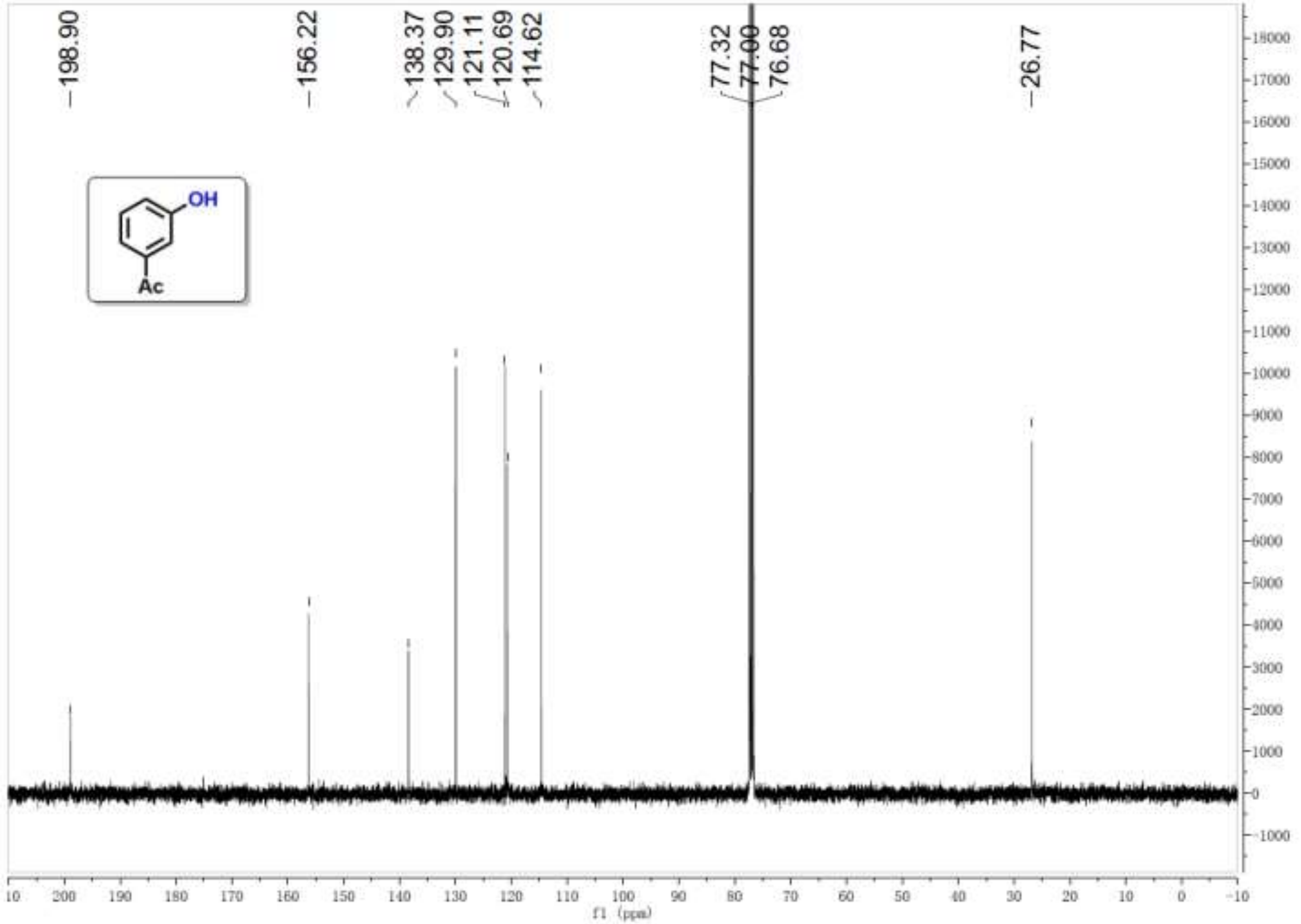
198.90
156.22
138.37
129.90
121.11
120.69
114.62
77.32
77.00
76.68
26.77
OH
Ac
18000
17000
16000
15000
14000
13000
12000
11000
10000
9000
8000
7000
6000
5000
4000
3000
2000
1000
0
-1000
10 200 190 180 170 160 150 140 130 120 110 100 90 80 70 60 50 40 30 20 10 0 -10
f1 (ppm)

^{1}H NMR of 2q

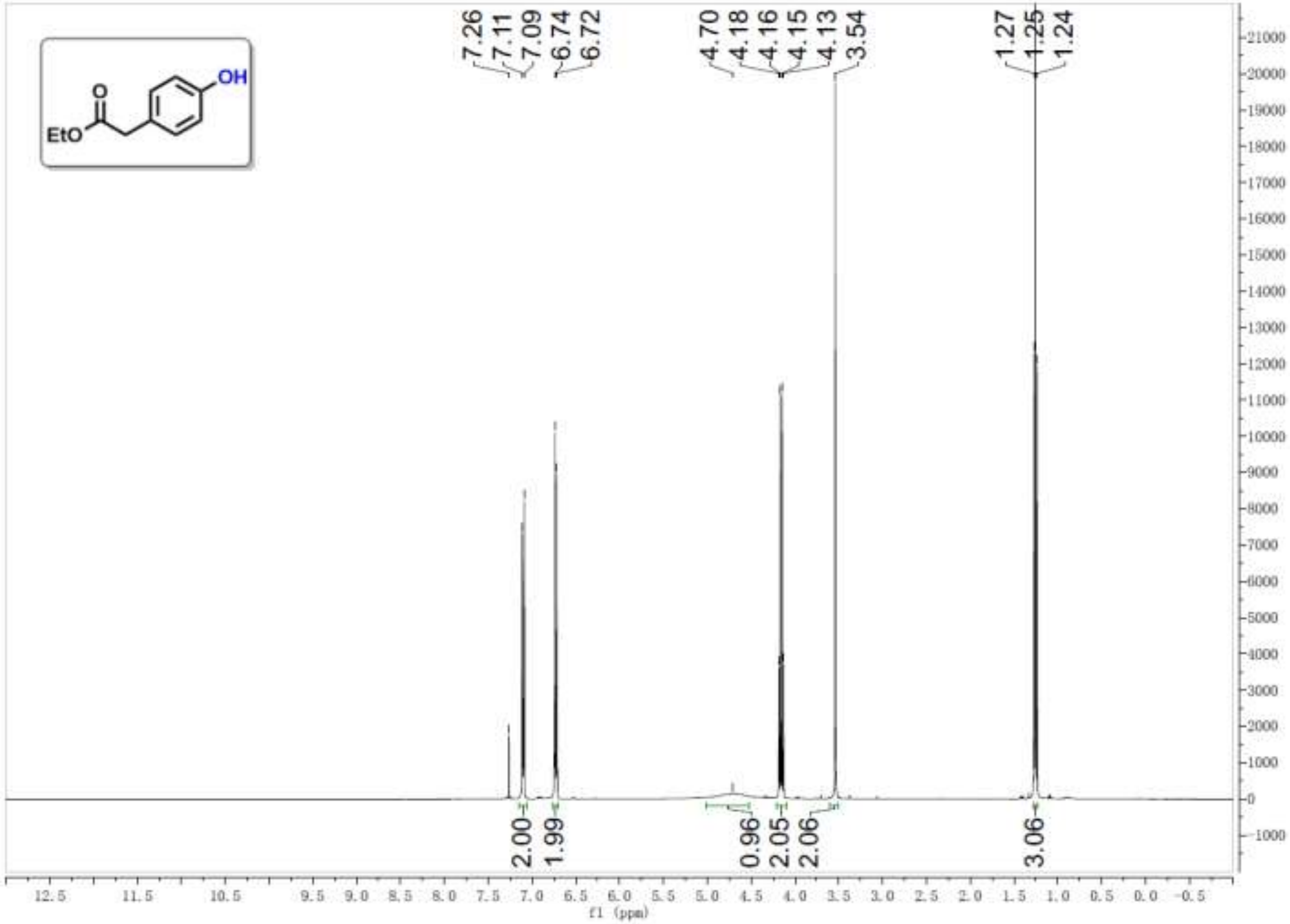

7.26
7.11
7.09
6.74
6.72
4.70
4.18
4.16
4.15
4.13
3.54
1.27
1.25
1.24
OH
EtO
O
2.00
1.99
0.96
2.05
2.06
3.06
f1 (ppm)

^{13}C NMR of 2q

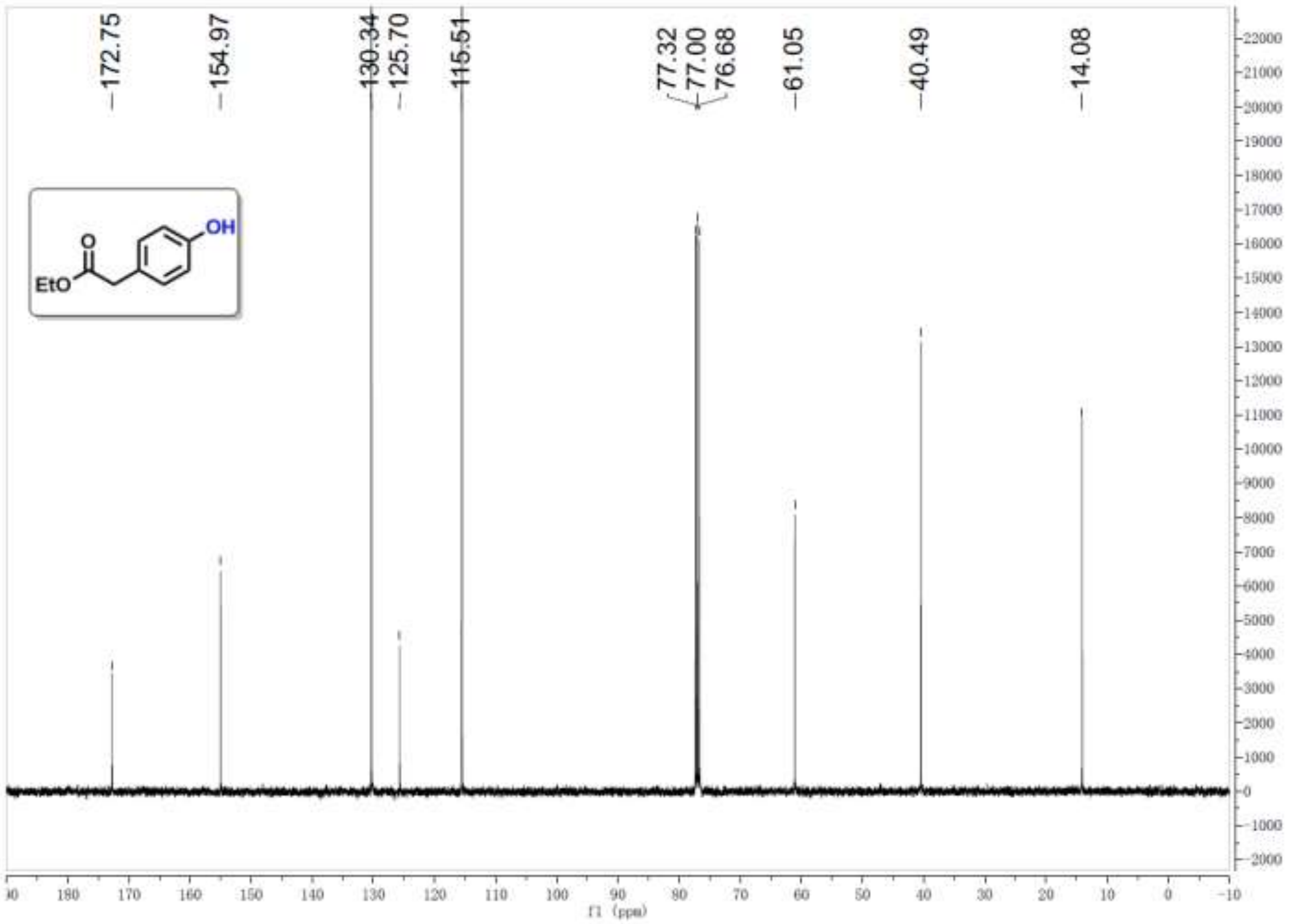

172.75
154.97
130.34
125.70
115.51
77.32
77.00
76.68
61.05
40.49
14.08
OH
O
EtO
f1 (ppm)

^{1}H NMR of 2r

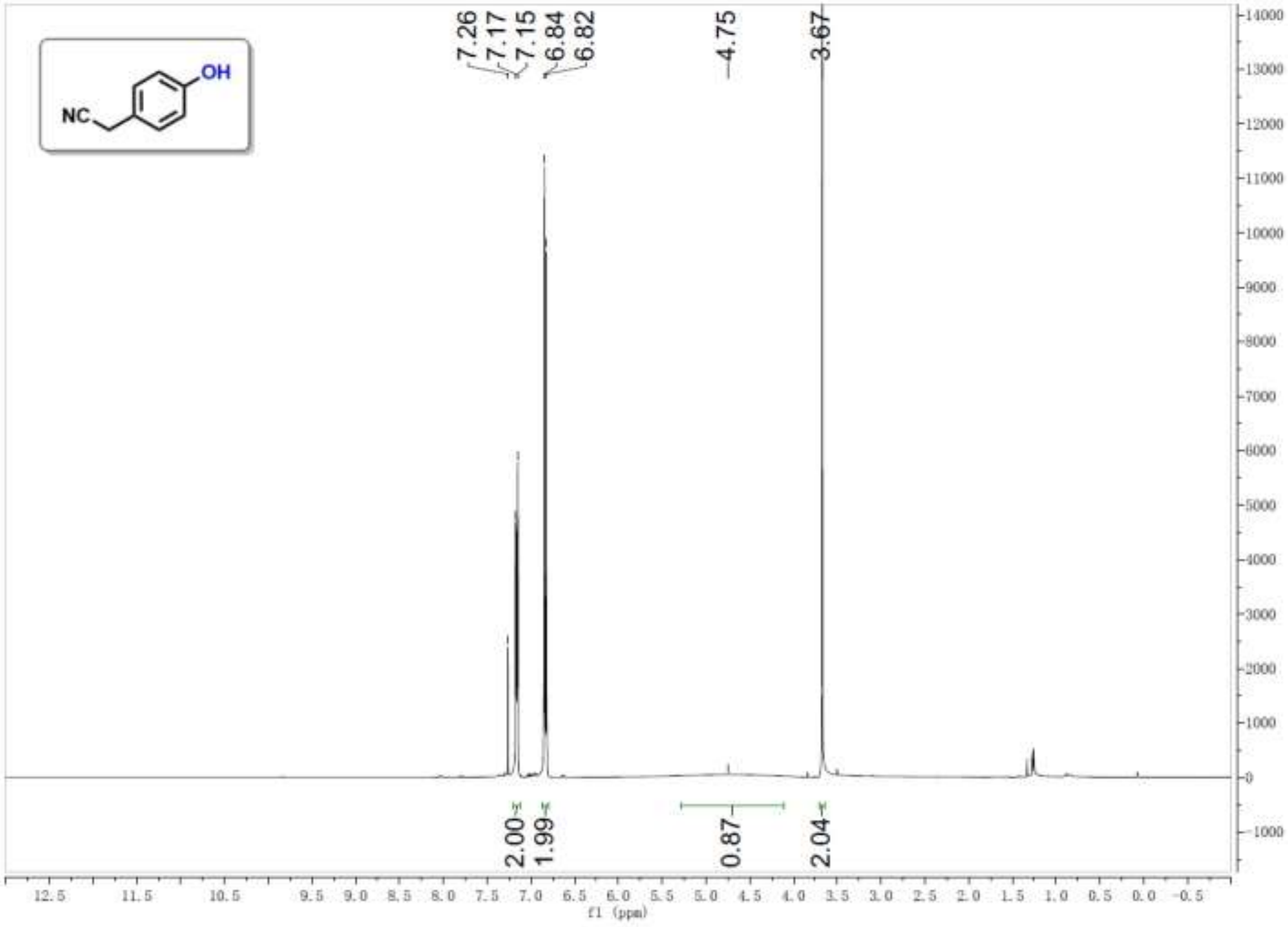

^{13}C NMR of 2r

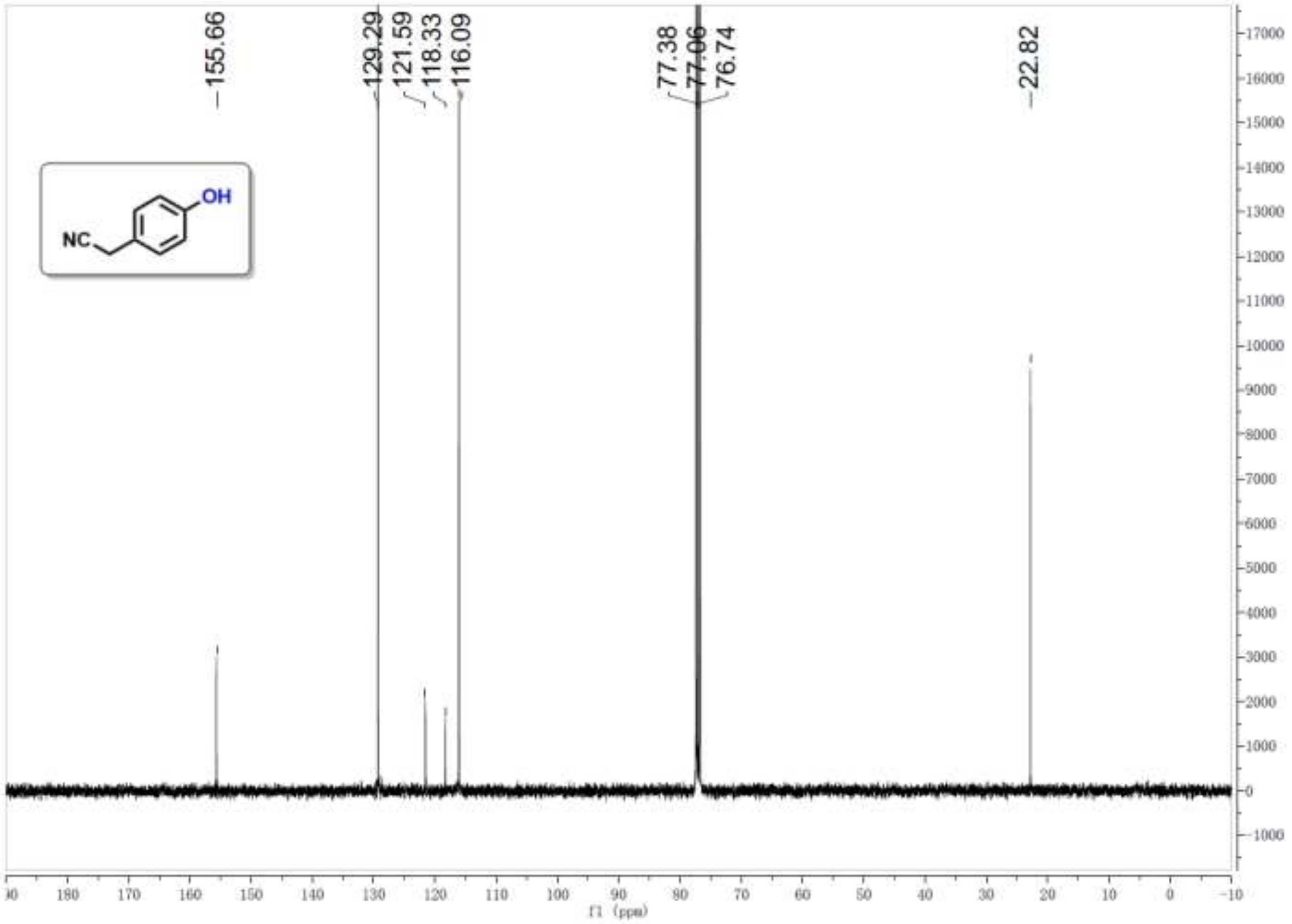

155.66
129.29
121.59
118.33
116.09
77.38
77.06
76.74
22.82
NC
OH
f1 (ppm)

^{1}H NMR of 2s

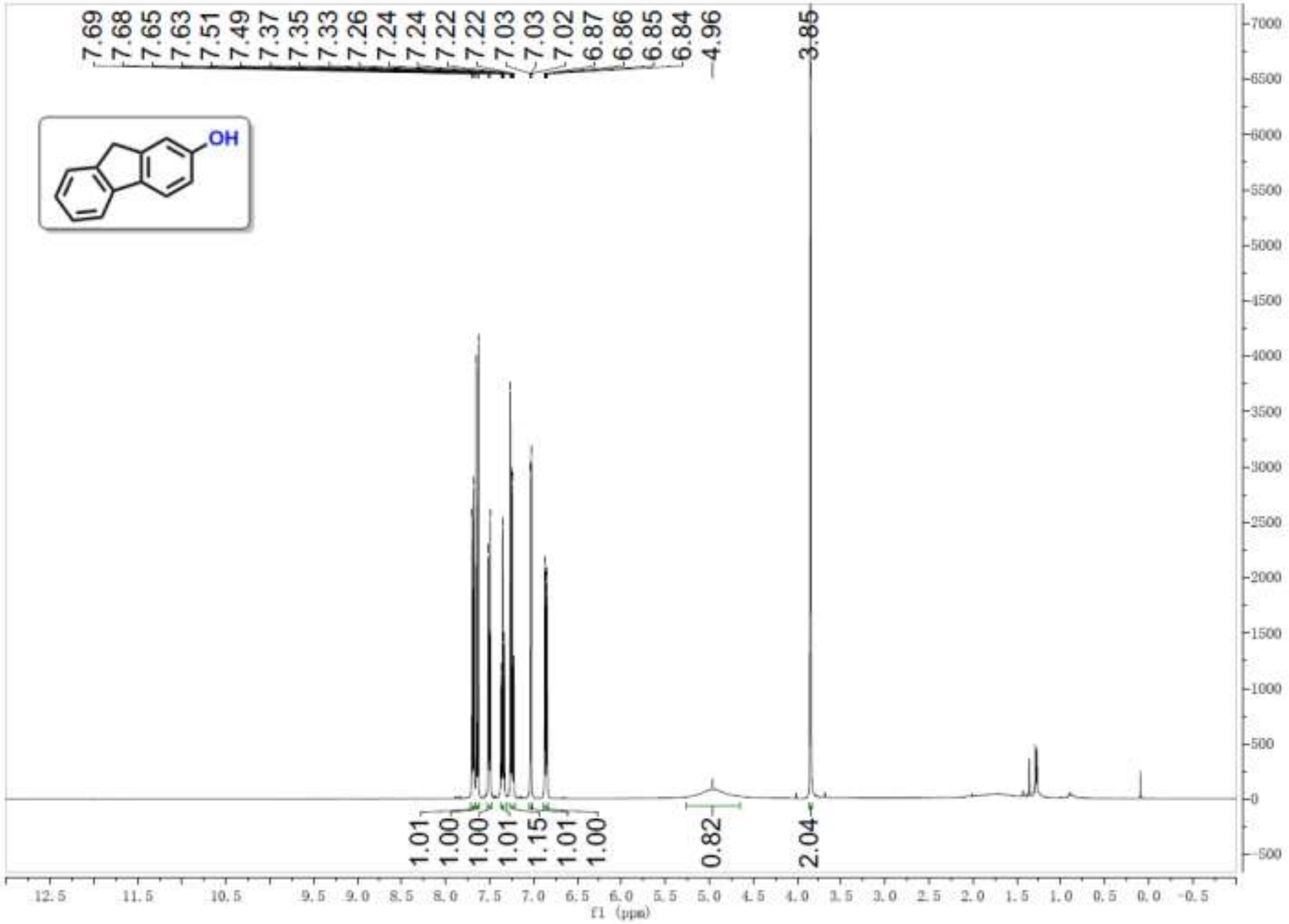

7.69
7.68
7.65
7.63
7.51
7.49
7.37
7.35
7.33
7.26
7.24
7.24
7.22
7.22
7.03
7.03
7.02
6.87
6.86
6.85
6.84
4.96
3.85
OH
1.01
1.00
1.00
1.01
1.15
1.01
1.00
0.82
2.04
f1 (ppm)

^{13}C NMR of 2s

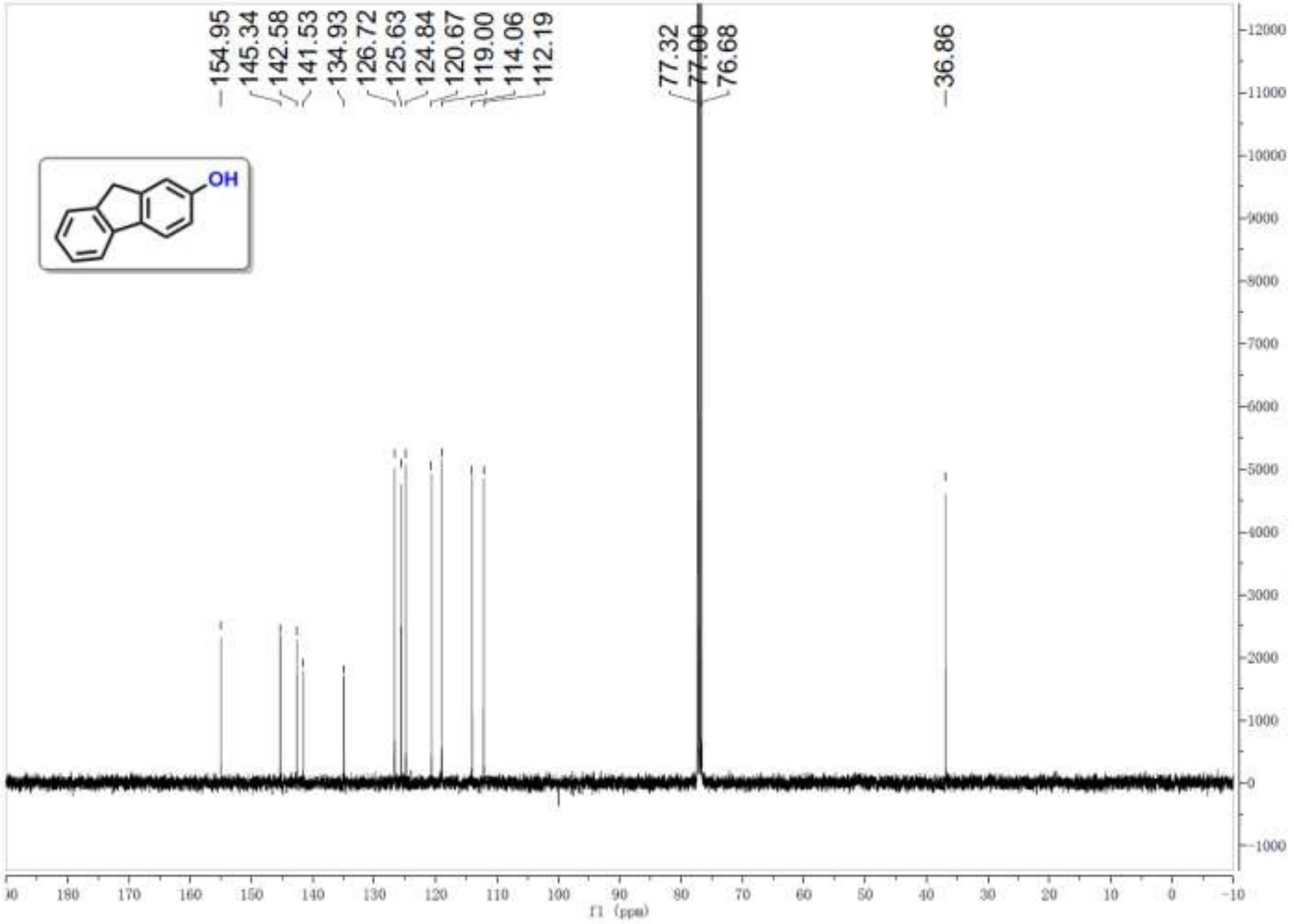

154.95
145.34
142.58
141.53
134.93
126.72
125.63
124.84
120.67
119.00
114.06
112.19
77.32
77.00
76.68
36.86
OH
f1 (ppm)

^{1}H NMR of 2t

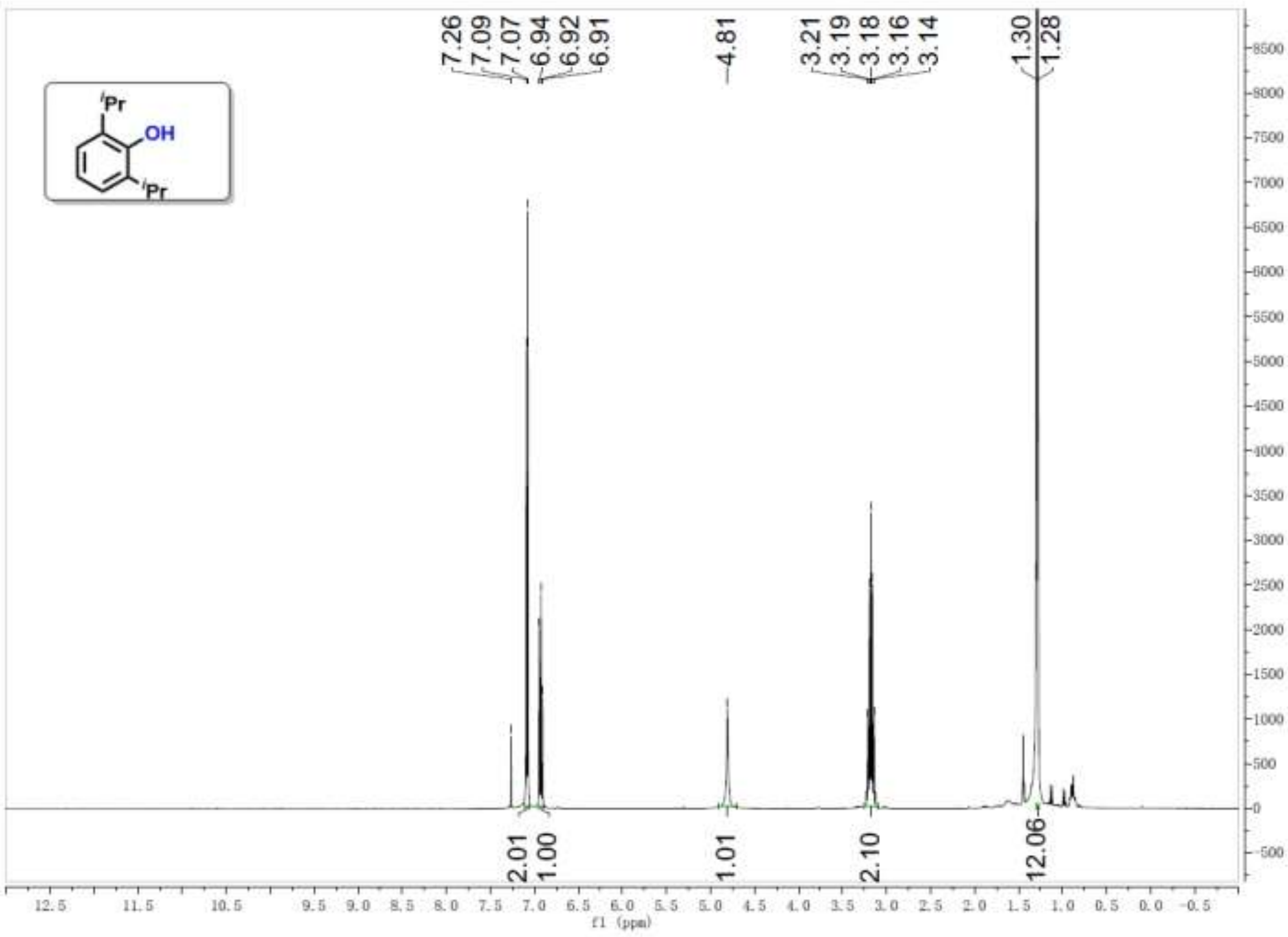

7.26
7.09
7.07
6.94
6.92
6.91
4.81
3.21
3.19
3.18
3.16
3.14
1.30
1.28
iPr
OH
iPr
2.01
1.00
1.01
2.10
12.06
f1 (ppm)

^{13}C NMR of 2t

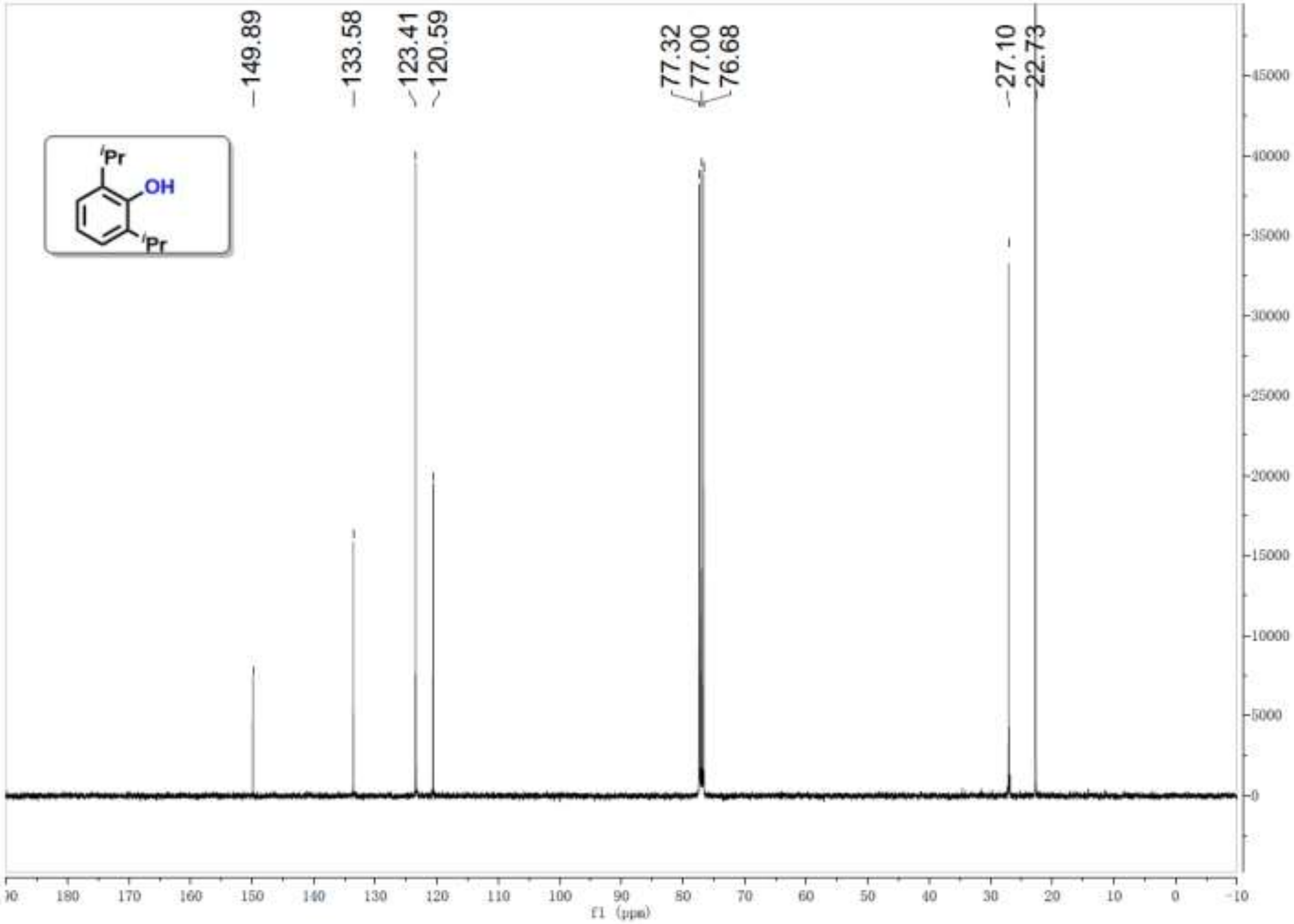

149.89
133.58
123.41
120.59
77.32
77.00
76.68
27.10
22.73
iPr
OH
iPr
45000
40000
35000
30000
25000
20000
15000
10000
5000
0
90
180
170
160
150
140
130
120
110
100
90
80
70
60
50
40
30
20
10
0
-10
f1 (ppm)

^{1}H NMR of 2u

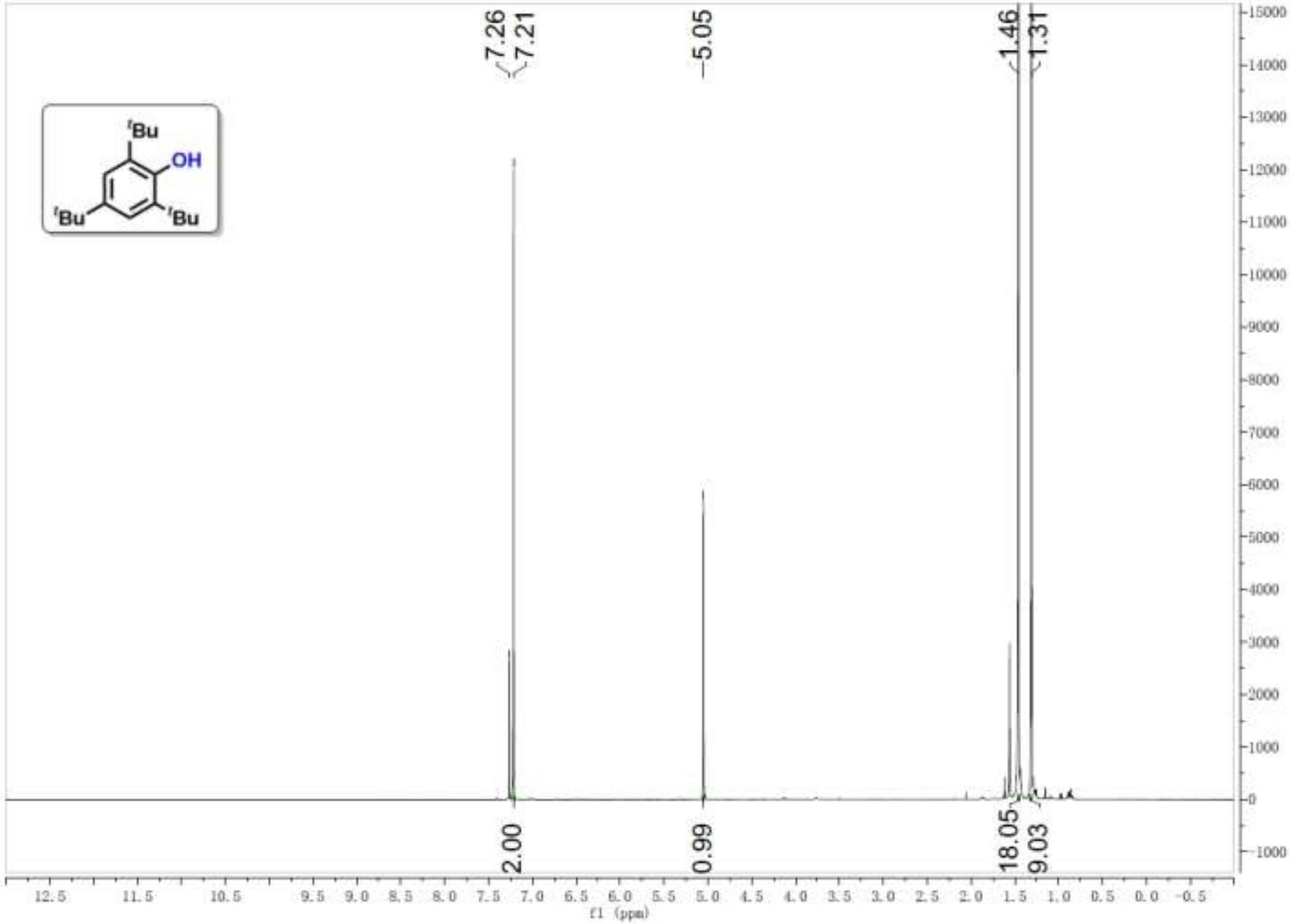

7.26
7.21
5.05
1.46
1.31
tBu
OH
tBu
tBu
2.00
0.99
18.05
9.03
12.5 11.5 10.5 9.5 9.0 8.5 8.0 7.5 7.0 6.5 6.0 5.5 5.0 4.5 4.0 3.5 3.0 2.5 2.0 1.5 1.0 0.5 0.0 -0.5
f1 (ppm)
15000 14000 13000 12000 11000 10000 9000 8000 7000 6000 5000 4000 3000 2000 1000 0 -1000

^{13}C NMR of 2u

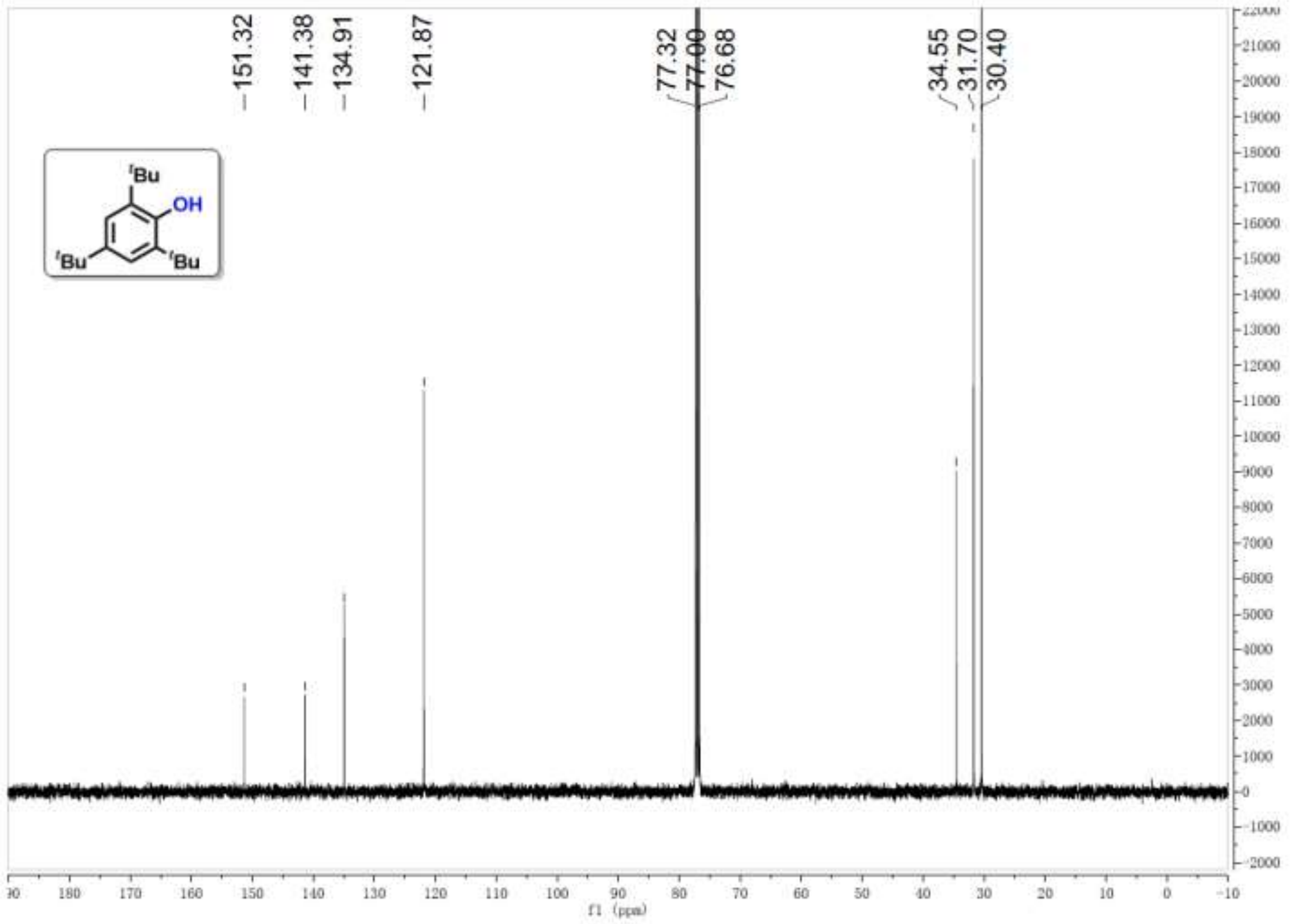

151.32
141.38
134.91
121.87
77.32
77.00
76.68
34.55
31.70
30.40
tBu
OH
tBu
tBu
f1 (ppm)

^{1}H NMR of 2v

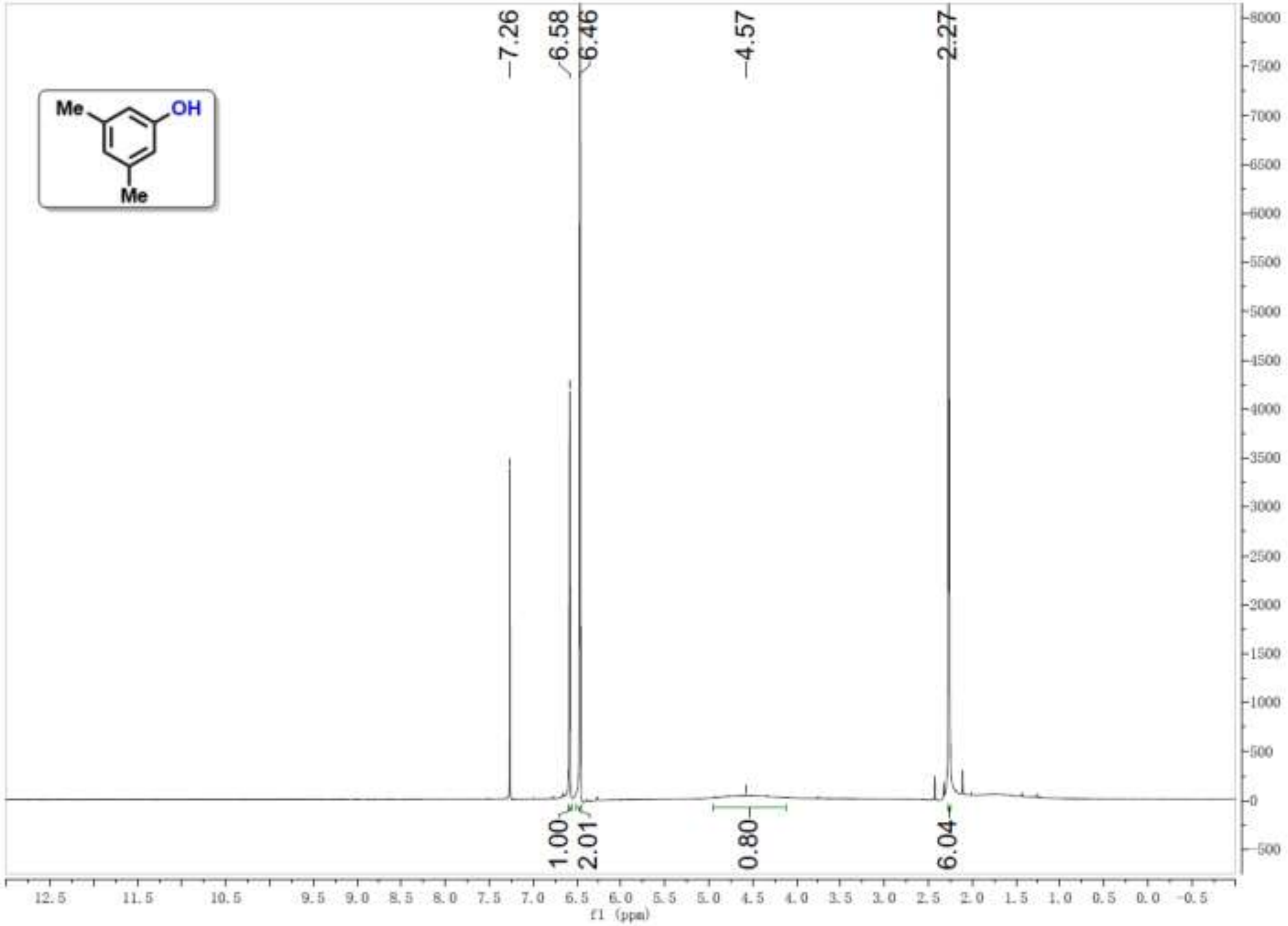

^{13}C NMR of 2v

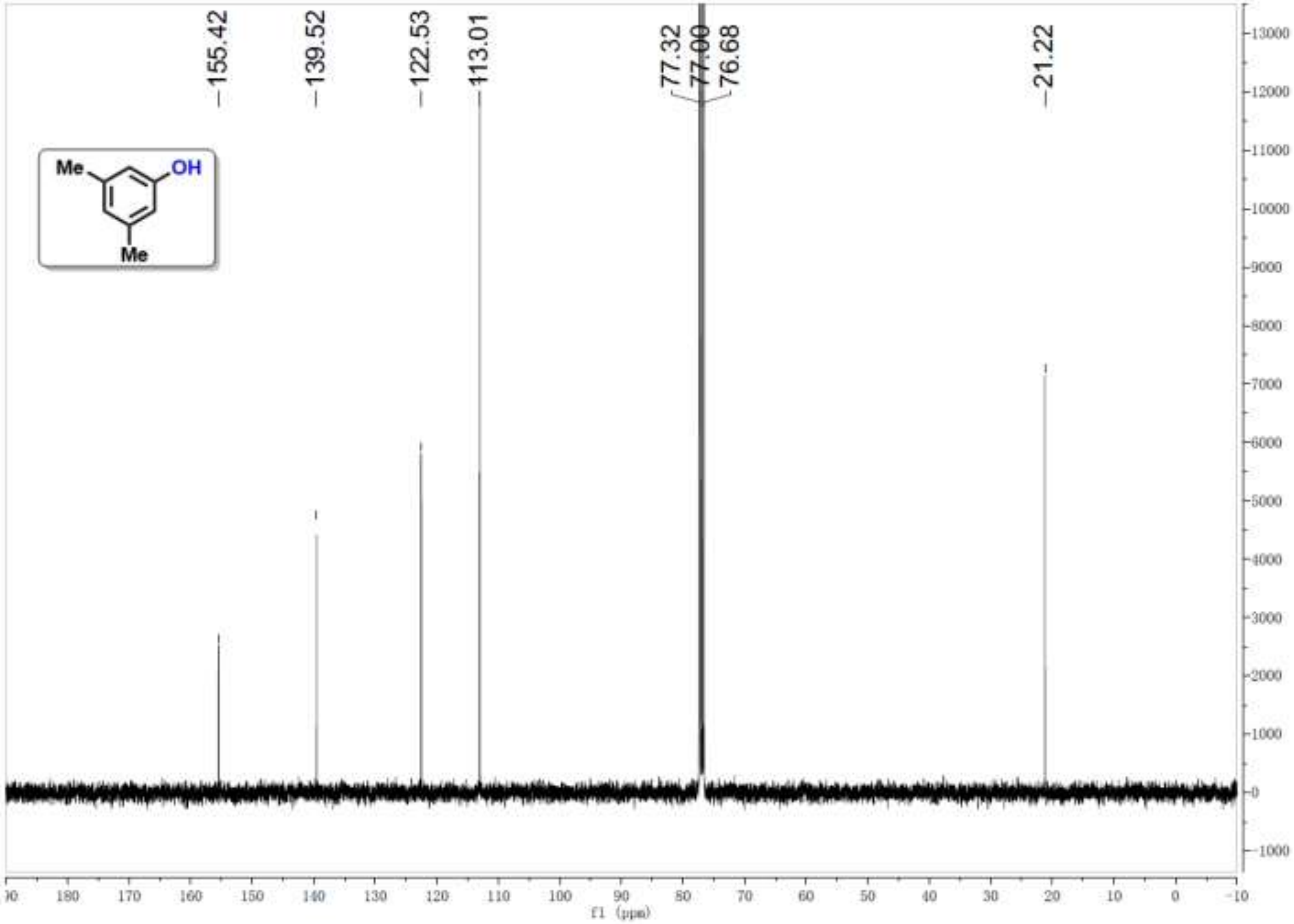

^{1}H NMR of 2w

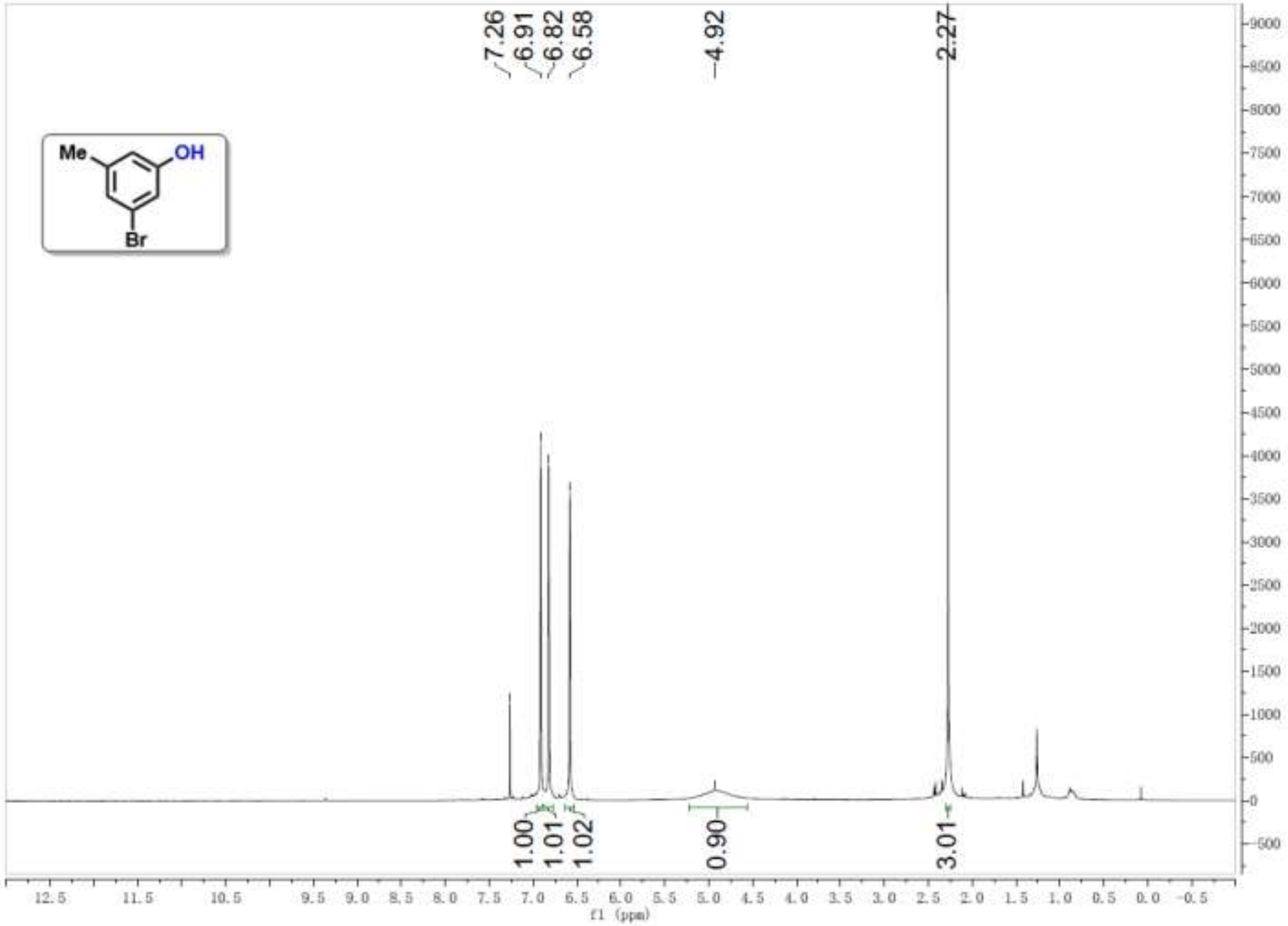

^{13}C NMR of 2w

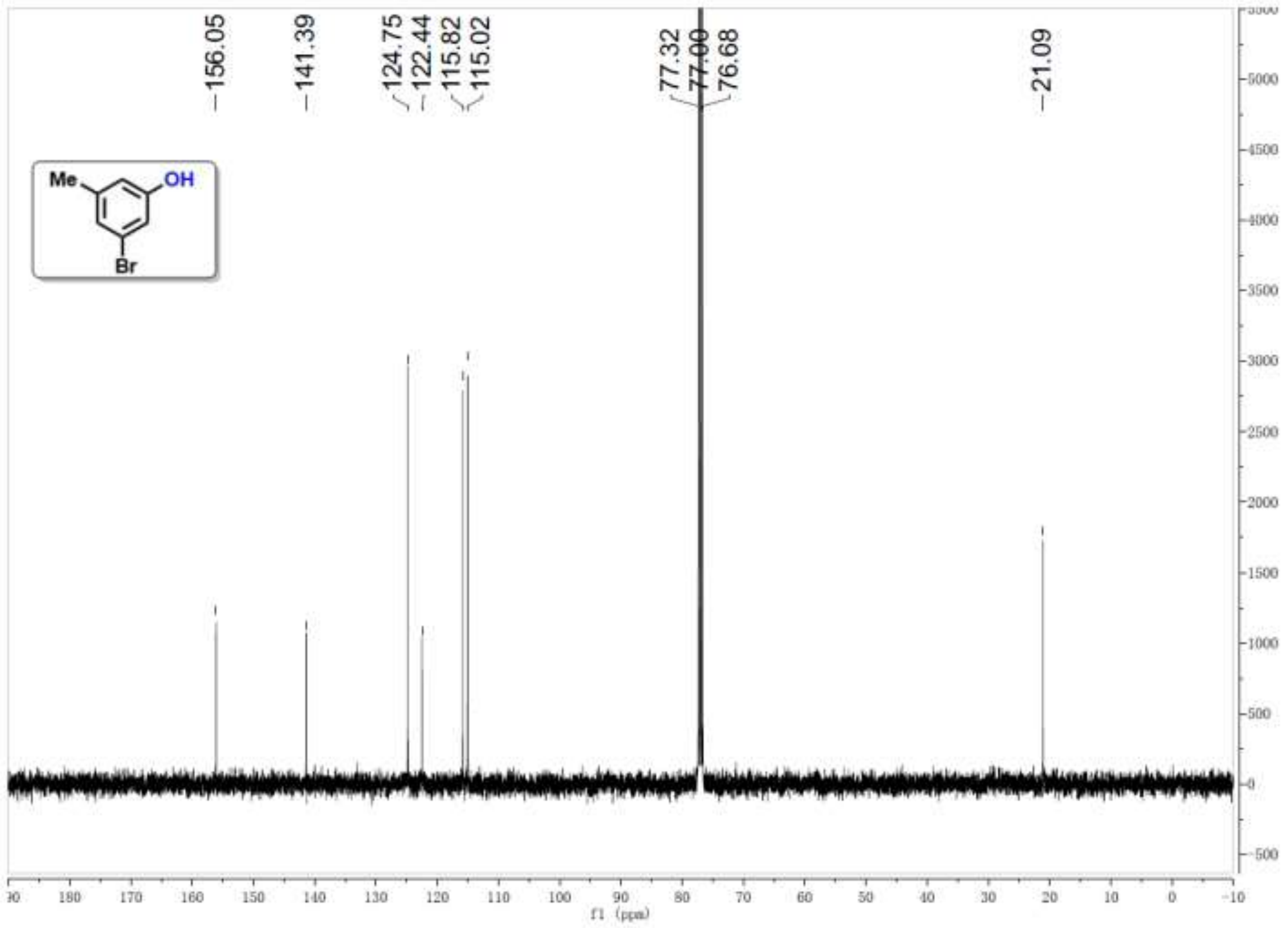

156.05
141.39
124.75
122.44
115.82
115.02
77.32
77.00
76.68
21.09
Me
OH
Br
f1 (ppm)

^{1}H NMR of 2x

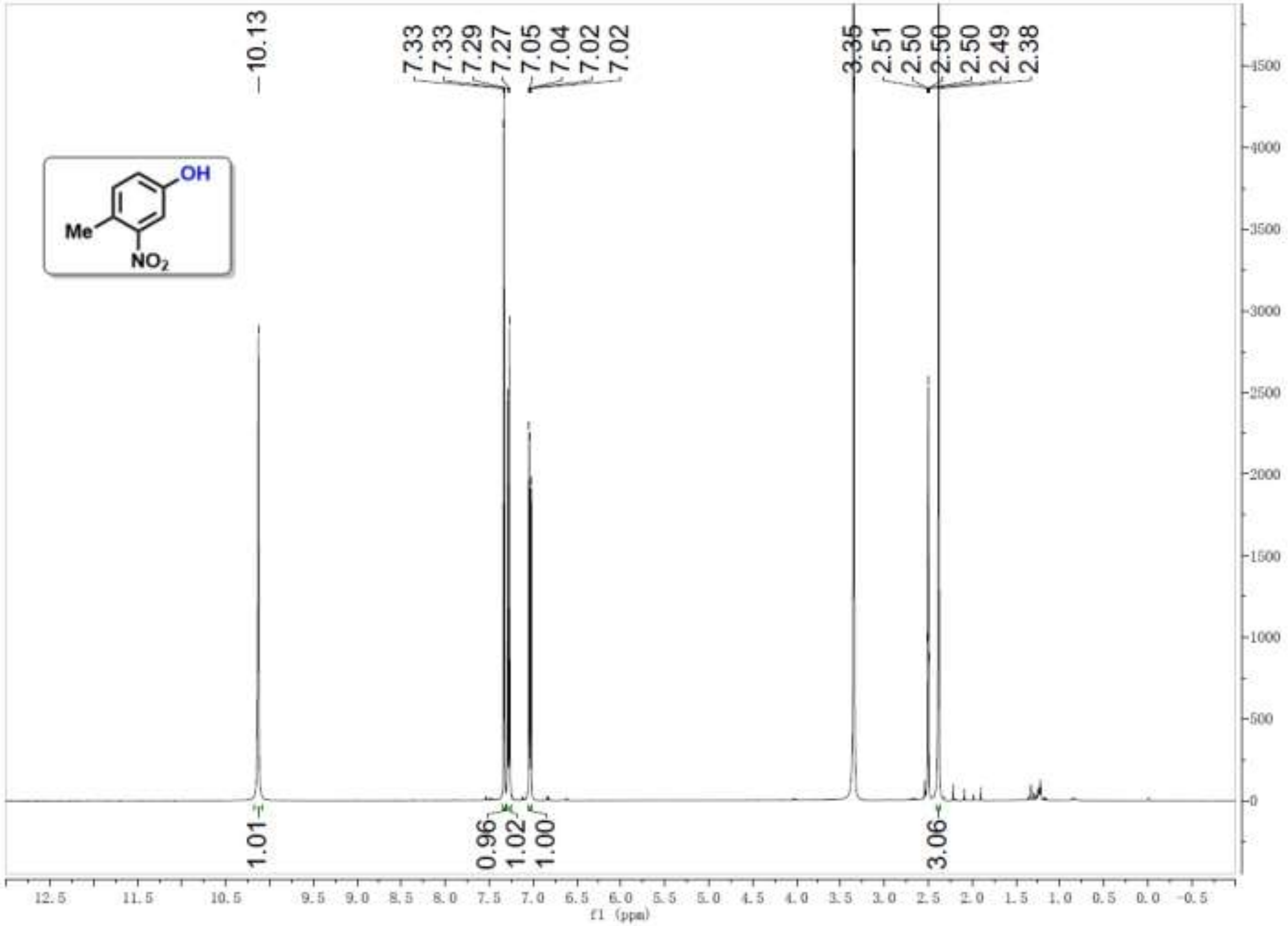

10.13
7.33
7.33
7.29
7.27
7.05
7.04
7.02
7.02
3.35
2.51
2.50
2.50
2.50
2.49
2.38
OH
Me
NO2
1.01
0.96
1.02
1.00
3.06
f1 (ppm)

^{13}C NMR of 2x

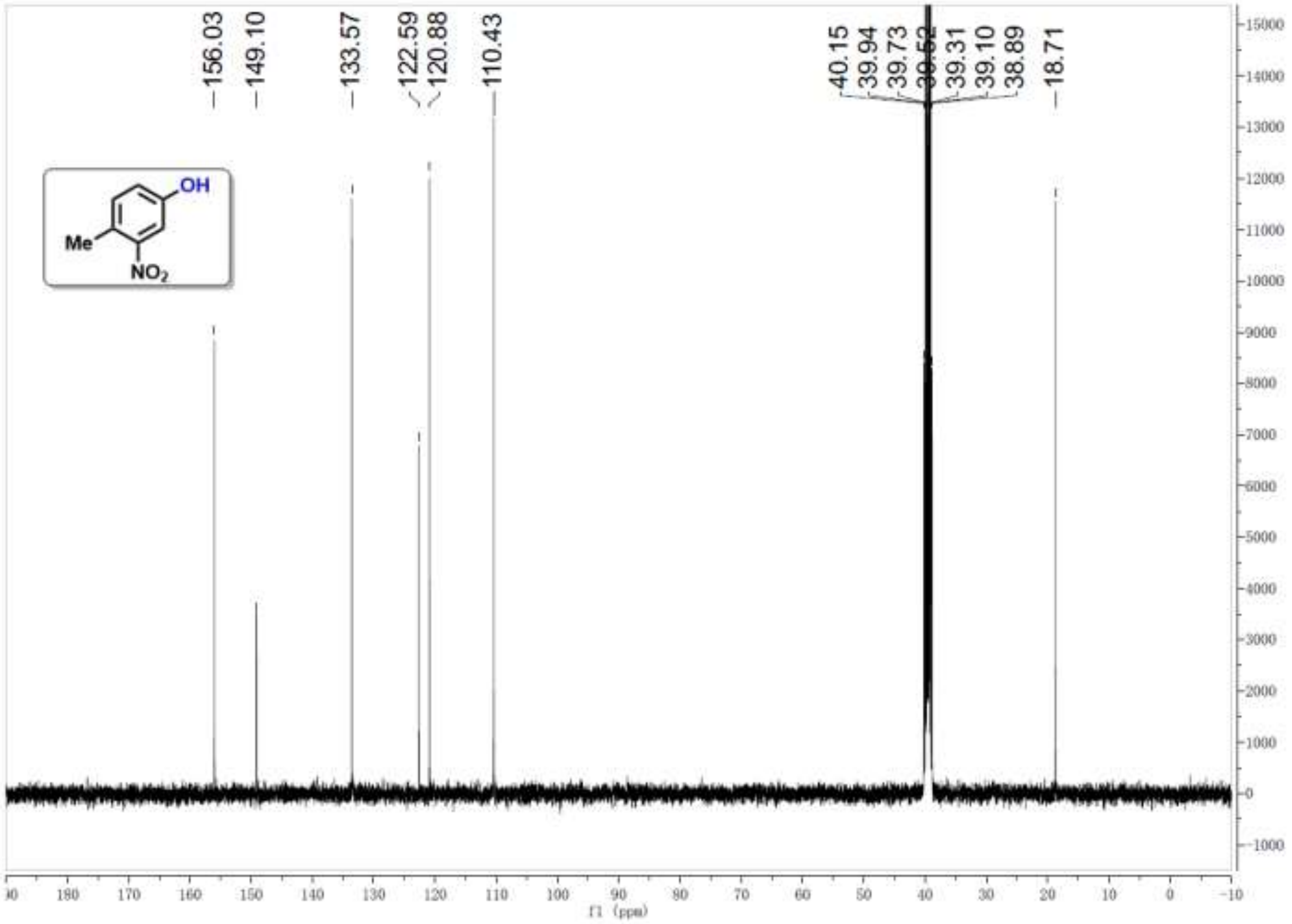

156.03
149.10
133.57
122.59
120.88
110.43
40.15
39.94
39.73
39.52
39.31
39.10
38.89
18.71
OH
Me
NO2
f1 (ppm)

^{1}H NMR of 2y

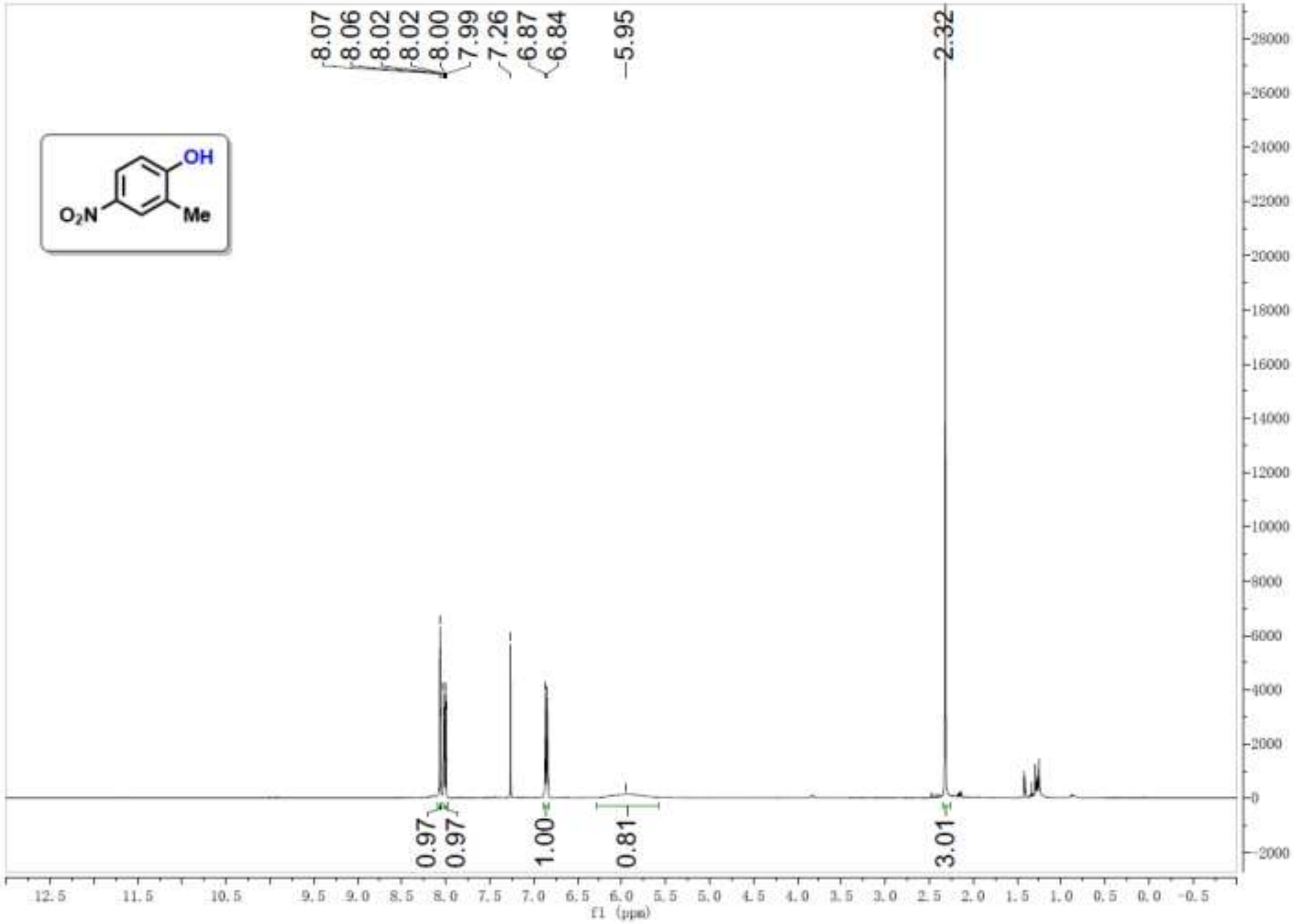

8.07
8.06
8.02
8.02
8.00
7.99
7.26
6.87
6.84
5.95
2.32
OH
O_2N
Me
0.97
0.97
1.00
0.81
3.01
f1 (ppm)

^{13}C NMR of 2y

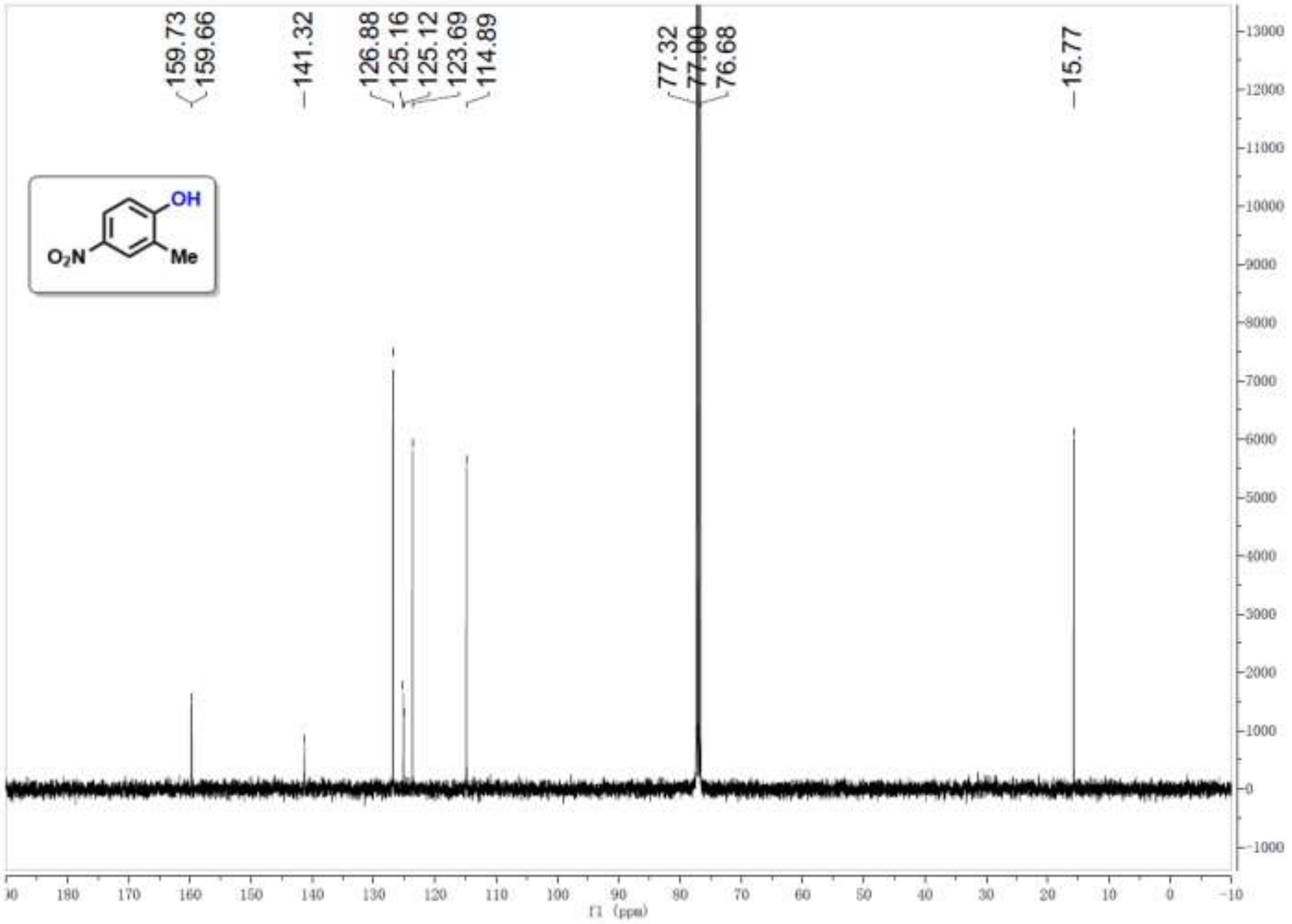

159.73
159.66
141.32
126.88
125.16
125.12
123.69
114.89
77.32
77.00
76.68
15.77
OH
O_2N
Me
f1 (ppm)

^{1}H NMR of 2z

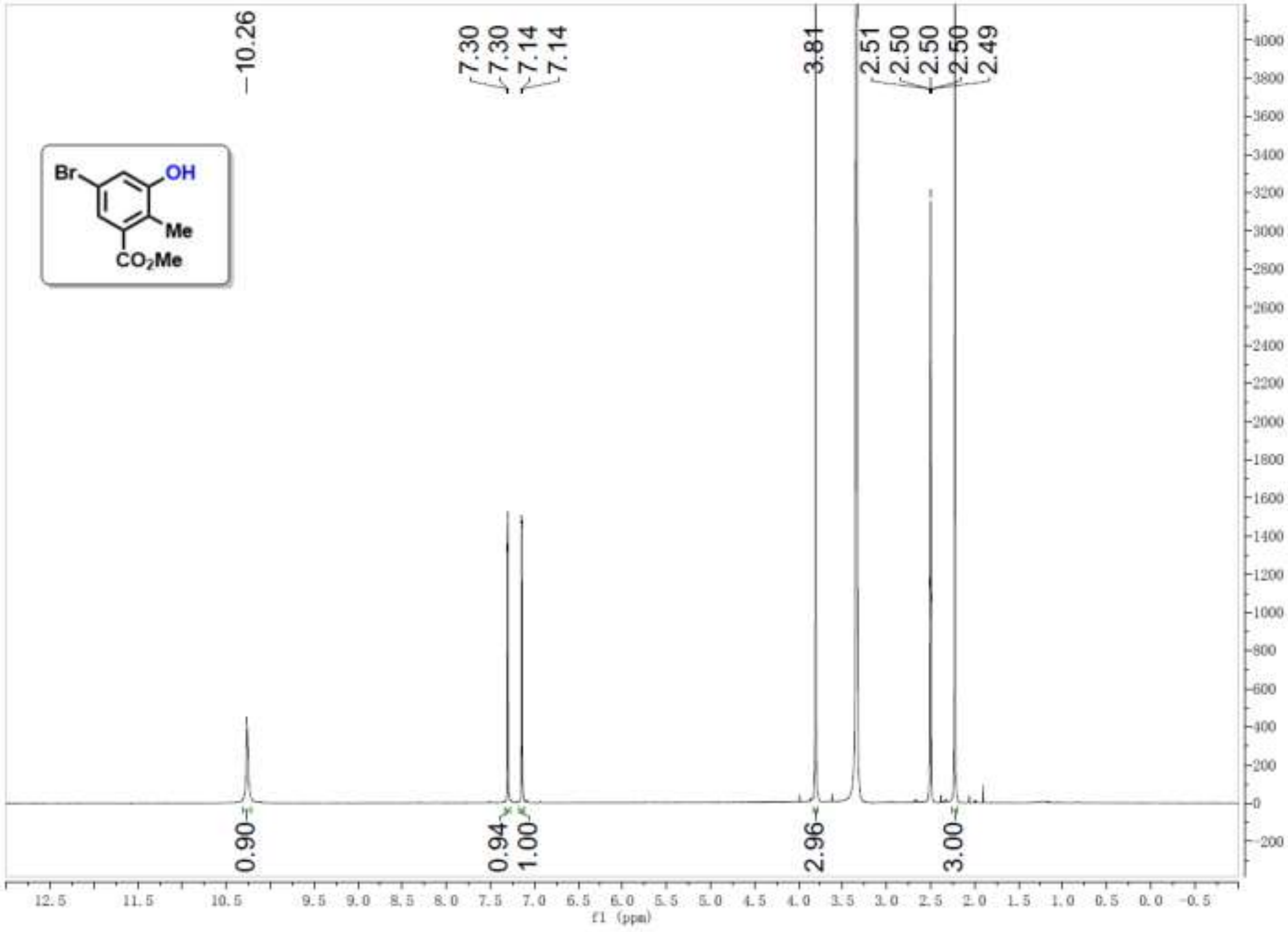

10.26
7.30
7.30
7.14
7.14
3.81
2.51
2.50
2.50
2.50
2.49
Br
OH
Me
CO2Me
0.90
0.94
1.00
2.96
3.00
f1 (ppm)

^{13}C NMR of 2z

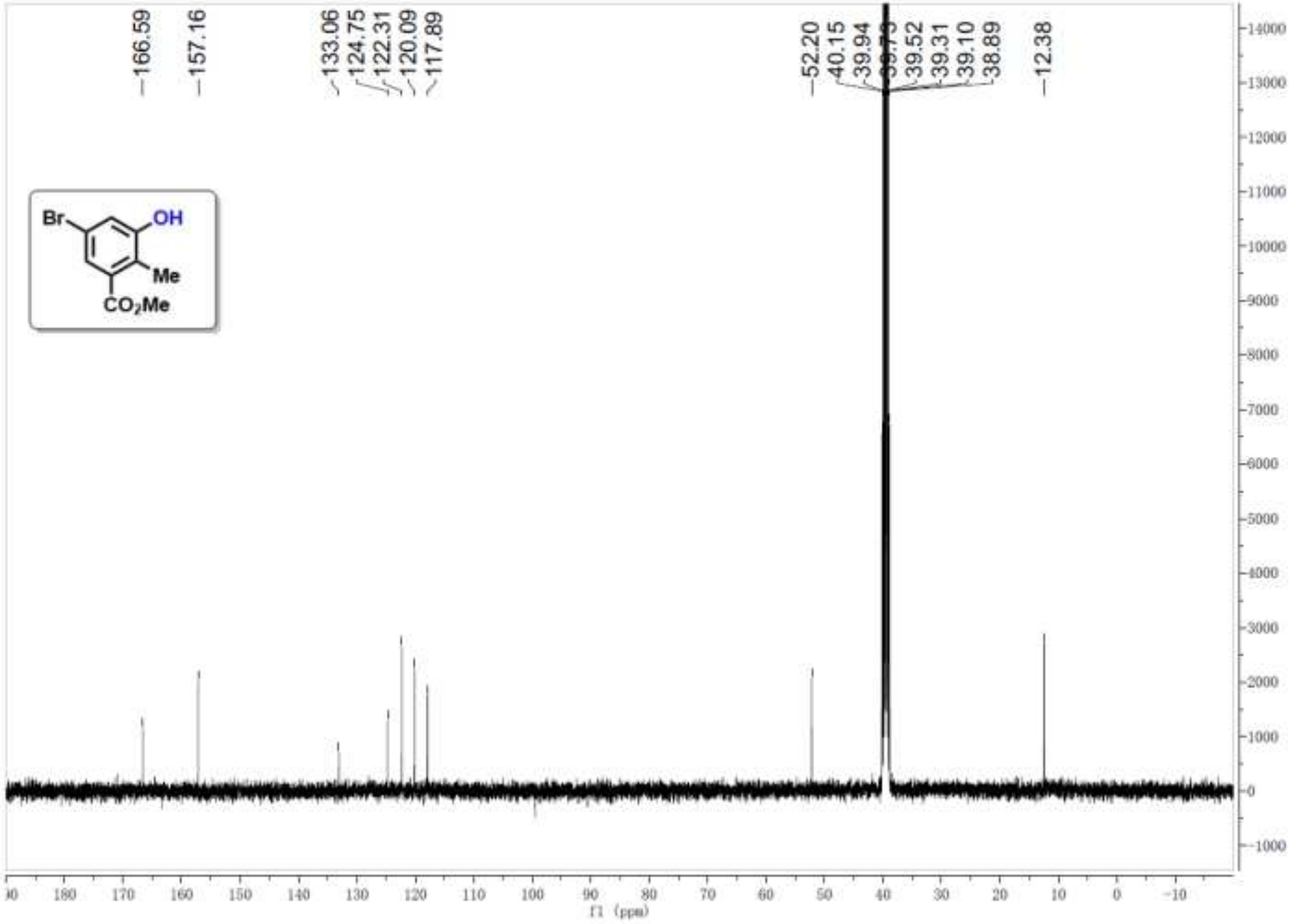

166.59
157.16
133.06
124.75
122.31
120.09
117.89
52.20
40.15
39.94
39.73
39.52
39.31
39.10
38.89
12.38
Br
OH
Me
CO2Me
f1 (ppm)

^{1}H NMR of 2aa

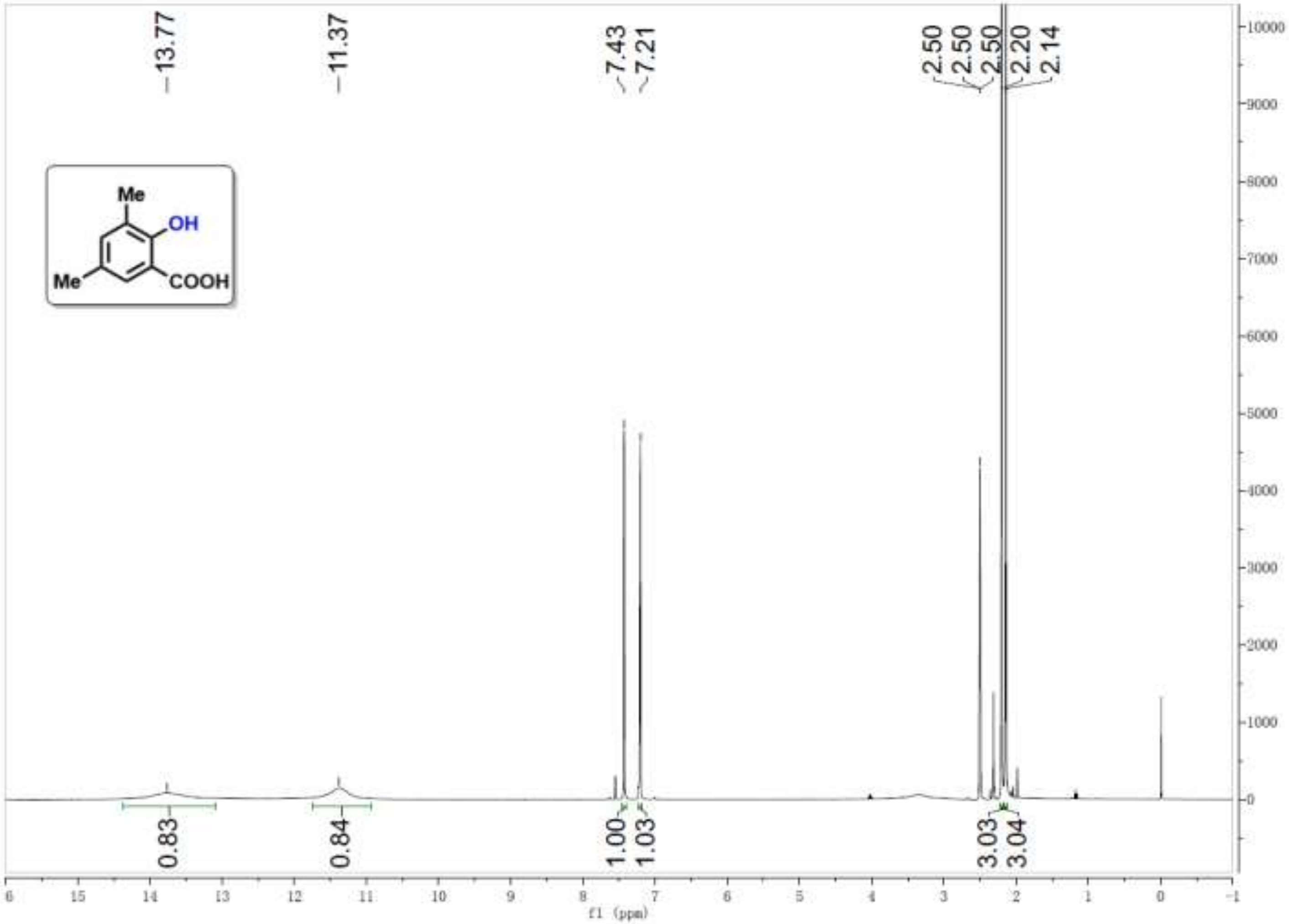

13.77
11.37
7.43
7.21
2.50
2.50
2.50
2.20
2.14
Me
OH
Me
COOH
0.83
0.84
1.00
1.03
3.03
3.04
f1 (ppm)

^{13}C NMR of 2aa

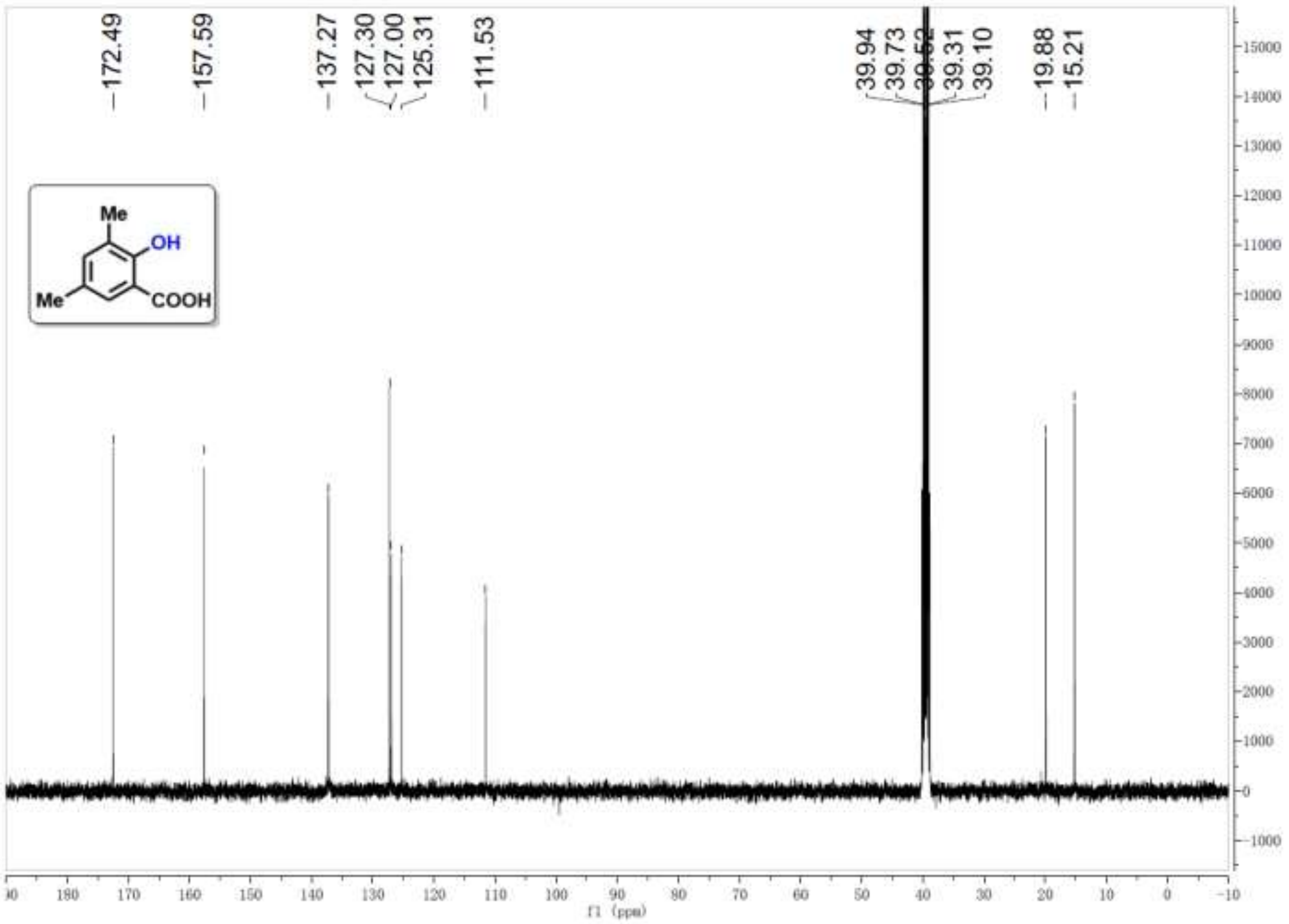

^{1}H NMR of 2ab

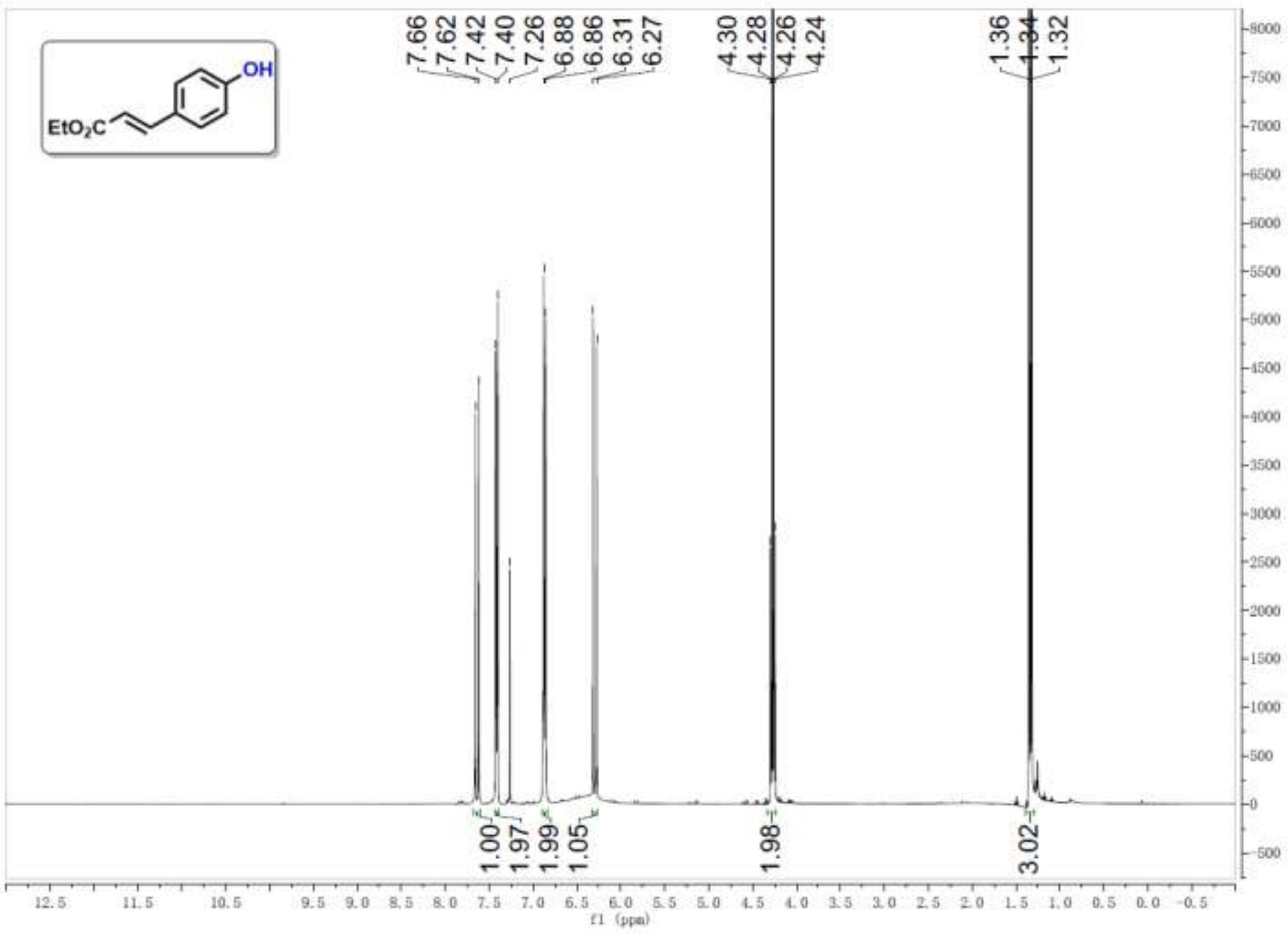

EtO2C
OH
7.66
7.62
7.42
7.40
7.26
6.88
6.86
6.31
6.27
4.30
4.28
4.26
4.24
1.36
1.34
1.32
1.00
1.97
1.99
1.05
1.98
3.02
f1 (ppm)

^{13}C NMR of 2ab

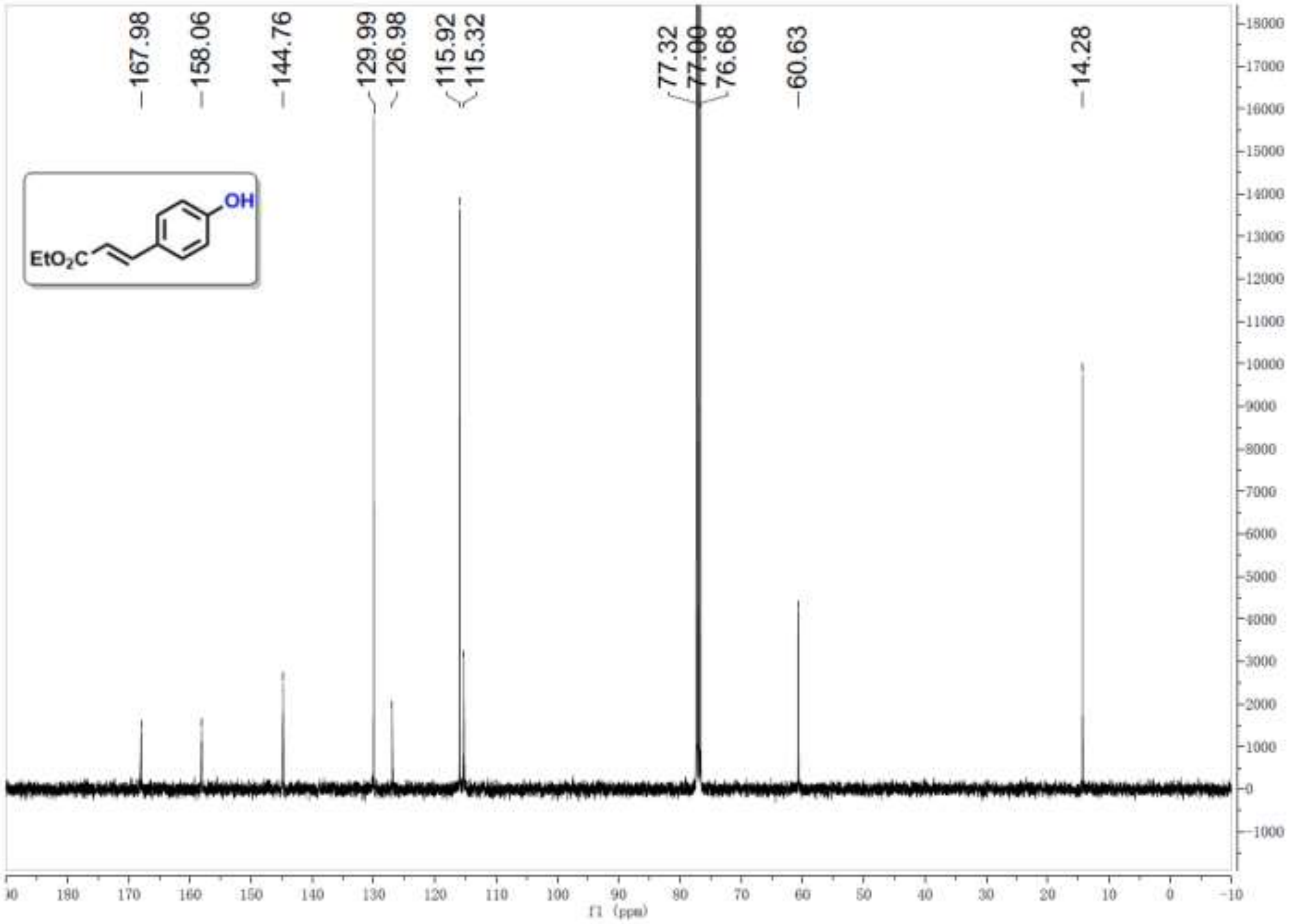

^{1}H NMR of 2ac

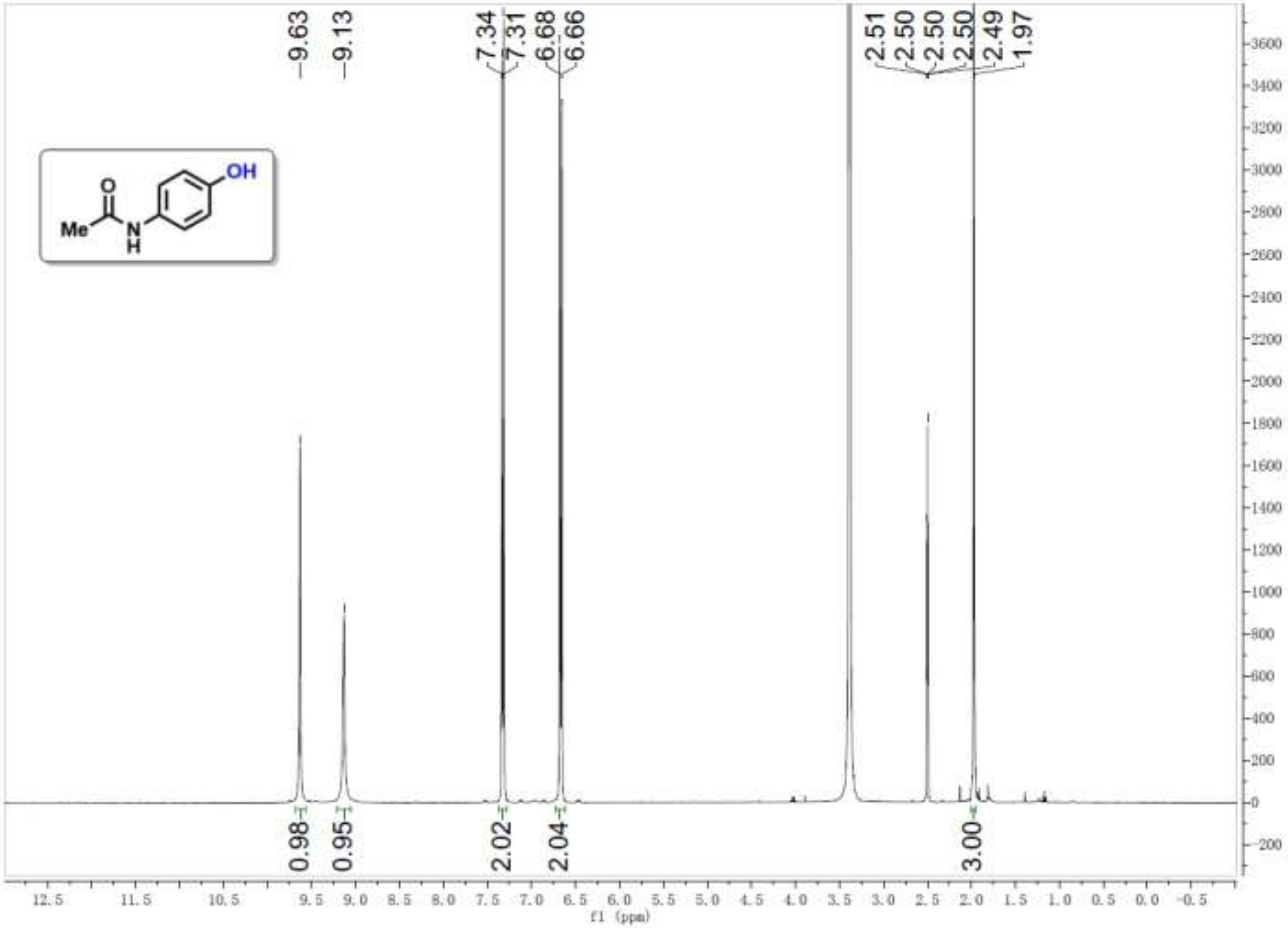

-9.63
-9.13
7.34
7.31
6.68
6.66
2.51
2.50
2.50
2.50
2.49
1.97
OH
O
Me
N
H
0.98
0.95
2.02
2.04
3.00
f1 (ppm)

^{13}C NMR of 2ac

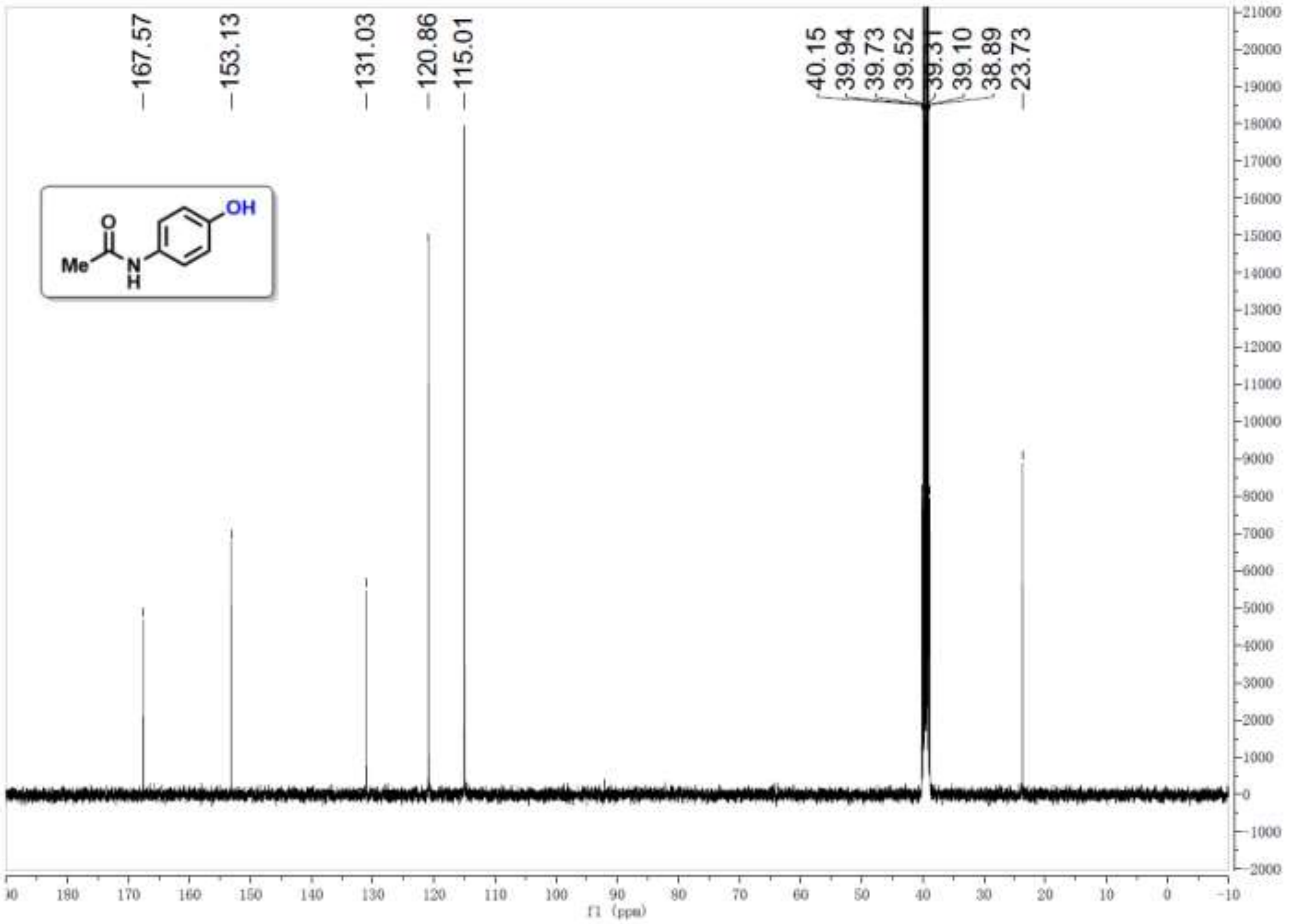

^1H NMR of 2ad

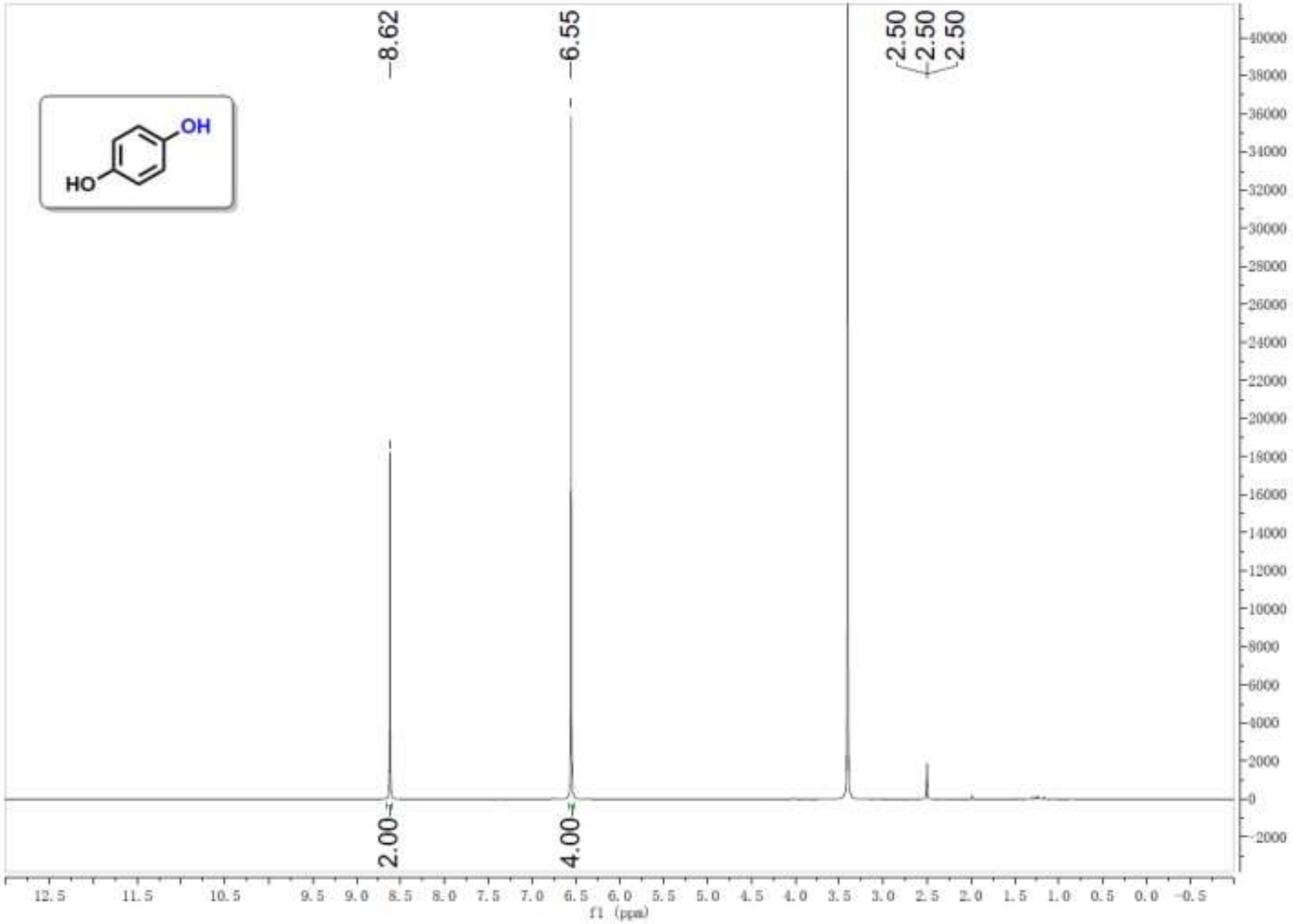

^{13}C NMR of 2ad

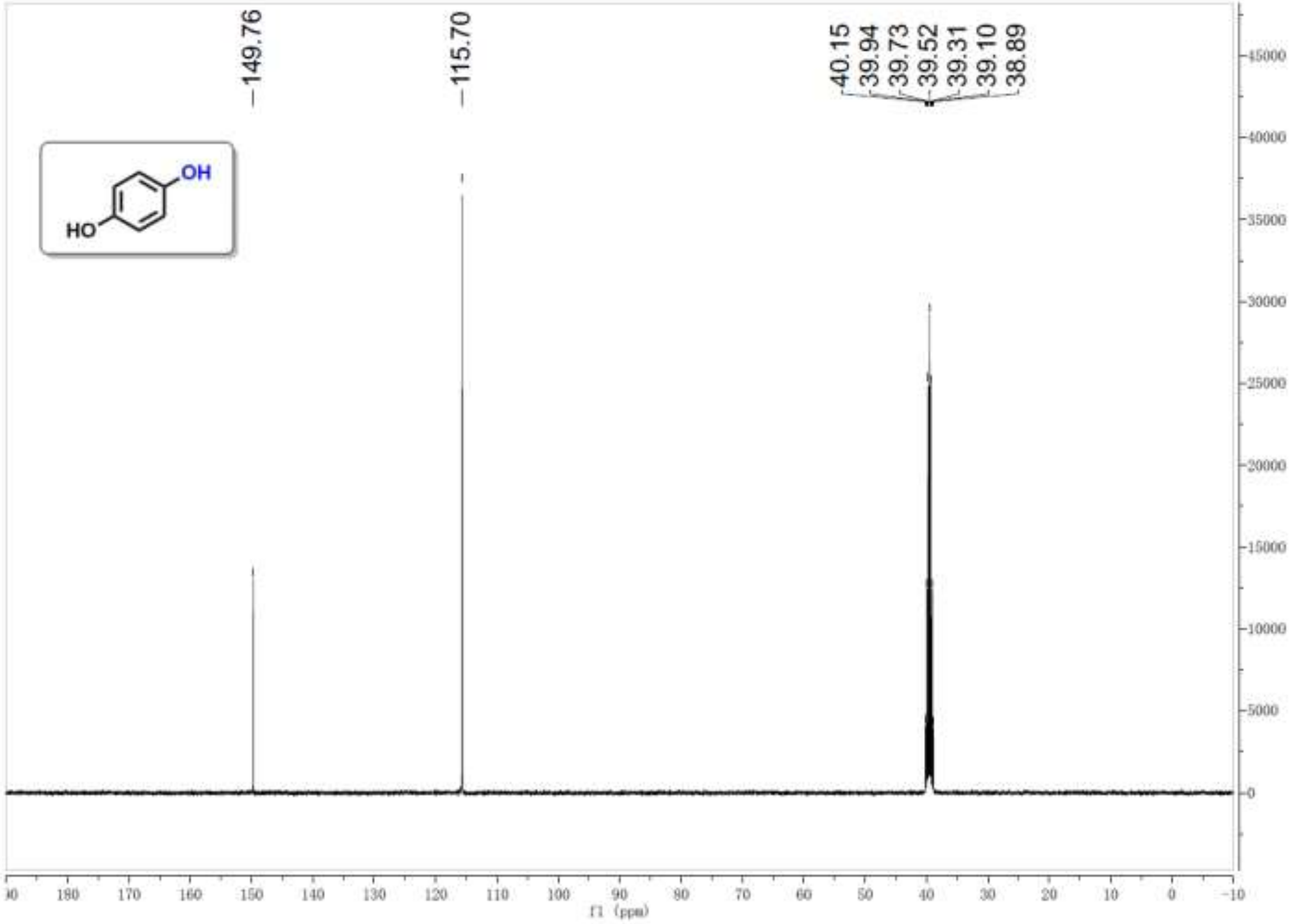

^{1}H NMR of 2ae

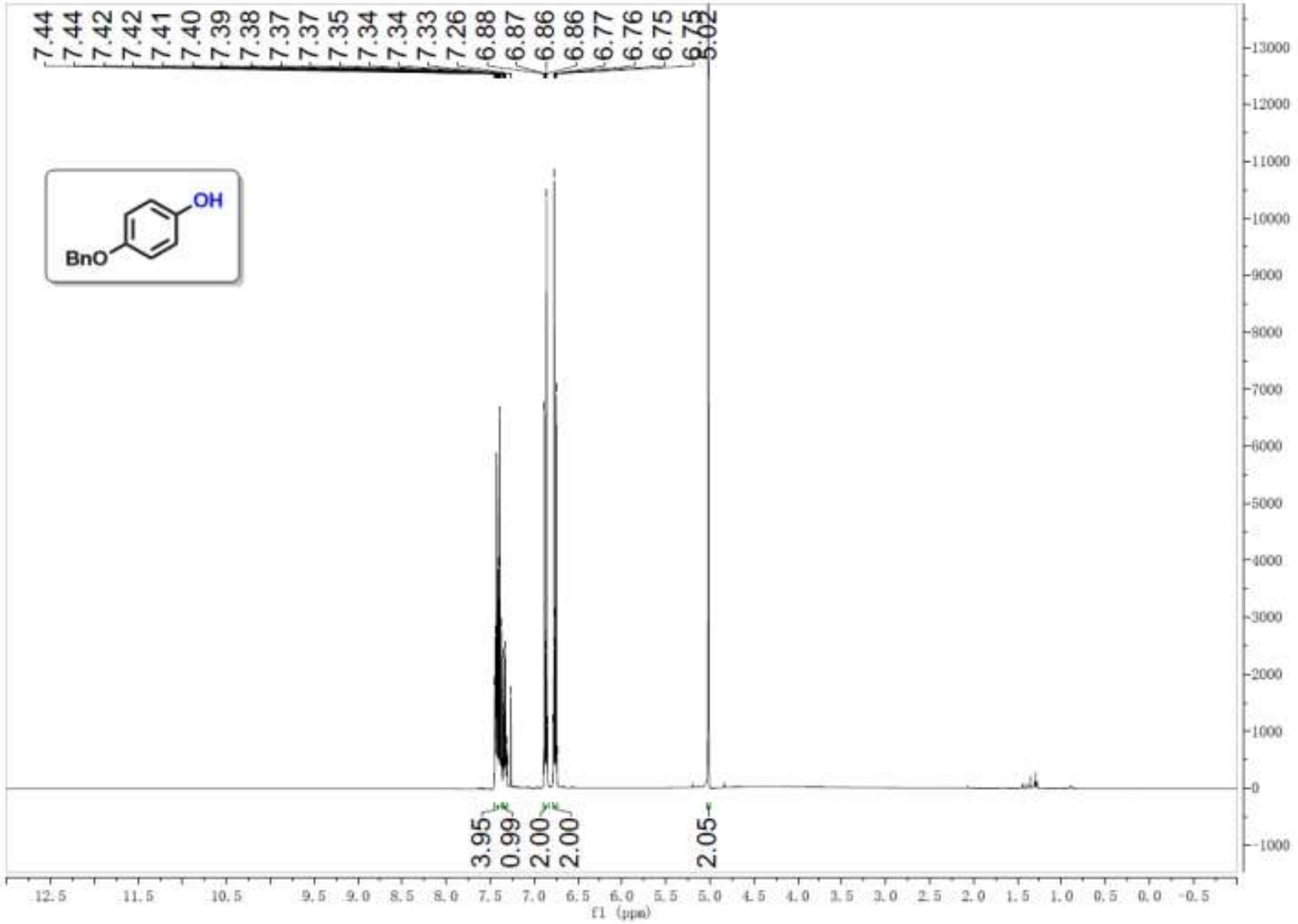

7.44
7.44
7.42
7.42
7.41
7.40
7.39
7.38
7.37
7.37
7.35
7.34
7.34
7.33
7.26
6.88
6.87
6.86
6.86
6.77
6.76
6.75
6.75
5.02
OH
BnO
3.95
0.99
2.00
2.00
2.05
f1 (ppm)

^{13}C NMR of 2ae

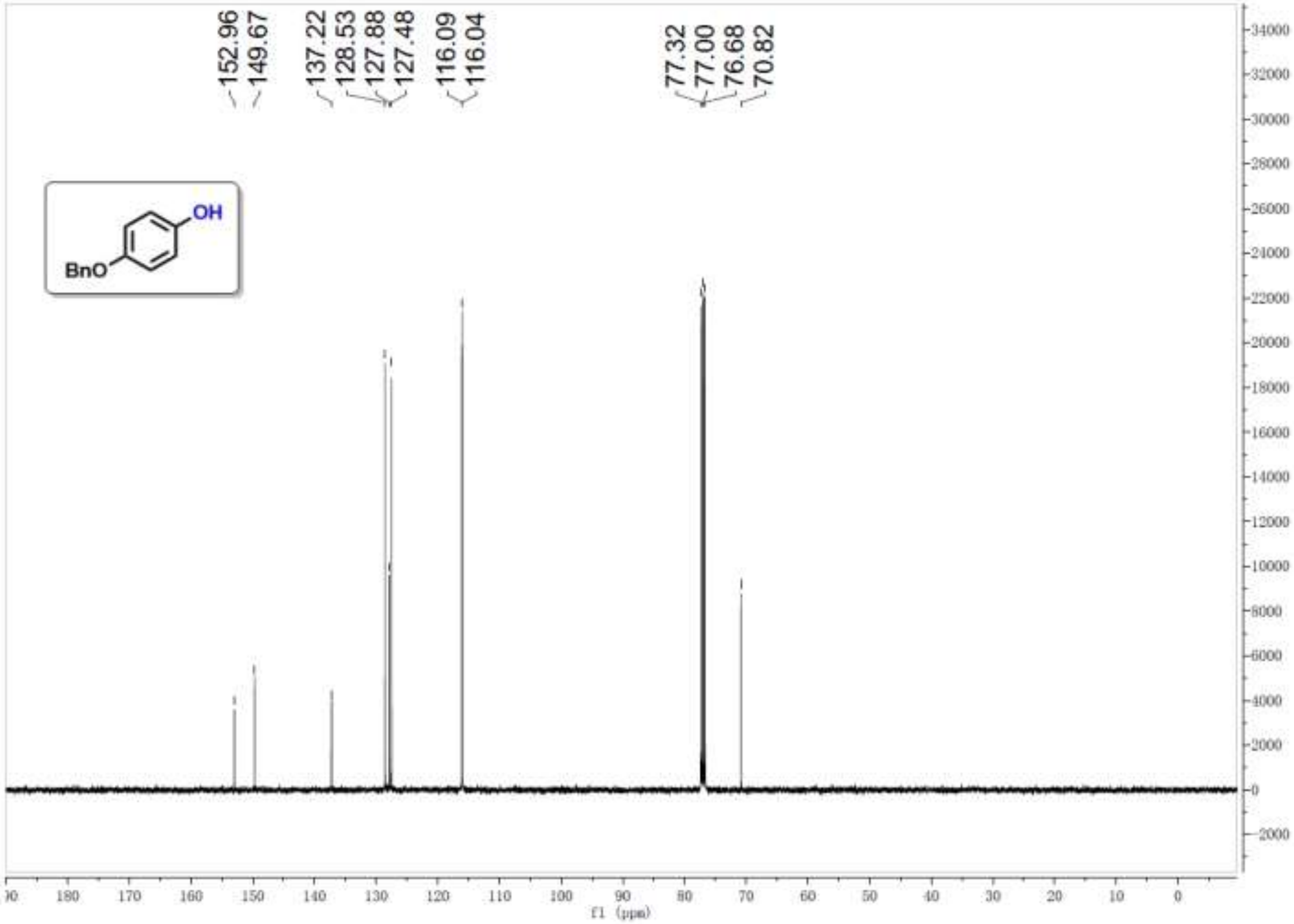

^{1}H NMR of 2af

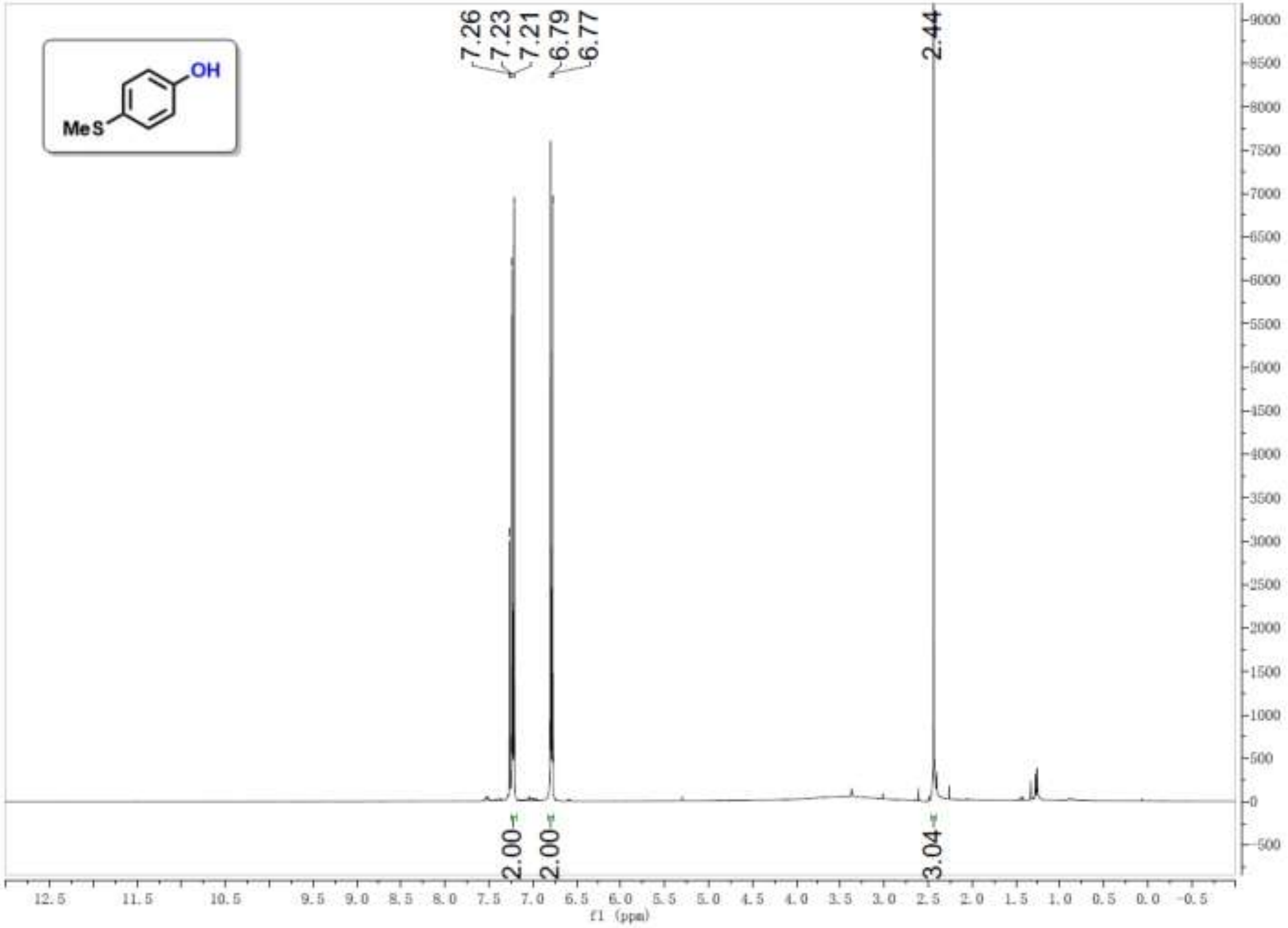

^{13}C NMR of 2af

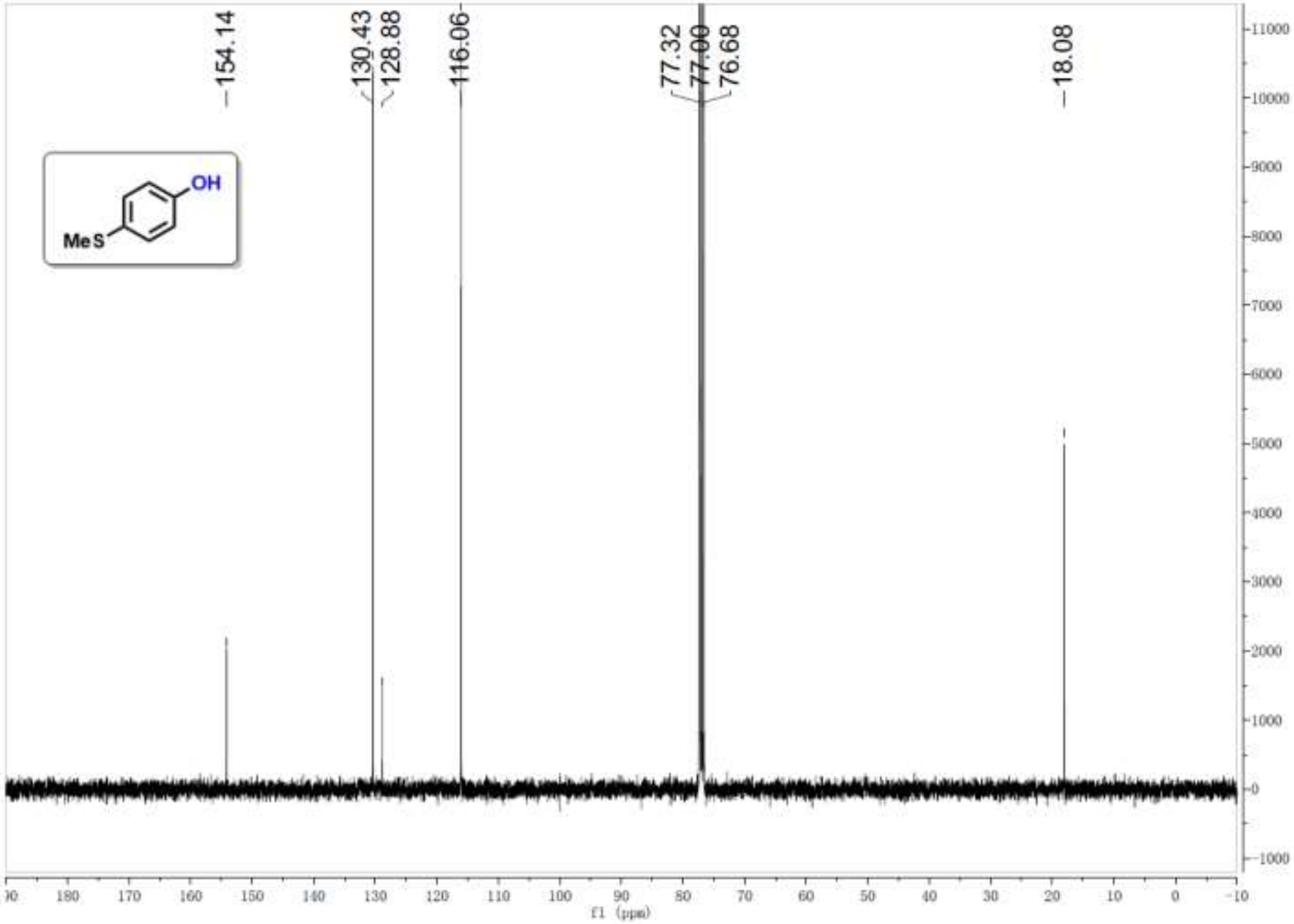

^{1}H NMR of 2ag

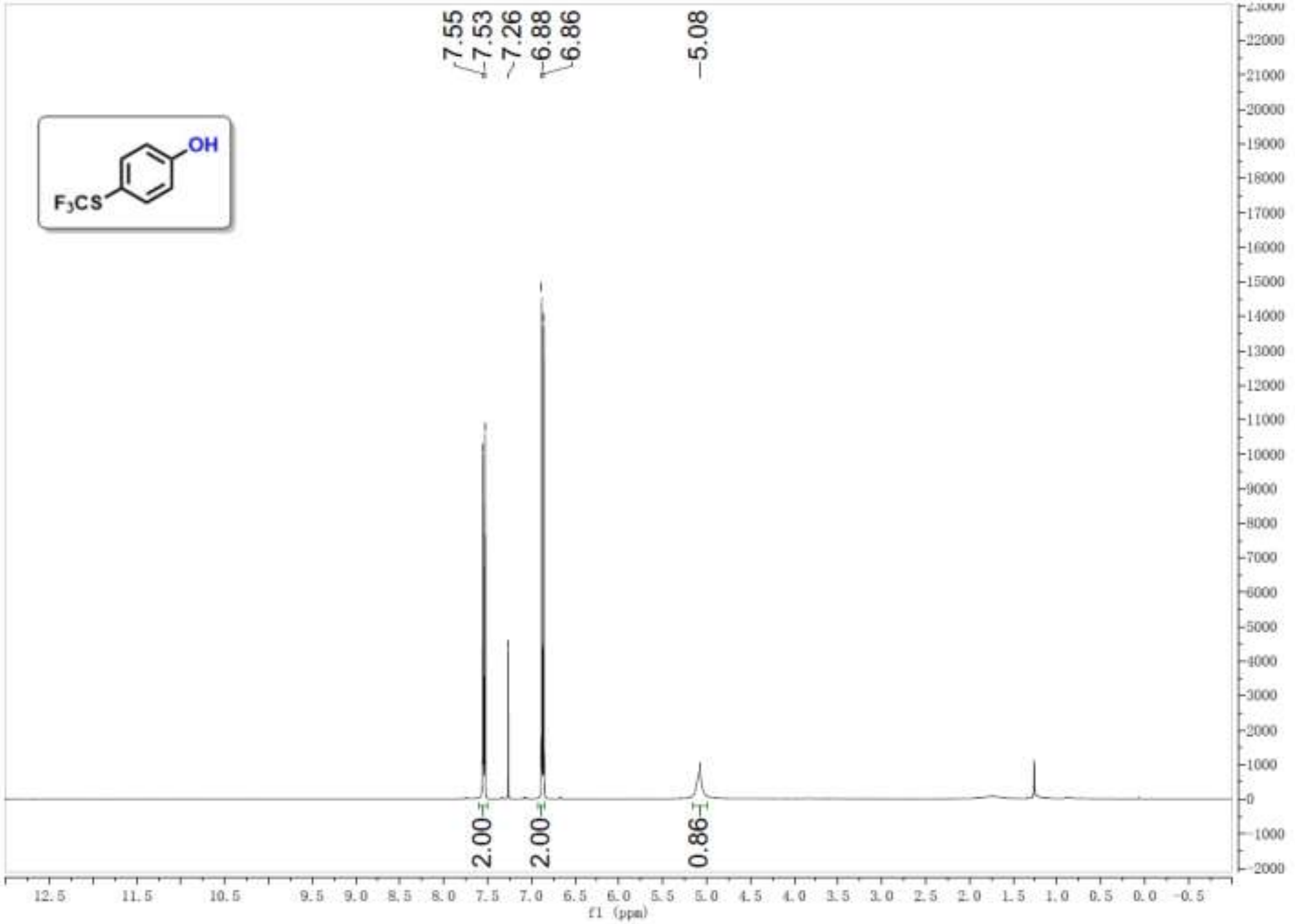

^{13}C NMR of 2ag

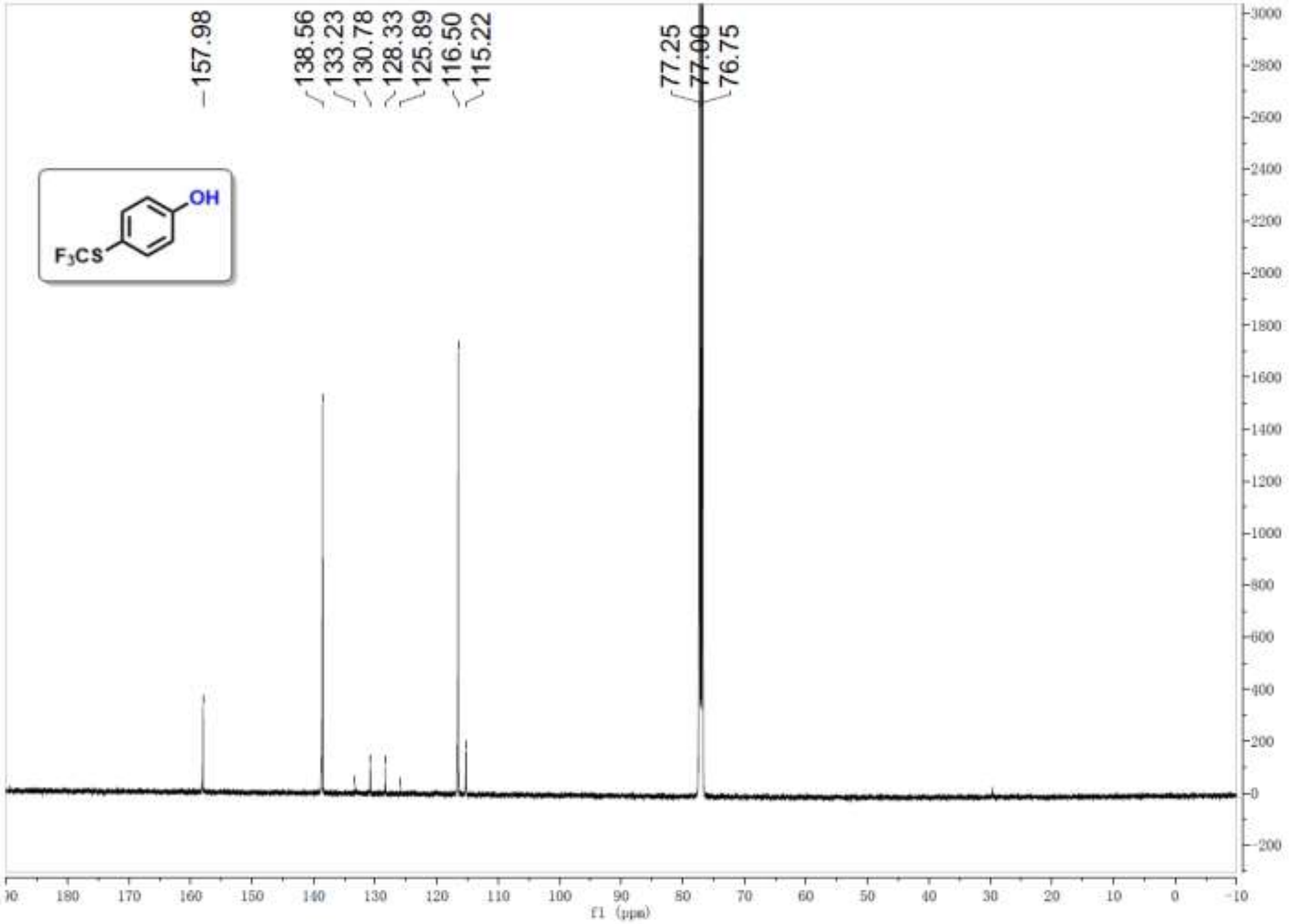

^{19}F NMR of 2ag

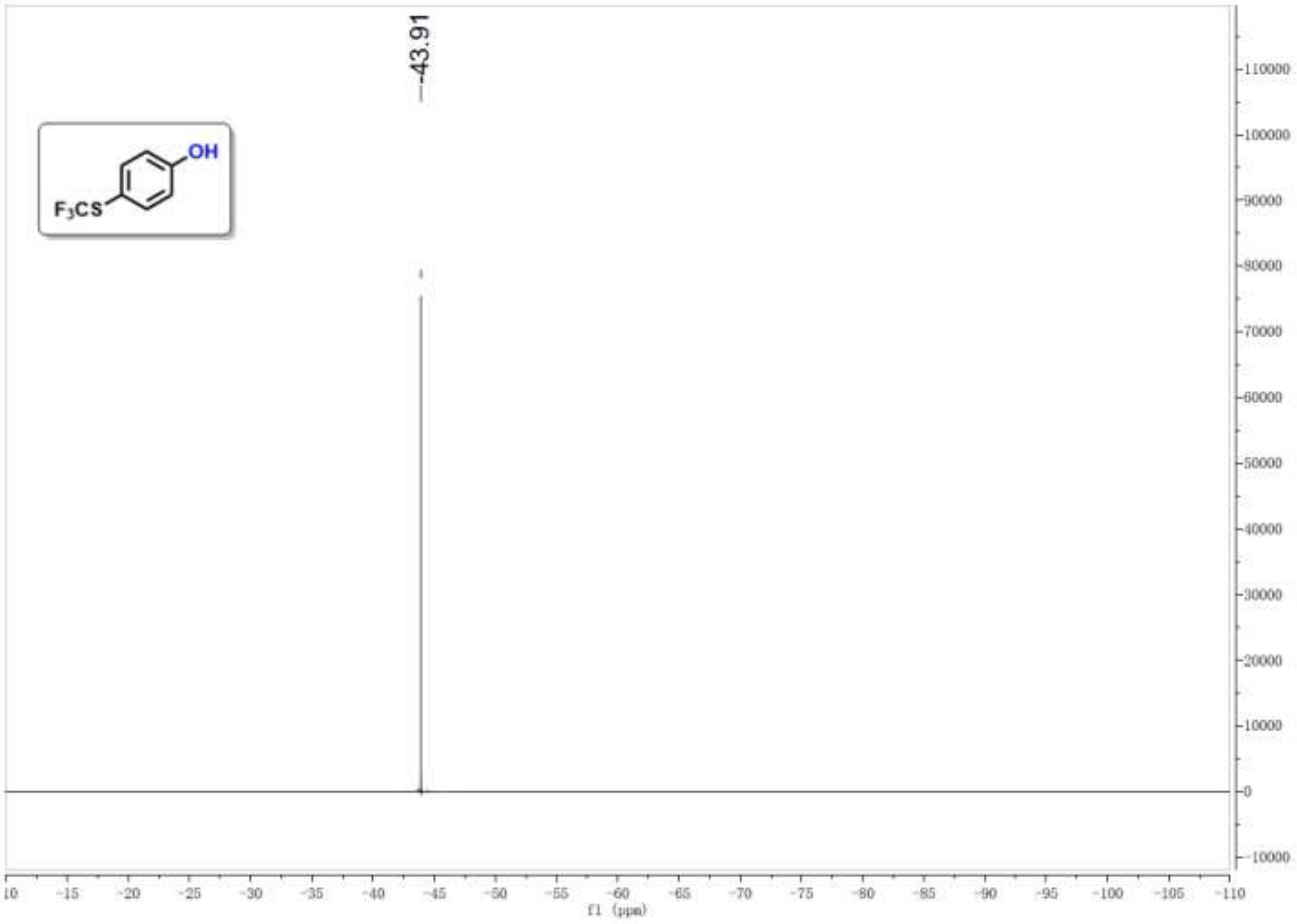

^{1}H NMR of 2ah

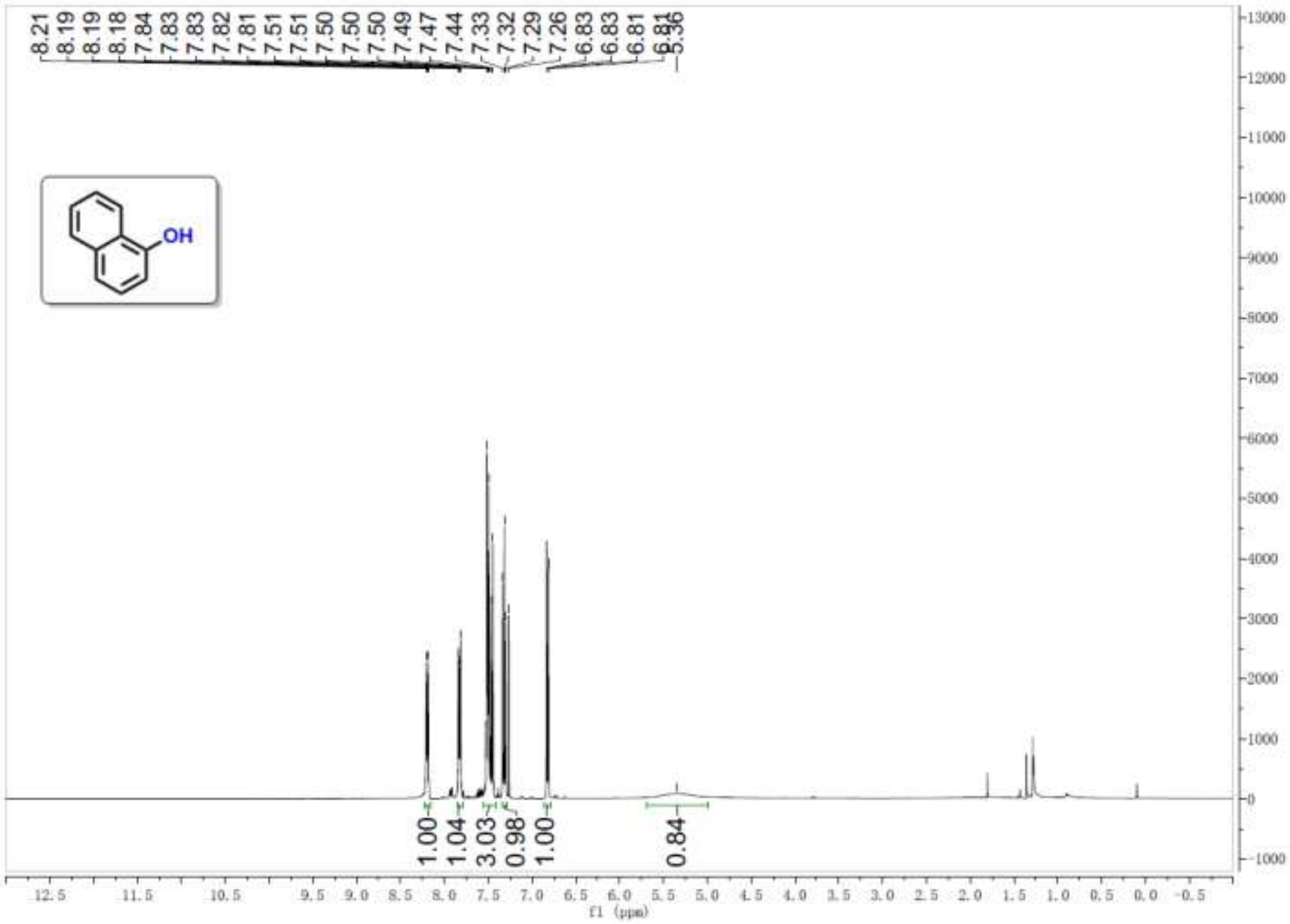

^{13}C NMR of 2ah

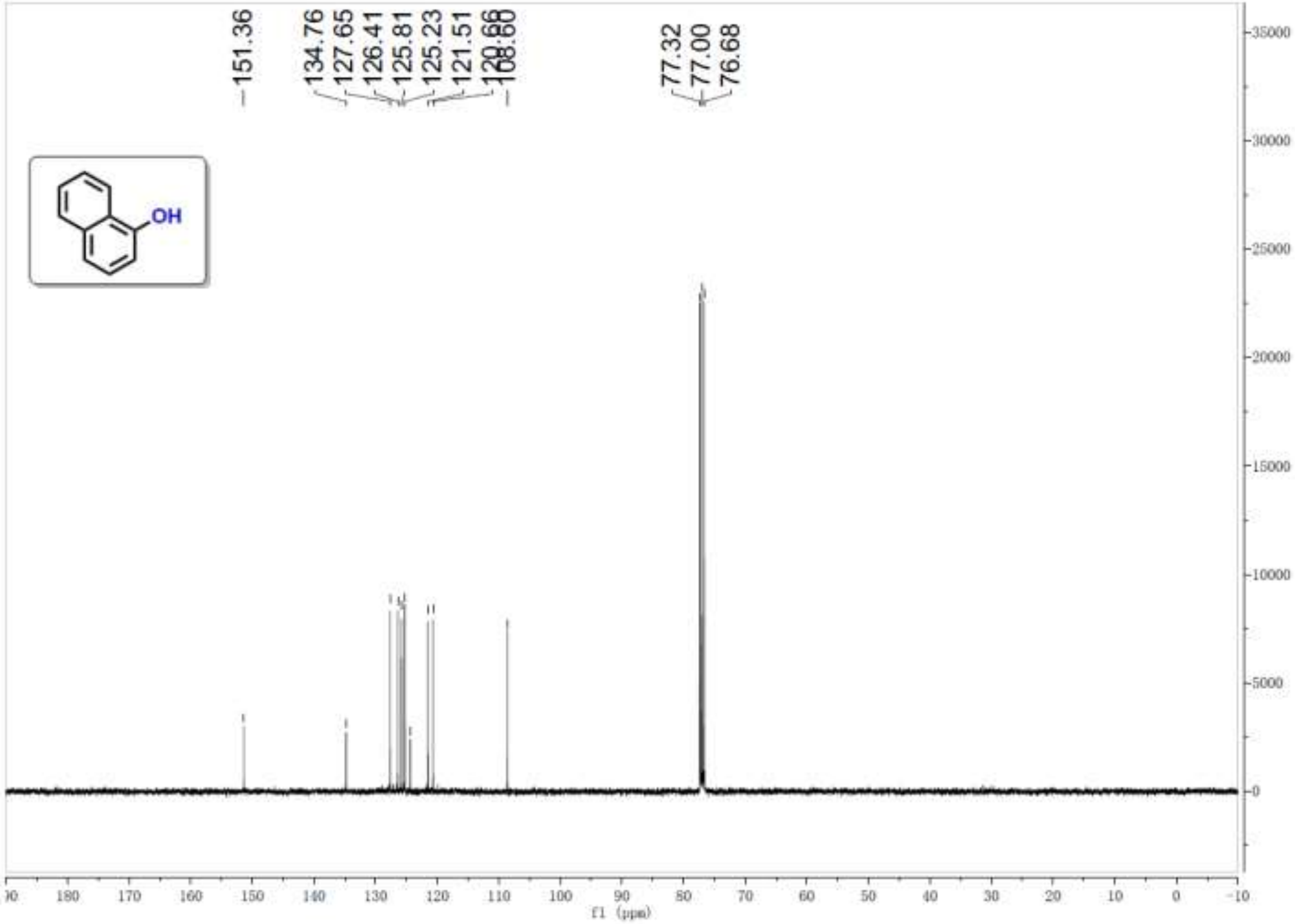

^{1}H NMR of 2ai

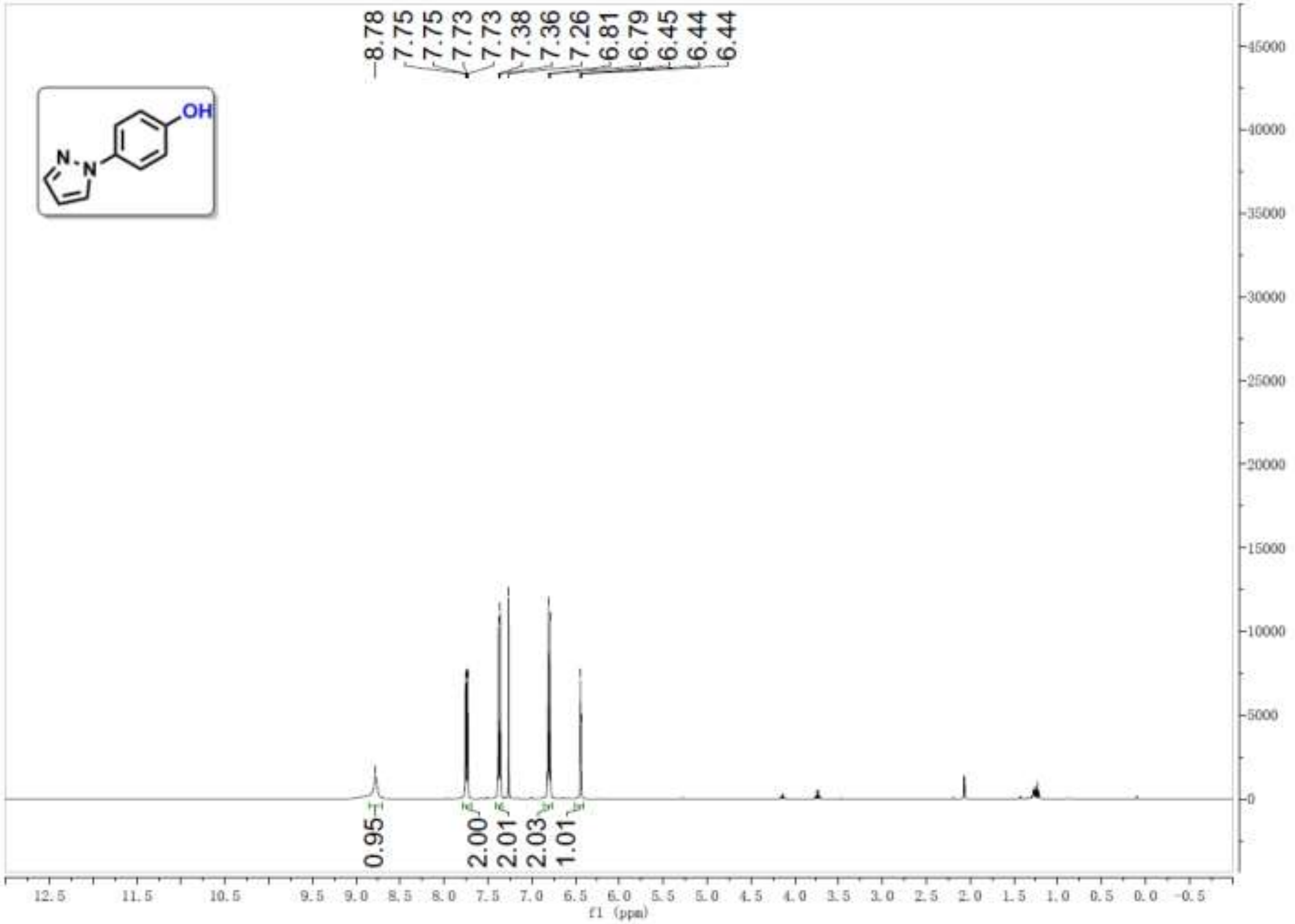

8.78
7.75
7.75
7.73
7.73
7.38
7.36
7.26
6.81
6.79
6.45
6.44
6.44
OH
N
N
0.95
2.00
2.01
2.03
1.01
f1 (ppm)

^{13}C NMR of 2ai

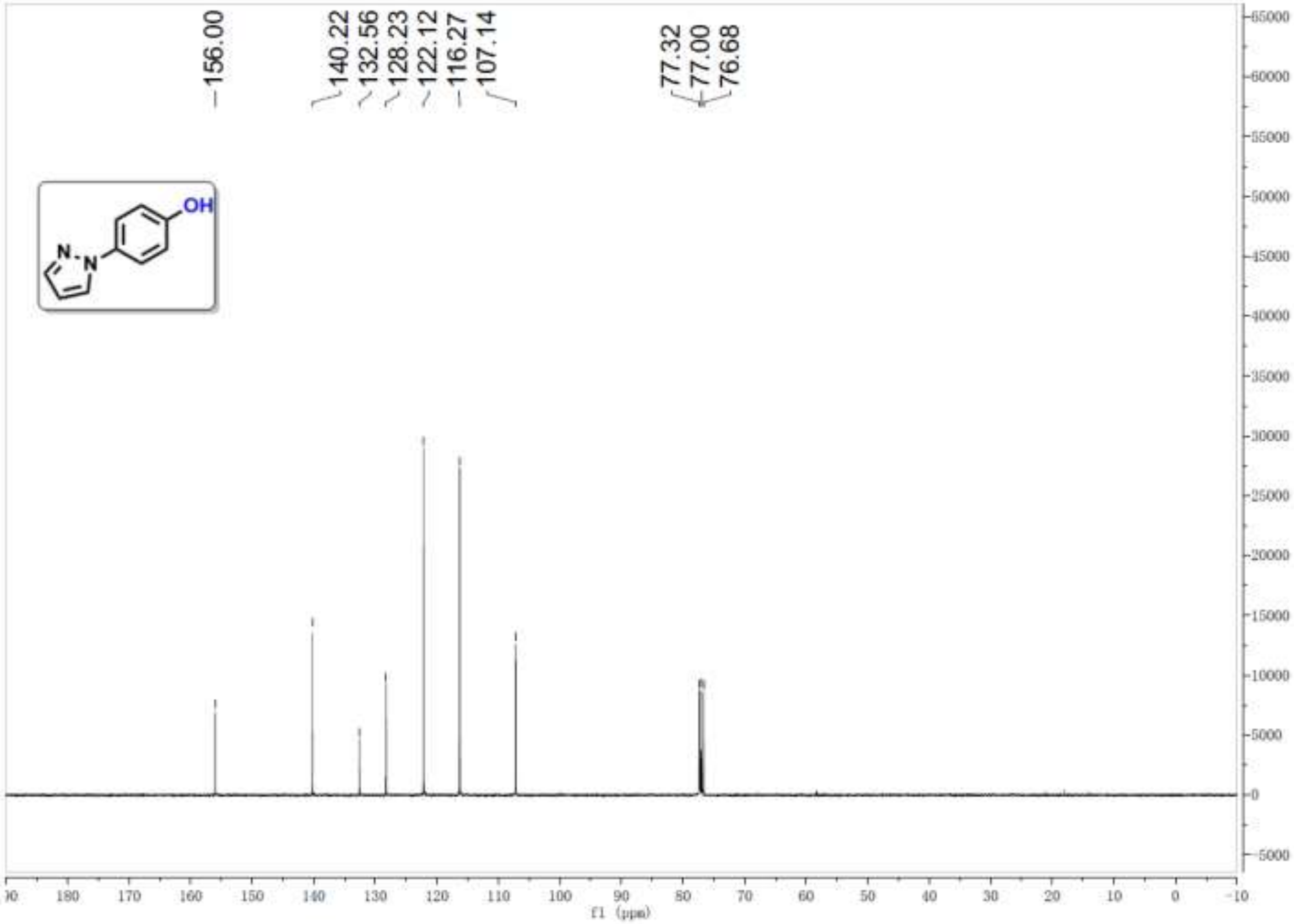

^{1}H NMR of 2aj

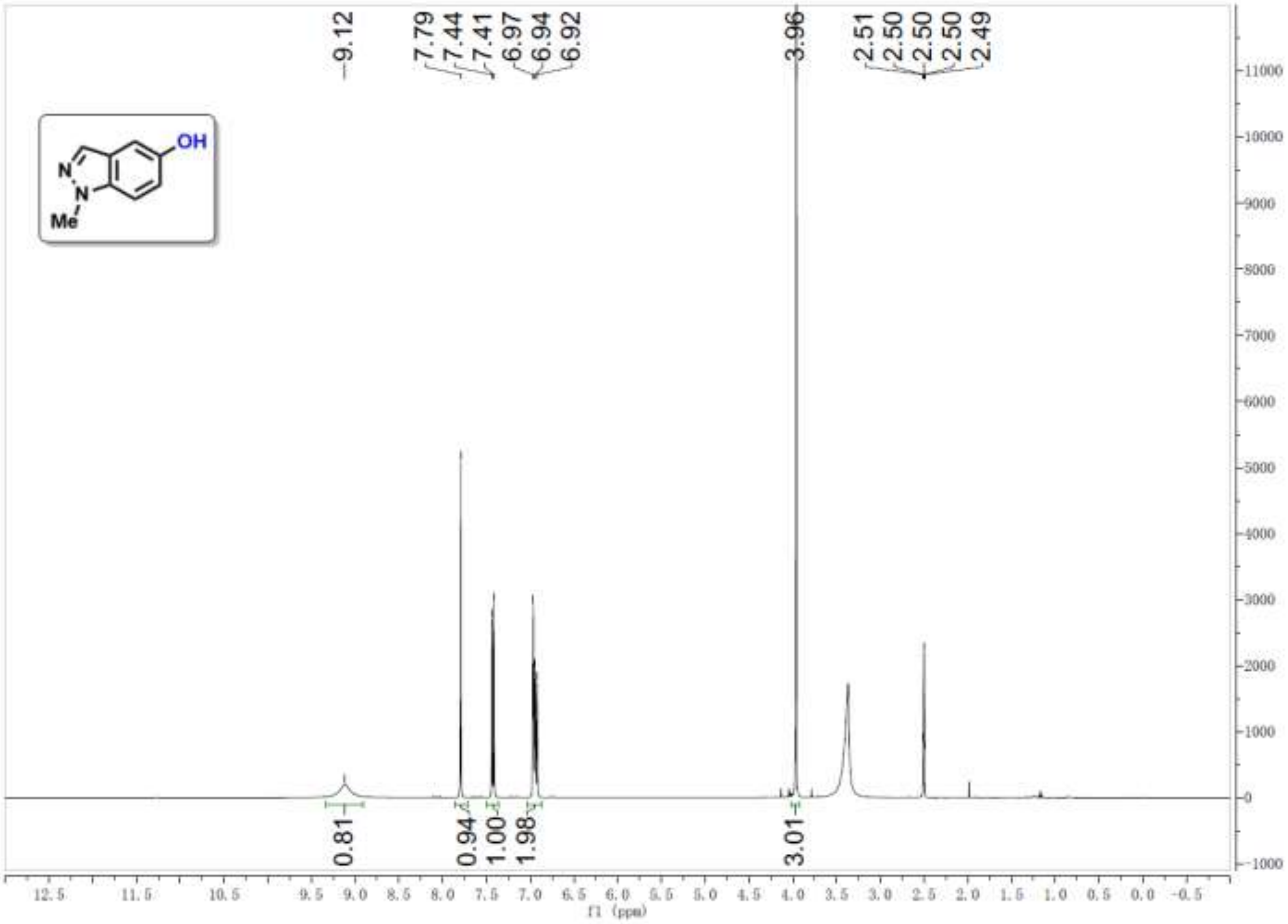

9.12
7.79
7.44
7.41
6.97
6.94
6.92
3.96
2.51
2.50
2.50
2.50
2.49
OH
N
N
Me
0.81
0.94
1.00
1.98
3.01
f1 (ppm)

^{13}C NMR of 2aj

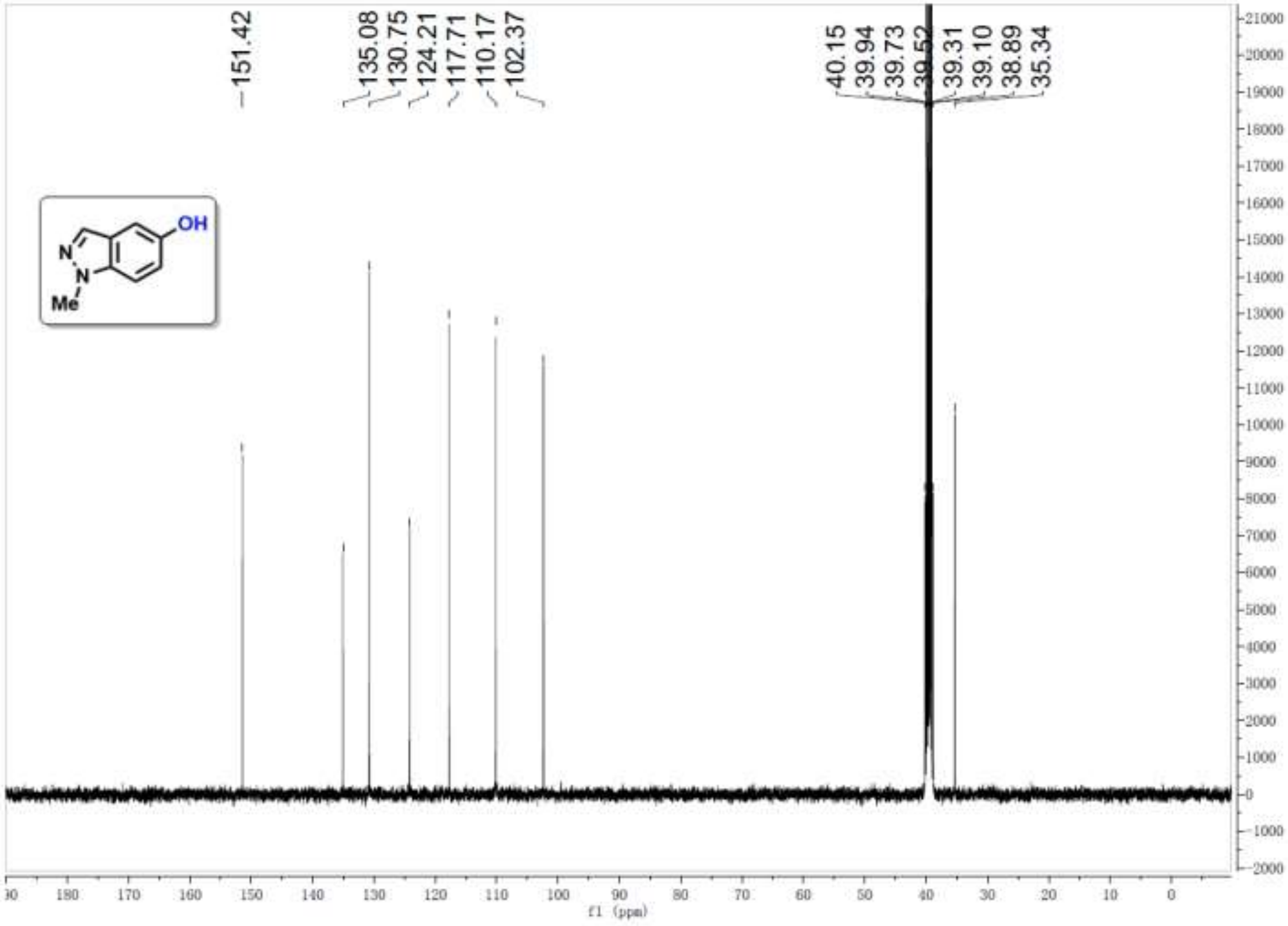

^{1}H NMR of 2ak

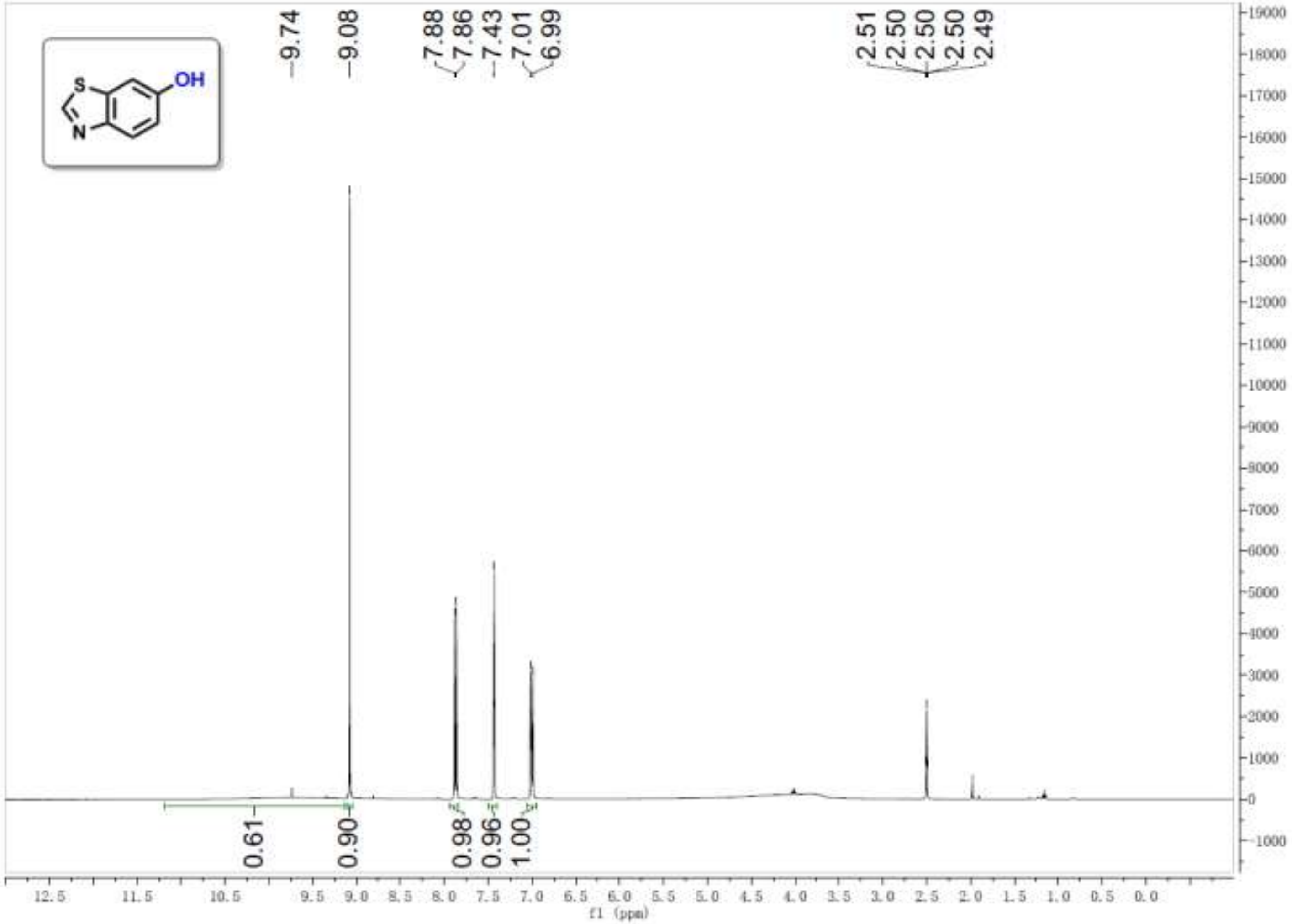

OH
S
N
-9.74
-9.08
7.88
7.86
-7.43
7.01
6.99
2.51
2.50
2.50
2.50
2.49
0.61
0.90
0.98
0.96
1.00
f1 (ppm)

^{13}C NMR of 2ak

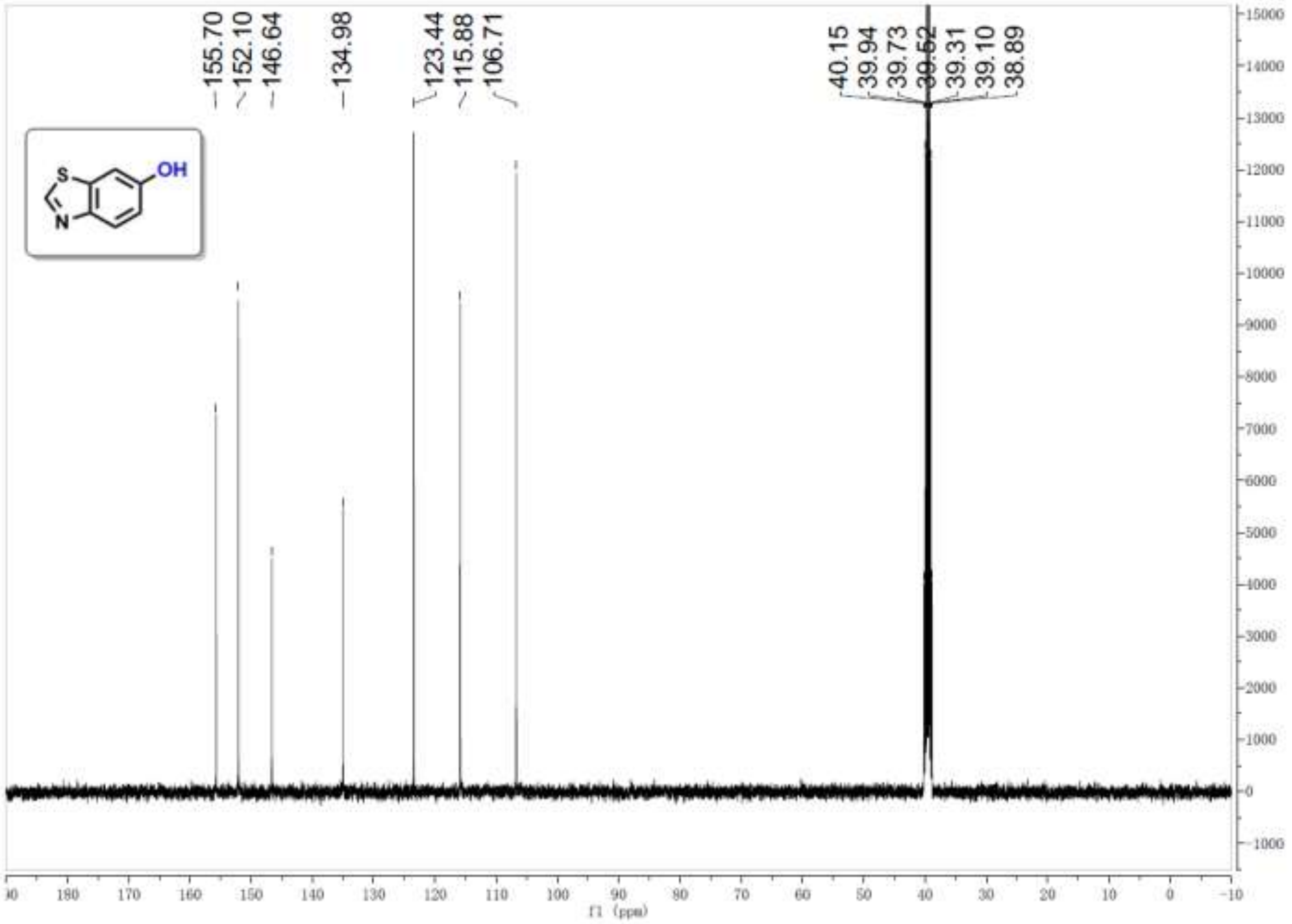

^{1}H NMR of 2al

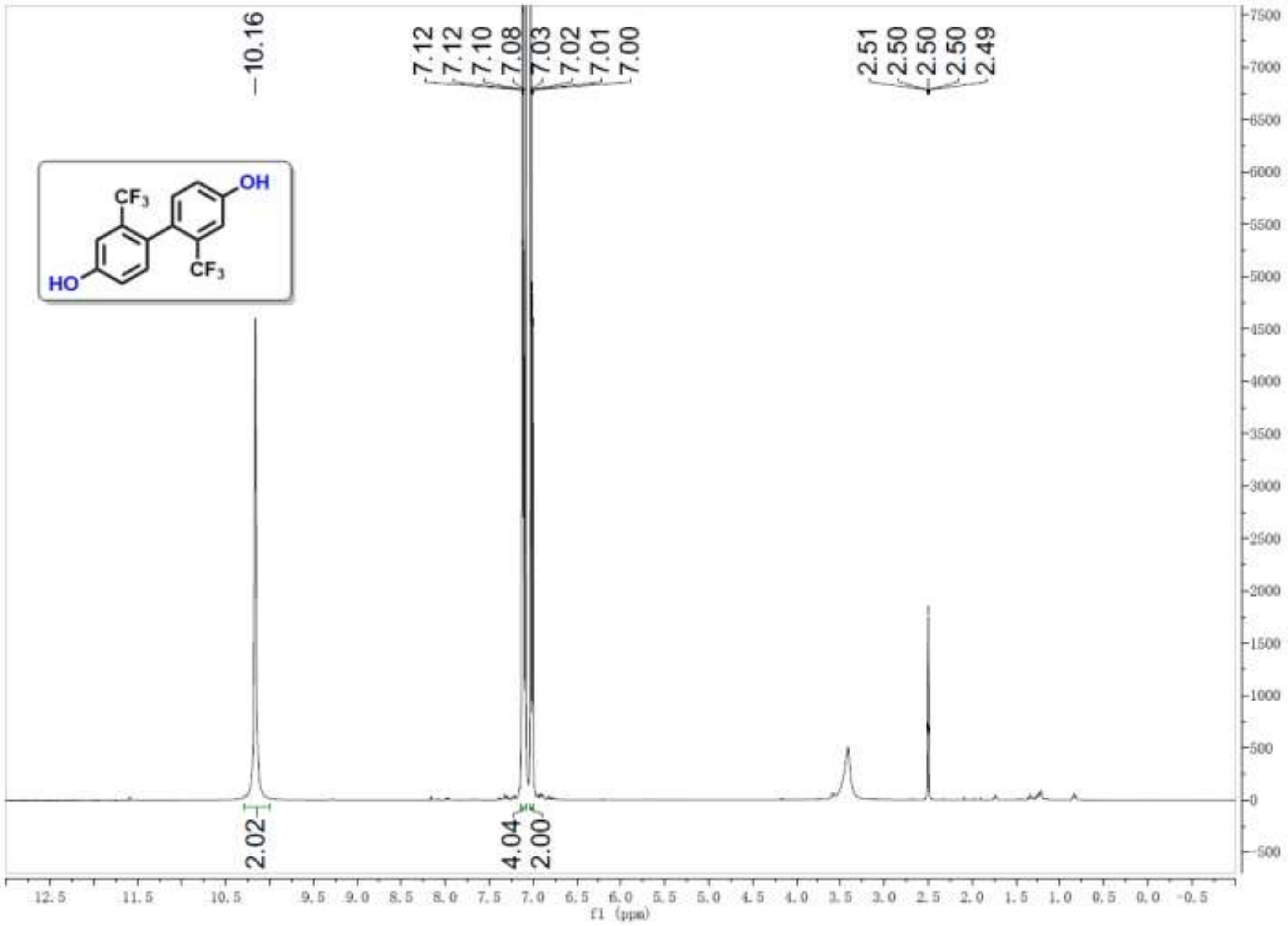

10.16
7.12
7.12
7.10
7.08
7.03
7.02
7.01
7.00
2.51
2.50
2.50
2.50
2.49
CF3
OH
HO
CF3
2.02
4.04
2.00
f1 (ppm)

^{13}C NMR of 2al

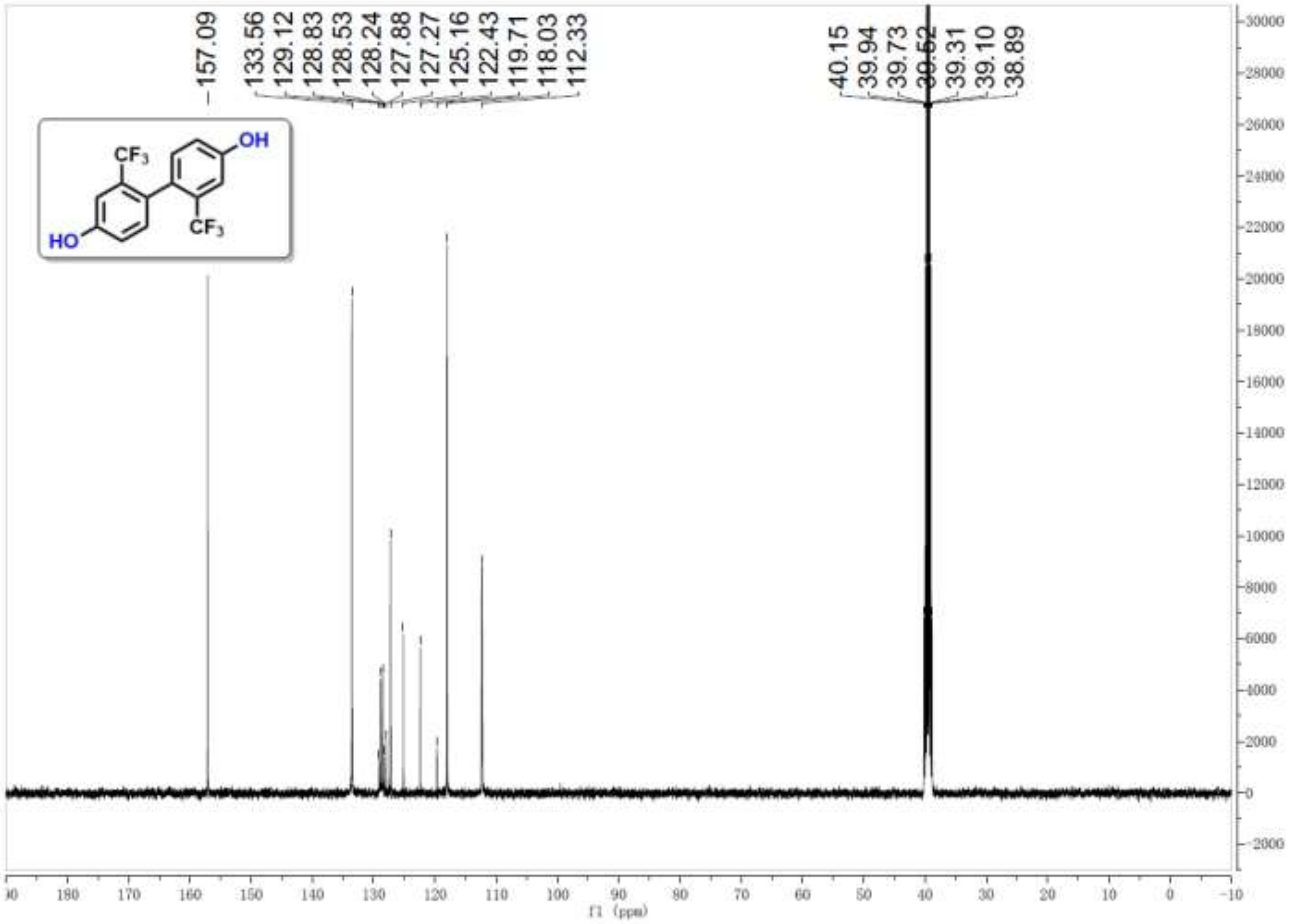

^{19}F NMR of 2al

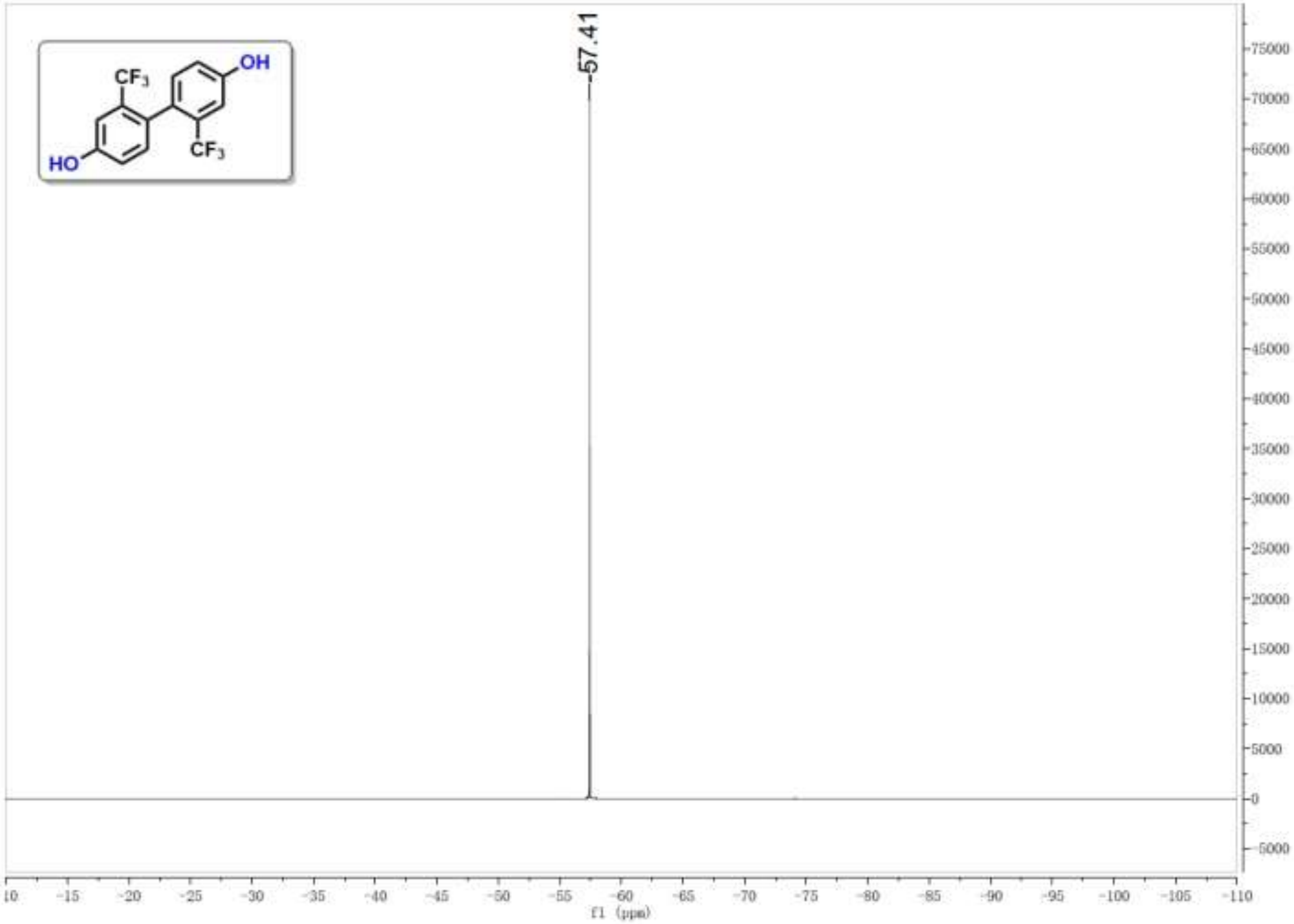

^{1}H NMR of 2am

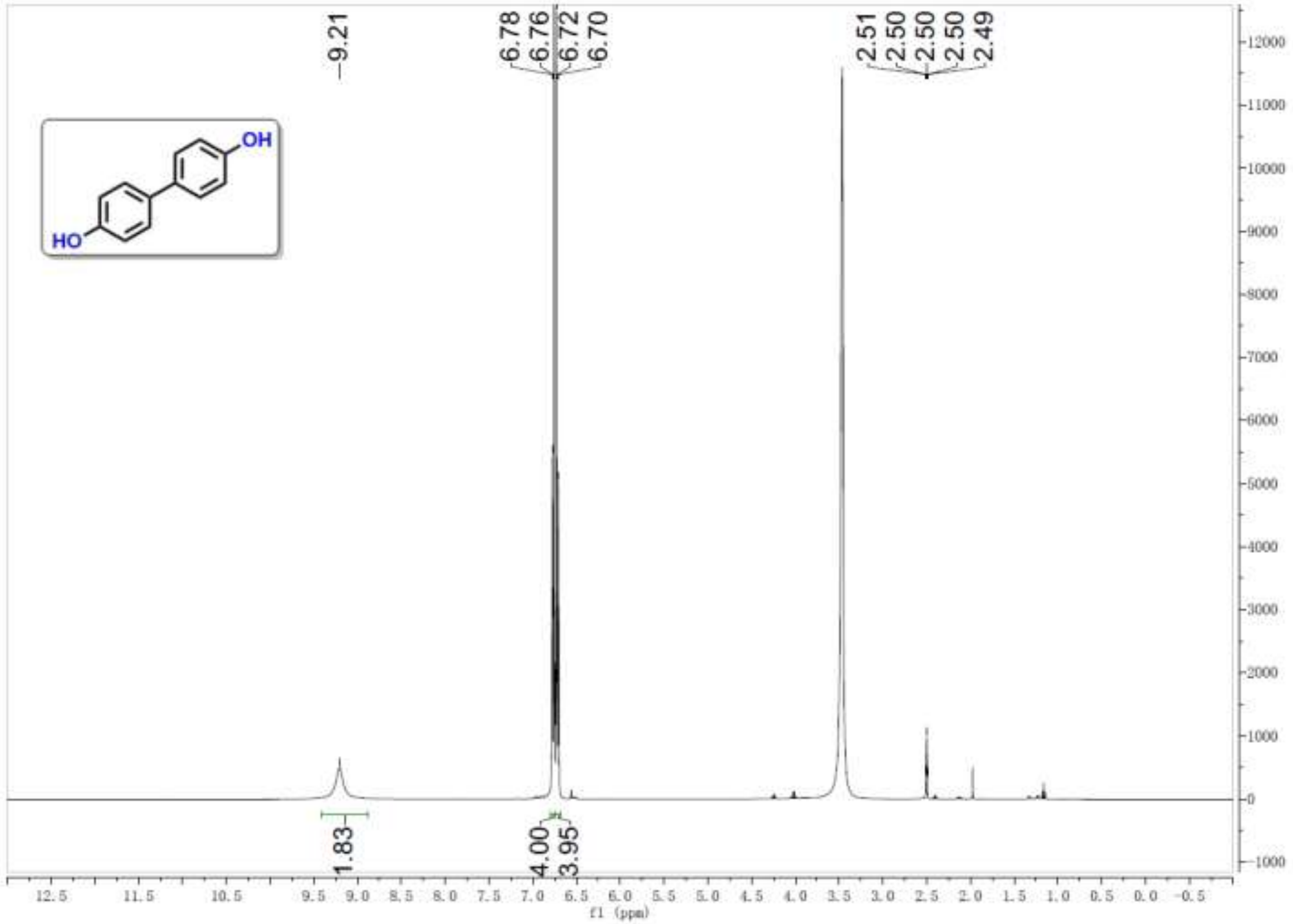

^{13}C NMR of 2am

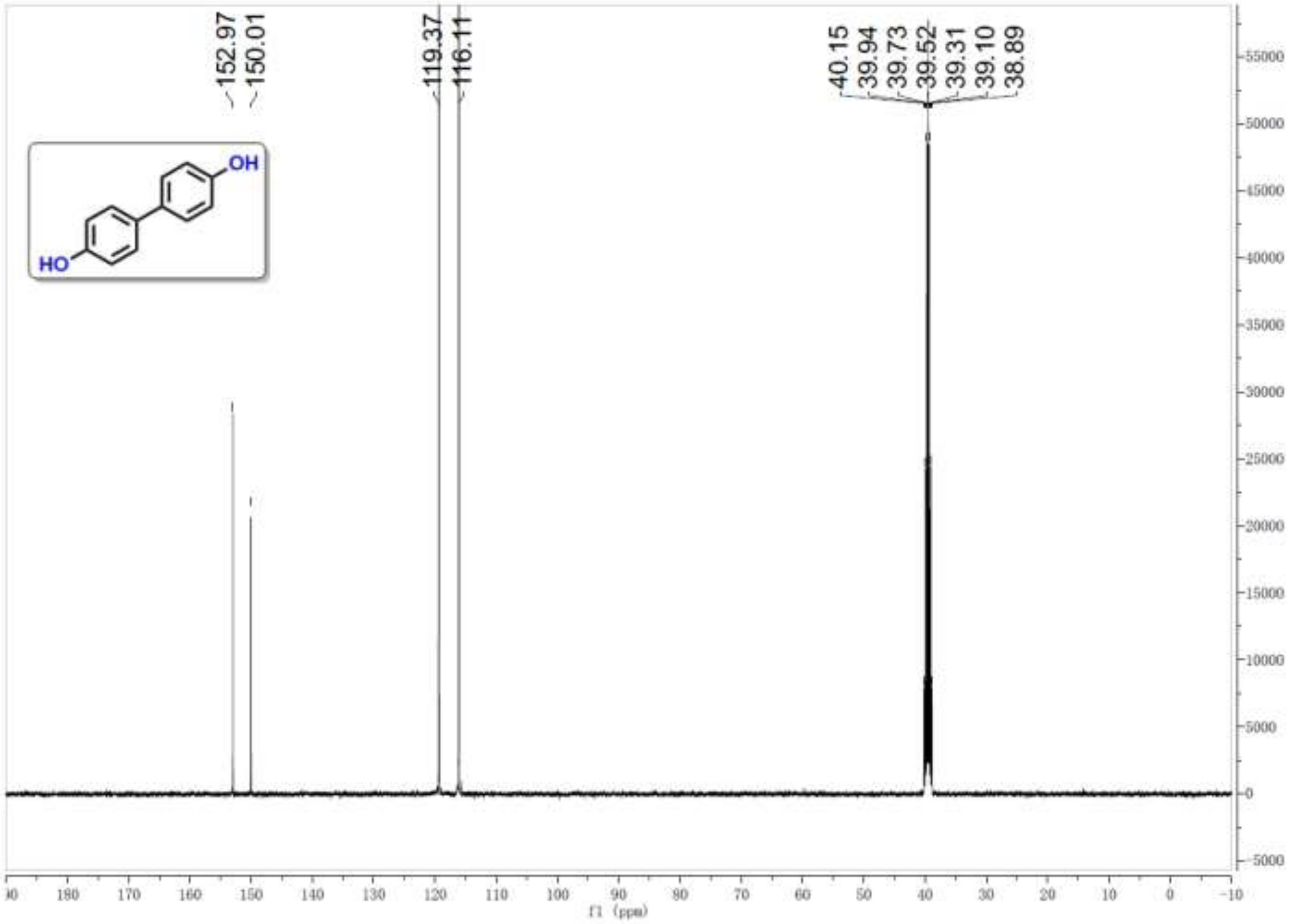

^{1}H NMR of 2an

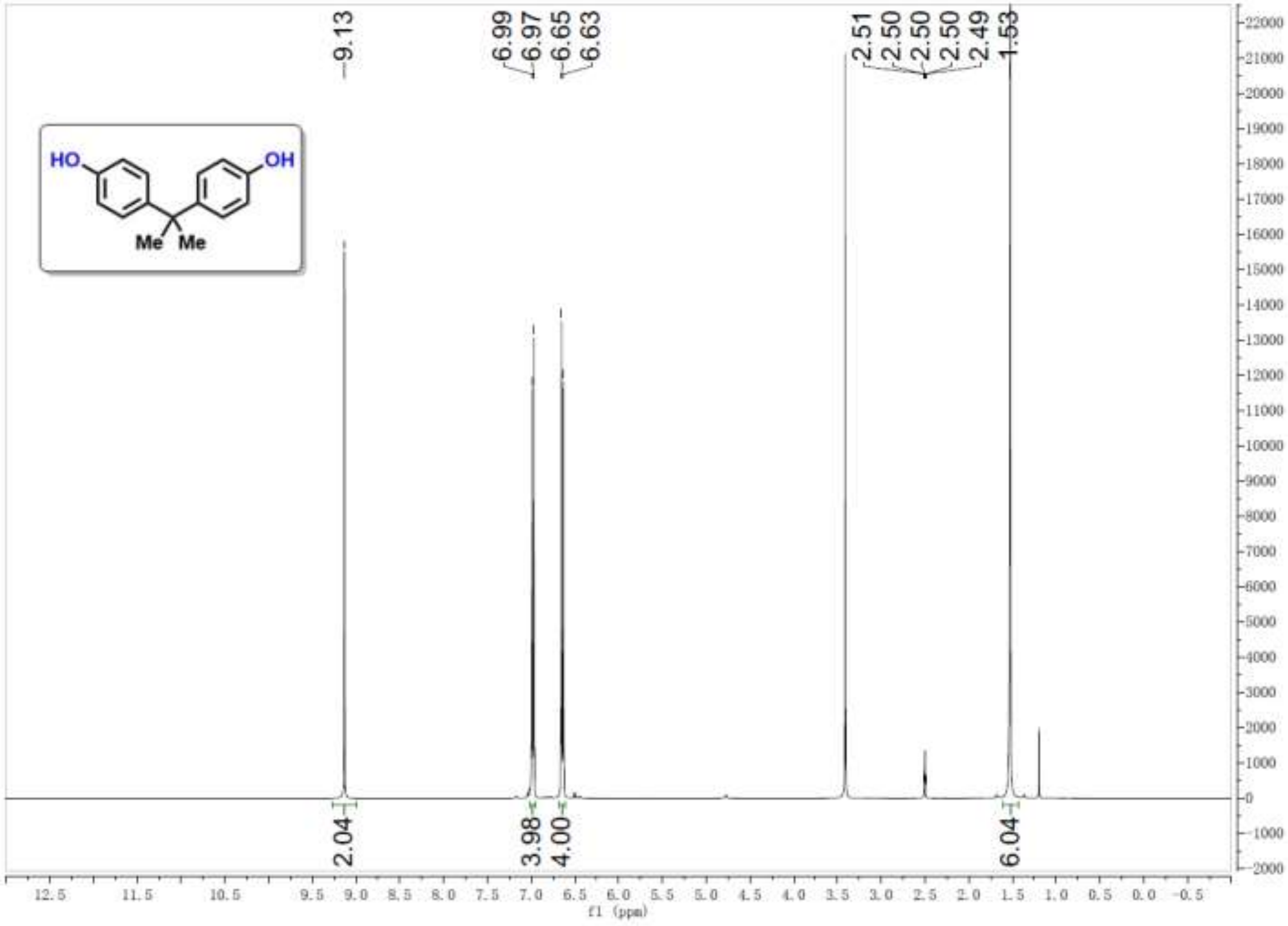

9.13
6.99
6.97
6.65
6.63
2.51
2.50
2.50
2.50
2.49
1.53
HO
OH
Me
Me
2.04
3.98
4.00
6.04
f1 (ppm)

^{13}C NMR of 2an

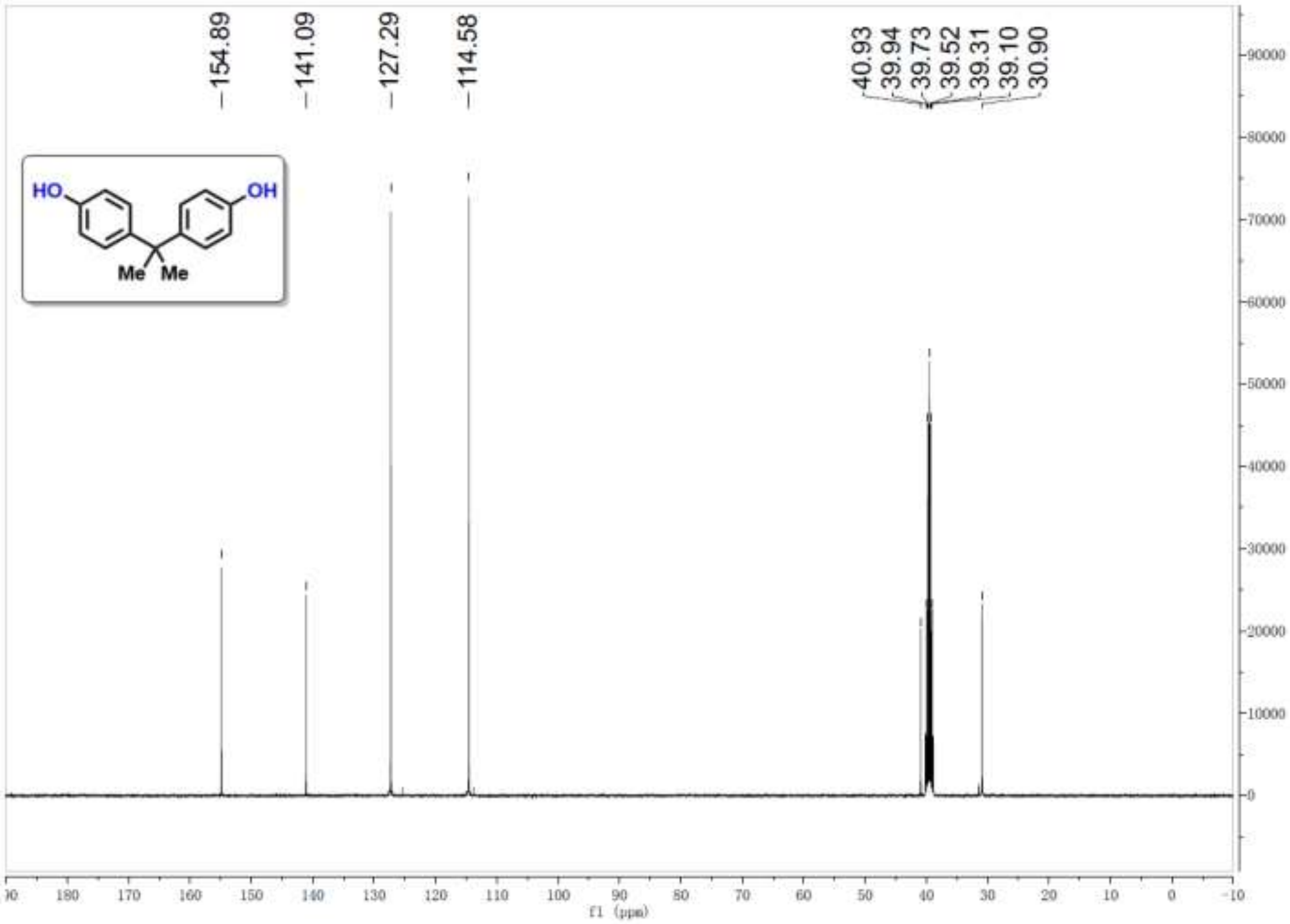

^{1}H NMR of 2ao

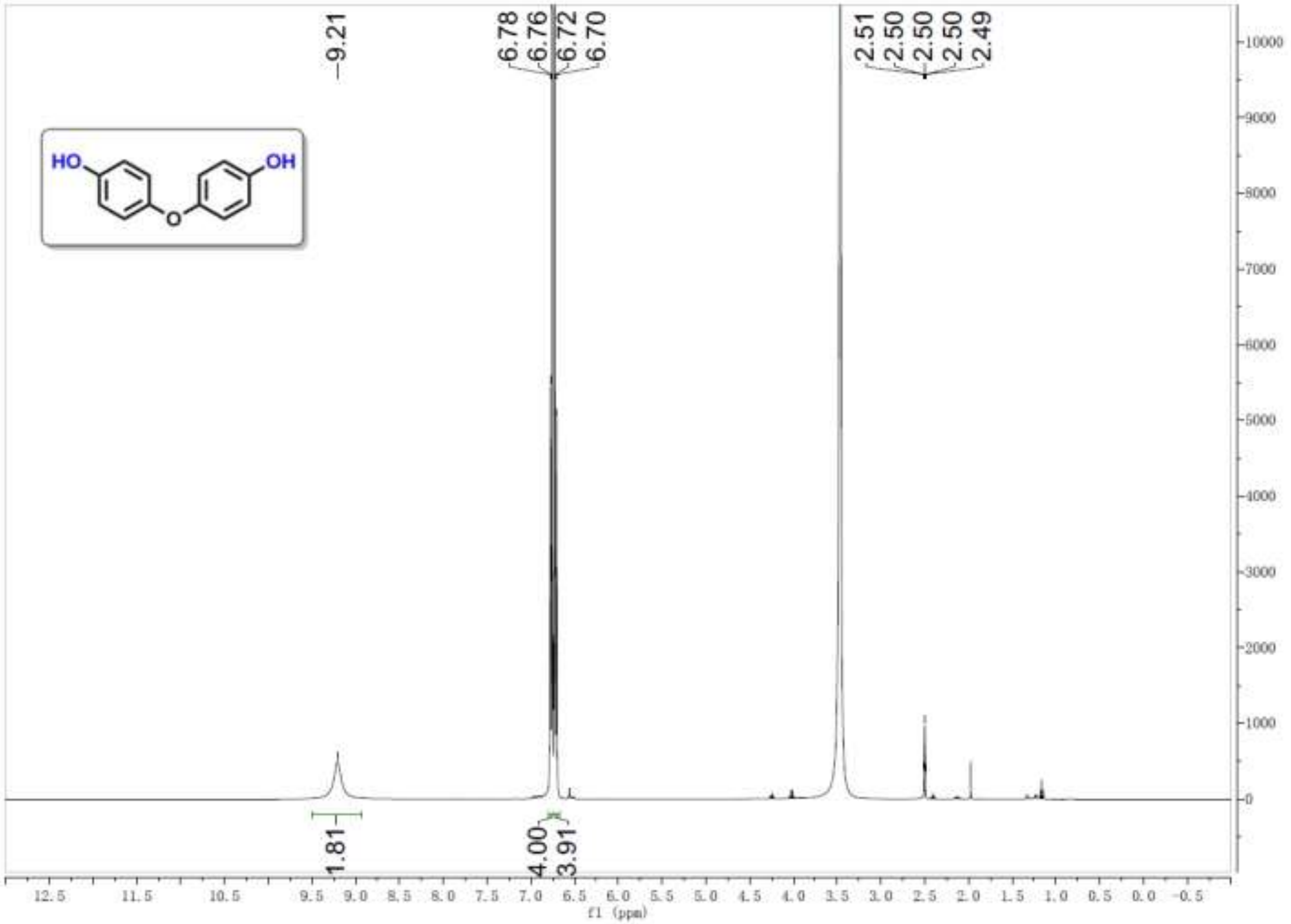

HO
OH
O
-9.21
6.78
6.76
6.72
6.70
2.51
2.50
2.50
2.50
2.49
1.81
4.00
3.91
10000
9000
8000
7000
6000
5000
4000
3000
2000
1000
0
12.5
11.5
10.5
9.5
9.0
8.5
8.0
7.5
7.0
6.5
6.0
5.5
5.0
4.5
4.0
3.5
3.0
2.5
2.0
1.5
1.0
0.5
0.0
-0.5
f1 (ppm)

^{13}C NMR of 2ao

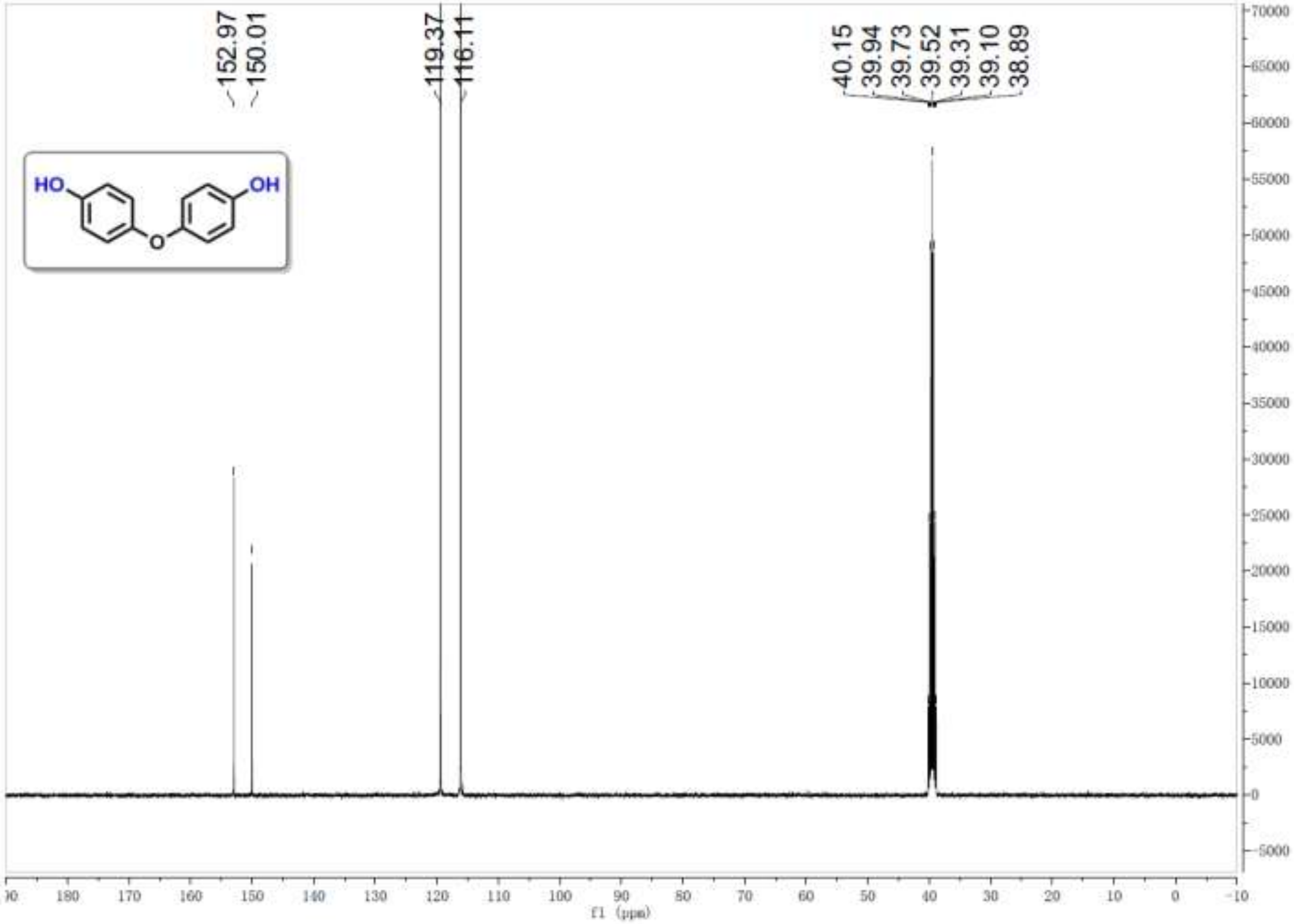

^{1}H NMR of 2ap

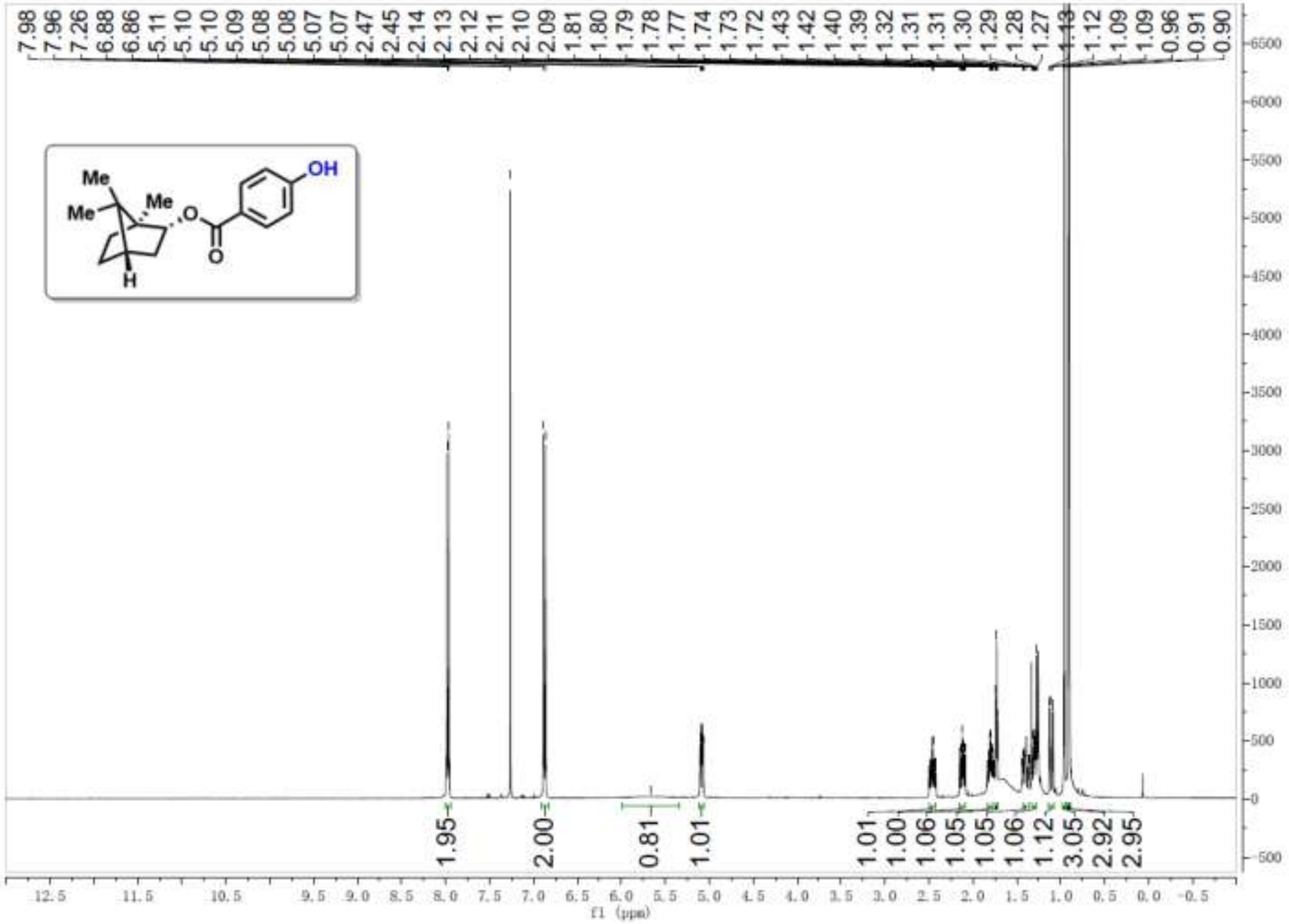

^{13}C NMR of 2ap

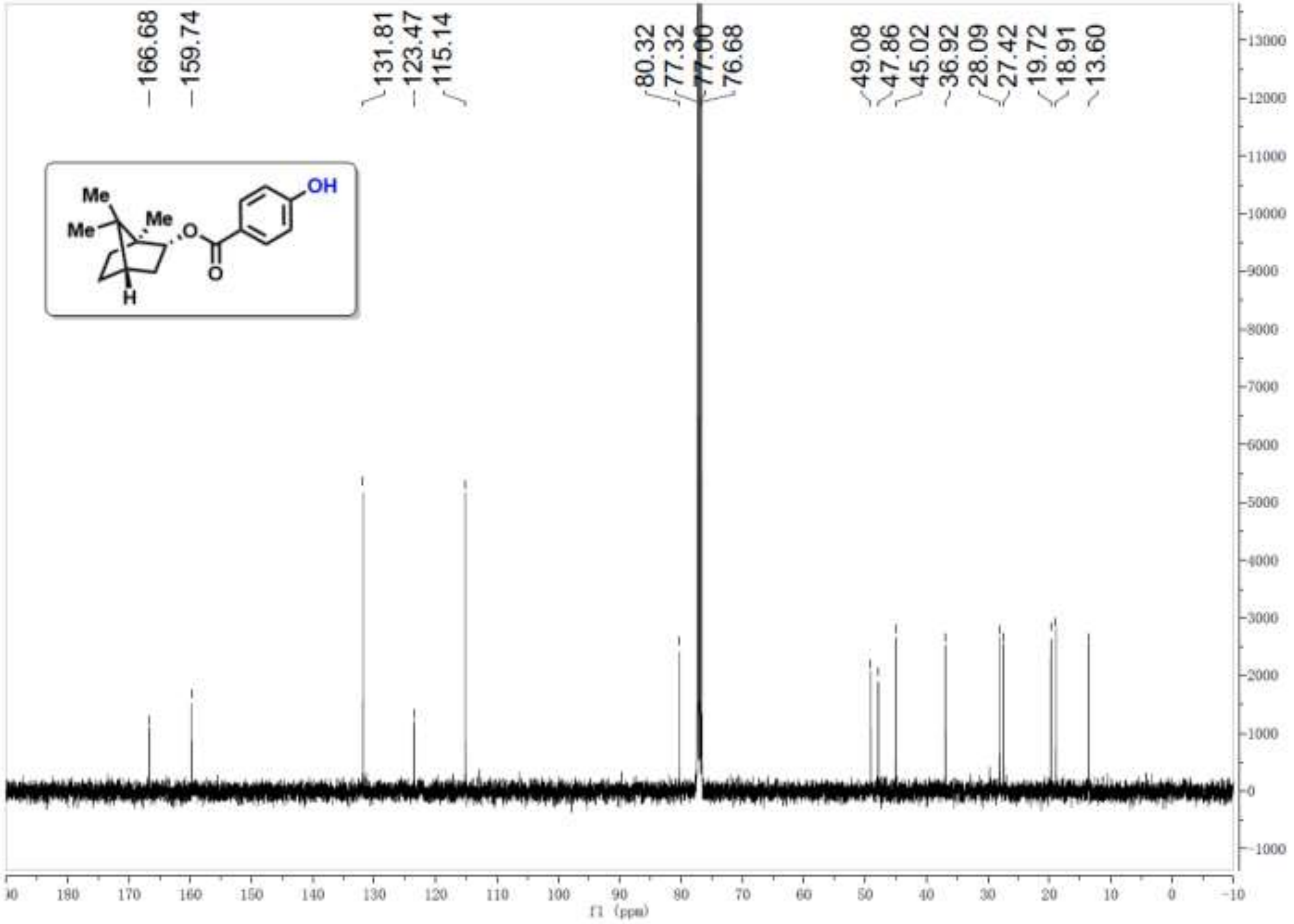

^{1}H NMR of 2aq

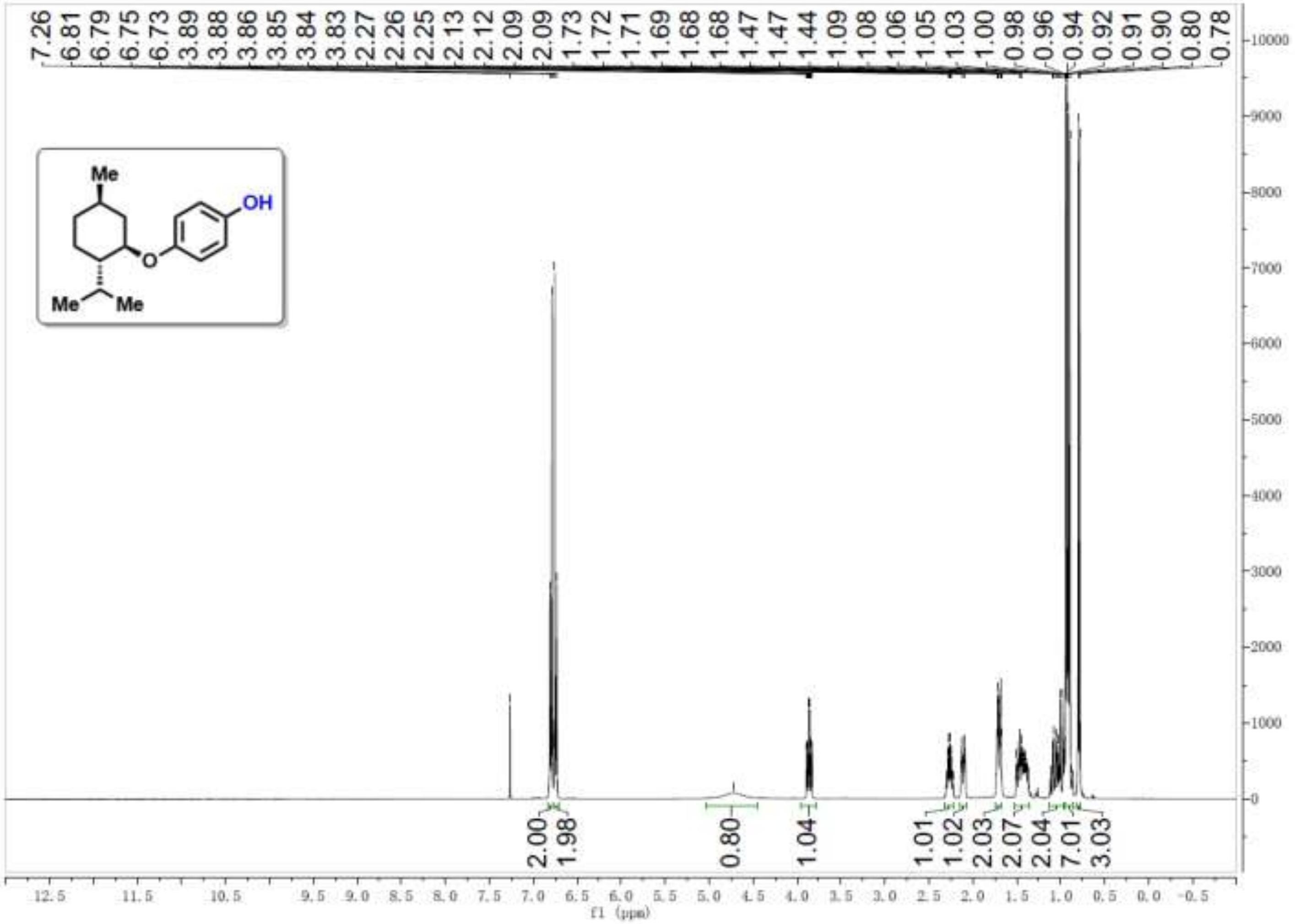

^{13}C NMR of 2aq

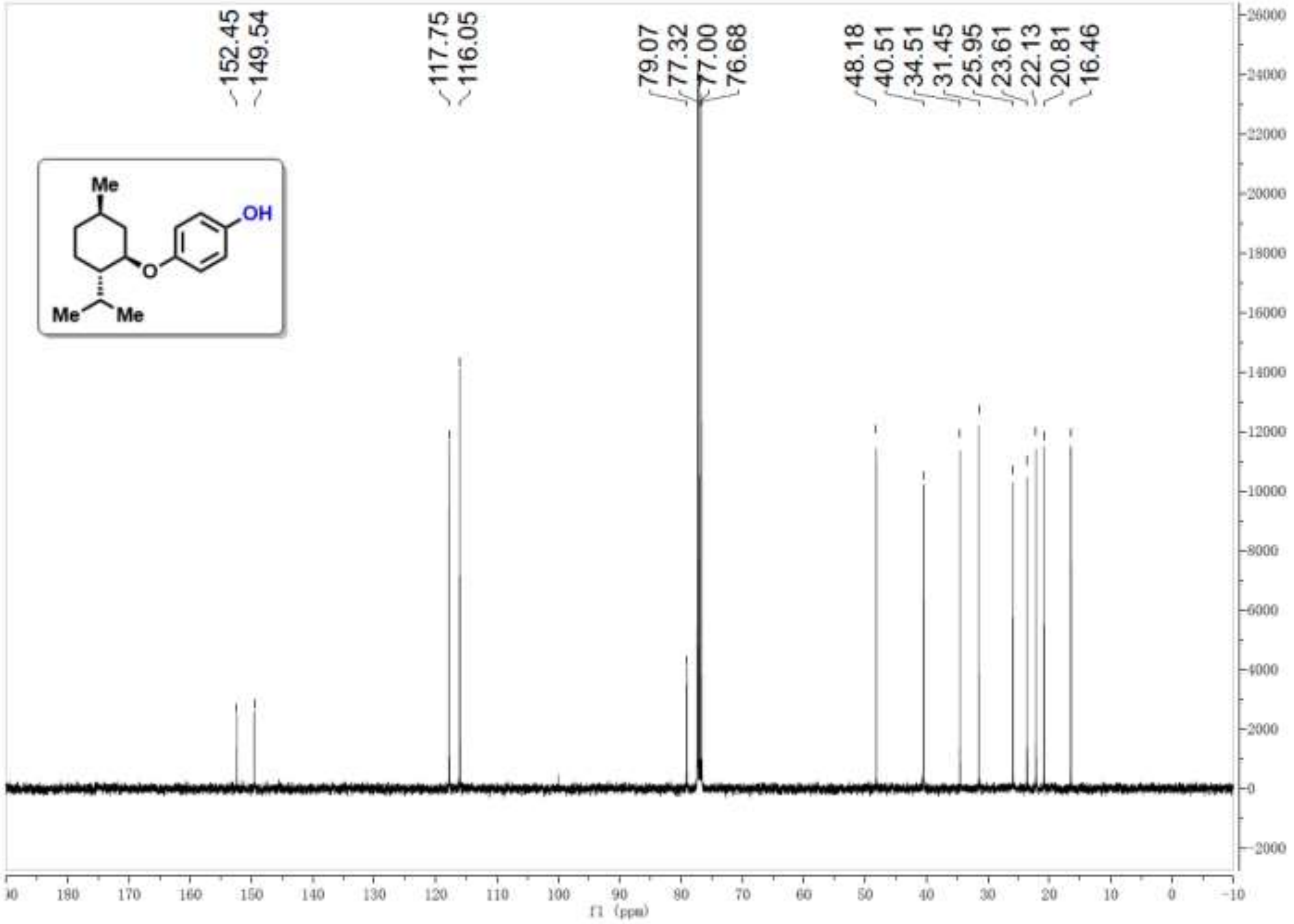

^{1}H NMR of 2ar

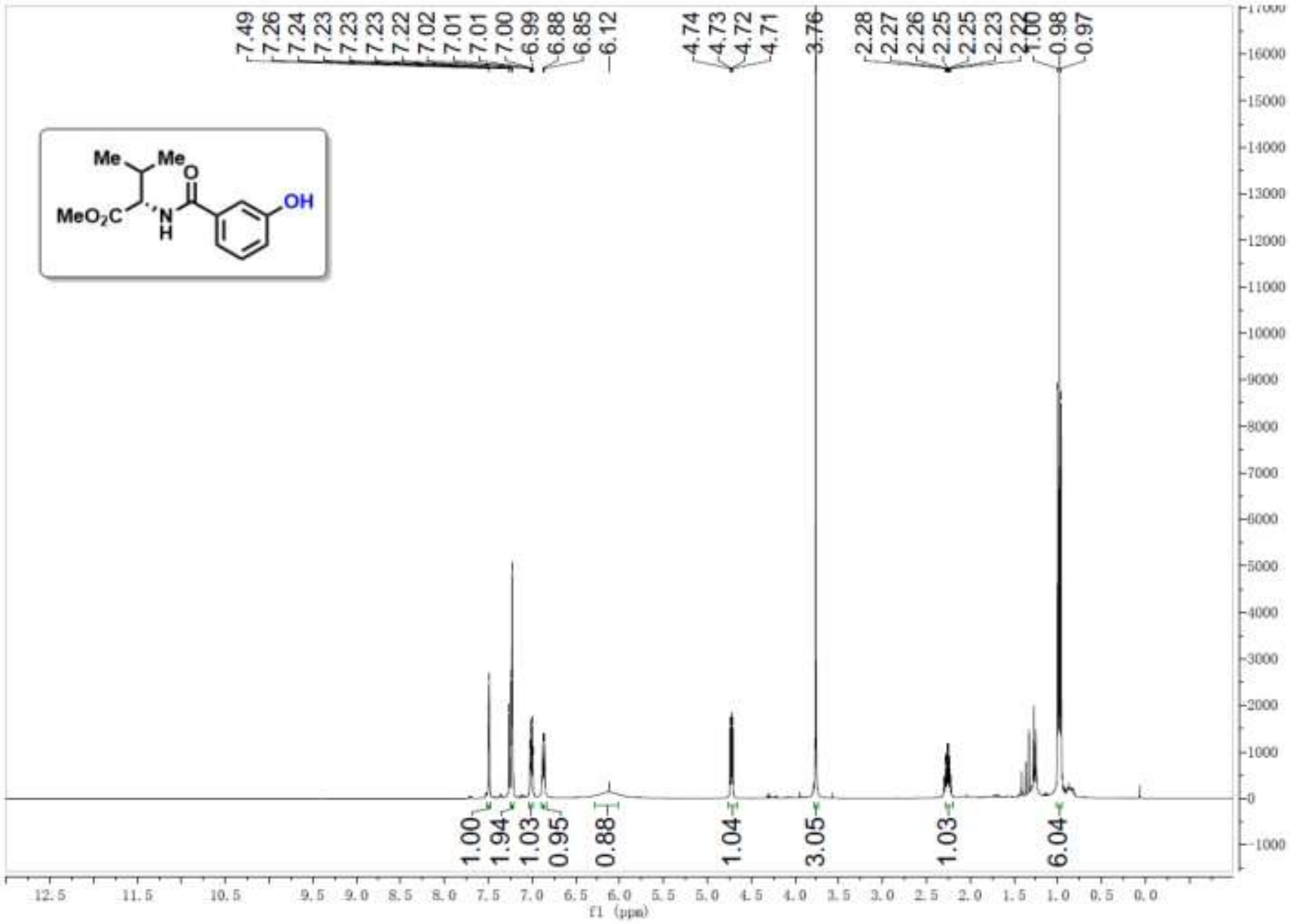

7.49
7.26
7.24
7.23
7.23
7.23
7.22
7.02
7.01
7.01
7.00
6.99
6.88
6.85
6.12
4.74
4.73
4.72
4.71
3.76
2.28
2.27
2.26
2.25
2.25
2.23
2.22
1.00
0.98
0.97
Me
Me
O
MeO2C
N
H
OH
1.00
1.94
1.03
0.95
0.88
1.04
3.05
1.03
6.04
f1 (ppm)

^{13}C NMR of 2ar

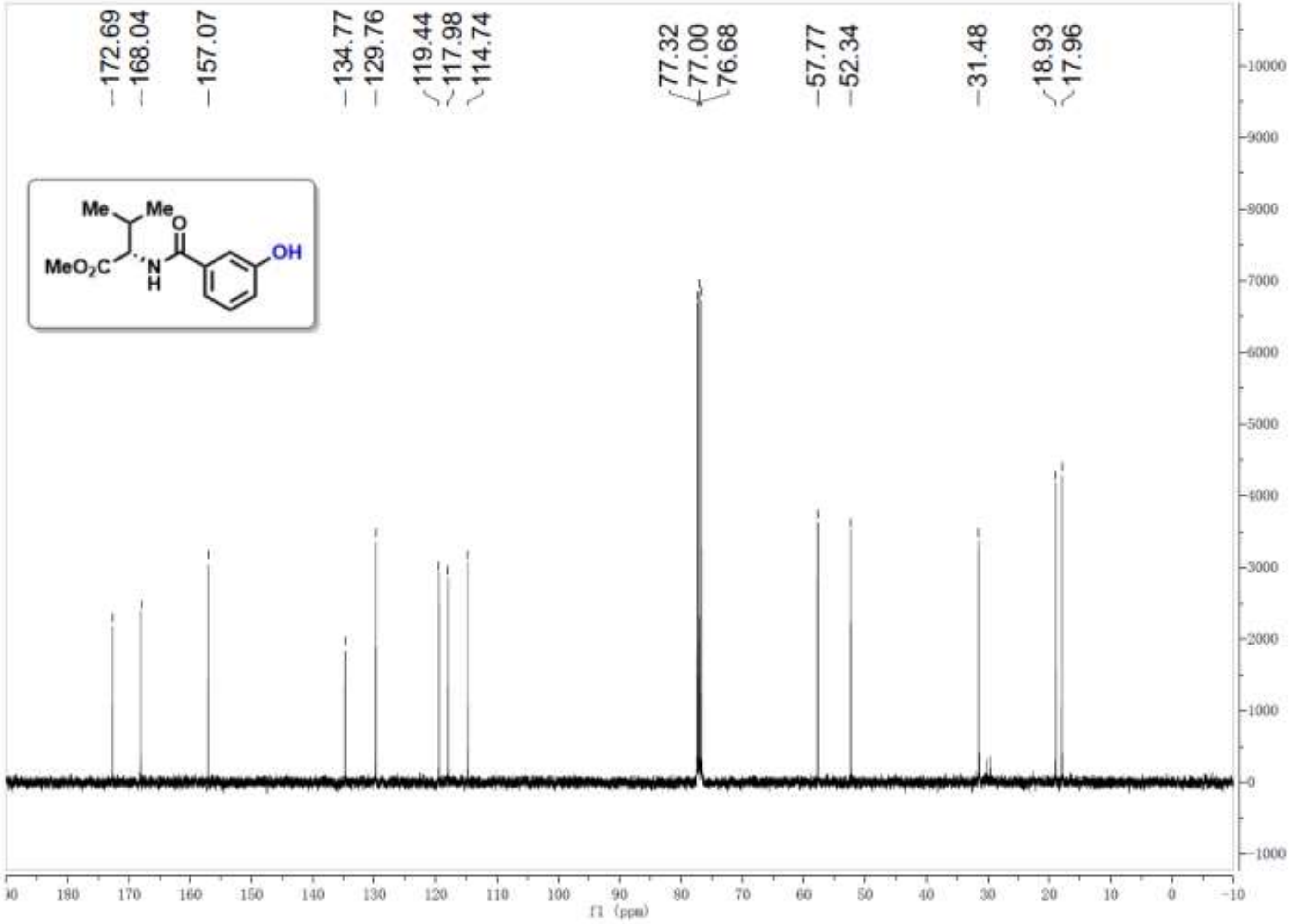

^{1}H NMR of 2as

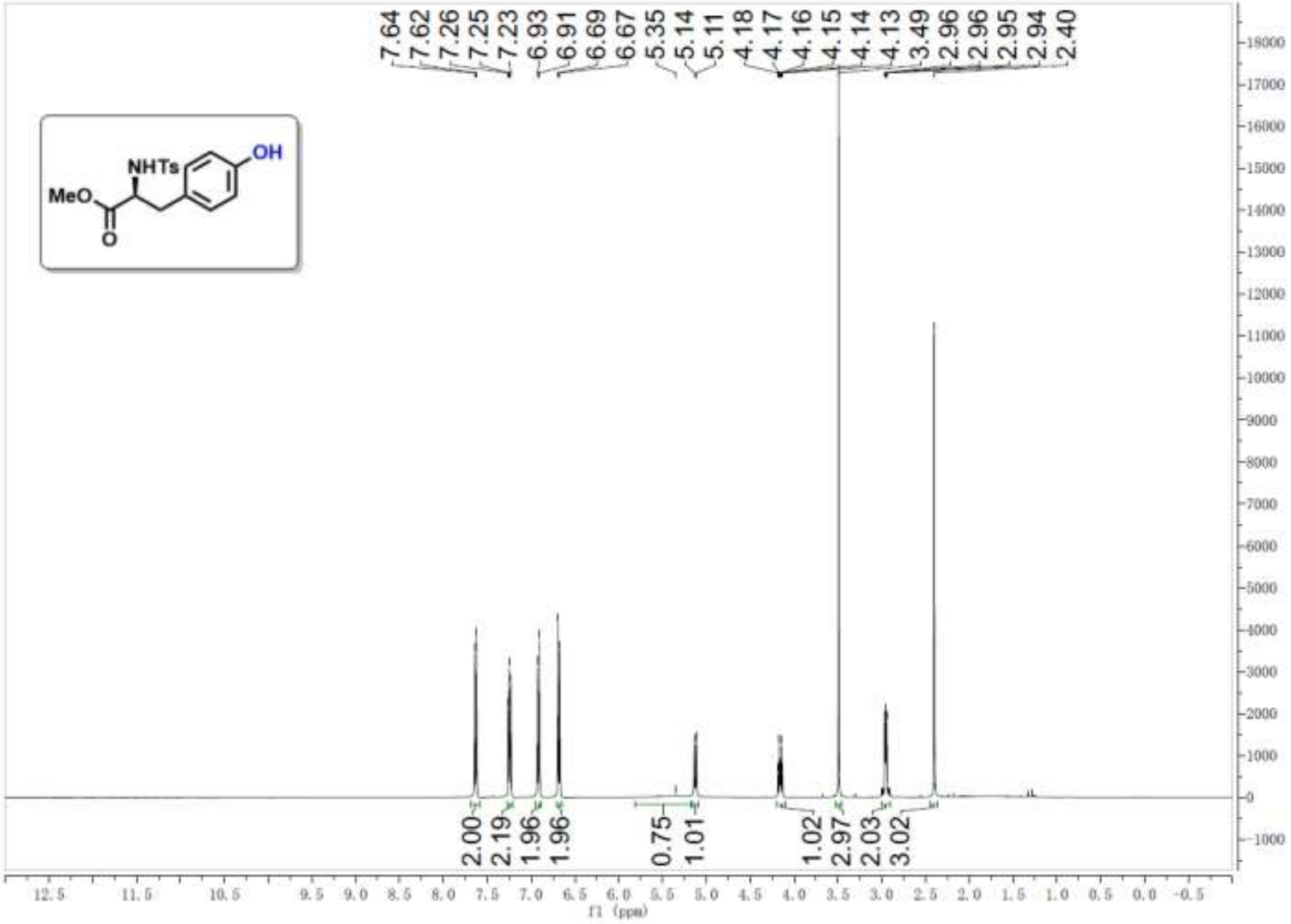

7.64
7.62
7.26
7.25
7.23
6.93
6.91
6.69
6.67
5.35
5.14
5.11
4.18
4.17
4.16
4.15
4.14
4.13
3.49
2.96
2.96
2.95
2.94
2.40
NHTs
OH
MeO
O
2.00
2.19
1.96
1.96
0.75
1.01
1.02
2.97
2.03
3.02
f1 (ppm)

^{13}C NMR of 2as

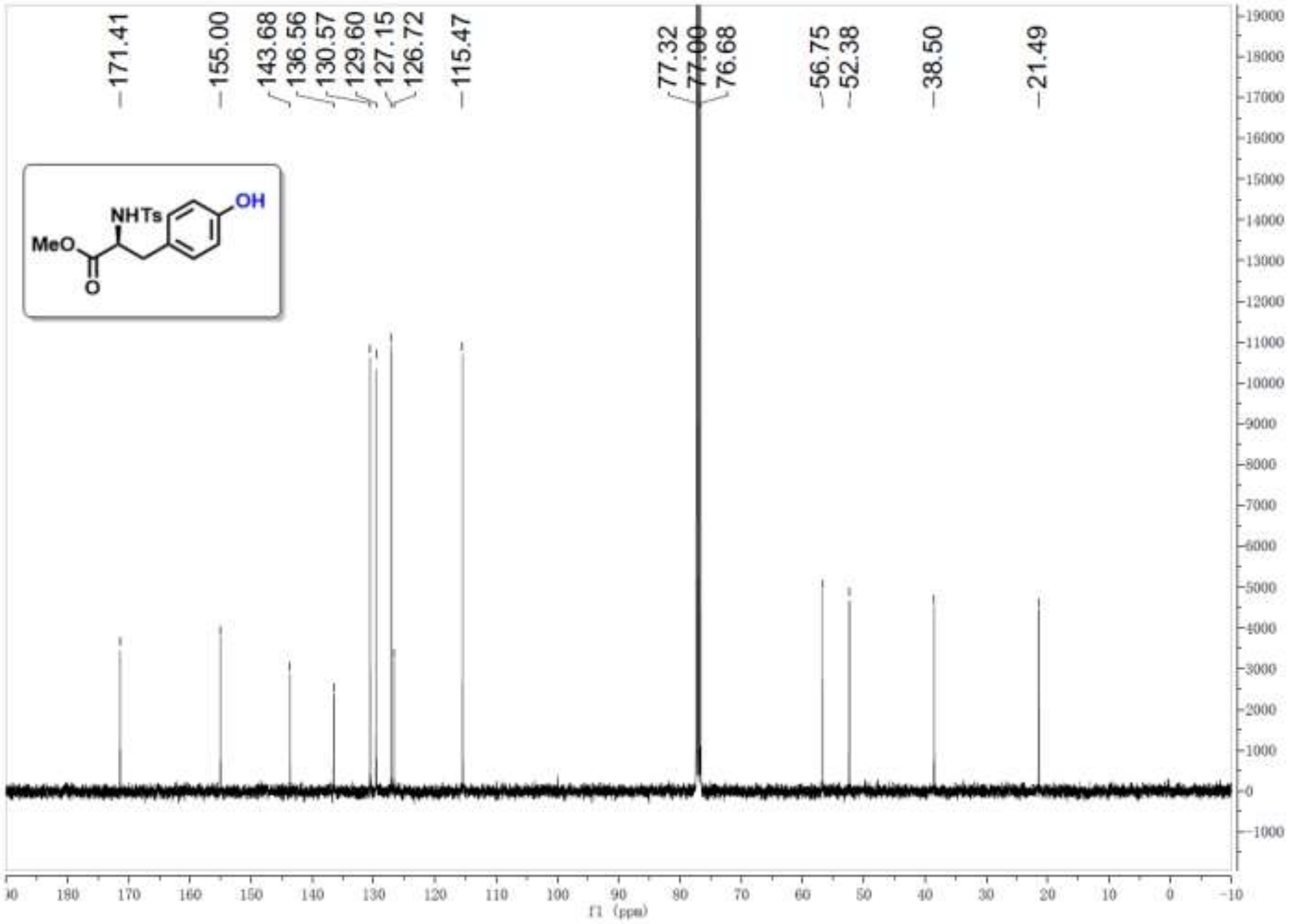

^{1}H NMR of 2at

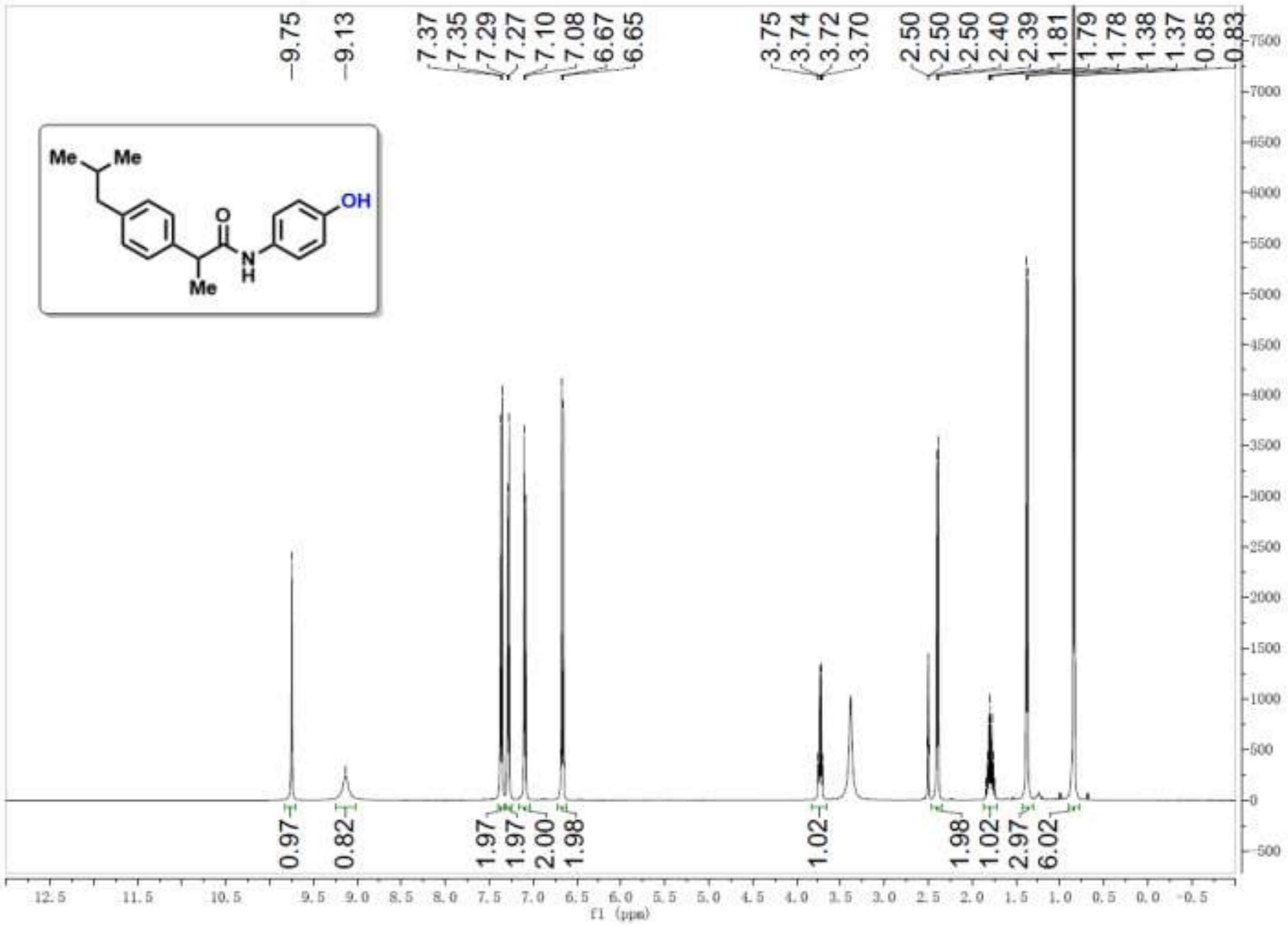

9.75
9.13
7.37
7.35
7.29
7.27
7.10
7.08
6.67
6.65
3.75
3.74
3.72
3.70
2.50
2.50
2.50
2.40
2.39
1.81
1.79
1.78
1.38
1.37
0.85
0.83
Me
Me
OH
O
N
H
Me
0.97
0.82
1.97
1.97
2.00
1.98
1.02
1.98
1.02
2.97
6.02
f1 (ppm)

^{13}C NMR of 2at

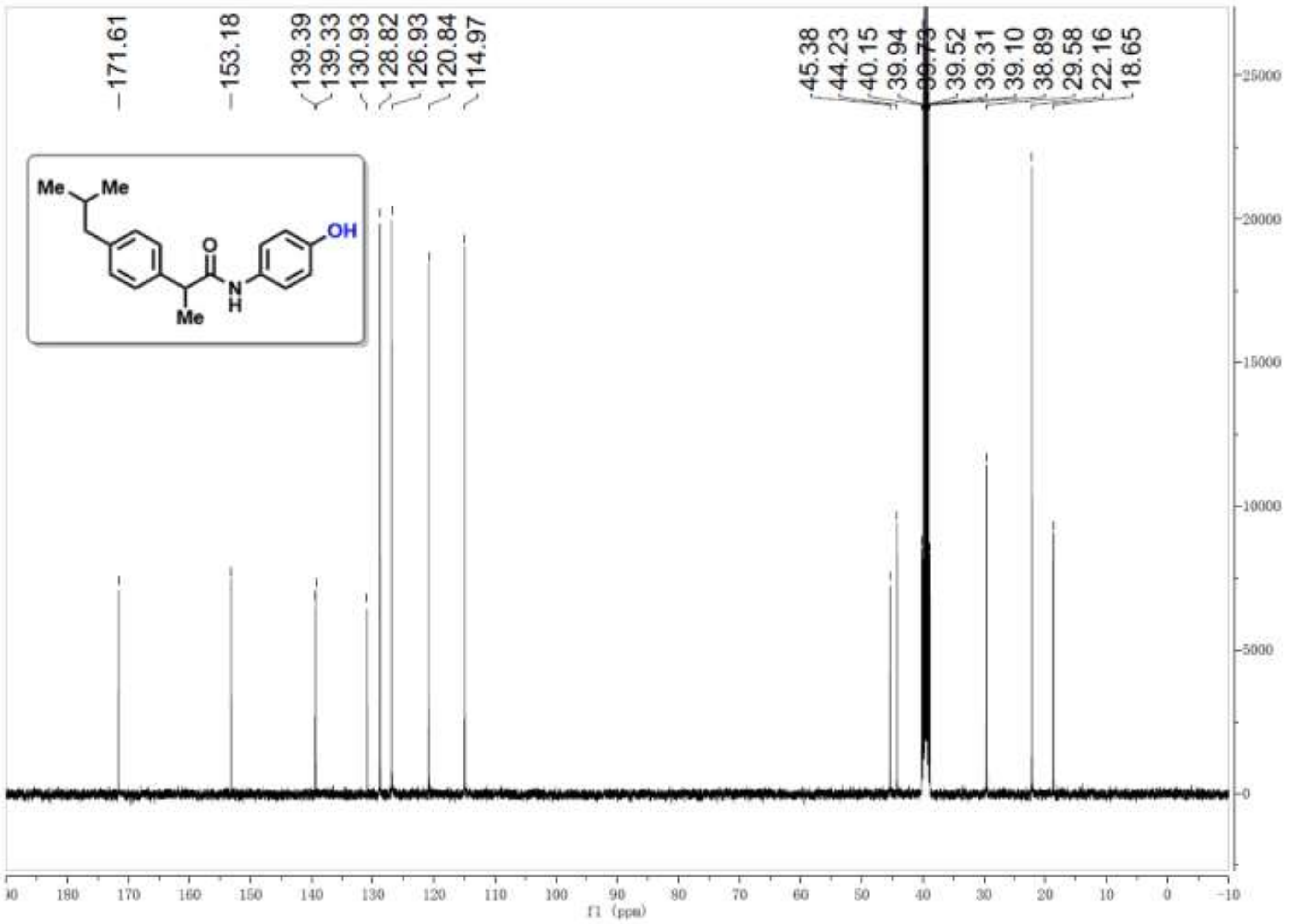

^{1}H NMR of 2au

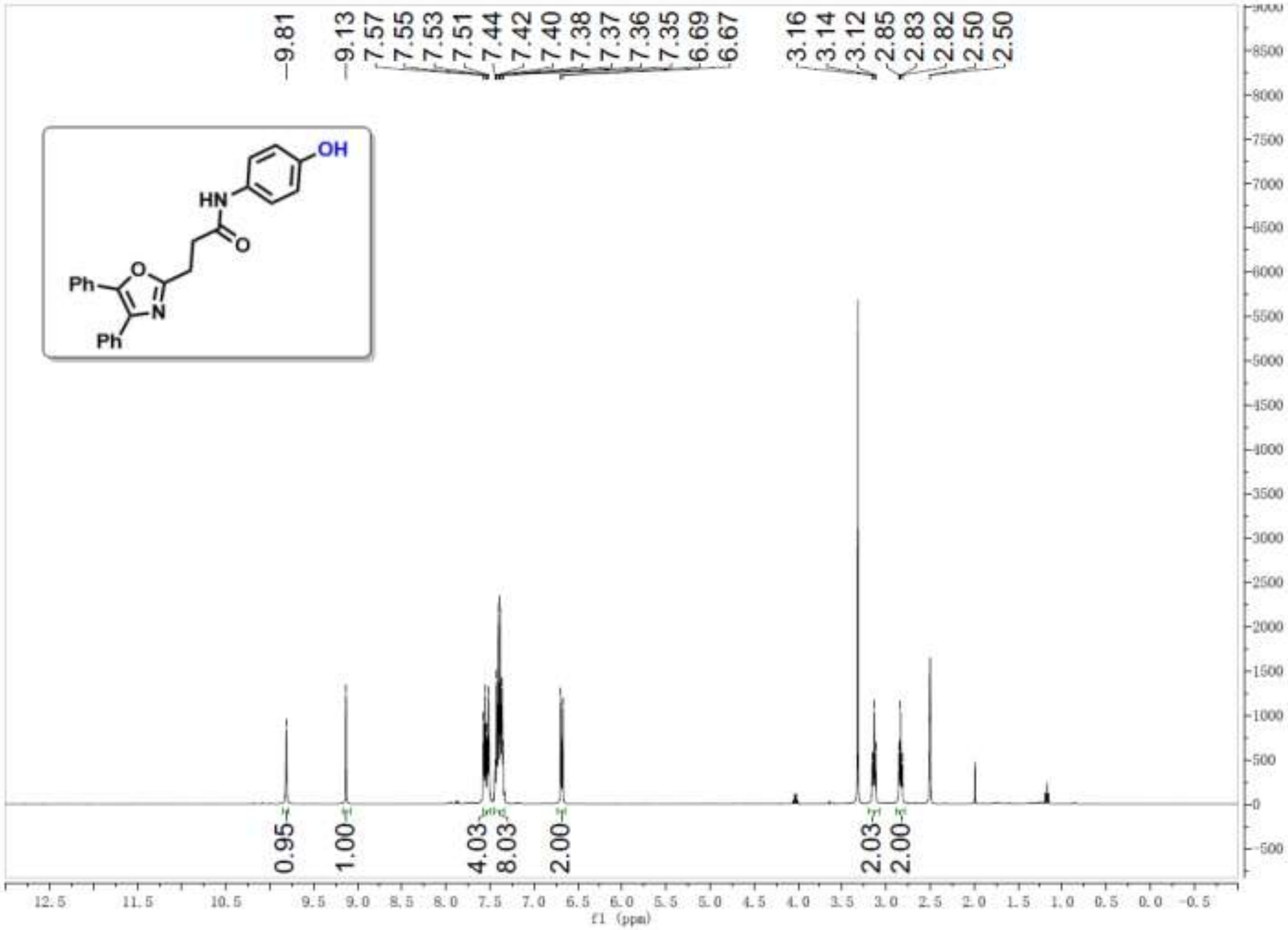

^{13}C NMR of 2au

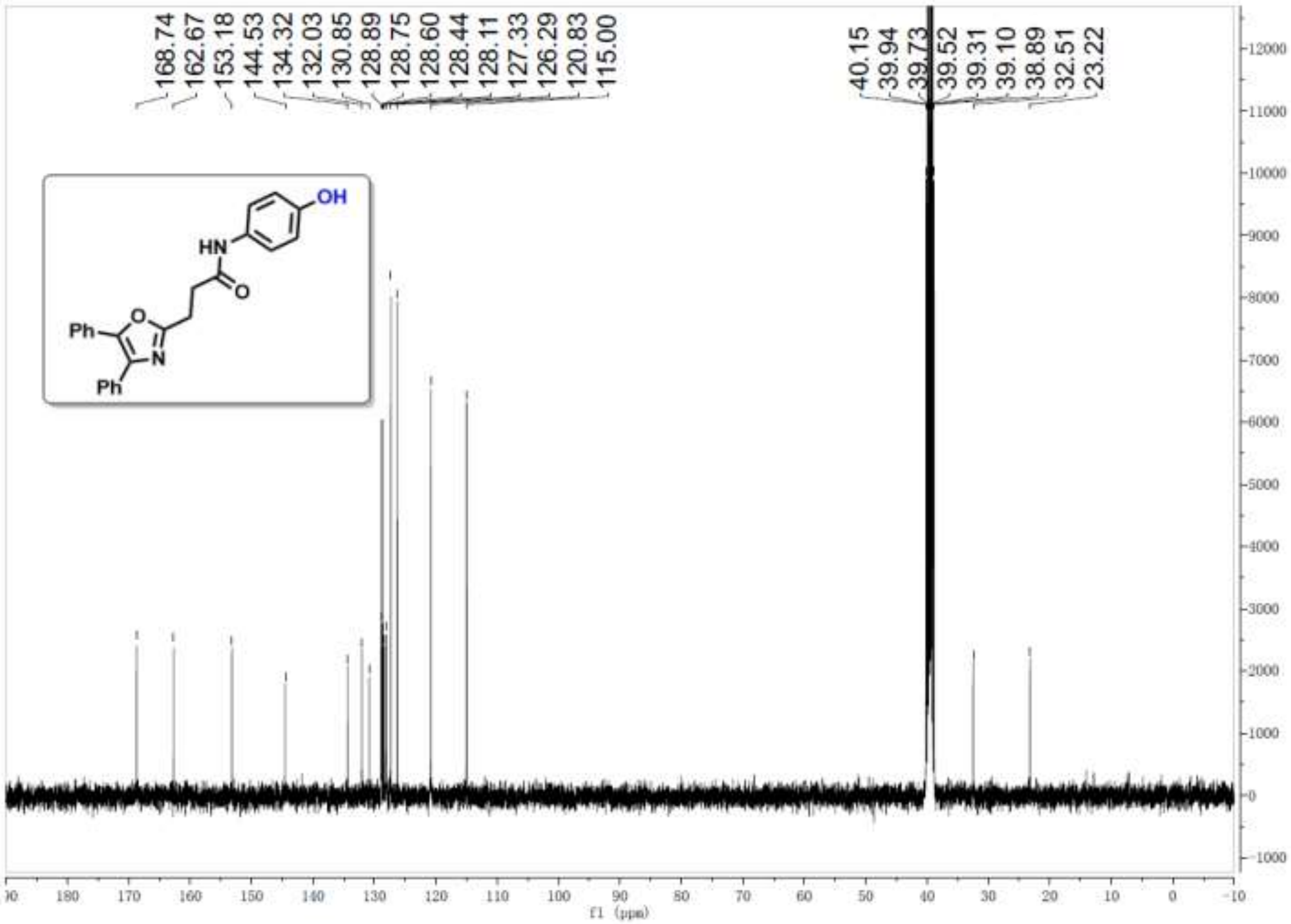

^{1}H NMR of 2av

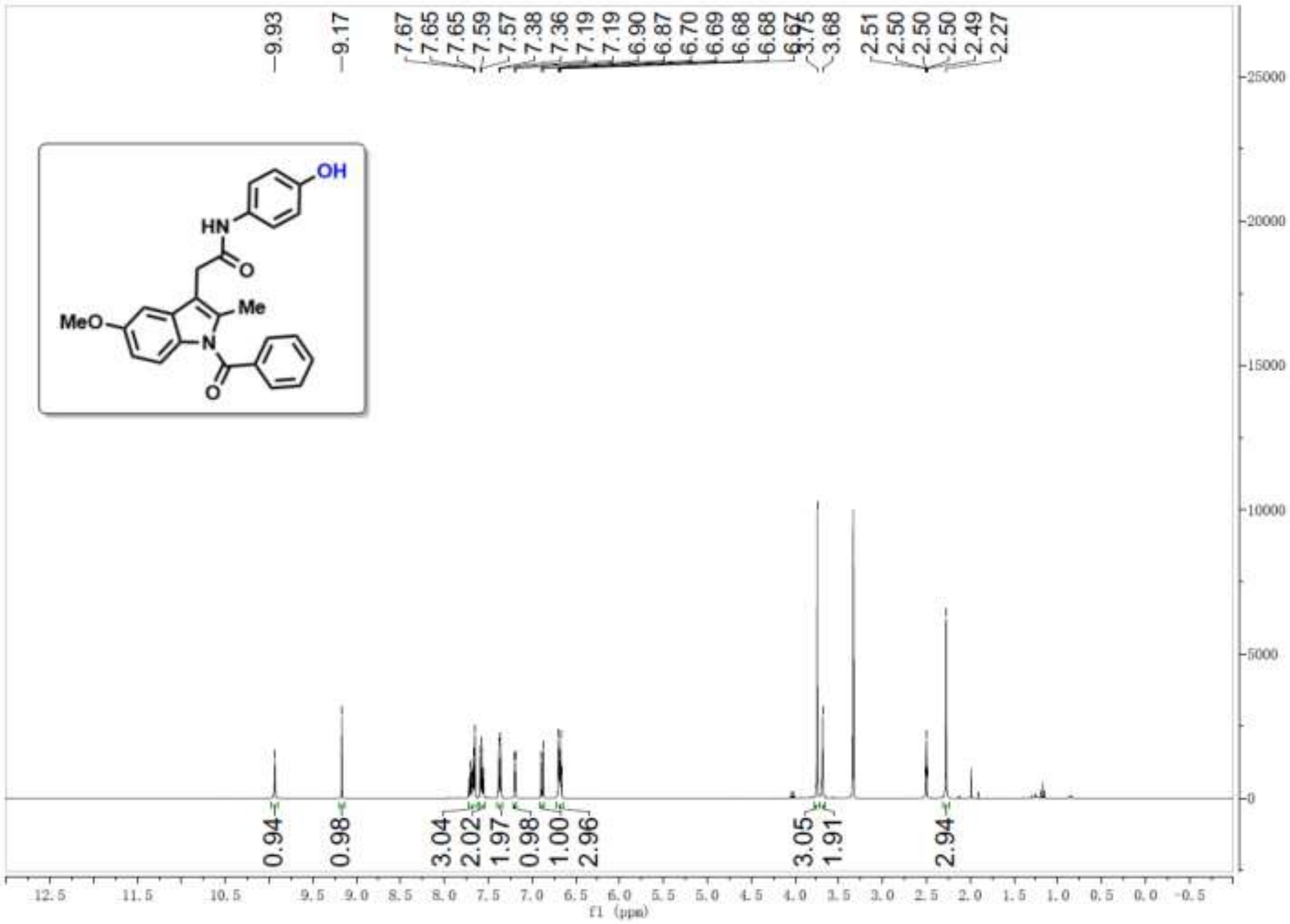

9.93
9.17
7.67
7.65
7.65
7.59
7.57
7.38
7.36
7.19
7.19
6.90
6.87
6.70
6.69
6.68
6.68
6.67
3.75
3.68
2.51
2.50
2.50
2.50
2.49
2.27
OH
HN
O
Me
MeO
N
O
0.94
0.98
3.04
2.02
1.97
0.98
1.00
2.96
3.05
1.91
2.94
25000
20000
15000
10000
5000
0
f1 (ppm)

^{13}C NMR of 2av

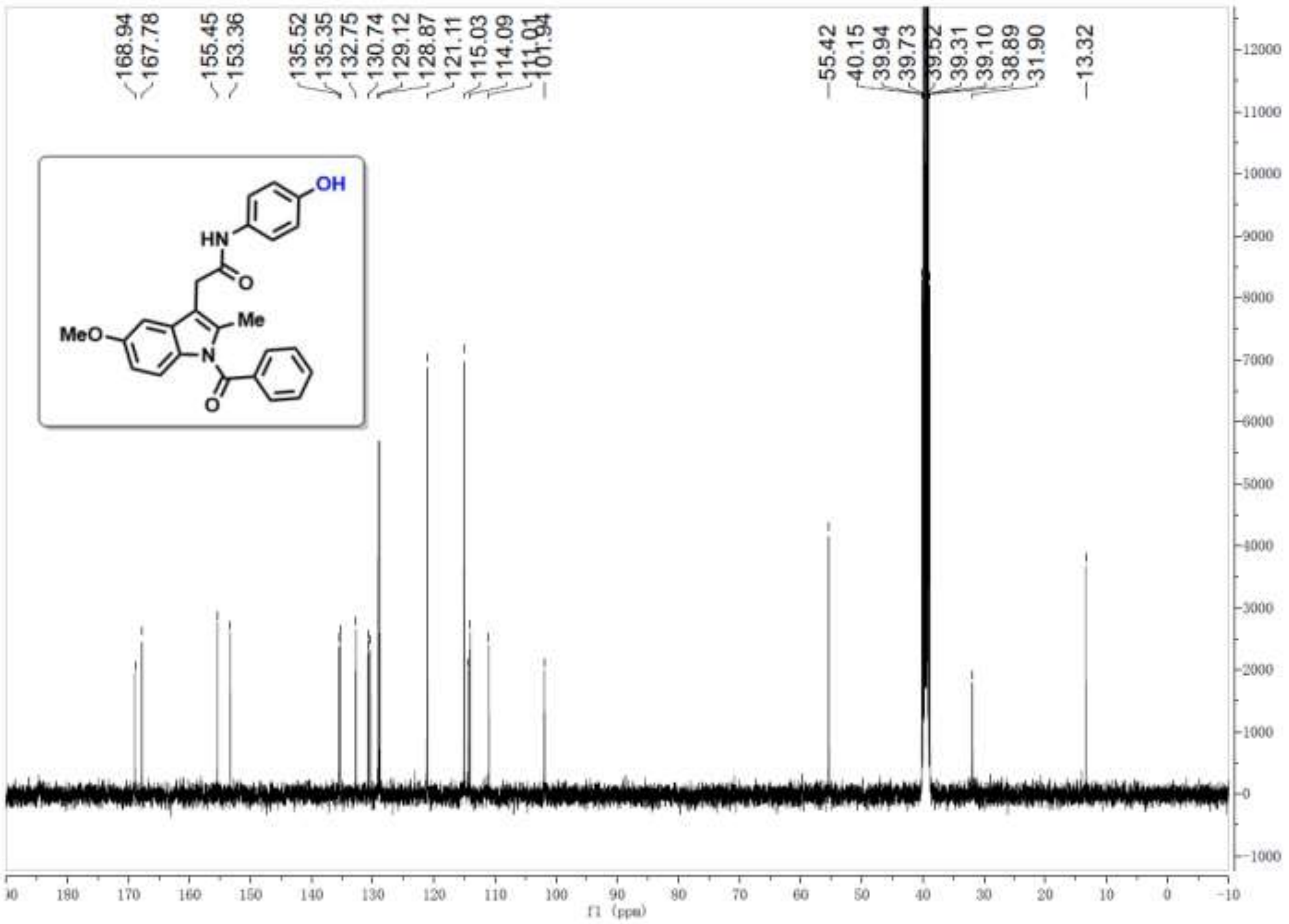

^{1}H NMR of 2aw

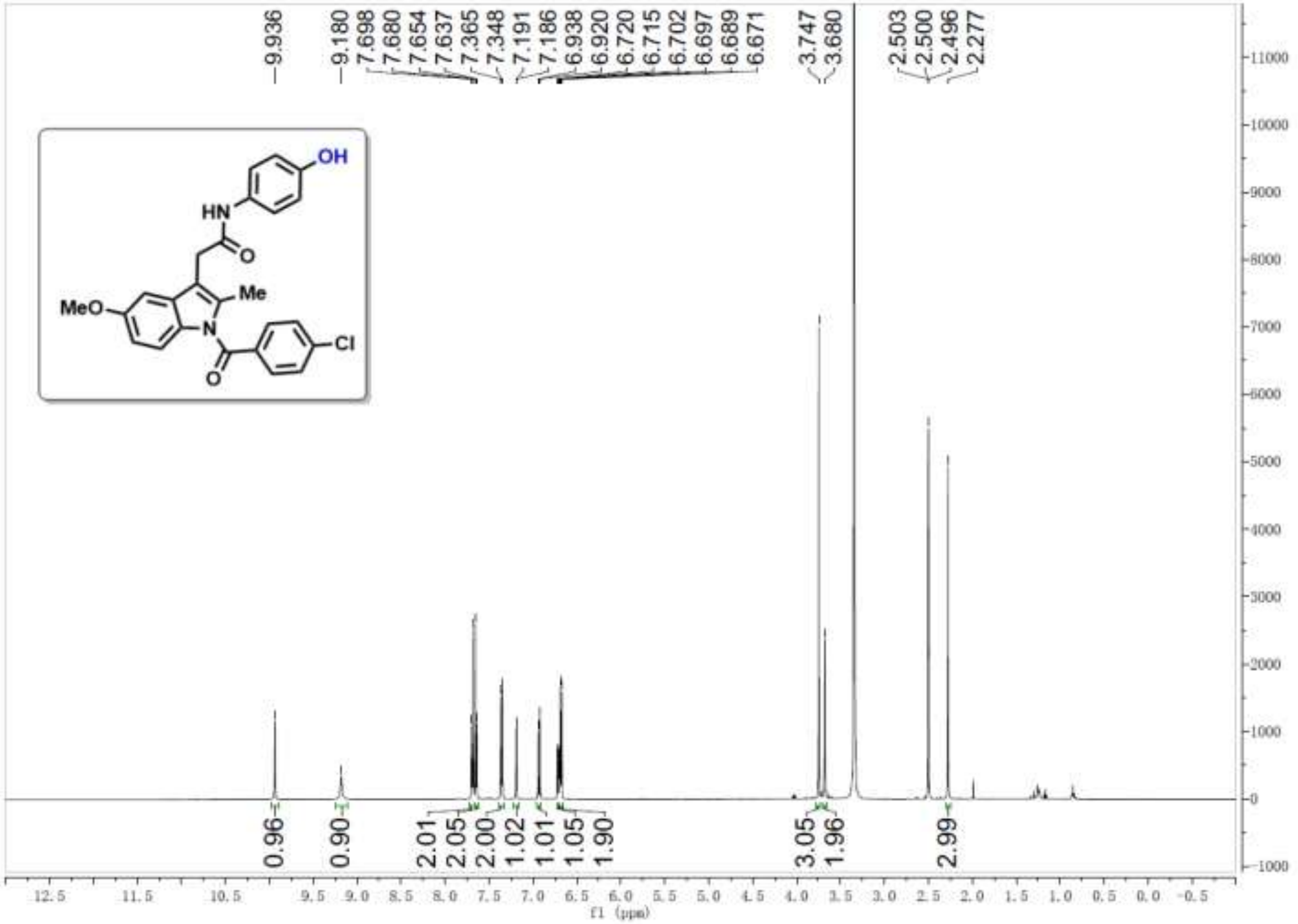
9.936
9.180
7.698
7.680
7.654
7.637
7.365
7.348
7.191
7.186
6.938
6.920
6.720
6.715
6.702
6.697
6.689
6.671
3.747
3.680
2.503
2.500
2.496
2.277
OH
HN
O
Me
MeO
N
Cl
O
0.96
0.90
2.01
2.05
2.00
1.02
1.01
1.05
1.90
3.05
1.96
2.99
f1 (ppm)

^{13}C NMR of 2aw

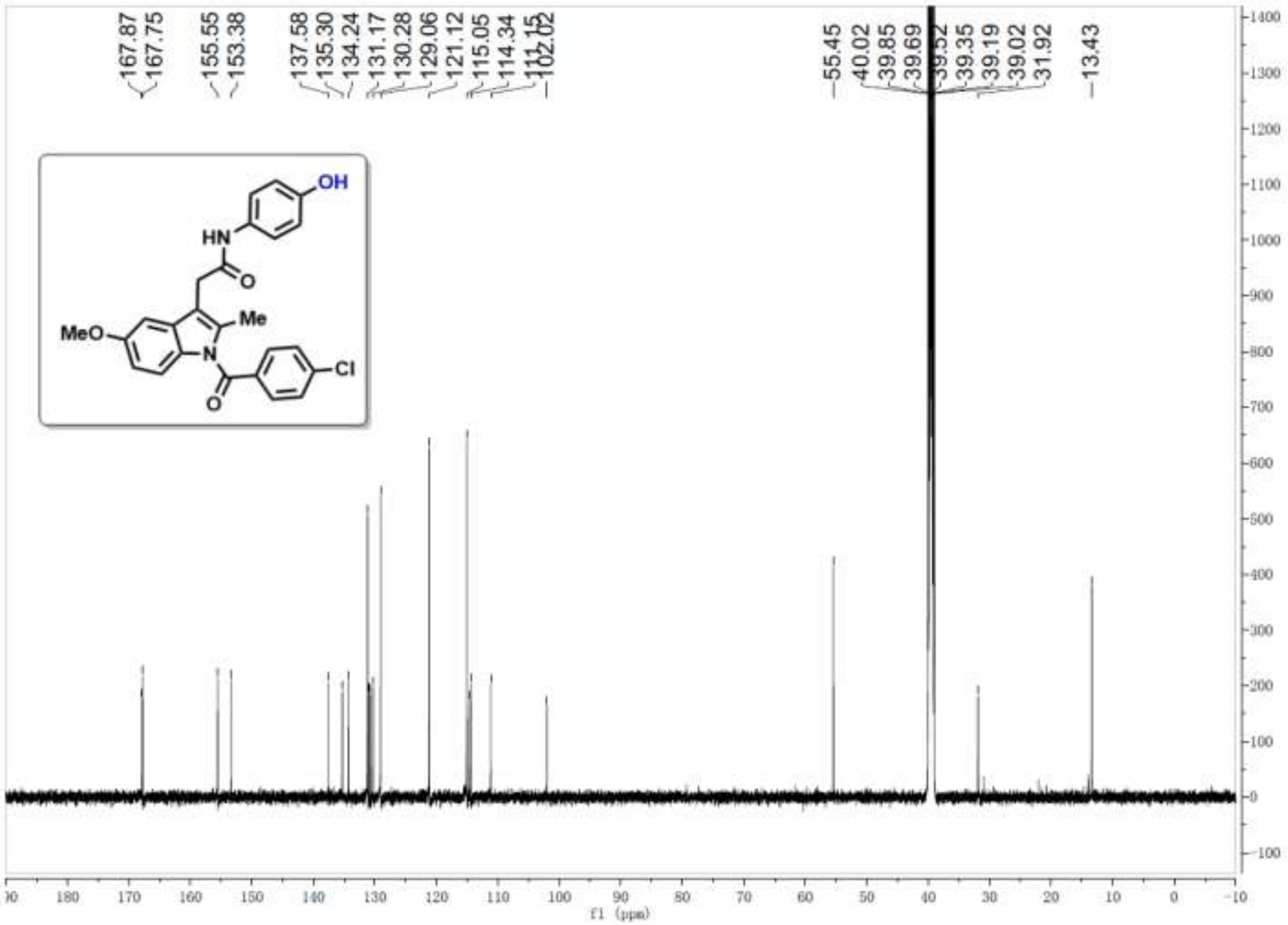

^{1}H NMR of 4a

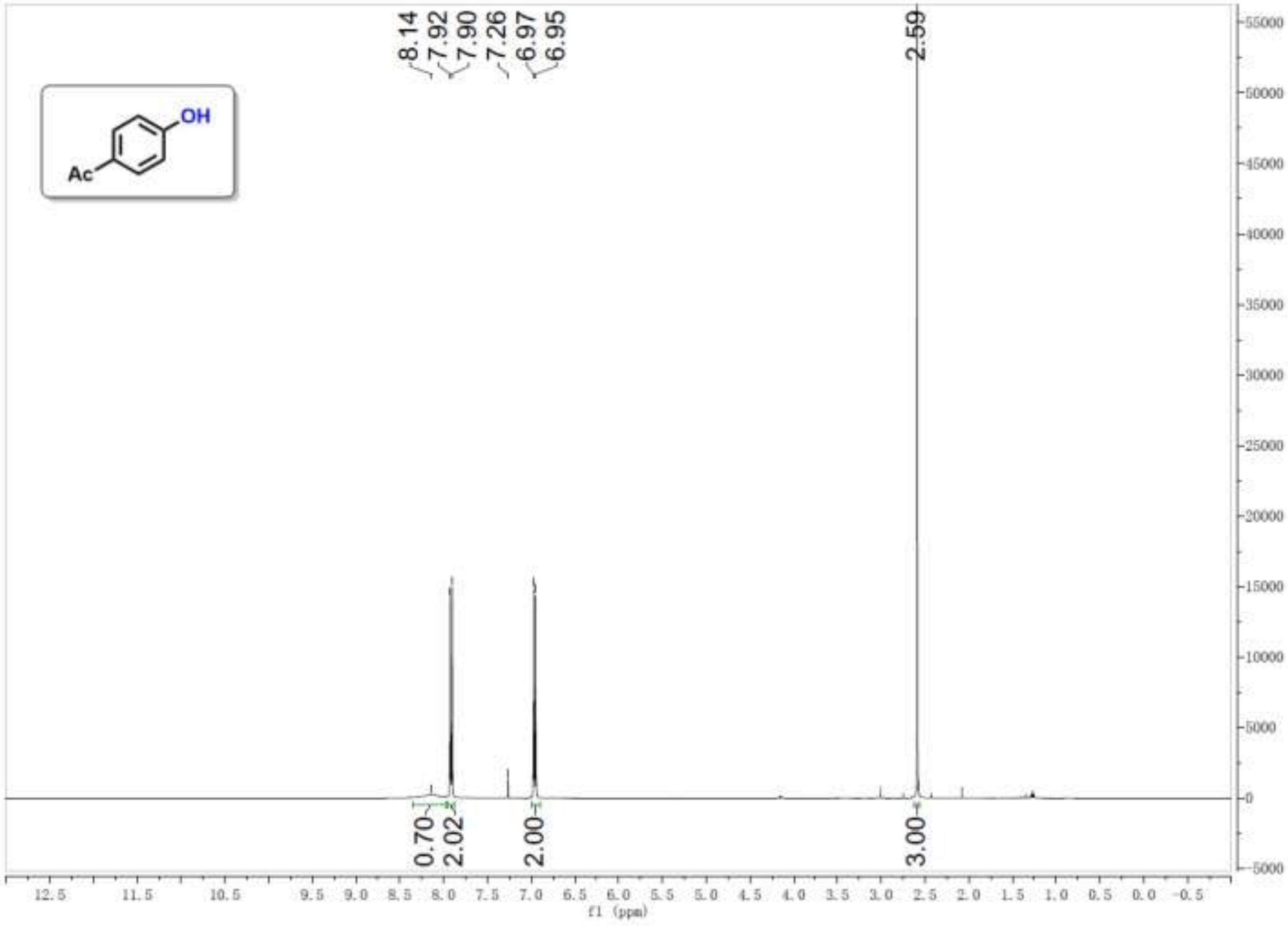

^{13}C NMR of 4a

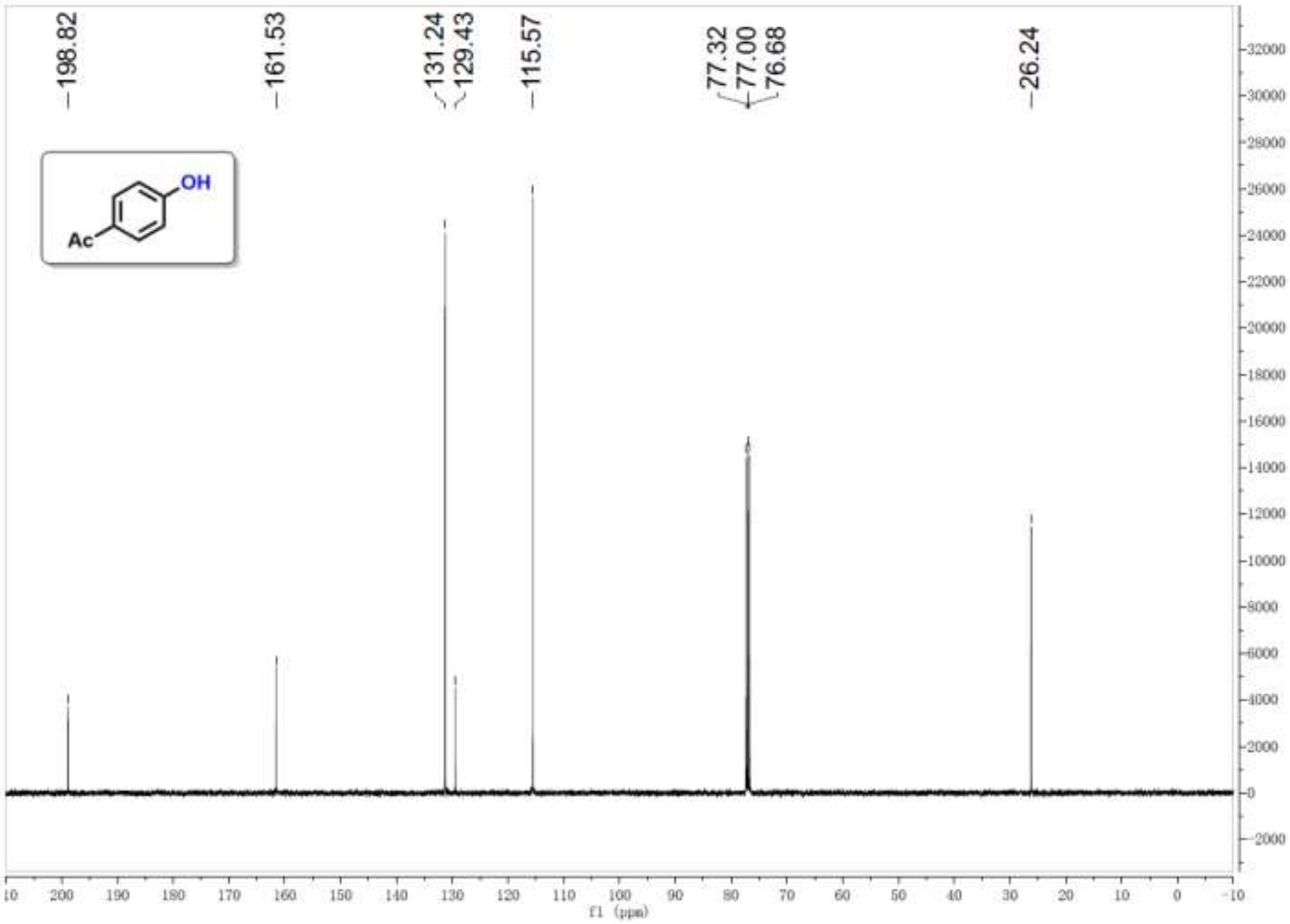

198.82
161.53
131.24
129.43
115.57
77.32
77.00
76.68
26.24
OH
Ac
f1 (ppm)

X. References:

1. Saito, K.; Kawamura, A.; Kanie, T.; Ueda, Y.; Kondo, S. *Hetrocycles* **2001**, *55*, 1071-1080.
2. Huvaere, K.; Olsen, K.; Skibsted, L. H. *J. Org. Chem.* **2009**, *74*, 7283-7293.
3. Luo, Y.-R. *Comprehensive Handbook of Chemical Bond Energies.* CRC press. **2007**.
4. Bahamonde, A.; Melchiorre, P., Mechanism of the Stereoselective α-Alkylation of Aldehydes Driven by the Photochemical Activity of Enamines. *J. Am. Chem. Soc.* **2016,** *138*, 8019-8030.
5. Zhang, W.; Zhu, Y.; Zhang, L.; Luo, S., Asymmetric *α*-Alkylation of *β*-Ketocarbonyls via Direct Phenacyl Bromide Photolysis by Chiral Primary Amine. *Chin. J. Chem.* **2018,** *36*, 716-722.
6. Montalti, M.; Credi, A.; Prodi, L.; Gandolfi, M. T. *Handbook of Photochemistry*. CRC Press, Taylor & Francis Group: **2006**.
7. Bhuvaneshwari, D. S.; Elango, K. P. *Z. Naturforsch.* **2006**, *61b*, 1254-1260.
8. Ando, S.; Tsuzaki, M.; Ishizuka, T. *J. Org. Chem.* **2020**, *85*, 11181-11189.
9. Zhu, C.; Wang, R.; Falck, J. R. *Org. Lett.* **2012**, *14*, 3494-3497.
10. Taniguchi, T.; Imoto, M.; Takeda, M.; Nakai, T.; Mihara, M.; Iwai, T.; Ito, T.; Mizuno, T.; Nomoto, A.; Ogawa, A. *Heteroatom Chem.* **2015**, *26*, 411-416.
11. Jones, R. M.; van de Water, R. W.; Lindsey, C. C.; Hoarau, C.; Ung, T.; Pettus, T. R. R. *J. Org. Chem.* **2001**, *66*, 3435-3441.
12. Ikeda, T.; Zhang, Z. Z.; Motoyama, Y. *Adv. Synth. Catal.* **2019**, *361*, 673-677.
13. Yang, L.; Huang, Z. Y.; Li, G.; Zhang, W.; Cao, R.; Wang, C.; Xiao, J. L.; Xue, D. *Angew. Chem. Int. Ed.* **2018**, *57*, 1968-1972.
14. Morin, J.; Zhao, Y. G.; Snieckus, V. *Org. Lett.* **2013**, *15*, 4102-4105.
15. Lin, F.; Song, Q. L.; Gao, Y. Y.; Cui, X. L. *RSC Adv.* **2014**, *4*, 19856-19860.
16. Pramanik, C.; Kotharkar, S.; Patil, P.; Gotrane, D.; More, Y.; Borhade, A.; Chaugule, B.; Khaladkar, T.; Neelakandan, K.; Chaudhari, A.; Kulkarni, M. G.; Tripathy, N. K.; Gurjar, M. K. *Org. Process Res. Dev.* **2014**, *18*, 152-156.
17. Goldsmith, C. R.; Jonas, R. T.; Stack, T. D. P. *J. Am. Chem. Soc.* **2002**, *124*, 83-96.
18. Maleczka, Jr., R. E.; Shi, F.; Holmes, D.; Smith, III, M. R. *J. Am. Chem. Soc.* **2003**, *125*, 7792-7793.
19. Feldman, P. L.; Rapoport, H. *Synthesis* **1986**, *9*, 735-737.
20. Imoto, M.; Matsui, Y.; Takeda, M.; Tamaki, A.; Taniguchi, H.; Mizuno, K.; Ikeda, H. *J. Org. Chem.* **2011**, *76*, 6356-6361.
21. Gao, Y.; Liu, J. J.; Li, Z. J.; Guo, T. F.; Xu, S. Q.; Zhu, H.; Wei, F. L.; Chen, S. M.; Gebru, H.; Guo, K. *J. Org. Chem.* **2018**, *83*, 2040-2049.
22. Luo, D. P.; Huang, Y. F.; Hong, X. Y.; Chen, D. B.; Li, G. X.; Huang, X. B.; Gao, W. X.; Liu, M. C.; Zhou, Y. B.; Wu, H. Y. *Adv. Synth. Catal.* **2019**, *361*, 961-964.
23. Jouvin, K.; Matheis, C.; Goossen, L. J. *Chem. Eur. J.* **2015**, *21*, 14324-14327.
24. Ji, H.; Wu, L. Y.; Cai, J. H.; Li, G. R.; Gan, N. N.; Wang, Z. H. *RSC Adv.* **2018**, *8*, 13643-13648.
25. Sahoo, B.; Surkus, A. E.; Pohl, M. M.; Radnik, J.; Schneider, M.; Bachmann, S.; Scalone, M.; Junge, K.; Beller, M. *Angew. Chem. Int. Ed.* **2017**, *56*, 11242-11247.
