# Supplementary material for: From aniline to phenol: carbon-nitrogen bond activation via uranyl photoredox catalysis
Source: Natl Sci Rev. 2021 Aug 20;9(6):nwab156. doi: 10.1093/nsr/nwab156 (PMC9283103; doi:10.1093/nsr/nwab156)

checkCIF/PLATON report

Structure factors have been supplied for datablock(s) exp_1639

THIS REPORT IS FOR GUIDANCE ONLY. IF USED AS PART OF A REVIEW PROCEDURE FOR PUBLICATION, IT SHOULD NOT REPLACE THE EXPERTISE OF AN EXPERIENCED CRYSTALLOGRAPHIC REFEREE.

No syntax errors found. CIF dictionary Interpreting this report

Datablock: exp_1639

```
Bond precision:  C-C = 0.0167 A          Wavelength=1.54184

Cell:            a=9.4784(3)     b=10.4081(3)       c=21.5648(7)
                 alpha=90        beta=93.555(3)     gamma=90
Temperature:     170 K

                Calculated                    Reported
Volume          2123.32(11)                   2123.32(11)
Space group     P 21                          P 1 21 1
Hall group      P 2yb                         P 2yb
Moiety formula  C25 H22 N2 O4 [+ solvent]     C25 H22 N2 O4
Sum formula     C25 H22 N2 O4 [+ solvent]     C25 H22 N2 O4
Mr              414.45                        414.44
Dx,g cm-3       1.296                         1.296
Z               4                             4
Mu (mm-1)       0.720                         0.720
F000            872.0                         872.0
F000'           874.71
h,k,lmax        11,12,25                      11,12,25
Nref            7581[ 4020]                   6824
Tmin,Tmax       0.772,0.944                   0.510,1.000
Tmin'           0.772

Correction method= # Reported T Limits: Tmin=0.510 Tmax=1.000
AbsCorr = MULTI-SCAN

Data completeness= 1.70/0.90      Theta(max)= 67.036

R(reflections)= 0.1226( 6031)     wR2(reflections)= 0.3296( 6824)

S = 0.878                  Npar= 565
```

The following ALERTS were generated. Each ALERT has the format
test-name_ALERT_alert-type_alert-level.
Click on the hyperlinks for more details of the test.

Alert level B

```
PLAT097_ALERT_2_B Large Reported Max.  (Positive) Residual Density       0.84 eA-3
PLAT340_ALERT_3_B Low Bond Precision on  C-C Bonds ...............    0.01667 Ang.
PLAT420_ALERT_2_B D-H Without Acceptor       O5       --H5A      .     Please Check
PLAT930_ALERT_2_B FCF-based   Twin Law ( 0 0 1)[ 1 0 5] Est.d BASF       0.37 Check
```

Alert level C

```
DIFMX02_ALERT_1_C  The maximum difference density is > 0.1*ZMAX*0.75
            The relevant atom site should be identified.
STRVA01_ALERT_4_C           Flack test results are ambiguous.
           From the CIF: _refine_ls_abs_structure_Flack    0.500
           From the CIF: _refine_ls_abs_structure_Flack_su    0.200
PLAT029_ALERT_3_C _diffrn_measured_fraction_theta_full value Low .      0.971 Why?
PLAT082_ALERT_2_C High R1 Value ..................................       0.12 Report
PLAT084_ALERT_3_C High wR2 Value (i.e. > 0.25) ...................       0.33 Report
PLAT089_ALERT_3_C Poor Data / Parameter Ratio (Zmax < 18) ........       6.91 Note
PLAT213_ALERT_2_C Atom O6              has ADP max/min Ratio .....        3.7 prolat
PLAT213_ALERT_2_C Atom C26             has ADP max/min Ratio .....        3.2 prolat
PLAT213_ALERT_2_C Atom C50             has ADP max/min Ratio .....        3.3 prolat
PLAT220_ALERT_2_C NonSolvent   Resd 2  C   Ueq(max)/Ueq(min) Range        4.5 Ratio
PLAT234_ALERT_4_C Large Hirshfeld Difference C46      --C47      .       0.20 Ang.
PLAT241_ALERT_2_C High   'MainMol' Ueq as Compared to Neighbors of        C27 Check
PLAT241_ALERT_2_C High   'MainMol' Ueq as Compared to Neighbors of        C45 Check
PLAT906_ALERT_3_C Large K Value in the Analysis of Variance ......      2.826 Check
PLAT911_ALERT_3_C Missing FCF Refl Between Thmin & STh/L=    0.597        116 Report
```

Alert level G

```
PLAT003_ALERT_2_G Number of Uiso or Uij Restrained non-H Atoms ...          4 Report
PLAT007_ALERT_5_G Number of Unrefined Donor-H Atoms ..............          4 Report
PLAT072_ALERT_2_G SHELXL First  Parameter in WGHT  Unusually Large       0.20 Report
PLAT083_ALERT_2_G SHELXL Second Parameter in WGHT  Unusually Large      15.60 Why ?
PLAT186_ALERT_4_G The CIF-Embedded .res File Contains ISOR Records          3 Report
PLAT605_ALERT_4_G Largest Solvent Accessible VOID in the Structure         32 A**3
PLAT790_ALERT_4_G Centre of Gravity not Within Unit Cell: Resd.  #          2 Note
              C25 H22 N2 O4
PLAT860_ALERT_3_G Number of Least-Squares Restraints .............         25 Note
PLAT870_ALERT_4_G ALERTS Related to Twinning Effects Suppressed ..          ! Info
PLAT909_ALERT_3_G Percentage of I>2sig(I) Data at Theta(Max) Still        73% Note
PLAT910_ALERT_3_G Missing # of FCF Reflection(s) Below Theta(Min).          1 Note
PLAT931_ALERT_5_G CIFcalcFCF  Twin Law ( 0 0 1)         Est.d BASF       0.37 Check
PLAT933_ALERT_2_G Number of OMIT Records in Embedded .res File ...        199 Note
```

---

 0 **ALERT level A** = Most likely a serious problem - resolve or explain
 4 **ALERT level B** = A potentially serious problem, consider carefully
15 **ALERT level C** = Check. Ensure it is not caused by an omission or oversight
13 **ALERT level G** = General information/check it is not something unexpected

 1 ALERT type 1 CIF construction/syntax error, inconsistent or missing data
14 ALERT type 2 Indicator that the structure model may be wrong or deficient
 9 ALERT type 3 Indicator that the structure quality may be low
 6 ALERT type 4 Improvement, methodology, query or suggestion
 2 ALERT type 5 Informative message, check

It is advisable to attempt to resolve as many as possible of the alerts in all categories. Often the minor alerts point to easily fixed oversights, errors and omissions in your CIF or refinement strategy, so attention to these fine details can be worthwhile. In order to resolve some of the more serious problems it may be necessary to carry out additional measurements or structure refinements. However, the purpose of your study may justify the reported deviations and the more serious of these should normally be commented upon in the discussion or experimental section of a paper or in the "special_details" fields of the CIF. checkCIF was carefully designed to identify outliers and unusual parameters, but every test has its limitations and alerts that are not important in a particular case may appear. Conversely, the absence of alerts does not guarantee there are no aspects of the results needing attention. It is up to the individual to critically assess their own results and, if necessary, seek expert advice.

Publication of your CIF in IUCr journals

A basic structural check has been run on your CIF. These basic checks will be run on all CIFs submitted for publication in IUCr journals (*Acta Crystallographica*, *Journal of Applied Crystallography*, *Journal of Synchrotron Radiation*); however, if you intend to submit to *Acta Crystallographica Section C* or *E* or *IUCrData*, you should make sure that full publication checks are run on the final version of your CIF prior to submission.

Publication of your CIF in other journals

Please refer to the *Notes for Authors* of the relevant journal for any special instructions relating to CIF submission.

PLATON version of 05/12/2020; check.def file version of 05/12/2020

Datablock exp_1639 - ellipsoid plot

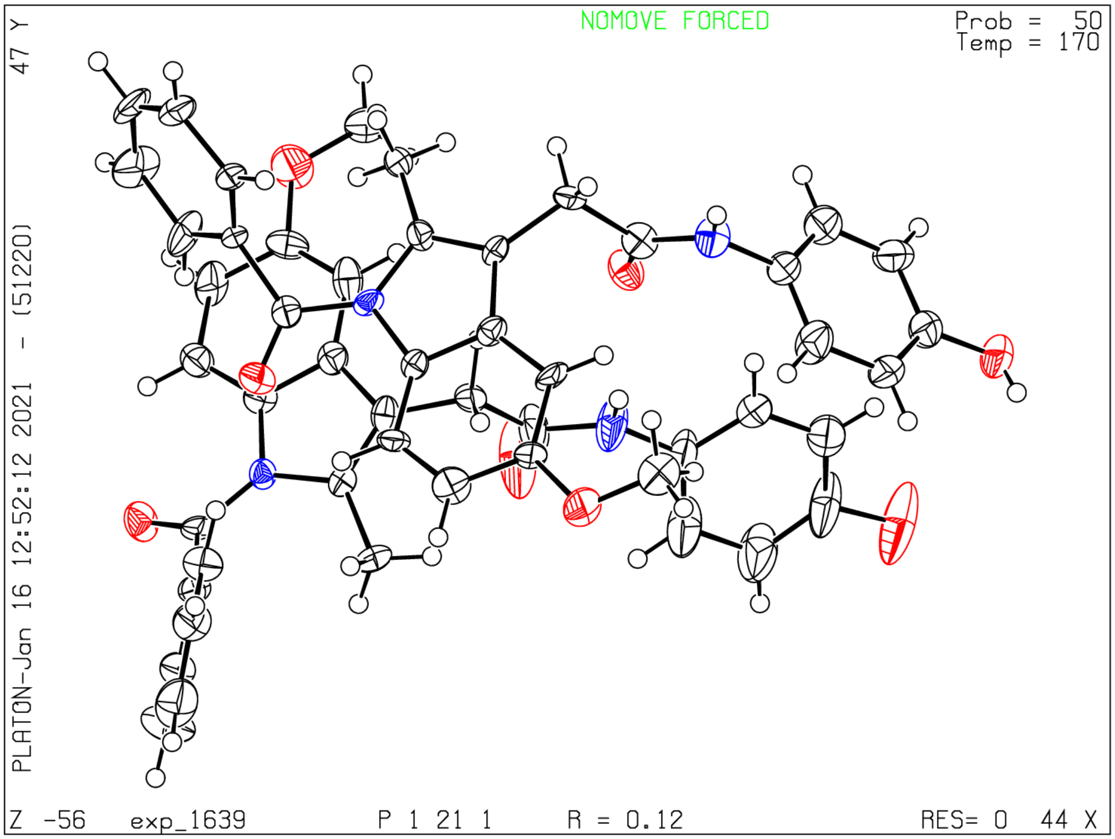

Supplement: nwab156_Supplemental_Files [file nwab156_supplemental_files.zip › X-ray-2av-checkcif.pdf]
